# Design and synthesis of new 1,2,4-oxadiazole/quinazoline-4-one hybrids with antiproliferative activity as multitargeted inhibitors

**DOI:** 10.3389/fchem.2024.1447618

**Published:** 2024-08-30

**Authors:** Amira M. Mohamed, Ola M. F. Abou-Ghadir, Yaser A. Mostafa, Kholood A. Dahlous, Stefan Bräse, Bahaa G. M. Youssif

**Affiliations:** ^1^ Pharmaceutical Organic Chemistry Department, Faculty of Pharmacy, Assiut University, Assiut, Egypt; ^2^ Department of Chemistry, College of Science, King Saud University, Riyadh, Saudi Arabia; ^3^ Institute of Biological and Chemical Systems, IBCS-FMS, Karlsruhe Institute of Technology, Karlsruhe, Germany

**Keywords:** quinazolinone, oxadiazole, kinases, apoptosis, antiproliferative, EGFR, BRAF

## Abstract

**Introduction:**

The combination of BRAF and tyrosine kinase (TK) inhibitors has been demonstrated to be highly effective in inhibiting tumor development and is an approach for overcoming resistance in clinical trials. Accordingly, a novel series of 1,2,4-oxadiazole/quinazoline-4-one hybrids was developed as antiproliferative multitargeted inhibitors.

**Methods:**

The structures of the newly synthesized compounds 9a-o were validated using IR, NMR, MS, and elemental techniques. 9a–o were tested as antiproliferative agents.

**Results and Discussion:**

The results showed that the majority of the tested compounds showed significant antiproliferative action with 9b, 9c, 9h, 9k, and 9l being the most potent. Compounds 9b, 9c, 9h, 9k, and 9l were tested as EGFR and BRAF^V600E^ inhibitors. These *in vitro* tests revealed that compounds 9b, 9c, and 9h are strong antiproliferative agents that may act as dual EGFR/BRAF^V600E^ inhibitors. 9b, 9c, and 9h were further investigated for their inhibitory effect on mutant EGFR (EGFR^T790M^), and the results showed that the tested compounds had considerable inhibitory action. Cell cycle study and apoptosis detection demonstrated that compound 9b exhibits cell cycle arrest at the G2/M transition. Molecular docking simulations reveal the binding mechanism of the most active antiproliferative agents.

## 1 Introduction

Drug developers have spent decades generating selective medicines for specific targets (Medina-Franco et al., 2013; Zhou et al., 2019). Despite the effectiveness of many single-target selective medications, the advancement of multifactorial disorders such as cancer and neurological diseases included many signaling pathways (Fu et al., 2017; Raghavendra et al., 2018). As a result, there is a growing interest in developing treatments that address many targets at the same time.

There are currently two opposing methodologies for designing multi-targeting medicines. The first technique involves establishing an additive or synergistic effect of various medications operating on separate targets through combination drug therapy. Preclinical evidence of enhanced apoptosis and delayed resistance to BRAF (Rapidly Accelerated Fibrosarcoma, B-family) inhibitors (Paraiso et al., 2010; Flaherty et al., 2012), for example, prompted the FDA to approve a combination of dabrafenib (BRAF inhibitor) and trametinib (MEK inhibitor) for the treatment of metastatic melanoma with BRAF mutations (Robert et al., 2015; Wahid et al., 2018). The use of palbociclib and letrozole in the treatment of advanced breast cancer is another example of successful combination therapy (Finn et al., 2016).

The second approach is designing and generating multiple-targeting medicines that synergistically block numerous carcinogenic pathways (Keith et al., 2005; Boran and Iyengar, 2010). The method of multi-targeting therapies is finding a single agent that can operate on two or more targets simultaneously. Cabozantinib, also known as cabometyx, was approved by the FDA as a small molecule dual-targeting inhibitor of the tyrosine kinases c-Met (mesenchymal-epithelial transition factor) and VEGFR-2 (Vascular Endothelial Growth Factor Receptor) and has been demonstrated to suppress tumor growth, metastasis, and angiogenesis (Food and Administration, 1997).

**FIGURE 1:**
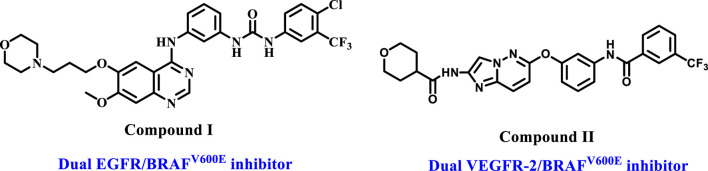
Structures of compounds I and II.

On the other hand, the combination of BRAF and tyrosine kinase (TK) inhibitors has been demonstrated to be highly effective in inhibiting tumor development and is an approach for overcoming resistance in clinical trials. Vemurafenib (BRAF^V600E^ inhibitor) resistance in thyroid cancer can be addressed by combining it with EGFR (Epidermal Growth Factor Receptor) inhibitors (Notarangelo et al., 2017). This combination has also shown promising results in BRAF^V600E^ colorectal cancer (Mondaca et al., 2018). In addition, various compounds have been discovered *in vitro* that include the key pharmacophoric groups required to inhibit tyrosine kinases, such as EGFR/VEGFR-2 and BRAF (Okaniwa et al., 2012; Zhang et al., 2013). Compound I (Figure 1) inhibited wild-type BRAF and EGFR with IC_50_ values in the nanomolar range. Additionally, imidazo [1,2-b]pyridazine II inhibited BRAF and VEGFR-2.

Heterocyclic moieties form the largest and most varied class of organic molecules. In medicinal chemistry, compounds containing heterocyclic nuclei have gained great interest because of their diverse therapeutic actions (Padwa and Bur, 2007). Heterocyclics play a crucial role in the breakdown of all living things and participate in various biochemical processes necessary for life (Kitadai and Maruyama, 2018). The heteroaromatic framework resembles biologically active compounds such as nucleic acids, hormones, and neurotransmitters (Meanwell, 2017). As a result, these moieties could be used to design safer medications. Heterocycles are often found in nature and have been exploited to develop anti-cancer drugs that target many sites and disrupt cancer growth pathways (Sharma et al., 2017). Heterocyclic rings can be modified with various substituents to cover various chemicals, making them ideal for designing anti-cancer drugs.

**FIGURE 2:**
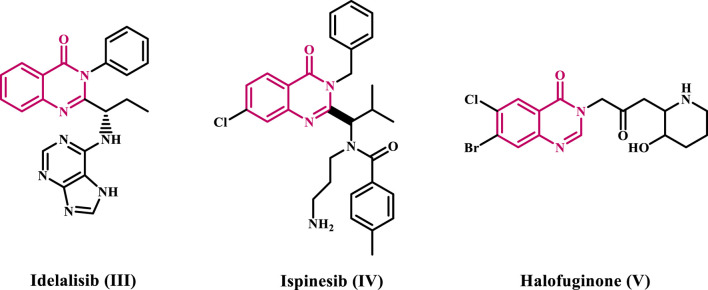
Examples of approved or commercialized anti-cancer medicines with the quinazoline-4-one scaffold.

Nitrogen-containing heterocyclic chemicals significantly affect about 75% of FDA-approved anti-cancer drugs (Kerru et al., 2020). Quinazolinone, a bicyclic system composed of benzene and pyrimidinone rings, is one of the most common nitrogen-containing heterocycles in medicinal chemistry, found in various compounds with diverse biological activity. Idelalisib III (Do et al., 2016), Ispinesib IV (Purcell et al., 2010), and Halofuginone V (Figure 2) (Derbyshire et al., 2012; McLaughlin et al., 2014) are examples of recently approved or marketed medications with anti-cancer properties.

**FIGURE 3:**
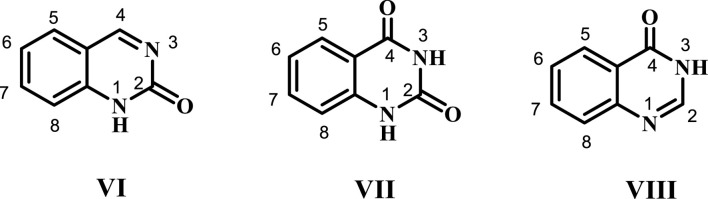
Different forms of quinazolinones.

Depending on the position of the keto or oxo group, three distinct forms are possible: quinazolin-2(1*H*)-one VI, quinazoline-2,4-(1*H*,3*H*)-di-one VII, and quinazolin-4(3*H*)-one VIII (Figure 3). Among these, quinazolin-4-one VIII is the most commonly used scaffold in synthetic processes or as a structural component of natural compounds (Sharma et al., 2017). This last scaffold is adaptable, allowing up to six potential substitutes in positions 2, 3, 5, 6, 7, and 8.

**FIGURE 4:**
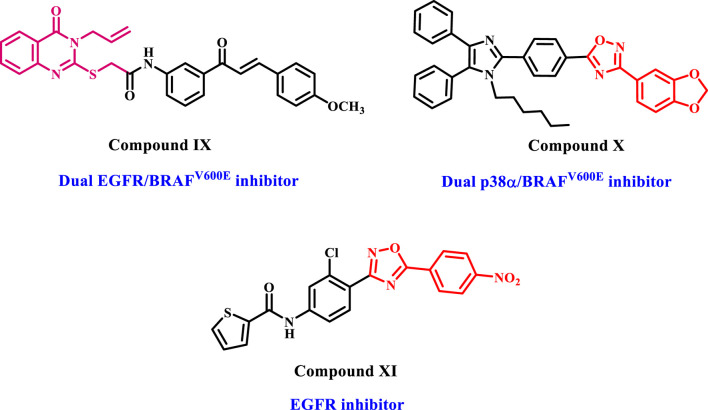
Structures of quinazoline-4-one and 1,2,4-oxadiazole-based derivatives as anticancer agents.

In recent publications (Hisham et al., 2022; Hisham et al., 2023), we present the design and synthesis of a new series of quinazoline-4-one/chalcone hybrids that function as dual inhibitors of EGFR and BRAF^V600E^ with antiproliferative activity. The target compounds were tested *in vitro* against various cancer cell lines and the EGFR and BRAF^V600E^ enzymes. Compound IX (Figure 4) was the most potent derivative, with a GI_50_ of 1.16 µM, compared to the reference drug Doxorubicin (GI_50_ = 1.14 µM). Compound IX showed significant inhibitory activity against EGFR and BRAF^V600E^, with IC_50_ values of 0.11 µM and 0.65 µM, respectively. Moreover, apoptosis assay results revealed that compound IX enhanced the level of active caspase-3, 8, and 9 with significant induction of cytochrome c and Bax levels and downregulation of the anti-apoptotic Bcl-2 levels.

On the other hand, literature reviews reveal that 1,2,4-oxadiazoles have statistical significance in bioorganic and medicinal chemistry. They are recognized for their diverse pharmacological characteristics (Benassi et al., 2020; El Mansouri et al., 2020; Loboda et al., 2020). The 1,2,4-oxadiazole exhibits bioisosteric equivalence with ester and amide moieties. When unstable conditions (e.g., hydrolysis) are identified, 1,2,4-oxadiazole is a highly effective alternative (Hendawy, 2022). The substantial biological impact of 1,2,4-oxadiazole derivatives on cancer cells can be attributed to various mechanisms of action. For example, we developed and synthesized novel 1,2,4-oxadiazole-based derivatives linked to a triaryl-imidazole moiety, with compound X (Figure 4) being the most potent (Youssif et al., 2022). *In vitro* studies assessed the antiproliferative effects of recently identified compounds inhibiting p38α and BRAF^V600E^. These compounds showed effective antiproliferative and kinase inhibition.

Another set of 1,2,4-oxadiazole-based compounds (compound XI, Figure 4) were designed, synthesized, and tested for antiproliferative properties against EGFR-TK. The experiment showed promising antiproliferative effects against cancer cell lines, with low micromolar IC_50_ values against EGFR, compared to the reference doxorubicin (Unadkat et al., 2021).

### 1.1 Rational design

**FIGURE 5:**
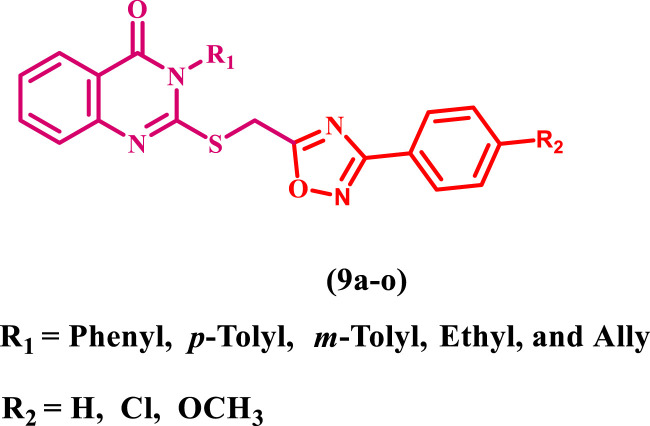
Structures of new targets 9a-o.

Consistent with prior findings and continuing our efforts to develop dual or multitargeted antiproliferative agents (Al-Wahaibi et al., 2020; Alshammari et al., 2022; Al-Wahaibi et al., 2023a; Abdel-Aziz et al., 2023; Al-Wahaibi et al., 2023b; Al-Wahaibi et al., 2023c; Al-Wahaibi et al., 2023d; Al-Wahaibi et al., 2023e), this study’s strategy was to design and synthesize new antiproliferative agents based on quinazoline-4-one/1,2,4-oxadiazole hybrids (Figure 5) to obtain new anti-tumor agents with synergistic activity.

The substitutions on the nitrogen atom of the quinazoline moiety were changed between alkyl (methyl, ethyl, and allyl) and aryl (phenyl or tolyl) moieties to examine the impact of rigidity on the antiproliferative activity of these compounds. In addition, different substituents, such as a chlorine atom (electron withdrawing group) or a methoxy group (electron donating group), were used to investigate the different electronic impacts of these substituents on 9a-o’s antiproliferative activity.

All of the novel analogs were examined for cell viability effect against a normal human mammary gland epithelial (MCF-10A) cell line as well as for antiproliferative activity against four human cancer cell lines: colon (HT-29), pancreatic (Panc-1), lung (A-549), and breast (MCF-7). Furthermore, the compounds with the highest antiproliferative activity were investigated *in vitro* as multi-targeting inhibitors of EGFR, EGFR^T790M^, and BRAF^V600E^ enzymes. The study was expanded to include one of the most active derivatives, 9b, as a representative agent to evaluate its mechanistic effects on the cell cycle and induction of apoptosis. Finally, docking studies were performed on the most active compounds against the selected enzymes to explain their *in vitro* results. Furthermore, the ADME analyses were performed to investigate their pharmacokinetic features.

## 2 Results and discussion

### 2.1 Chemistry

**SCHEME 1:**
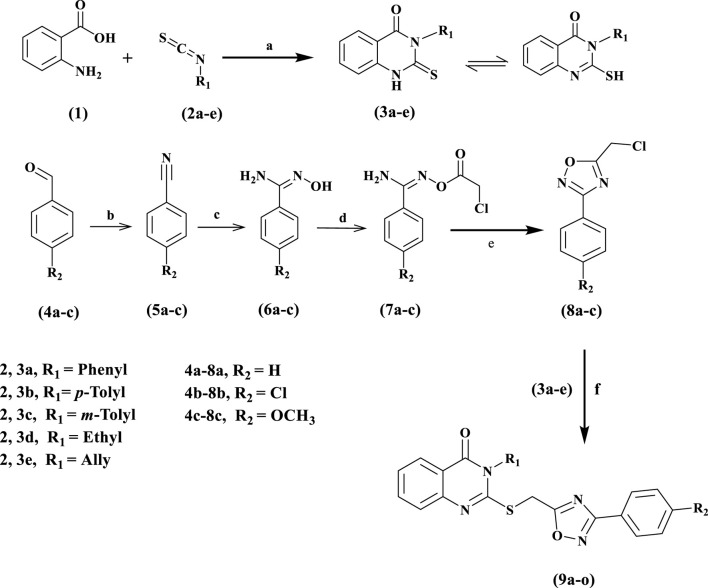
Synthesis of the new target compounds 9a-o

**SCHEME 2:**
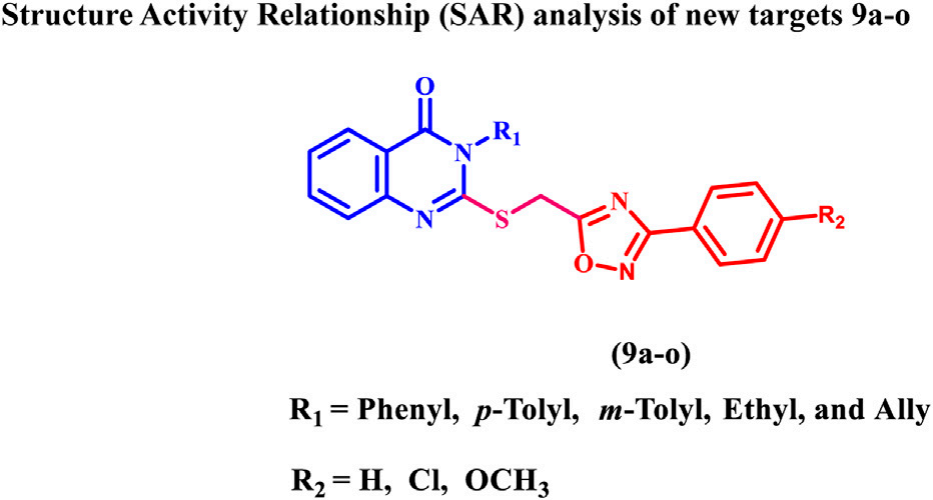
Structure Activity Relationship (SAR) analysis of new targets 9a-o.

Scheme 1 summarizes the synthetic pathways of the new target compounds 9a-o. Anthranilic acid (1) was refluxed in ethanol with isothiocyanate derivatives 2a-e for 8 h. After the reaction was completed (as determined by TLC), the resulting white precipitate was collected by filtration and recrystallized from an ethanol/dioxane mixture (1:1) to give the corresponding quinazoline derivatives 3a-e in 90%–95% yields (Moussa et al., 2018). On the other hand, compounds 6a-c, amidoxime derivatives, were synthesized in 50%–60% yields over two steps, Scheme 2. The first step involved reacting the corresponding aldehydes 4a-c with 28% aqueous ammonia and iodine in THF for 2–3 h to yield the corresponding aryl nitrile derivatives 5a-c in 76%–80% (Yan et al., 2017). The second step was a 12- to 18-h methanol reflux of compounds 5a-c with hydroxylamine chloride and sodium carbonate (Youssif et al., 2019). Compounds 6a-c were reacted with chloroacetyl chloride in dry acetone to yield benzimidamides (7a-c), which were cyclized by refluxing in toluene to the corresponding 3-aryl-5-(chloromethyl)-1,2,4-oxadiazole derivatives 8a-c as a yellow oil. Compounds 8a-c were purified using column chromatography with hexane: ethyl acetate (9:1) as an eluent (Minin et al., 2023). For example, the ^1^H NMR spectrum of compound 8b confirmed the disappearance of two protons from the NH_2_ group of the corresponding amidoxime 7b. Moreover, the spectrum displayed a singlet signal corresponding to the methylene protons (Cl-CH_2_) at δ 4.74. The spectra also revealed a characteristic pair of doublets in the aromatic region for 4-ClC_6_H_4_ at δ 8.01 and 7.46.

Reagents and Conditions: a) Triethylamine, ethanol, Reflux 8 h; b) ammonia (28%), I_2_, THF, Stirring 1h; c) NH_2_OHHCl, Na_2_CO_3_, THF, Reflux 18 h; d) Chloroacetyl Chloride, K_2_CO_3_, Dry acetone, Stirring 24hrs; e) Toluene, Reflux 10 h; f) K_2_CO_3_, KI, DMF, Stirring 24 h.

Finally, the target novel compounds, 9a-o, were synthesized in high yields by coupling compounds 3a-e with the corresponding 1,2,4-oxadiazoles 8a-c in DMF using anhydrous K_2_CO_3_ and KI and stirring for 24 h at room temperature. 9a-o were purified via ethanol recrystallization. The structures of 9a-o were elucidated using ^1^H NMR, ^13^C NMR, and elemental microanalyses. The ^1^H NMR spectra of compound 9l, as an example, confirmed the presence of ethyl group characteristic signals in the form of triplet at δ 1.31 (t, *J* = 7.1 Hz, 3H, N-CH_2_CH_3_) and quartet at δ 4.11 (q, *J* = 7.1 Hz, 2H, N-CH_2_). The spectrum also revealed two distinct singlet signals: at δ 3.81 (s, 3H, OCH_3_) and at δ 4.91 (s, 2H, S-CH_2_). Additionally, the spectrum revealed a pair of doublets of the aromatic ring’s *para* di substitution pattern and extra signals for the aromatic protons in the quinazoline moiety. The ^13^C NMR spectrum of 9l indicated the presence of ethyl group characteristic signals at δ 39.56 and δ 13.01, methylene group at δ 26.62, and methoxy group at δ 55. Elemental microanalysis of 9l confirmed that the calculated data (%) were C, 60.90; H, 4.60; N, 14.20; S, 8.13, while the found data (%) were C, 61.13; H, 4.74; N, 14.37; S, 8.20.

### 2.2 Biology

#### 2.2.1 Assay of cell viability effect

The human mammary gland epithelial (MCF-10A) normal cell line was used to test the viability of novel compounds 9a-o (Mahmoud et al., 2022; Mekheimer et al., 2022). After 4 days of incubation on MCF-10A cells, the cell viability of compounds 9a-o was determined using the MTT test. Table 1 demonstrates that none of the compounds examined were cytotoxic, and all hybrids showed more than 89% cell viability at 50 µM.

**TABLE 1:** Cell viability percent and antiproliferative activity (IC_50_ values) of compounds 9a-o.

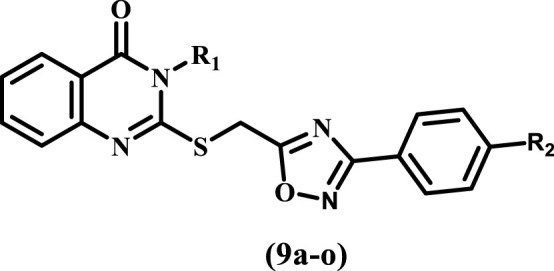								
Comp	Cell viability %	R_1_	R_2_	Antiproliferative activity IC_50_ ± SEM (nM)				
				A-549	MCF-7	Panc-1	HT-29	Average (GI_50_)
9a	90	Phenyl	H	46 ± 4	50 ± 4	48 ± 4	48 ± 4	48
9b	89	Phenyl	Cl	22 ± 2	26 ± 2	24 ± 2	24 ± 2	24
9c	91	Phenyl	OMe	24 ± 2	28 ± 3	26 ± 2	25 ± 2	26
9d	90	*p-*Tolyl	H	54 ± 4	58 ± 5	54 ± 5	55 ± 5	55
9e	91	*p-*Tolyl	Cl	40 ± 3	44 ± 4	42 ± 4	42 ± 4	42
9f	92	*p-*Tolyl	OMe	50 ± 4	53 ± 5	52 ± 5	52 ± 5	52
9g	90	*m-*Tolyl	H	49 ± 4	52 ± 5	50 ± 5	50 ± 5	50
9h	89	*m-*Tolyl	Cl	28 ± 2	31 ± 3	29 ± 2	30 ± 3	30
9i	91	*m-*Tolyl	OMe	65 ± 6	69 ± 6	66 ± 6	68 ± 6	67
9j	90	Ethyl	H	69 ± 6	76 ± 7	68 ± 6	68 ± 6	70
9k	93	Ethyl	Cl	32 ± 3	35 ± 3	34 ± 3	34 ± 3	34
9l	90	Ethyl	OMe	36 ± 3	40 ± 4	38 ± 3	37 ± 3	38
9m	92	Allyl	H	62 ± 6	65 ± 6	64 ± 6	62 ± 6	63
9n	91	Allyl	Cl	43 ± 4	47 ± 4	45 ± 4	44 ± 4	45
9o	89	Allyl	OMe	56 ± 4	59 ± 5	57 ± 5	57 ± 5	57
Erlotinib	ND	—	—	30 ± 3	40 ± 3	30 ± 3	30 ± 3	33

ND: not determined.

#### 2.2.2 Assay of antiproliferative action

The MTT assay was used to investigate the antiproliferative activity of hybrids 9a-o versus four human cancer cell lines using Erlotinib as a control: colon cancer (HT-29) cell line, pancreatic cancer (Panc-1) cell line, lung cancer (A-549) cell line, and breast cancer (MCF-7) cell line (El-Sherief et al., 2019; Al-Wahaibi et al., 2022). Table 1 displays the median inhibitory concentration (IC_50_) and GI_50_ (average IC_50_) against the four cancer cell lines.

In general, the hybrids 9a-o had significant antiproliferative action with GI_50_ values ranging from 24 nM to 70 nM versus the four cancer cell lines evaluated, compared to Erlotinib, which had a GI_50_ value of 33 nM. Compounds 9b, 9c, 9h, 9k, and 9l were the most potent five derivatives, with GI_50_ values of 24, 26, 30, 34, and 38 nM, making 9b, 9c, and 9h more potent than Erlotinib (GI_50_ = 33 nM). Out of all the newly synthesized hybrids 9a-o, compound 9b (R_1_ = phenyl, R_2_ = Cl) had the highest potency, with a GI_50_ value of 24 nM, which was 1.4 times more potent than the reference Erlotinib (GI_50_ = 33 nM).

The type of the aryl/alkyl moieties at position 3 of the quinazoline moiety appears to be critical for 9a-o hybrids antiproliferative activity. The GI_50_ value of compound 9h (R_1_ = *m*-tolyl, R_2_ = Cl) was 30 nM, less potent than compound 9b but still more potent than the reference erlotinib (GI_50_ = 33 nM).

Moreover, Compounds 9e (R_1_ = *p*-tolyl, R_2_ = Cl), 9k (R_1_ = ethyl, R_2_ = Cl), and 9n (R_1_ = allyl, R_2_ = Cl) demonstrated GI_50_ values of 42, 34, and 45 nM, respectively, being less potent than compounds 9b, 9h, and even Erlotinib. These results indicated the importance of the quinazoline moiety position three substitution pattern on antiproliferative activity, with activity rising in the following order: phenyl > *m*-tolyl > *p*-tolyl > ethyl > allyl.

Compound 9c (R_1_ = phenyl, R_2_ = OMe) rated second in activity against the four cancer cell lines, with a GI_50_ value of 26 nM, slightly less effective than 9b but still more potent than Erlotinib (GI_50_ = 33 nM). The unsubstituted phenyl derivative, 9a (R_1_ = phenyl, R_2_ = H), was less potent than 9b and 9c, indicating that the substitution pattern at the fourth position of the phenyl group in the 1,2,4-oxadiazole moiety affects the antiproliferative activity of these hybrids, with activity increasing in the order Cl > OMe > H. Regardless of the nature of the substitution pattern at position 3 of the quinazoline moiety, the same rule (Cl > OMe > H in activity) applies to other derivatives.

#### 2.2.3 EGFR inhibitory assay

The EGFR-TK test (Abdel-Aziz et al., 2023) was used to assess the inhibitory potency of the most effective antiproliferative derivatives 9b, 9c, 9h, 9k, and 9l against EGFR, and the results are shown in Table 2. This assay’s results are in line with the antiproliferative assay’s, which found that compounds 9b (R_1_ = phenyl, R_2_ = Cl), 9c (R_1_ = phenyl, R_2_ = OMe), and 9h (R = *m*-tolyl, R_2_ = Cl), the most effective antiproliferative hybrids, were the most potent derivatives of EGFR inhibitors, with IC_50_ values of 57 ± 4 nM, 64 ± 5 nM, and 72 ± 5 nM, respectively, surpassing the reference drug Erlotinib (IC_50_ = 80 ± 5). Compounds 9k (R_1_ = ethyl, R_2_ = Cl) and 9l (R_1_ = ethyl, R_2_ = OMe) demonstrated significant anti-EGFR activity, with IC_50_ values of 84 ± 6 nM and 91 ± 07 nM, respectively, which were less potent than Erlotinib. These findings show that compounds 9b, 9c, and 9h have significant EGFR inhibitory action and could be used as antiproliferative agents.

**TABLE 2:** IC_50_ values of compounds 9b, 9c, 9h, 9k, and 9l against EGFR, BRAF^V600E^ and EGFR^T790M^.

Compound	EGFR inhibition IC_50_ ± SEM (nM)	BRAF^V600E^ inhibition IC_50_ ± SEM (nM)	EGFR^T790M^ inhibition IC_50_ ± SEM (nM)
9b	57 ± 4	48 ± 4	10 ± 1
9c	64 ± 5	51 ± 5	11 ± 2
9h	72 ± 5	57 ± 5	15 ± 2
9k	84 ± 6	62 ± 5	ND
9l	91 ± 7	70 ± 5	ND
Erlotinib	80 ± 5	60 ± 5	ND
Vemurafenib	ND	30 ± 3	ND
Osimertinib	ND	ND	8 ± 1

ND: not determined.

#### 2.2.4 BRAF^V600E^ inhibitory assay

An *in vitro* investigation assessed the anti-BRAF^V600E^ activity of 9b, 9c, 9h, 9k, and 9l (Youssif et al., 2022). The enzyme assay demonstrated that the five hybrids examined substantially inhibited BRAF^V600E^, with IC_50_ values ranging from 48 to 70 nM, Table 2. In all cases, the IC_50_ of the examined compounds is greater than that of the reference Vemurafenib (IC_50_ = 30). Compounds 9b, 9c, and 9h demonstrated the most effective inhibitory activity against BRAF^V600E^ (IC_50_ = 48, 51, and 57 nM, respectively) and were discovered to be potent inhibitors of cancer cell growth (GI_50_ = 24, 26, and 30 nM, respectively). As a result, compounds 9b, 9c, and 9h are effective antiproliferative agents that function as dual EGFR/BRAF^V600E^ inhibitors.

#### 2.2.5 EGFR^T790M^ inhibitory assay

The HTRF KinEASE-TK assay (Miles et al., 2020) was used to evaluate the inhibitory action of the most potent hybrids, 9b, 9c, and 9h, against mutant-type EGFR (EGFR^T790M^). As demonstrated in Table 2, compounds 9b, 9c, and 9h displayed excellent inhibitory effect against EGFR^T790M^, with IC_50_ values of 10 ± 1, 11 ± 1, and 15 ± 1 nM, respectively, being equivalent to the reference Osimertinib (IC_50_ = 8 ± 1 nM), which may explain their robust antiproliferative activity. These findings suggested that phenyl and *m*-tolyl substitutions in the quinazoline moiety’s third position, as well as chlorine atom and methoxy substitutions in the *para*-position of the phenyl group in the 1,2,4-oxadiazole moiety, are required for the inhibitory impact on EGFR, BRAF^V600E^, and EGFR^T790M^.

1. The type of the aryl/alkyl moieties at position 3 of the quinazoline moiety appears to be critical for 9a-o hybrids antiproliferative activity, with activity increasing in the following order: phenyl > *m*-tolyl > *p*-tolyl > ethyl > allyl.
2. The substitution pattern at the fourth position of the phenyl group in the 1,2,4-oxadiazole moiety affects the antiproliferative activity of these hybrids as well as EGFR, BRAF^V600E^, and EGFR^T790M^ inhibition, with activity increasing in the order Cl > OMe > H.
3. Regardless of the nature of the substitution pattern at position 3 of the quinazoline moiety, the same rule (Cl > OMe > H in activity) applies to other derivatives.

#### 2.2.6 Cell cycle analysis and apoptosis assays

##### 2.2.6.1 Cell cycle analysis

**FIGURE 6:**
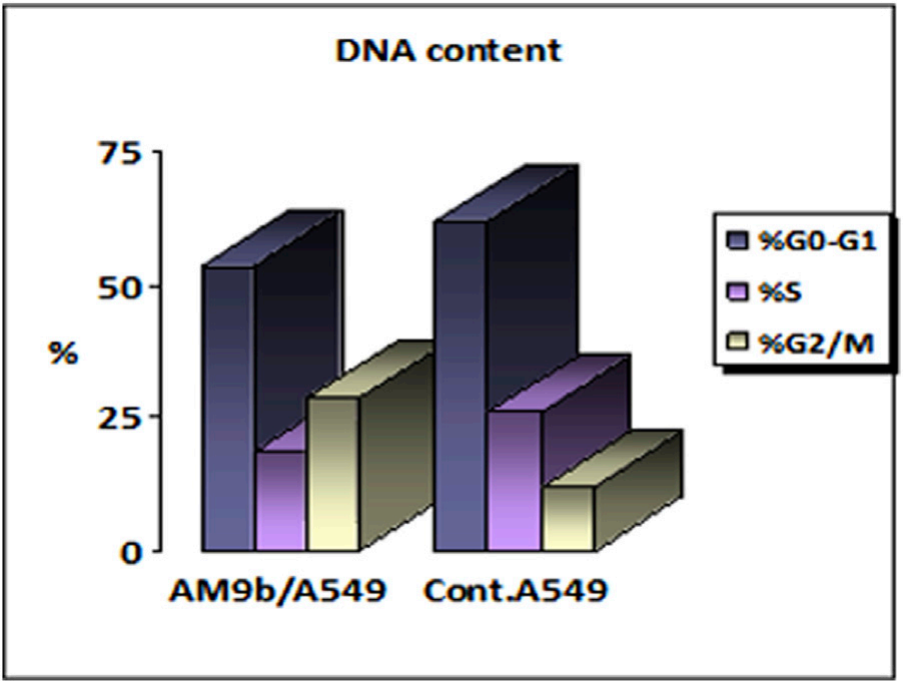
Cell cycle analysis results for compound 9b.

Compound 9b was investigated for its effects on cell cycle progression and apoptosis induction in A-549 cells. A lung cancer (A-549) cell line was treated for 24 h with an IC_50_ concentration of 9b. The cell line was labeled with PI/Annexin V, and flow cytometry was done with a BD FASC Caliber (El-Sherief et al., 2018). The results (Figure 6) showed that A-549 treated with compound 9b had a significant percentage of cell accumulation (29%) in the G2/M phase after 24 h of incubation, indicating cell cycle arrest at the G2/M transition.

##### 2.2.6.2 Apoptosis induction assay

**FIGURE 7:**
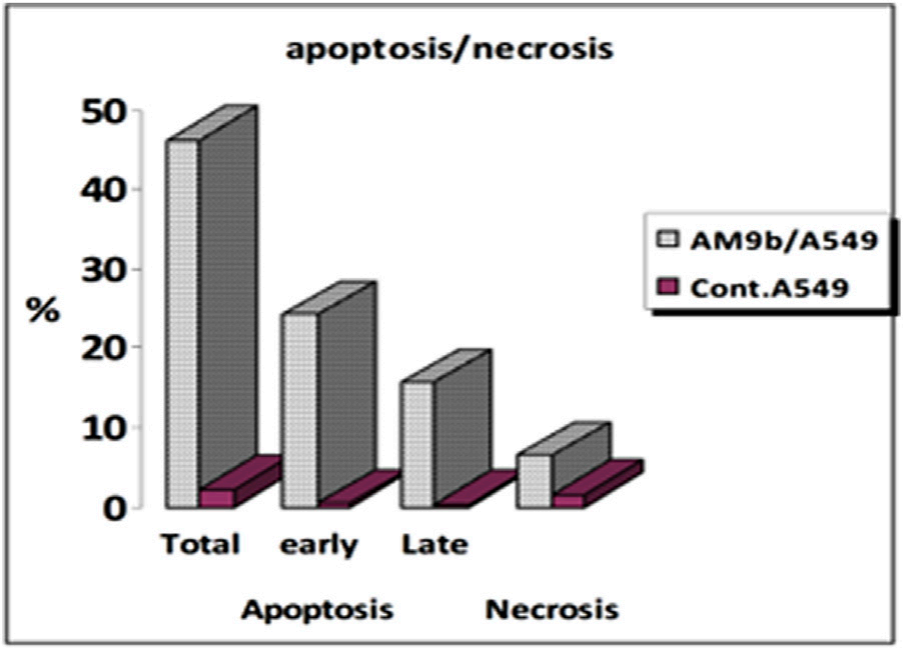
Apoptosis induction results of compound 9b.

**FIGURE 8:**
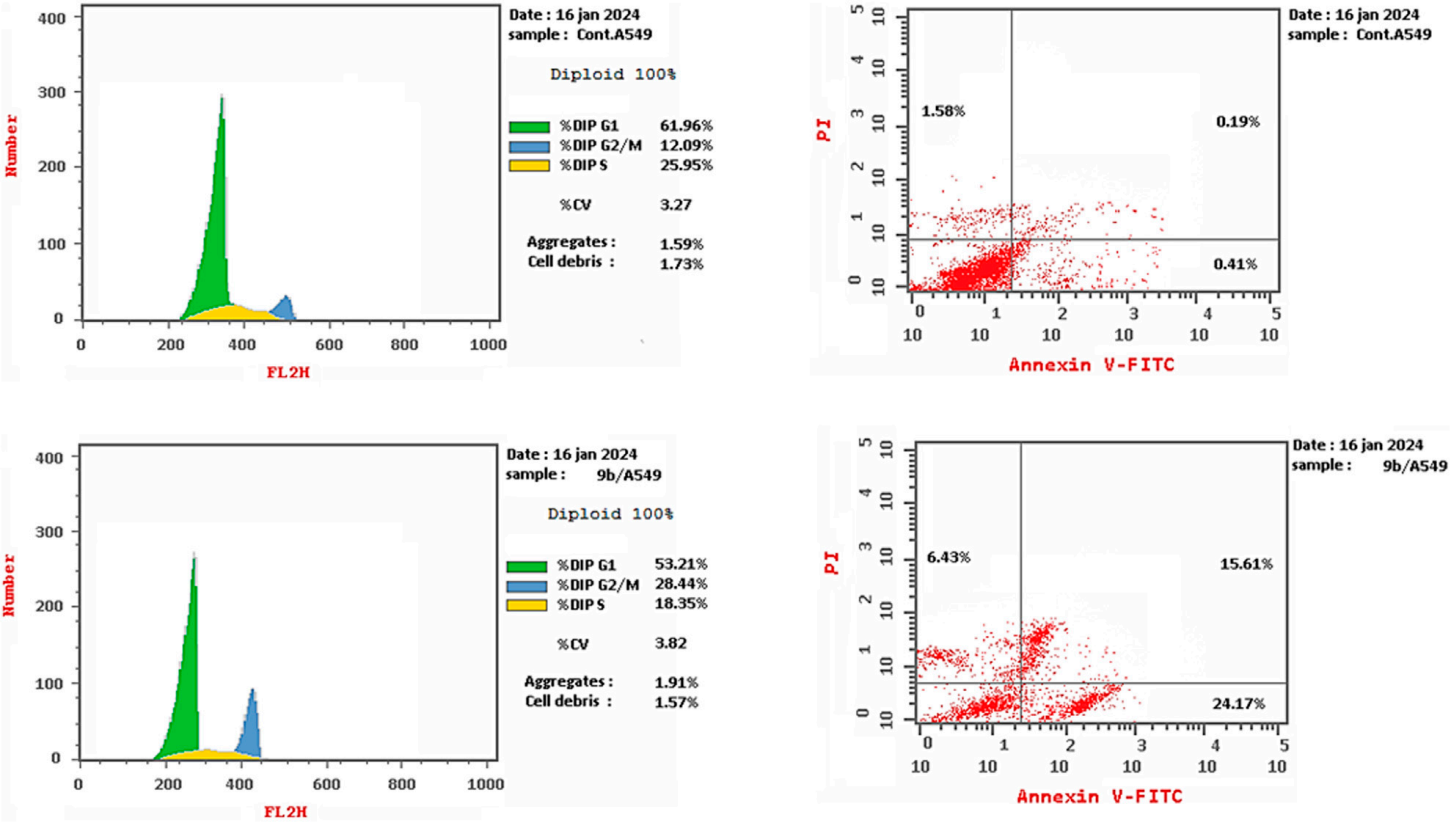
Cell cycle analysis and apoptosis induction results of compound 9b.

To assess 9b′s potential to induce apoptosis, A-549 cells were labeled with Annexin V/PI, grown for 24 h, and evaluated. Examining early and late apoptosis demonstrated that compound 9b could produce high levels of apoptosis, with a necrosis percentage 6.43 (Figures 7, 8).

### 2.3 Docking simulations

Starting with RSCB deposited crystal structure of EGFR protein having Erlotinib as a co-crystallized ligand (PDB: 1M17) (Bhat et al., 2022), and re-docking of Erlotinib revealed a docking score (S) of −7.30 kcal/mol and an RMSD of 1.28 Å, in addition to formation of the two characteristic H-bond interactions with two of key amino acid residues, Gln767 and Met769, indicating validity of docking study parameters. While running docking simulations within EGFR active site (PDB ID: 1M17) showed that most of the test derivatives (9a-o) showed moderate to strong binding interactions (S = −5.93 to −7.52; c.f. Erlotinib: 7.30 kcal/mol) as listed in Table 3. These interactions were variable between H-bond and/or H-pi, with key amino acid residues (Met 769, Lys 721, Gly 772, and Leu 694) lining the active site, as shown in Supplementary Figure S1 (Supplementary Material).

**TABLE 3:** Oxadiazoles docking scores in EGFR, EGFR^T790M^, and BRAF^V600E^ active sites.

Comp	R_1_	R_2_	Docking score (S; kcal/mol)		
			EGFR (1M17)	EGFR^T790M^ (2JIU)	BRAF^V600E^ (5JRQ)
9a	Phenyl	H	−6.0118	−7.1900	−7.0458
9b	Phenyl	Cl	−6.5073	−7.4268	−7.0985
9c	Phenyl	OMe	−6.0388	−7.2876	−7.2278
9d	*p-*Tolyl	H	−6.0314	−6.7971	−6.9232
9e	*p-*Tolyl	Cl	−6.4310	−5.6713	−7.3078
9f	*p-*Tolyl	OMe	−6.8251	−6.7228	−6.9971
9g	*m-*Tolyl	H	−7.5281	−6.8538	−6.8646
9h	*m-*Tolyl	Cl	−6.2685	−6.4894	−6.8470
9i	*m-*Tolyl	OMe	−6.8622	−6.7373	−7.6516
9j	Ethyl	H	−6.6348	−6.9894	−6.2829
9k	Ethyl	Cl	−5.7431	−6.3041	−6.5824
9l	Ethyl	OMe	−5.9343	−6.1808	−6.7029
9m	Allyl	H	−6.1516	−7.2311	−6.8390
9n	Allyl	Cl	−6.2599	−6.3498	−6.8475
9o	Allyl	OMe	−6.7193	−6.7397	−6.8732
Erlotinib			−7.3034	—	—
Osimertinib			—	−8.5638	—
Vemurafenib			—	---	−9.3319

**FIGURE 9:**
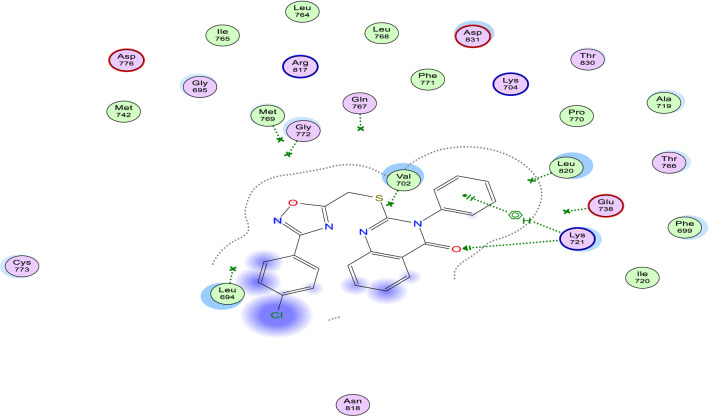
Binding Interactions of 9b within EGFR (PDB ID: 1M17) active site showing both H-bonds and H-Pi interactions as green-dotted arrows and lines, respectively.

Remarkably, derivative 9b (R_1_ = Ph. and R_2_ = Cl) showed a higher docking score (S = −6.51 kcal/mol) over its methoxy congener (S = −6.04 kcal/mol) and has the best docking score over other *p*-chloro derivatives (9e, 9h, and 9k). Visual inspection of the docking pose with the lowest RMSD value and highest docking score of compound 9b, we observed a stabilizing H-bonding and H-pi binding interactions through N-phenyl and amidic carbonyl group of quinazoline ring with Lys721 amino acid residue, Figure 9. Such interactions were not found in other *p*-chloro derivatives, 9e and 9k (except with derivative 9h), because of the hydrophilic tale of Gln738 amino acid residue, which repels closely found non-hydrophilic groups as methyl group of 9e and ethyl group of 9k, as shown in Supplementary Figure S1.

**FIGURE 10:**
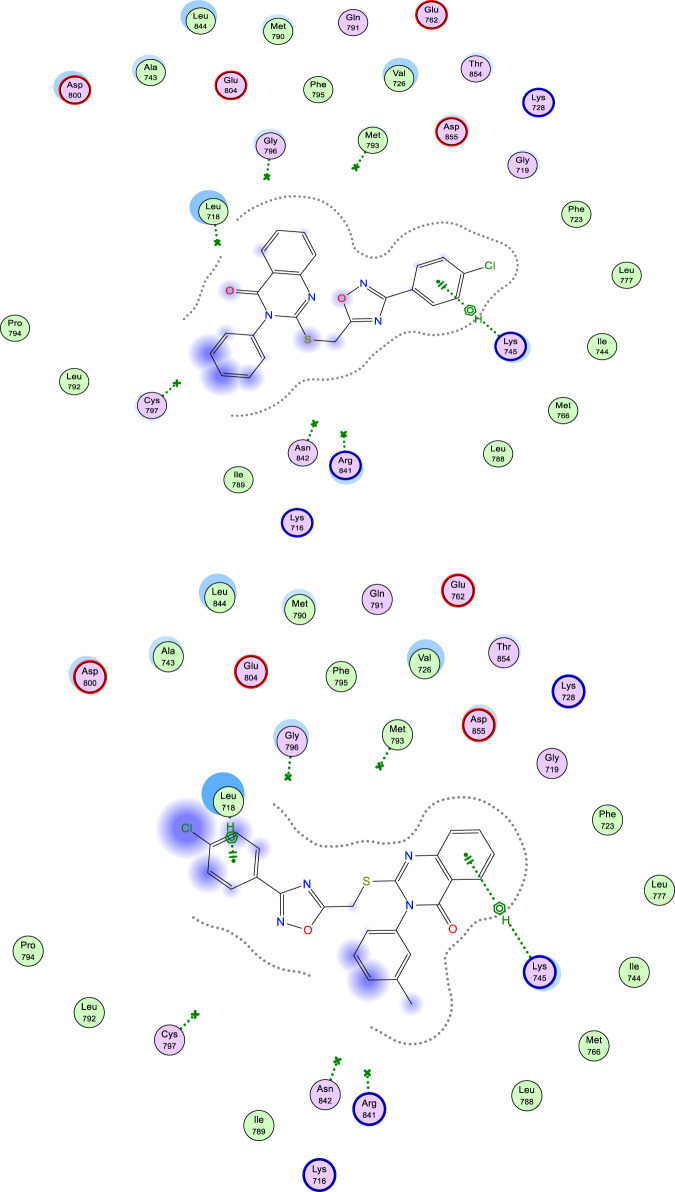
Docking poses of compound 9b (top figure) and 9h (bottom figure) within the active site of EGFR^T790M^ (PDB ID: 2JIU) showing H-Pi interactions with key amino acid residues.

Whereas upon working on binding interactions within mutant EGFR (EGFR^T790M^; PDB ID: 2JIU) active site, the *p*-Cl derivative (9b) gave the highest docking score (S = −7.43 kcal/mol) among all 15 derivatives tested, as shown in Table 3. Most test derivatives commonly interacted through H-bonds and/or H-pi interactions with Lys 745 and Leu 718, as seen with docking poses of derivatives 9b and 9h, Figure 10.

**FIGURE 11:**
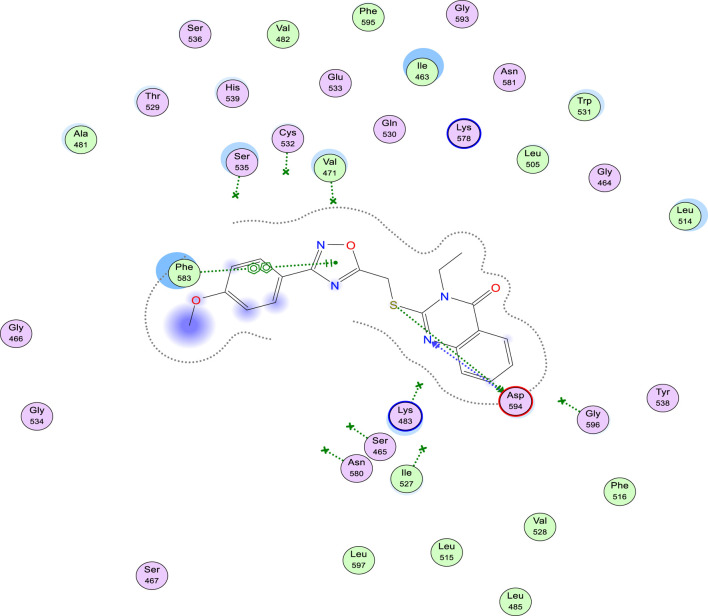
2D-Binding interactions of compound 9l within BRAF^V600E^ (PDB ID: 5JRQ) active site showing H-bonds (as blue-arrows) and pi-pi interactions (as green-dotted lines) with Asp 594 and Phe 583, respectively.

Finally, docking scores of derivatives (9a-o) interactions within BRAF (PDB ID: 5JRQ) (Umar et al., 2020) active site were high and so close to each other (S = −6.24 to −7.65 kcal/mol). Additionally, multiple interactions varying from the weak Pi-Pi interactions, through H-Pi to the strong H-bonds with either Phe 583, Val 471, Asp 594, or Lys 483, as shown in Figure 11 of compound 9l.

To summarize, all the 15 derivatives showed good binding profiles within target proteins EGFR, mutant EGFR (EGFR^T790M^), and BRAF^V600E,^ as seen from their docking scores and interactions with amino acid residues lining their active sites, and this could be used to explain the possible mechanism by which such class of compounds inhibit these proteins activity.

### 2.4 Calculation of ADME properties

The drug-likeness of new compounds 9a-o was calculated using the SwissADME website (Daina et al., 2017) to predict their transport properties through membranes like GIT and/or BBB. All the test compounds obey Lipinski’s rule of five (RO5) with MLogP below 5, in addition to having both a topological polar surface area below 140 Å^2^ and molar refractivity below 130, indicating their facile transport through cell membranes and hence better oral bioavailability (F), as shown in Table 4.

**TABLE 4:** Theoretical calculations of ADME properties of compounds (9a-o) using swissADME software.

Cpd	MW	Lipinski Parameters^a^					F	MR	Water Solubility Silicos-IT class	GI absorption	BBB
		HBA	HBD	nrotb	TPSA	MLogP					
9a	412.46	5	0	5	99.11	3.6	0.55	116.84	Poor	High	No
9b	446.91	5	0	5	99.11	4.08	0.55	121.85	Poor	High	No
9c	442.49	6	0	6	108.34	3.28	0.55	123.33	Poor	High	No
9d	426.49	5	0	5	99.11	3.81	0.55	121.81	Poor	High	No
9e	460.94	5	0	5	99.11	4.29	0.55	126.82	Poor	High	No
9f	456.52	6	0	6	108.34	3.49	0.55	128.3	Poor	High	No
9g	426.49	5	0	5	99.11	3.81	0.55	121.81	Poor	High	No
9h	460.94	5	0	5	99.11	4.29	0.55	126.82	Poor	High	No
9i	456.52	6	0	6	108.34	3.49	0.55	128.3	Poor	High	No
9j	364.42	5	0	5	99.11	2.92	0.55	101.57	Poor	High	No
9k	398.87	5	0	5	99.11	3.15	0.55	106.58	Poor	High	No
9l	394.45	6	0	6	108.34	2.35	0.55	108.06	Poor	High	No
9m	376.43	5	0	6	99.11	3.08	0.55	105.9	Poor	High	No
9n	410.88	5	0	6	99.11	3.3	0.55	110.91	Poor	High	No
9o	406.46	6	0	7	108.34	2.5	0.55	112.4	Poor	High	No

MW, molecular weight; HBA, H-bond acceptor; HBD, H-bond donor; nrotb, no. of rotatable bonds; TPSA, topological polar surface area (Å^2^); MLogP, n-octanol/water distribution coefficient; F, Abbott bioavailability scores (0–1).

^a^ Drug lead-like character: MW ≤ 500, HBA ≤10, HBD ≤5, nrotb ≤10, TPSA ≤140, lipophilicity parameter MLogP ≤5; MR, 40–130; BBB, Blood-Brain Barrier.

## 3 Conclusion

A novel set of quinazoline-4-one/1,2,4-oxadiazole hybrids (9a-o) was designed and synthesized as EGFR, EGFR^T790M^, and BRAF^V600E^ inhibitors in the search for multitargeted antiproliferative scaffold. The novel hybrids showed encouraging antiproliferative actions. Compounds 9b, 9c, 9h, 9k, and 9l were evaluated as EGFR and BRAF^V600E^ inhibitors. These *in vitro* experiments demonstrated that compounds 9b, 9c, and 9h are potent antiproliferative agents capable of acting as dual EGFR/BRAF^V600E^ inhibitors. 9b, 9c, and 9h were further studied for their inhibitory effect on mutant EGFR (EGFR^T790M^), with the results indicating that the evaluated compounds had a significant inhibitory effect. Cell cycle analysis and apoptosis induction assay of 9b revealed cell cycle arrest at the G2/M phase, which can induce apoptosis. EGFR and BRAF^V600E^ docking simulations inside their active regions shed light on these compounds’ possible modes of inhibition. ADME calculations revealed that all test compounds satisfy Lipinski’s rule of five (RO5) with MLogP <5, with easy transport through cell membranes and higher oral bioavailability. These new hybrids may have potential as anti-cancer drugs after optimization.

## 4 Experimental

### 4.1 Chemistry

#### 4.1.1 General details

The starting materials, quinazolines 3a-e (Moussa et al., 2018) and 3-aryl-5-(chloromethyl)-1,2,4-oxadiazole derivatives, compounds 8a-c (Minin et al., 2023), were prepared according to literature methods.

#### 4.1.2 General procedures for the synthesis of compounds (9a-o)

To a stirred solution of quinazoline derivatives (0.60 mmol, 1 eq), compounds 3a-e, in DMF (5 mL), anhydrous K_2_CO_3_ (0.72 mmol, 1.20 eq, 0.10 g) was added and stirred for 1h at room temperature. Then, 3-aryl-5-(chloromethyl)-1,2,4-oxadiazole derivatives, compounds 8a-c, (0.60 mmol, 1 eq.) was added, and KI (0.60 mmol, 1 eq, 0.10 g) was also added to the reaction mixture and stirring was continued for 24 h. After completion of the reaction (checked by TLC using Hexane: Ethyl acetate 2:1), the reaction mixture was poured into crushed ice while stirring. The obtained precipitate was filtered off, washed several times with water, dried at 60°C, and crystallized from ethanol.

#### 4.1.3 2-((3-Phenyl-1,2,4-oxadiazol-5-yl)methylthio)-3-phenylquinazolin-4(3*H*)-one (9a)

Yield: 0.21 g (85%), White solid, m.p: 162°C–164°C, *R*_*f*_. 0.66 (Hexane: Ethyl acetate, 2:1, v/v). ^1^H NMR (400 MHz, *δ* ppm DMSO-*d*_6_): 8.07 (d, *J* = 7.7 Hz, 1H, Ar-H), 7.99 (d, *J* = 6.4 Hz, 2H, Ar-H), 7.81 (t, *J* = 7.4 Hz, 1H, Ar-H), 7.60 (d, *J* = 11.0 Hz, 4H, Ar-H), 7.57–7.52 (m, 4H, Ar-H), 7.47 (t, *J* = 8.0 Hz, 2H, Ar-H), 4.79 (s, 2H, S-CH_2_); ^13^C NMR (100 MHz, *δ* ppm DMSO*-d*_6_): 177.09, 167.84, 160.56, 155.46, 146.85, 135.49, 135.01, 131.59, 130.22, 129.67, 129.46, 129.25, 126.98, 126.61, 126.32, 126.03, 125.90, 119.60, 26.97; Anal. Calc. (%) For C_23_H_16_N_4_O_2_S: C, 66.97; H, 3.91; N, 13.58; S, 7.77. Found: C, 66.81; H, 3.85; N, 13.82; S, 7.85.

#### 4.1.4 2-((3-(4-Chlorophenyl)-1,2,4-oxadiazol-5-yl)methylthio)-3 phenylquinazolin-4(3*H*)-one (9b)

Yield: 0.24 g (89%), White solid, m.p: 172°C–174°C, *R*_*f*_. 0.67 (Hexane: Ethyl acetate, 2:1, v/v). ^1^H NMR (400 MHz, *δ* ppm DMSO-*d*_6_): 8.07 (dd, *J* = 8.2, 1.5 Hz, 1H, Ar-H), 8.00 (d, *J* = 8.7 Hz, 2H, Ar-H *p*-Cl C_6_H_4_), 7.84–7.79 (m, 1H, Ar-H), 7.63 (d, *J* = 8.7 Hz, 2H, Ar-H *p*-Cl C_6_H_4_), 7.62–7.60 (m, 3H, Ar-H), 7.57–7.52 (m, 2H, Ar-H), 7.51–7.44 (m, 2H, Ar-H), 4.78 (s, 2H, S-CH_2_); ^13^C NMR (100 MHz, *δ* ppm DMSO-*d*_6_): 177.84, 167.52, 161.02, 155.92, 147.31, 136.82, 135.95, 135.47, 130.70, 130.14, 129.93, 129.89, 129.24, 127.08, 126.79, 126.36, 125.33, 120.08, 27.44; Anal. Calc. (%) For C_23_H_15_ClN_4_O_2_S: C, 61.81; H, 3.38; N, 12.54; S, 7.17. Found: C, 61.97; H, 3.50; N, 12.71; S, 7.28.

#### 4.1.5 2-((3-(4-Methoxyphenyl)-1,2,4-oxadiazol-5-yl)methylthio)-3-phenylquinazolin-4(3*H*)-one (9c)

Yield: 0.23 g (88%), White solid, m.p: 186°C–188°C, *R*_*f.*_ 0.65 Hexane: Ethyl acetate, 2:1, v/v). ^1^H NMR (400 MHz, *δ* ppm DMSO-*d*_*6*_): 8.07 (d, *J* = 7.7 Hz, 1H, Ar-H), 7.92 (d, *J* = 8.7 Hz, 2H, Ar-H *p*-OCH_3_ C_6_H_4_), 7.81 (t, *J* = 7.3 Hz, 1H, Ar-H), 7.62 (d, *J* = 3.9 Hz, 3H, Ar-H), 7.59–7.35 (m, 4H, Ar-H), 7.09 (d, *J* = 8.7 Hz, 2H, Ar-H *p*-OCH_3_ C_6_H_4_), 4.76 (s, 2H, S-CH_2_), 3.82 (s, 3H, OCH_3_); ^13^C NMR (100 MHz, *δ* ppm DMSO-*d*_*6*_): 176.64, 167.52, 161.71, 160.54, 155.46, 146.85, 135.48, 134.99, 130.20, 129.64, 129.45, 128.67, 126.60, 126.30, 125.89, 119.60, 118.28, 114.63, 55.36, 26.89; Anal. Calc. (%) For C_24_H_18_N_4_O_3_S: C, 65.14; H, 4.10; N, 12.66; S, 7.25. Found: C, 64.91; H, 4.27; N, 12.89; S, 7.23.

#### 4.1.6 2-((3-Phenyl-1,2,4-oxadiazol-5-yl)methylthio)-3*-p*-tolylquinazolin-4(3*H*)-one (9d)

Yield: 0.22 g (86%), White solid, m.p: 168°C–170°C, *R*_*f*_. 0.69 (Hexane: Ethyl acetate, 2:1, v/v). ^1^H NMR (400 MHz, *δ* ppm DMSO-*d*_6_): 8.06 (d, *J* = 7.5 Hz, 1H, Ar-H), 7.98 (d, *J* = 6.5 Hz, 2H, Ar-H), 7.81 (t, *J* = 7.4 Hz, 3H, Ar-H), 7.59–7.52 (m, 4H, Ar-H), 7.47 (d, *J* = 6.1 Hz, 1H, Ar-H), 7.39 (q, *J* = 8.2 Hz, 2H, Ar-H), 4.76 (s, 2H, S-CH_2_), 2.42 (s, 3H, CH_3_); ^13^C NMR (100 MHz, *δ* ppm DMSO-*d*_6_): 177.12, 167.83, 160.62, 155.78, 146.87, 139.99, 135.01, 132.86, 131.63, 130.16, 129.29, 129.18, 126.99, 126.63, 126.32, 126.03, 125.89, 119.60, 26.96, 20.89; Anal. Calc. (%) For C_24_H_18_N_4_O_2_S: C, 67.59; H, 4.25; N, 13.14; S, 7.52. Found: C, 67.34; H, 4.43; N, 13.40; S, 7.68.

#### 4.1.7 2-((3-(4-Chlorophenyl)-1,2,4-oxadiazol-5-yl)methylthio)-3-*p*-tolylquinazolin-4(3*H*)-one (9e)

Yield: 0.25 g (90%), White solid, m.p: 156°C–158°C, *R*_*f*_. 0.70 (Hexane: Ethyl acetate, 2:1, v/v). ^1^H NMR (400 MHz, *δ* ppm DMSO-*d*_6_): 8.05 (d, *J* = 6.9 Hz, 1H, Ar-H), 7.98 (d, *J* = 7.5 Hz, 2H, Ar-H *p*-Cl C_6_H_4_), 7.80 (t, *J* = 6.4 Hz, 1H, Ar-H), 7.62 (d, *J* = 7.5 Hz, 2H, Ar-H *p*-Cl C_6_H_4_), 7.45 (d, *J* = 7.5 Hz, 2H, Ar-H), 7.42–7.31 (m, 4H, Ar-H), 4.76 (s, 2H, S-CH_2_), 2.42 (s, 3H, CH_3_); ^13^C NMR (100 MHz, *δ* ppm DMSO-*d*_6_): 177.42, 167.06, 160.62, 155.76, 146.86, 140.00, 136.36, 135.02, 132.84, 130.17, 129.47, 129.17, 128.80, 126.62, 126.34, 125.89, 124.87, 119.59, 26.99, 20.90; Anal. Calc. (%) For C_24_H_17_ClN_4_O_2_S: C, 62.54; H, 3.72; N, 12.16; S, 6.96. Found: C, 62.37; H, 3.80; N, 12.42; S, 6.89.

#### 4.1.8 2-((3-(4-Methoxyphenyl)-1,2,4-oxadiazol-5-yl)methylthio)-3-*p*-tolylquinazolin-4(3*H*)-one (9f)

Yield: 0.24 g (89%), White solid, m.p: 165°C–167°C, *R*_*f*_. 0.68 (Hexane: Ethyl acetate, 2:1, v/v). ^1^H NMR (400 MHz, *δ* ppm DMSO-*d*_6_): 8.06 (d, *J* = 7.6 Hz, 1H, Ar-H), 7.91 (d, *J* = 8.5 Hz, 2H, Ar-H *p*-OCH_3_ C_6_H_4_), 7.80 (t, *J* = 7.4 Hz, 1H, Ar-H), 7.48 (d, *J* = 7.8 Hz, 2H, Ar-H), 7.39 (q, *J* = 7.7 Hz, 4H, Ar-H), 7.09 (d, *J* = 8.5 Hz, 2H, Ar-H *p*-OCH_3_ C_6_H_4_), 4.74 (s, 2H, S-CH_2_), 3.82 (s, 3H, OCH_3_), 2.42 (s, 3H, CH_3_); ^13^C NMR (100 MHz, *δ* ppm DMSO-*d*_6_): 176.65, 167.54, 161.73, 160.62, 155.74, 146.87, 139.98, 134.97, 132.86, 130.15, 129.17, 128.68, 126.62, 126.29, 125.90, 119.59, 118.30, 114.65, 55.38, 26.90, 20.90; Anal. Calc. (%) For C_25_H_20_N_4_O_3_S: C, 65.77; H, 4.42; N, 12.27; S, 7.02. Found: C, 65.62; H, 4.61; N, 12.41; S, 6.98.

#### 4.1.9 2-((3-Phenyl-1,2,4-oxadiazol-5-yl)methylthio)-3-*m*-tolylquinazolin-4(3*H*)-one (9g)

Yield: 0.22 g (86%), White solid, m.p: 170°C–172°C, *R*_*f*_. 0.69 (Hexane: Ethyl acetate, 2:1, v/v). ^1^H NMR (400 MHz, *δ* ppm DMSO-*d*_6_): 8.06 (dd, *J* = 8.2, 1.4 Hz, 1H, Ar-H), 7.98 (dd, *J* = 7.9, 1.7 Hz, 2H, Ar-H), 7.82–7.78 (m, 1H, Ar-H), 7.59–7.54 (m, 3H, Ar-H), 7.49–7.45 (m, 3H, Ar-H), 7.41 (d, *J* = 7.7 Hz, 1H, Ar-H), 7.31 (d, *J* = 8.7 Hz, 2H, Ar-H), 4.76 (s, 2H, S-CH_2_), 2.40 (s, 3H, CH_3_); ^13^C NMR (100 MHz, *δ* ppm DMSO-*d*_6_): 177.15, 167.86, 160.57, 155.56, 146.87, 139.34, 135.40, 135.07, 131.65, 130.91, 129.65, 129.49, 129.31, 127.01, 126.63, 126.44, 126.38, 126.04, 125.92, 119.60, 26.99, 20.79; Anal. Calc. (%) For C_24_H_18_N_4_O_2_S: C, 67.59; H, 4.25; N, 13.14; S, 7.52. Found: C, 67.36; H, 4.09; N, 13.41; S, 7.60.

#### 4.1.10 2-((3-(4-Chlorophenyl)-1,2,4-oxadiazol-5-yl)methylthio)-3-*m-*tolylquinazolin-4(3*H*)-one (9h)

Yield: 0.25 g (90%), White solid, m.p: 178°C–180°C, *R*_*f*_. 0.70 (Hexane: Ethyl acetate, 2:1, v/v). ^1^H NMR (400 MHz, *δ* ppm DMSO-*d*_6_): 8.09 (d, *J* = 7.7 Hz, 1H, Ar-H), 8.02 (d, *J* = 8.6 Hz, 2H, Ar-H *p-*Cl C_6_H_4_), 7.84 (t, *J* = 8.4 Hz, 1H, Ar-H), 7.66 (d, *J* = 8.6 Hz, 2H, Ar-H *p-*Cl C_6_H_4_), 7.60–7.41 (m, 4H, Ar-H), 7.34 (d, *J* = 10.4 Hz, 2H, Ar-H), 4.79 (s, 2H, S-CH_2_), 2.43 (s, 3H, CH_3_); ^13^C NMR (100 MHz, *δ* ppm DMSO-*d*_*6*_): 177.37, 167.03, 160.50, 155.49, 146.81, 139.28, 136.33, 135.35, 134.99, 130.86, 129.61, 129.43, 129.23, 128.76, 126.58, 126.40, 126.31, 125.86, 124.85, 119.57, 26.95, 20.75; Anal. Calc. (%) For C_24_H_17_ClN_4_O_2_S: C, 62. 54; H, 3.72; N, 12.16; S, 6.96. Found: C, 62.39; H, 3.85; N, 12.40; S, 6.89.

#### 4.1.11 2-((3-(4-Methoxyphenyl)-1,2,4-oxadiazol-5-yl)methylthio)-3*-m*-tolylquinazolin-4(3*H*)-one (9i)

Yield: 0.24 g (89%), White solid, m.p: 194°C–196°C, *R*_*f*_ .0.68 (Hexane: Ethyl acetate, 2:1, v/v). ^1^H NMR (400 MHz, *δ* ppm DMSO-*d*_6_): 8.09 (d, *J* = 7.6 Hz, 1H, Ar-H), 7.95 (d, *J* = 8.7 Hz, 2H, Ar-H *p*-OCH_3_ C_6_H_4_), 7.84 (t, *J* = 7.3 Hz, 1H, Ar-H), 7.59–7.47 (m, 3H, Ar-H), 7.44 (d, *J* = 7.6 Hz, 1H, Ar-H), 7.34 (d, *J* = 10.8 Hz, 2H, Ar-H), 7.12 (d, *J* = 8.7 Hz, 2H, Ar-H *p*-OCH_3_ C_6_H_4_), 4.77 (s, 2H, S-CH_2_), 3.85 (s, 3H, OCH_3_), 2.43 (s, 3H, CH_3_); ^13^C NMR (100 MHz, *δ* ppm DMSO-*d*_6_): 176.66, 167.51, 161.72, 160.52, 155.52, 146.83, 139.28, 135.37, 135.00, 130.85, 129.62, 129.43, 128.67, 126.58, 126.41, 126.31, 125.89, 119.57, 118.28, 114.65, 55.37, 26.90, 20.75; Anal. Calc. (%) For C_25_H_20_N_4_O_3_S: C, 65.77; H, 4.42; N, 12.27; S, 7.02. Found: C, 65.62; H, 4.61; N, 12.49; S, 7.14.

#### 4.1.12 2-((3-Phenyl-1,2,4-oxadiazol-5-yl)methylthio)-3-ethylquinazolin-4*(*3*H*)-one (9j)

Yield: 0.17 g (79%), White solid, m.p: 114°C–116°C, *R*_*f*_. 0.60 (Hexane: Ethyl acetate, 2:1, v/v). ^1^H NMR (400 MHz, *δ* ppm DMSO-*d*_6_): 8.04 (d, *J* = 8.0 Hz, 1H, Ar-H), 7.97 (d, *J* = 6.4 Hz, 2H, Ar-H), 7.74 (t, *J* = 7.4 Hz, 1H, Ar-H), 7.57–7.54 (m, 3H, Ar-H), 7.42 (t, *J* = 7.4 Hz, 1H, Ar-H), 7.35 (d, *J* = 8.4 Hz, 1H, Ar-H), 4.94 (s, 2H, S-CH_2_), 4.11 (q, *J* = 7.0 Hz, 2H, N-CH_2_), 1.31 (t, *J* = 7.0 Hz, 3H, CH_3_); ^13^C NMR (100 MHz, *δ* ppm DMSO-*d*_6_): 177.21, 167.84, 160.13, 154.45, 146.41, 134.73, 131.60, 129.27, 126.95, 126.38, 126.22, 126.00, 125.66, 118.84, 26.70, 13.04; Anal. Calc. (%) For C_19_H_16_N_4_O_2_S: C, 62.62; H, 4.43; N, 15.37; S, 8.80. Found: C, 62.89; H, 4.51; N, 15.62; S, 8.71.

#### 4.1.13 2-((3-(4-Chlorophenyl)-1,2,4-oxadiazol-5-yl)methylthio)-3-ethylquinazolin-4(3*H*)-one (9k)

Yield: 0.20 g (84%), White solid, m.p: 118°C–120°C, *R*_*f*_. 0.62 (Hexane: Ethyl acetate, 2:1, v/v). ^1^H NMR (400 MHz, *δ* ppm DMSO-*d*_6_): 8.04 (dd, *J* = 7.9, 1.0 Hz, 1H, Ar-H), 7.98 (d, *J* = 8.6 Hz, 2H, Ar-H *p-*Cl C_6_H_4_), 7.75–7.72 (m, 1H, Ar-H), 7.60 (d, *J* = 8.6 Hz, 2H, Ar-H *p-*Cl C_6_H_4_), 7.44–7.41 (m, 1H, Ar-H), 7.35 (d, *J* = 8.1 Hz, 1H, Ar-H), 4.94 (s, 2H, S-CH_2_), 4.11 (q, *J* = 7.0 Hz, 2H, N-CH_2_), 1.31 (t, *J* = 7.0 Hz, 3H, CH_3_); ^13^C NMR (100 MHz, *δ* ppm DMSO-*d*_6_): 177.49, 167.05, 160.11, 154.42, 146.39, 136.35, 134.72, 129.43, 128.73, 126.37, 126.22, 125.64, 124.82, 118.83, 39.58, 26.67, 13.03; Anal. Calc. (%) For C_19_H_15_ClN_4_O_2_S: C, 57.21; H, 3.79; N, 14.05; S, 8.04. Found: C, 57.49; H, 3.86; N, 14. 27; S, 8.12.

#### 4.1.14 2-((3-(4-Methoxyphenyl)-1,2,4-oxadiazol-5-yl)methylthio)-3-ethylquinazolin-4*(*3*H*)-one (9l)

Yield: 0.20 g (84%), White solid, m.p: 134°C–136°C, *R*_*f*_. 0.57 (Hexane: Ethyl acetate, 2:1, v/v). ^1^H NMR (400 MHz, *δ* ppm DMSO-*d*_6_): 8.04 (dd, *J* = 8.0, 1.3 Hz, 1H, Ar-H), 7.91 (d, *J* = 8.9 Hz, 2H, Ar-H *p*-OCH_3_ C_6_H_4_), 7.76–7.71 (m, 1H, Ar-H), 7.44–7.40 (m, 1H, Ar-H), 7.37 (d, *J* = 7.6 Hz, 1H, Ar-H), 7.07 (d, *J* = 8.9 Hz, 2H, Ar-H *p*-OCH_3_ C_6_H_4_), 4.91 (s, 2H, S-CH_2_), 4.11 (q, *J* = 7.1 Hz, 2H, N-CH_2_), 3.81 (s, 3H, OCH_3_), 1.31 (t, *J* = 7.1 Hz, 3H, CH_3_); ^13^C NMR (100 MHz, *δ* ppm DMSO-*d*_6_): 176.74, 167.52, 161.71, 160.11, 154.42, 146.40, 134.69, 128.63, 126.35, 126.9, 125.65, 118.83, 118.25, 114.63, 55.35, 39.56, 26.62, 13.01; Anal. Calc. (%) For C_20_H_18_N_4_O_3_S: C, 60.90; H, 4.60; N, 14.20; S, 8.13. Found: C, 61.13; H, 4.74; N, 14.37; S, 8.20.

#### 4.1.15 2-((3-Phenyl-1,2,4-oxadiazol-5-yl)methylthio)-3-allylquinazolin-4(3*H*)-one (9m)

Yield: 0.18 g (80%), White solid, m.p: 110°C–112°C, *R*_*f*_. 0.61(Hexane: Ethyl acetate, 2:1, v/v). ^1^H NMR (400 MHz, *δ* ppm DMSO-*d*_6_): 8.04 (d, *J* = 7.8 Hz, 1H, Ar-H), 7.97 (d, *J* = 6.8 Hz, 2H, Ar-H), 7.75 (t, *J* = 7.5 Hz, 1H, Ar-H), 7.62–7.49 (m, 3H, Ar-H), 7.43 (t, *J* = 7.5 Hz, 1H, Ar-H), 7.37 (d, *J* = 8.1 Hz, 1H, Ar-H), 6.11–5.83 (m, 1H, =CH), 5.24 (d, *J* = 10.4 Hz, 1H, =CH_2_), 5.15 (d, *J* = 17.3 Hz, 1H, =CH_2_), 4.92 (s, 2H, S-CH_2_), 4.72 (d, *J* = 4.0 Hz, 2H, N-CH_2_); ^13^C NMR (100 MHz, *δ* ppm DMSO-*d*_6_): 177.20, 167.84, 160.19, 154.93, 146.44, 134.94, 131.64, 131.25, 129.30, 126.96, 126.53, 126.37, 126.01, 125.74, 118.73, 117.60, 45.97, 26.80; Anal. Calc. (%) For C_20_H_16_N_4_O_2_S: C, 63.81; H, 4.28; N, 14.88; S, 8.52. Found: C, 64.05; H, 4.42; N, 15.17; S, 8.64.

#### 4.1.16 2-((3-(4-Chlorophenyl)-1,2,4-oxadiazol-5-yl)methylthio)-3-allylquinazolin-4(3*H*)-one (9n)

Yield: 0.21 g (85%), White solid, m.p: 115°C–117°C, *R*_*f*_. 0.63 (Hexane: Ethyl acetate, 2:1, v/v). ^1^H NMR (400 MHz, *δ* ppm DMSO-*d*_6_): 8.07 (dd, *J* = 8.0, 1.2 Hz, 1H, Ar-H), 7.99 (d, *J* = 8.7 Hz, 2H, Ar-H *p*-Cl C_6_H_4_), 7.80–7.74 (m, 1H, Ar-H), 7.63 (d, *J* = 8.7 Hz, 2H, Ar-H *p*-Cl C_6_H_4_), 7.49–7.43 (m, 1H, Ar-H), 7.39 (d, *J* = 8.2, 1H, Ar-H), 6.04–5.90 (m, 1H, =CH), 5.26 (dd, *J* = 10.4, 1.2 Hz, 1H, =CH_2_), 5.17 (dd, *J* = 17.2, 1.2 Hz, 1H, =CH_2_), 4.93 (s, 2H, S-CH_2_), 4.74 (d, *J* = 5.1 Hz, 2H, N-CH_2_); ^13^C NMR (100 MHz, *δ* ppm DMSO-*d*_6_): 177.39, 167.02, 160.10, 154.80, 146.37, 136.32, 134.81, 131.19, 129.38, 128.68, 126.47, 126.27, 125.68, 124.80, 118.69, 117.61, 45.92, 26.72; Anal. Calc. (%) For C_20_H_15_ClN_4_O_2_S: C, 58.46; H, 3.68; N, 13.64; S, 7.80. Found: C, 58.70; H, 3.73; N, 13. 91; S, 7. 94.

#### 4.1.17 2-((3-(4-Methoxyphenyl)-1,2,4-oxadiazol-5-yl)methylthio)-3-allylquinazolin 4(3*H*)-one (9o)

Yield: 0.20 g (83%), White solid, m.p: 122°C–124°C, *R*_*f*_. 0.59 (Hexane: Ethyl acetate, 2:1, v/v). ^1^H NMR (400 MHz, *δ* ppm DMSO-*d*_6_): 8.07 (d, *J* = 7.6 Hz, 1H, Ar-H), 7.92 (d, *J* = 8.7 Hz, 2H, Ar-H *p*-OCH_3_ C_6_H_4_), 7.78 (t, *J* = 7.2 Hz, 1H, Ar-H), 7.46 (t, *J* = 7.4 Hz, 1H, Ar-H), 7.40 (d, *J* = 8.4 Hz, 1H, Ar-H), 7.09 (d, *J* = 8.7 Hz, 2H, Ar-H *p*-OCH_3_ C_6_H_4_), 6.01–5.91 (m, 1H, =CH), 5.26 (d, *J* = 10.4 Hz, 1H, =CH_2_), 5.17 (d, *J* = 17.3 Hz, 1H, =CH_2_), 4.91 (s, 2H, S-CH_2_), 4.74 (d, *J* = 4.7 Hz, 2H, 2H, N-CH_2_), 3.82 (s, 3H, OCH_3_); ^13^C NMR (100 MHz, *δ* ppm DMSO-*d*_6_): 176.73, 167.53, 161.73, 160.16, 154.89, 146.43, 134.88, 131.24, 128.64, 126.51, 126.33, 125.74, 118.72, 118.28, 117.58, 114.66, 55.37, 45.93, 26.70; Anal. Calc. (%) For C_21_H_18_N_4_O_3_S: C, 62.05; H, 4.46; N, 13.78; S, 7.89. Found: C, 62.29; H, 4.53; N, 14.02; S, 7.81.

### 4.2 Biology

#### 4.2.1 Assay of cell viability of 9a-o

The human mammary gland epithelial (MCF-10A) normal cell line was used to test the viability of compounds 9a-o (Mahmoud et al., 2022; Mekheimer et al., 2022). Refer to Supplementary Appendix A (Supplementary Material) for more details.

#### 4.2.2 Assay of antiproliferative action

The MTT assay was used to investigate the antiproliferative activity of hybrids 9a-o versus four human cancer cell lines using Erlotinib as a control: colon cancer (HT-29) cell line, pancreatic cancer (Panc-1) cell line, lung cancer (A-549) cell line, and breast cancer (MCF-7) cell line (El-Sherief et al., 2019; Al-Wahaibi et al., 2022). See Supplementary Appendix A for more information.

#### 4.2.3 EGFR inhibitory assay

The EGFR-TK test (Abdel-Aziz et al., 2023) assessed the inhibitory potency of the most effective antiproliferative derivatives 9b, 9c, 9h, 9k, and 9l against EGFR. For more details, see Supplementary Appendix A.

#### 4.2.4 BRAF^V600E^ inhibitory assay

An *in vitro* investigation assessed the anti-BRAF^V600E^ activity of 9b, 9c, 9h, 9k, and 9l (Youssif et al., 2022). Refer to Supplementary Appendix A for more details.

#### 4.2.5 EGFR^T790M^ inhibitory assay

The HTRF KinEASE-TK assay (Miles et al., 2020) was used to evaluate the inhibitory action of the most potent hybrids, 9b, 9c, and 9h, against mutant-type EGFR (EGFR^T790M^). For more details, see Supplementary Appendix A.

#### 4.2.6 Cell cycle analysis and apoptosis detection

Compound 9b was investigated for its effects on cell cycle progression and apoptosis induction in A-549 cells. A lung cancer (A-549) cell line was treated for 24 h with an IC_50_ concentration of 9b. The cell line was labeled with PI/Annexin V, and flow cytometry was done with a BD FASC Caliber (El-Sherief et al., 2018). See Supplementary Appendix A for more details.

### 4.3 Docking study

Molecular docking simulations of 15 derivatives (9a-o) were performed via Molecular Operating Environment (MOE^®^) software according to reported protocols (Abdel-Aziz et al., 2023) within the active site of EGFR tyrosine kinase domain (PDB ID: 1M17), mutant EGFR kinase domain T790M (EGFR^T790M^; PDB ID: 2JIU), and mutant BRAF kinase domain (BRAF^V600E^; PDB ID: 5JRQ) crystals structures downloaded from RSCB protein data bank (https://www.rcsb.org/). For more details, see Supplementary Appendix A.

### 4.4 Calculations of SwissADME

Pharmacokinetics and drug-likeness prediction for all the newly synthesized compounds was performed using the online tool SwissADME predictor software (http://www.swissadme.ch/) made by the Swiss Institute of Bioinformatics.

## Data Availability

Data will be available upon request from the authors.
