# Supplementary material for: Design and synthesis of new 1,2,4-oxadiazole/quinazoline-4-one hybrids with antiproliferative activity as multitargeted inhibitors
Source: Front Chem. 2024 Aug 30;12:1447618. doi: 10.3389/fchem.2024.1447618 (PMC11393688; doi:10.3389/fchem.2024.1447618)
Supplement: Supplementary file 1 [file DataSheet1.PDF]

Supporting Information

Design and synthesis of new 1,2,4-Oxadiazole/Quinazoline-4-one hybrids with antiproliferative activity as multitargeted inhibitors

Amira M. Mohamed[1], Ola F. Abou-Ghadir[1], Yaser A. Mostafa[1], Kholood A. Dahlous[2], Stefan Bräse[3]*, Bahaa G. M. Youssif[1]*

[1]Pharmaceutical Organic Chemistry Department, Faculty of Pharmacy, Assiut University, Assiut 71526, Egypt; [2]Department of Chemistry, College of Science, King Saud University, Riyadh 11451, Saudi Arabia; [3]Institute of Biological and Chemical Systems, IBCS-FMS, Karlsruhe Institute of Technology, 76131 Karlsruhe, Germany

**To whom correspondence should be addressed:*

Bahaa G. M. Youssif, Ph.D. Pharmaceutical Organic Chemistry Department, Faculty of Pharmacy, Assiut University, Assiut 71526, Egypt.

Tel.: (002)-01098294419

E-mail address: bgyoussif2@gmail.com, bahaa.youssif@pharm.aun.edu.eg

Stefan Bräse

Institute of Biological and Chemical Systems, IBCS-FMS, Karlsruhe Institute of Technology, 76131 Karlsruhe, Germany. E-mail: braese@kit.edu

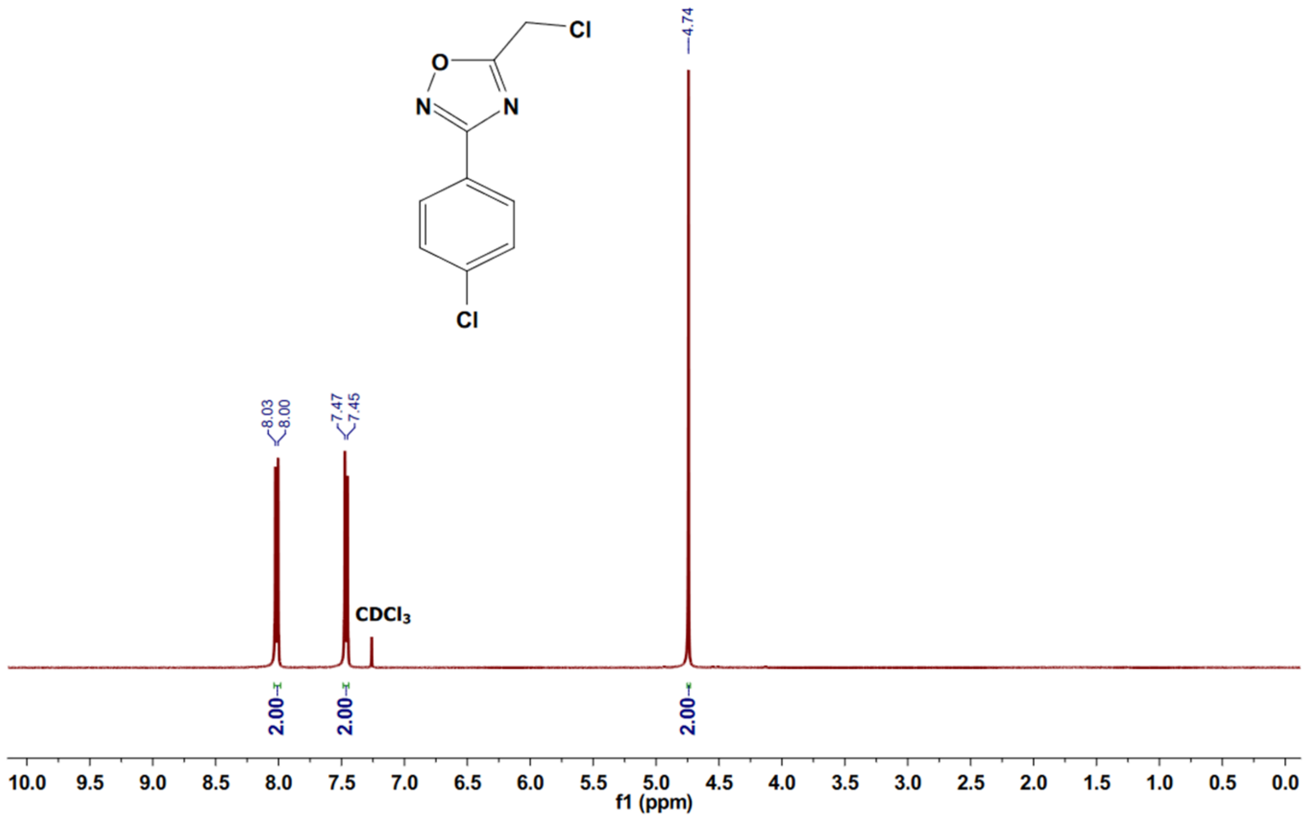

Figure S1: ^{1}H NMR spectrum (400 MHz, $CDCl_3$) of compound **8b**

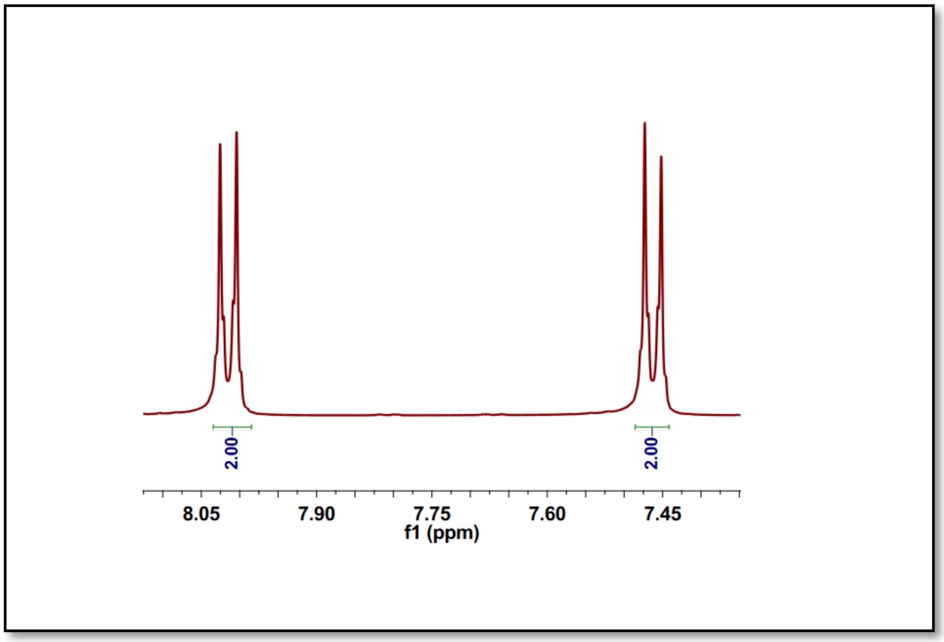

Figure S2: Expanded aromatic region of ^{1}H NMR spectrum (400 MHz, $CDCl_3$) of compound **8b**

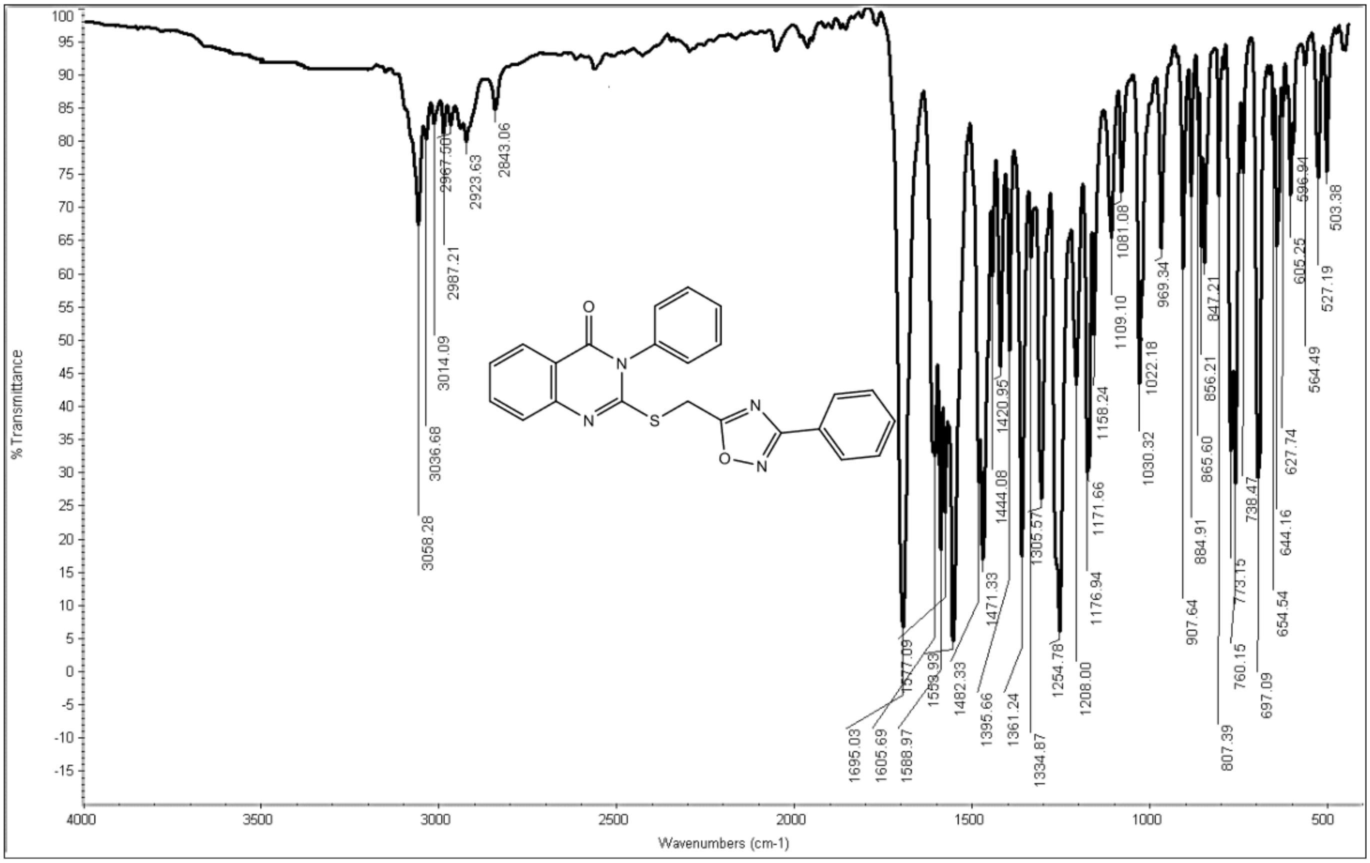

Figure S3: FTIR spectrum of compound **9a**

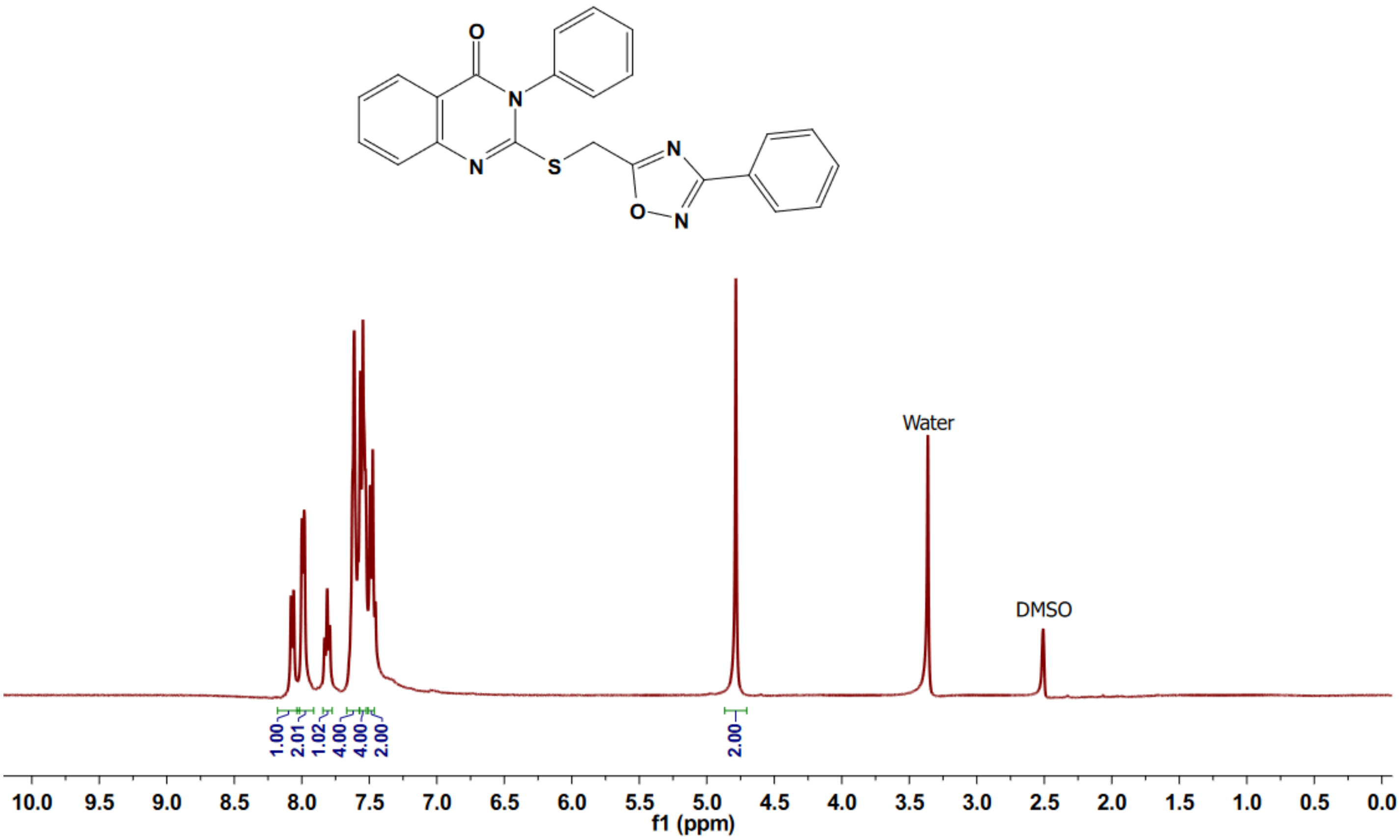

Figure S4: ^{1}H NMR spectrum (400 MHz, DMSO-d_6) of compound **9a**

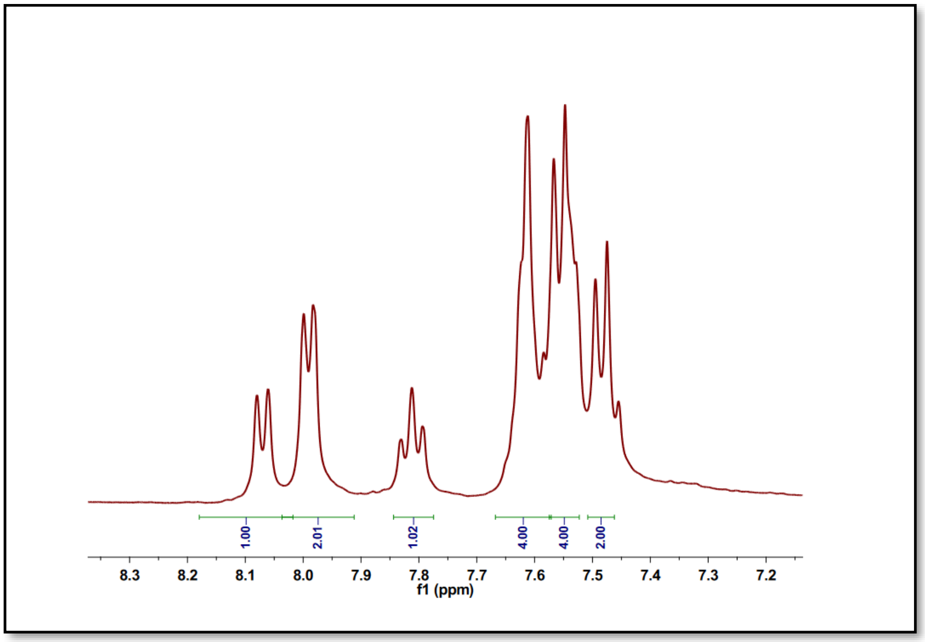

Figure S5: Expanded aromatic region of ^{1}H NMR spectrum (400 MHz, DMSO-d_6) of compound **9a**

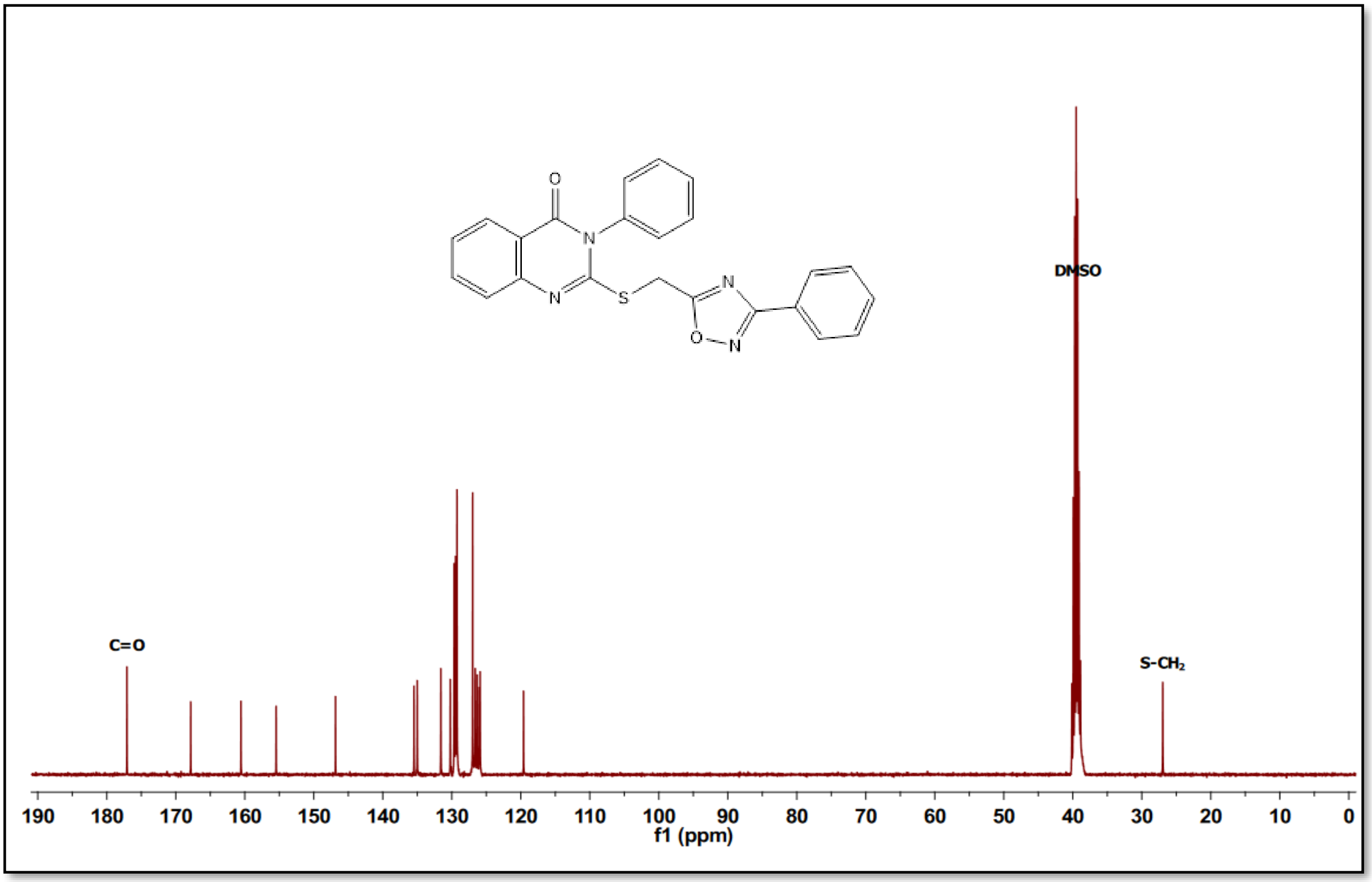

Figure S6: ^{13}C NMR spectrum (100 MHz, DMSO-d_6) of compound **9a**

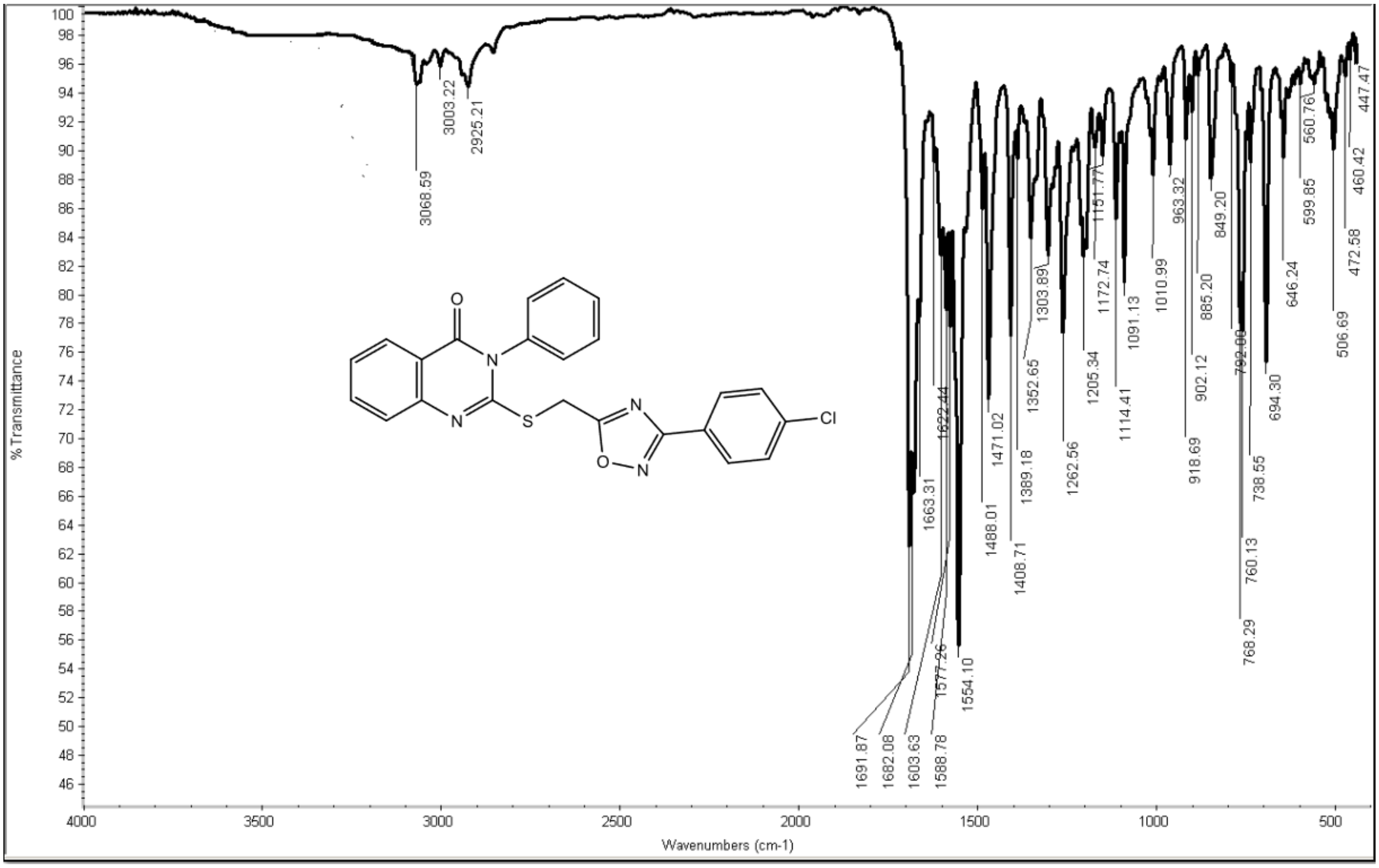

Figure S7: FTIR spectrum of compound **9b**

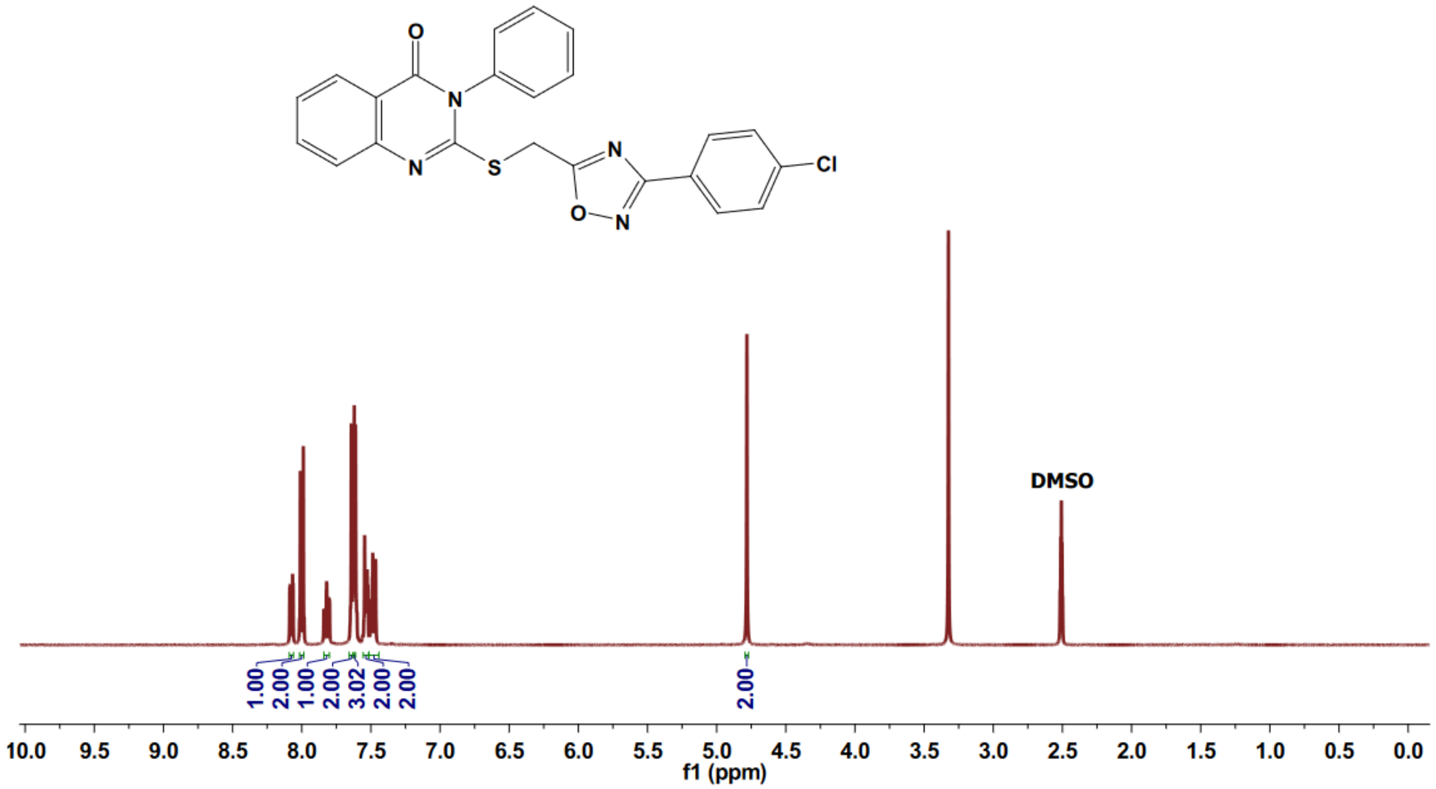

Figure S8: ^{1}H NMR spectrum (400 MHz, DMSO-d_6) of compound **9b**

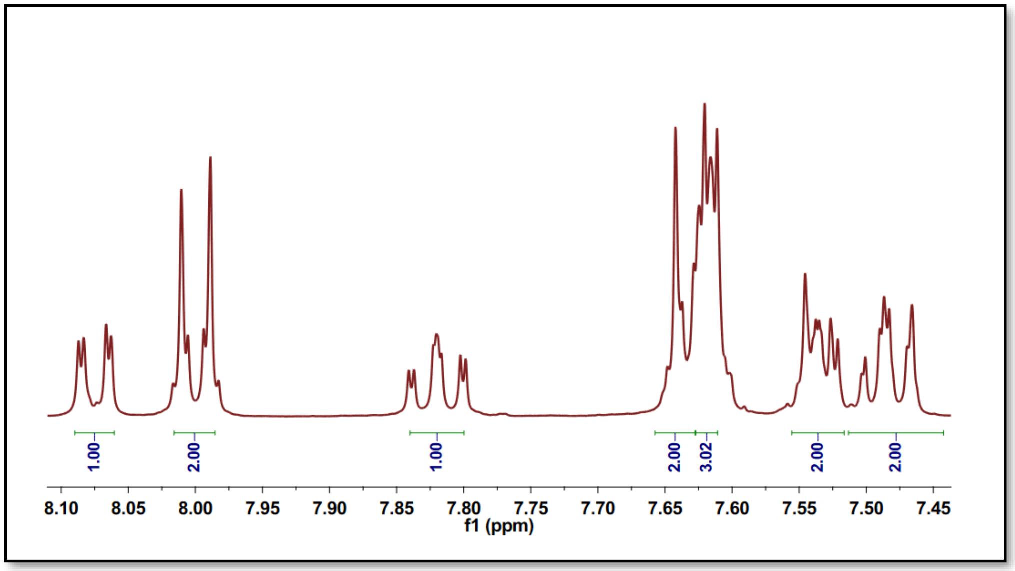

Figure S9: Expanded aromatic region of ^{1}HNMR spectrum (400 MHz, DMSO-d_6) of compound **9b**

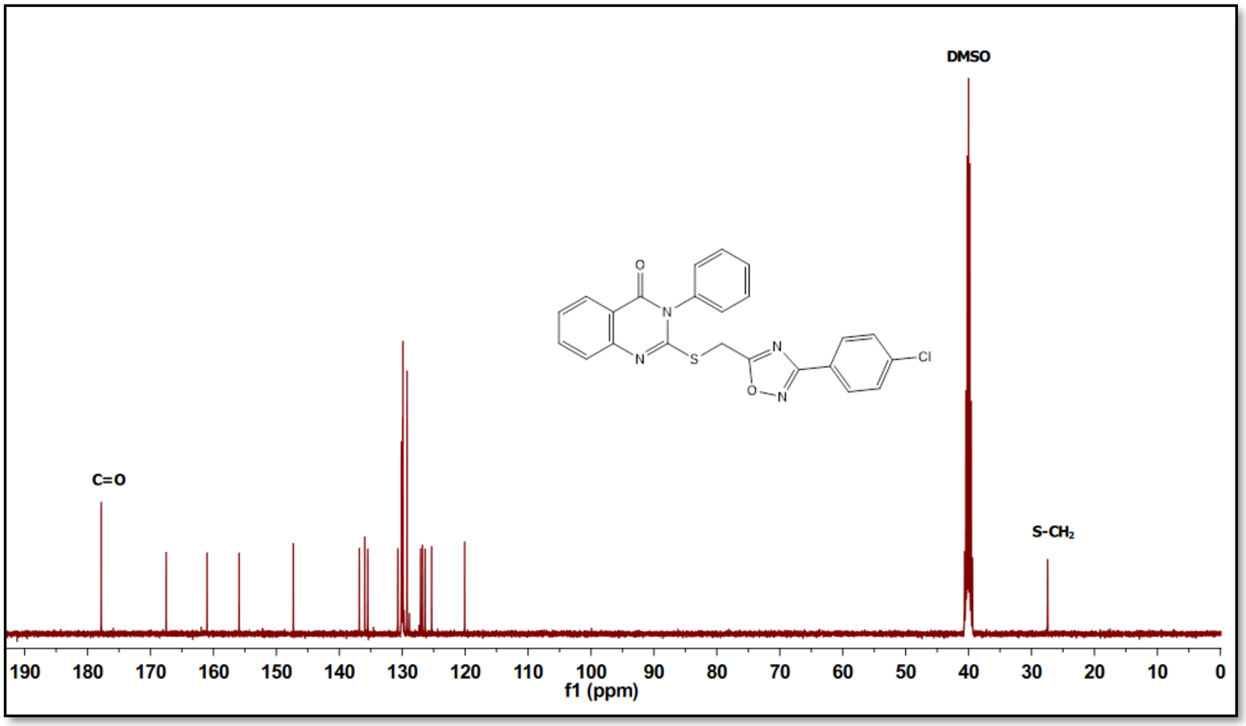

Figure S10: ^{13}C NMR spectrum (100 MHz, DMSO-d_6) of compound **9b**

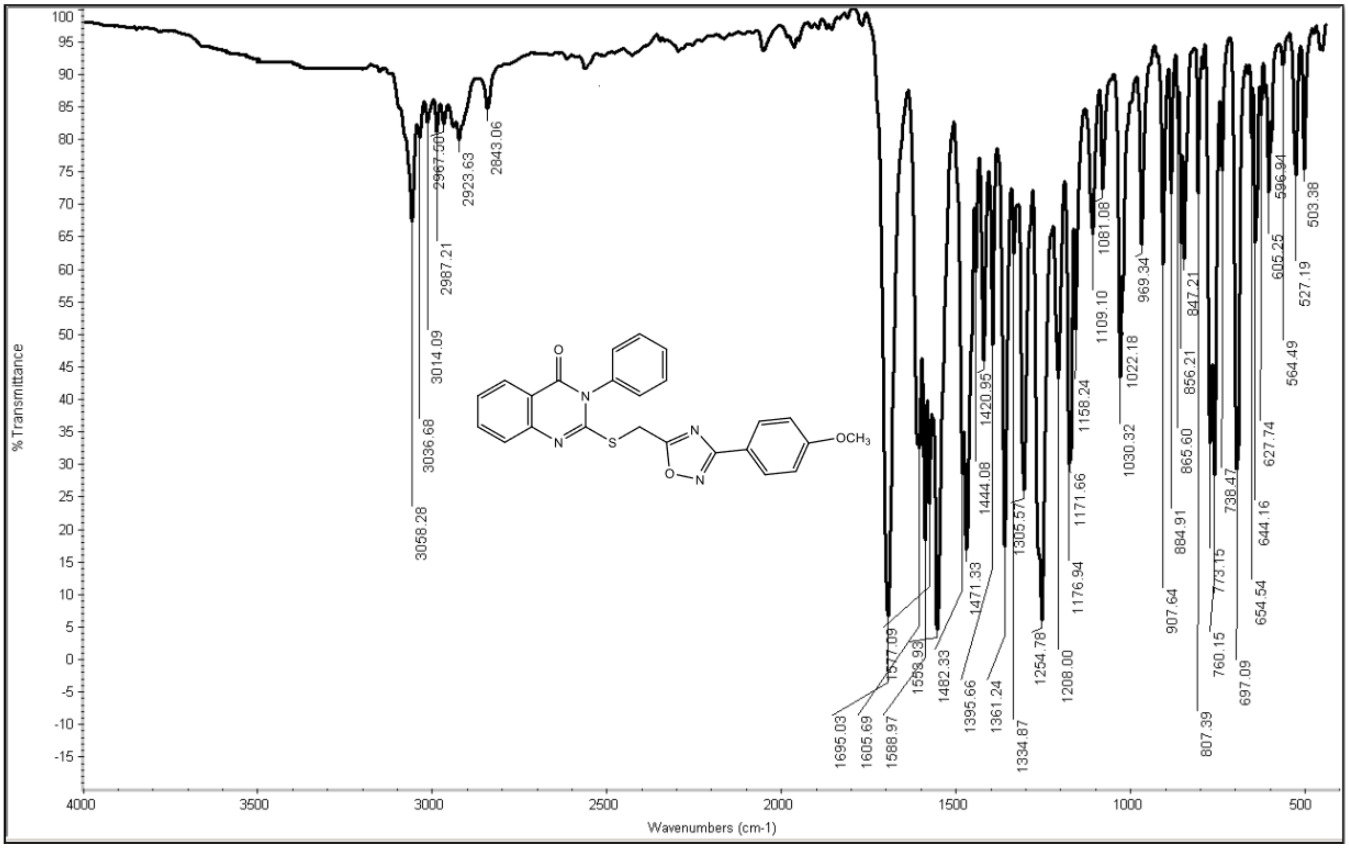

Figure S11: FTIR spectrum of compound **9c**

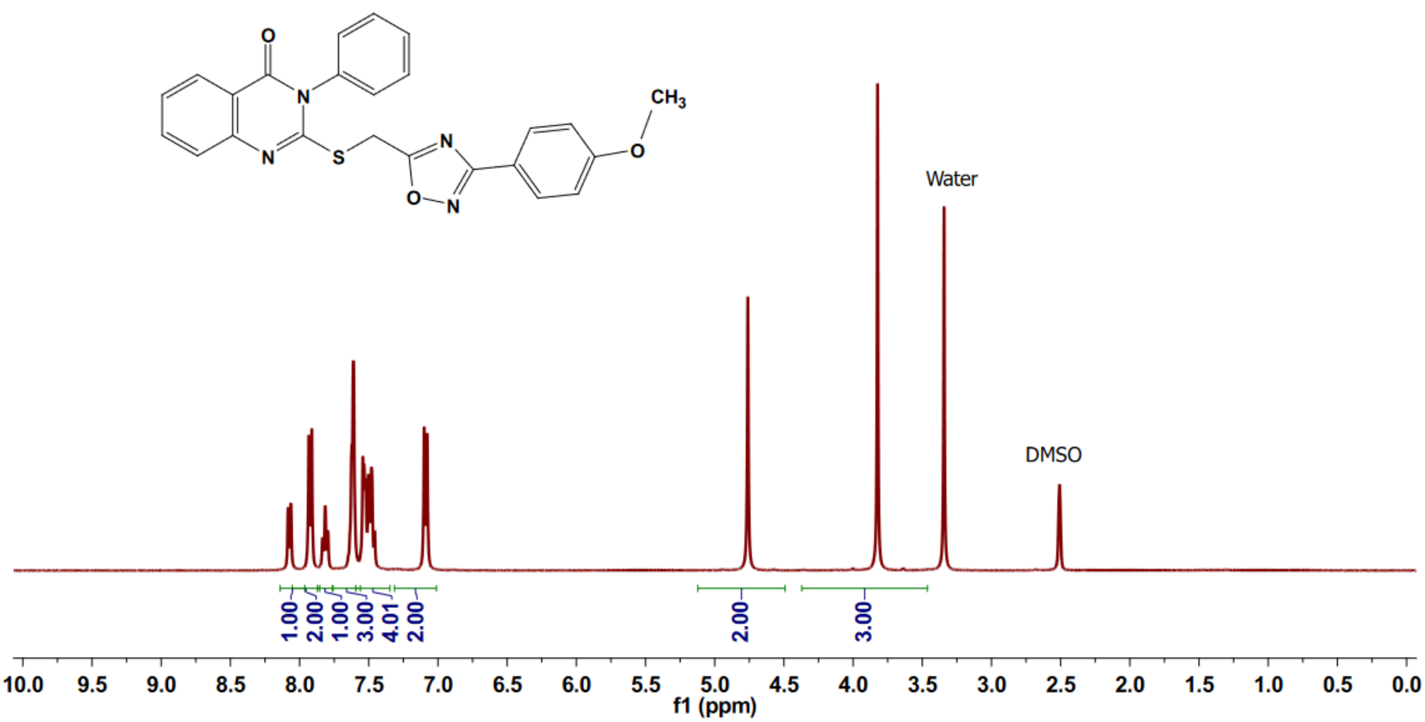

Figure S12: ^{1}H NMR spectrum (400 MHz, DMSO-d_6) of compound **9c**

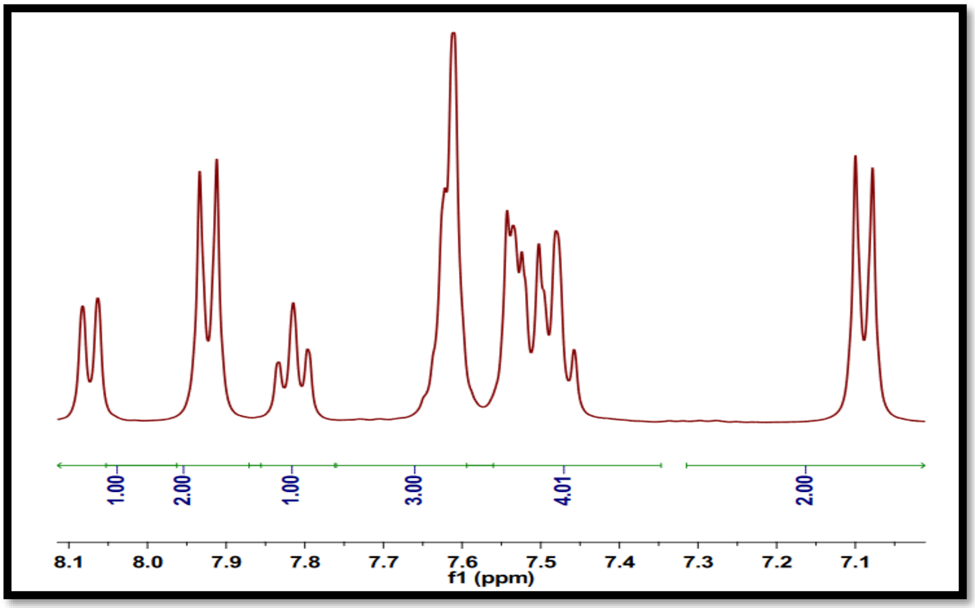

Figure S13: Expanded aromatic region of ^{1}HNMR spectrum (400 MHz, DMSO-d_6) of compound **9c**

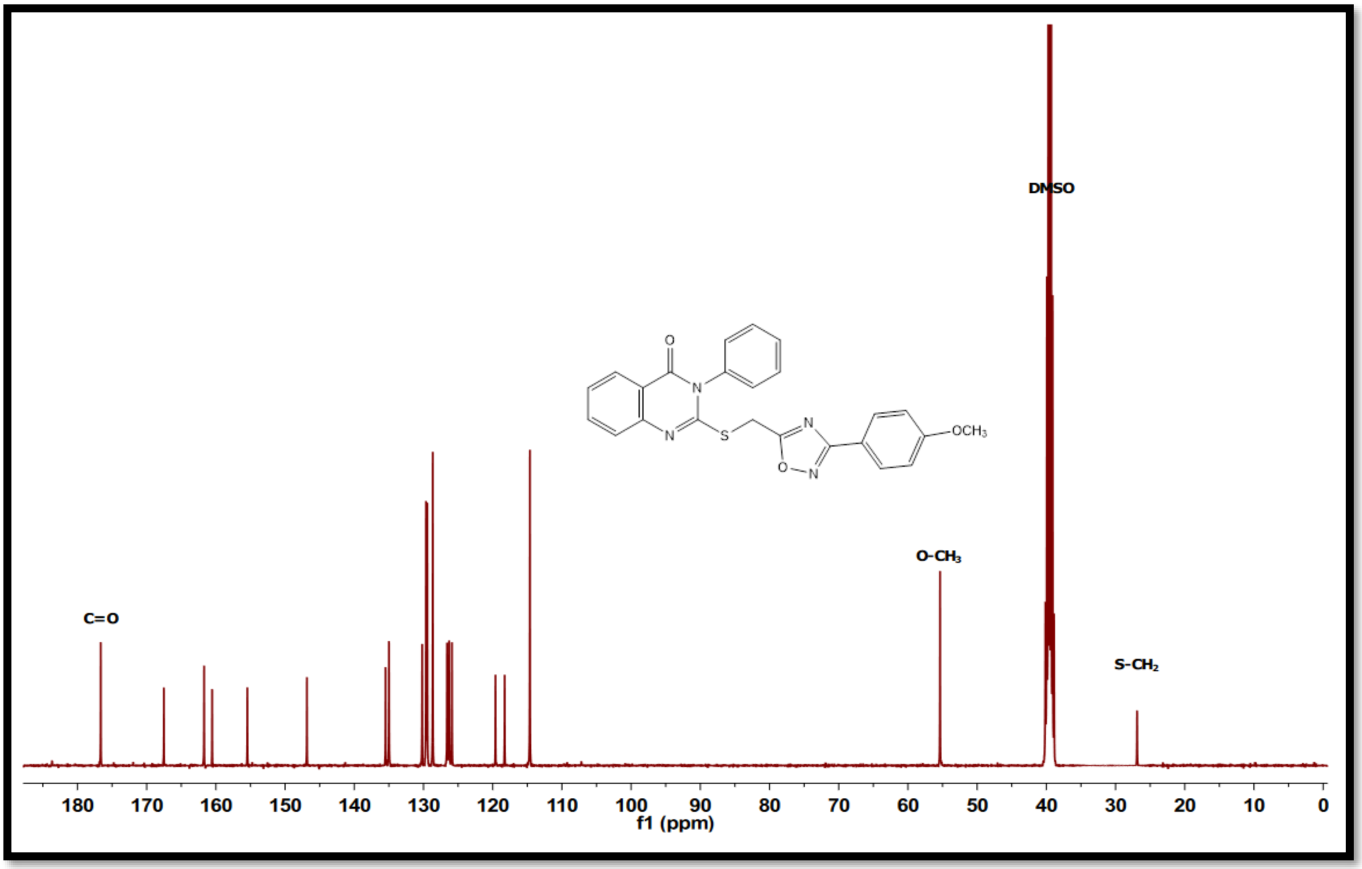

Figure S14: ^{13}C NMR spectrum (100 MHz, DMSO-d_6) of compound **9c**

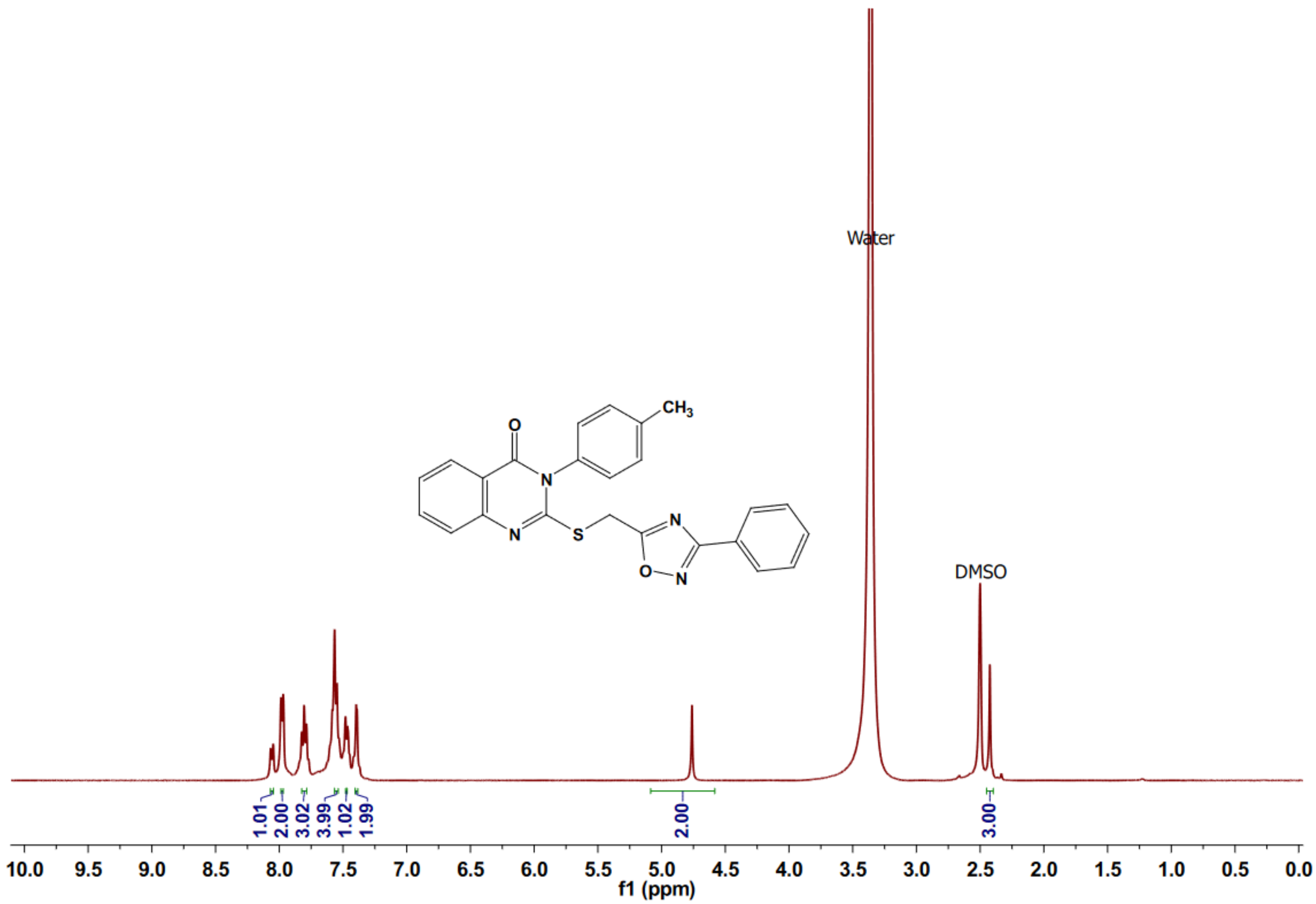

Figure S15: ^{1}H NMR spectrum (400 MHz, DMSO-d_6) of compound **9d**

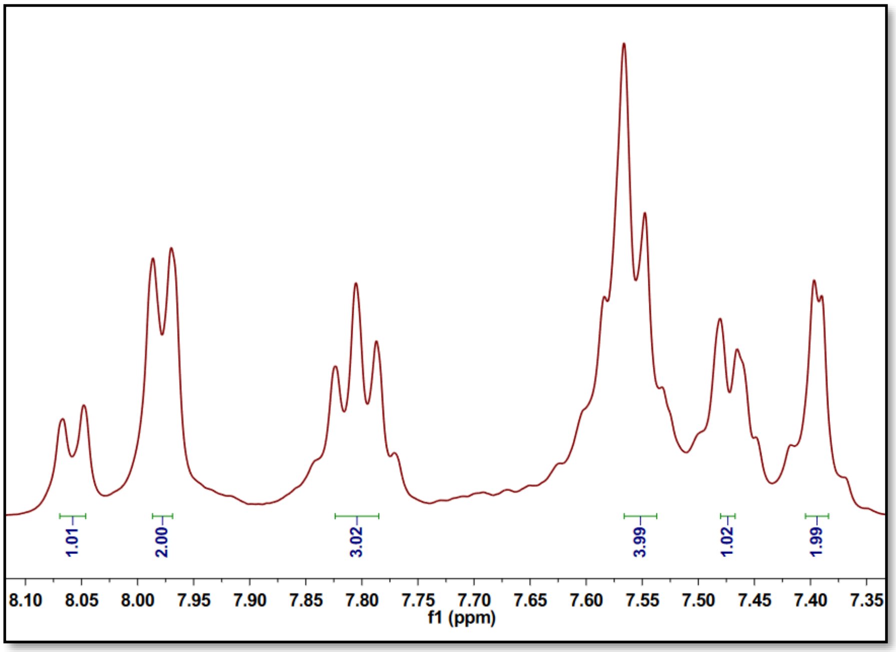

Figure S16: Expanded aromatic region of ^{1}H NMR spectrum (400 MHz, DMSO-d_6) of compound **9d**

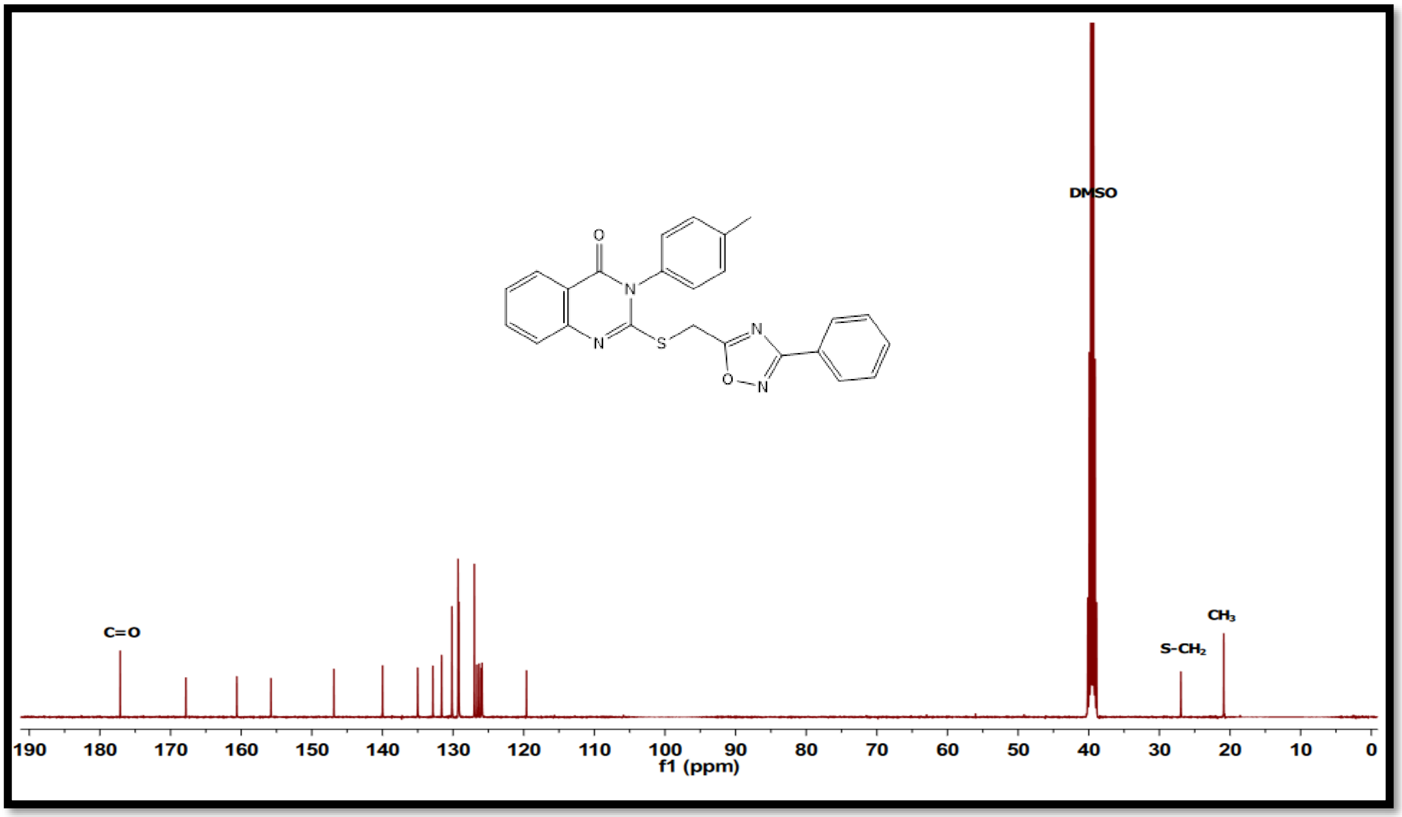

Figure S17: ^{13}C NMR spectrum (100 MHz, DMSO-d_6) of compound **9d**

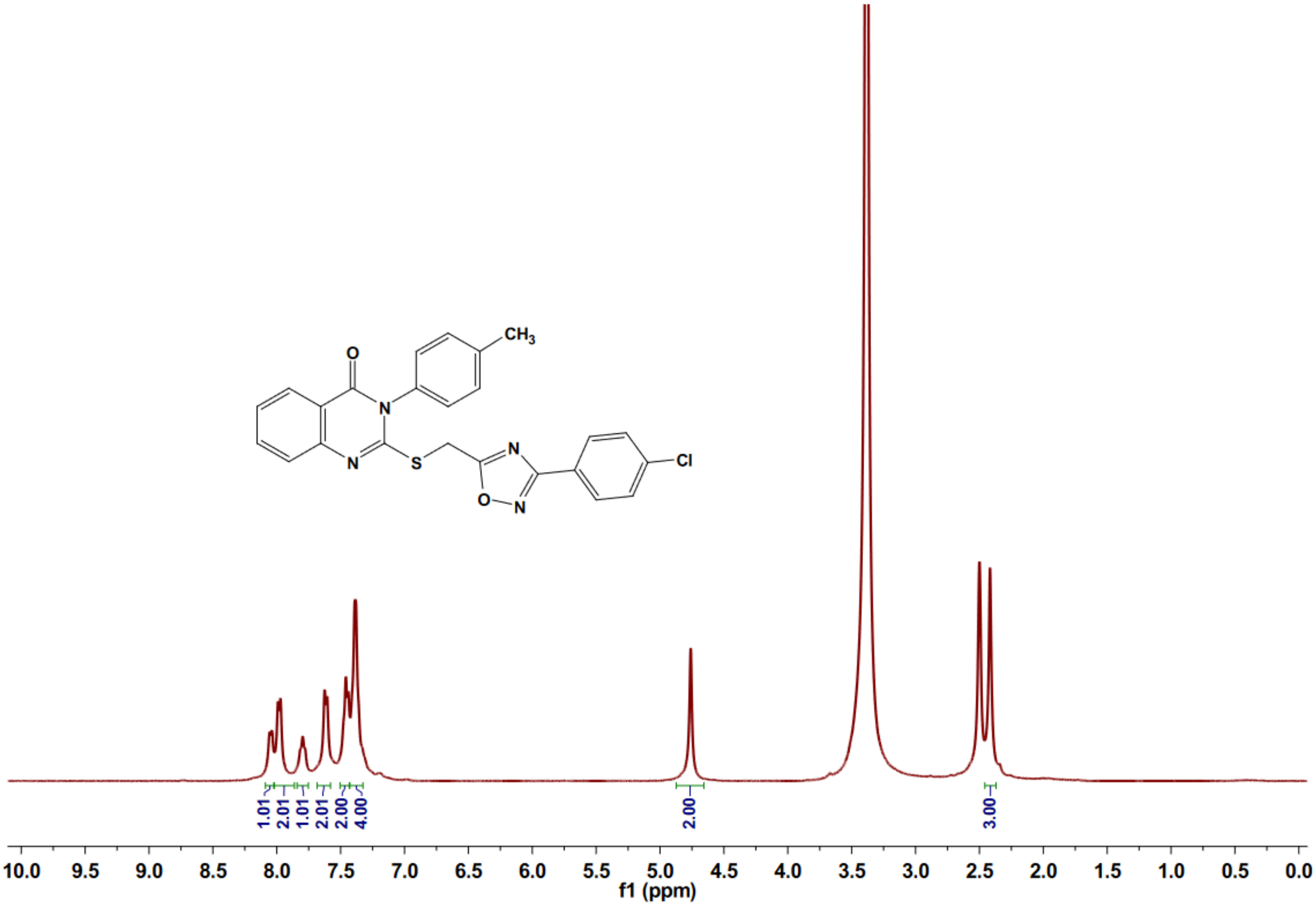

Figure s18: ^{1}H NMR spectrum (400 MHz, DMSO-d_6) of compound **9e.**

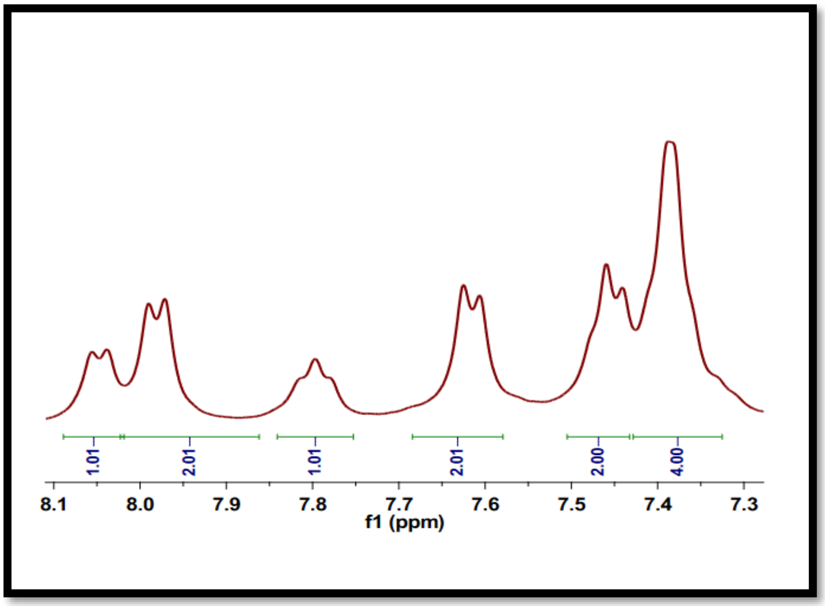

Figure S19: Expanded aromatic region of ^{1}HNMR spectrum (400 MHz, DMSO-d_6) of compound **9e**

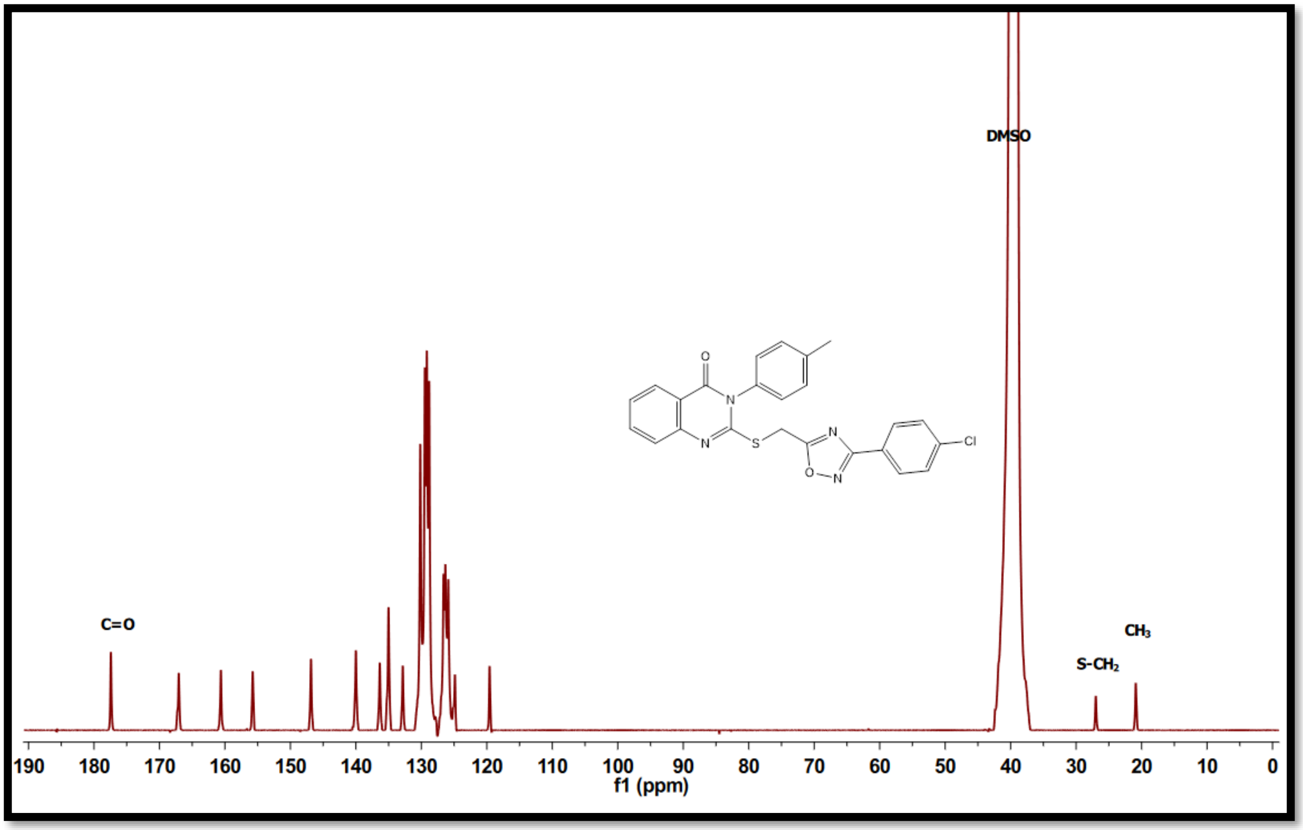

Figure S20: ^{13}C NMR spectrum (100 MHz, DMSO-d_6) of compound **9e**

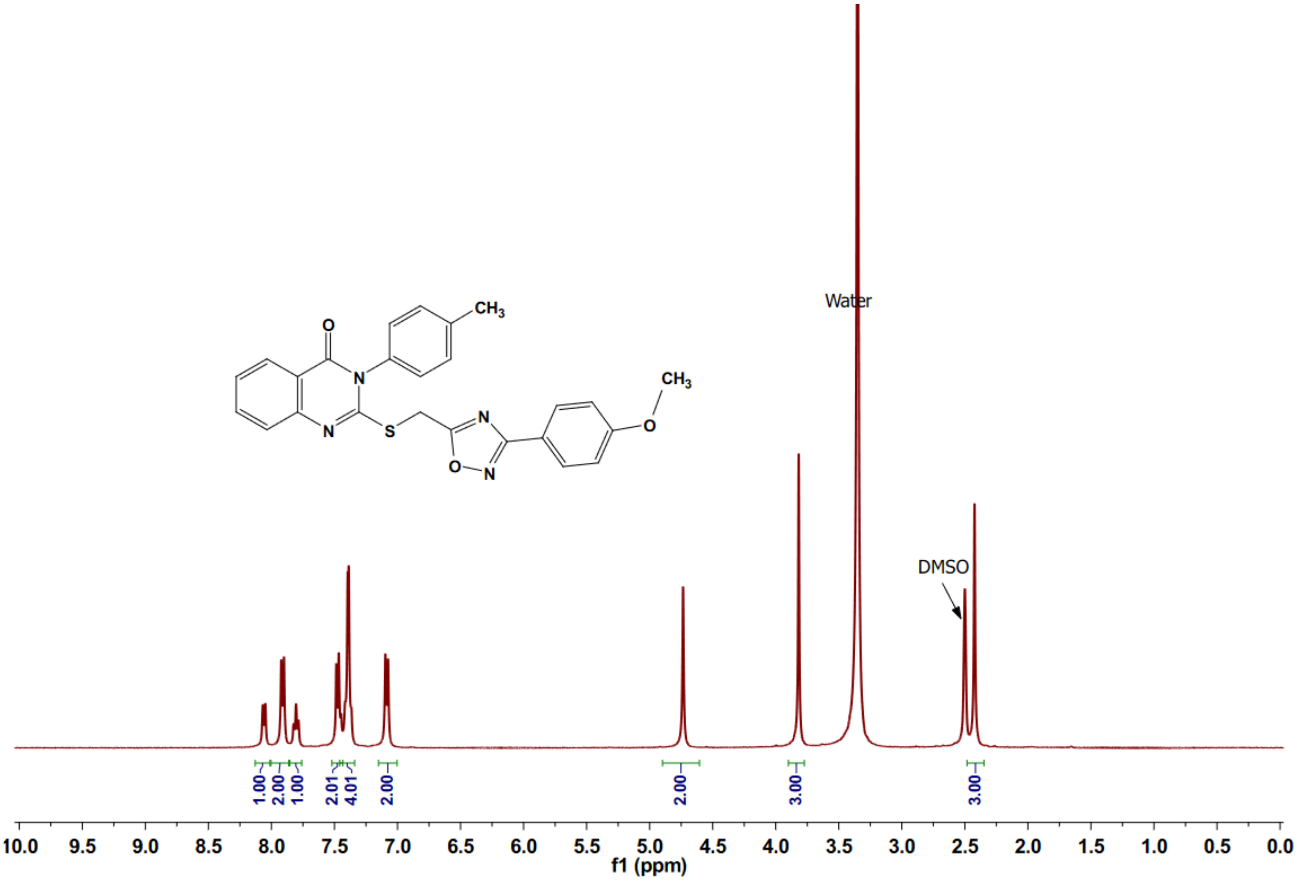

Figure S21: ^{1}H NMR spectrum (400 MHz, DMSO-d_6) of compound **9f**

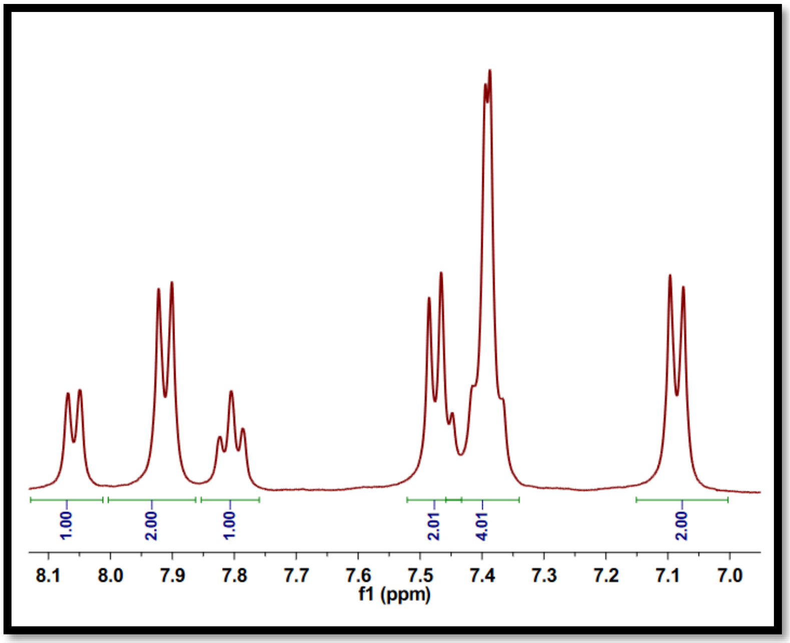

Figure S22: Expanded aromatic region of ^{1}HNMR spectrum (400 MHz, DMSO-d_6) of compound **9f**

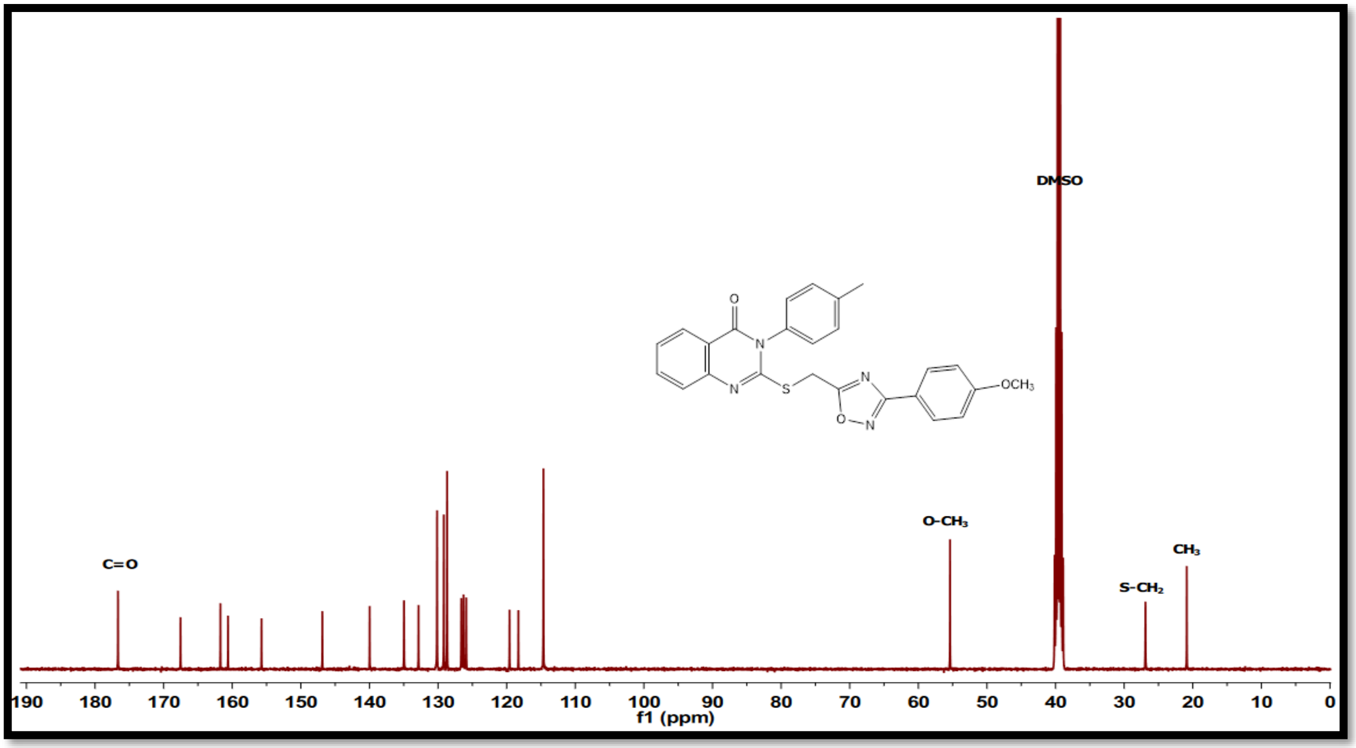

Figure S23: ^{13}C NMR spectrum (100 MHz, DMSO-d_6) of compound **9f**

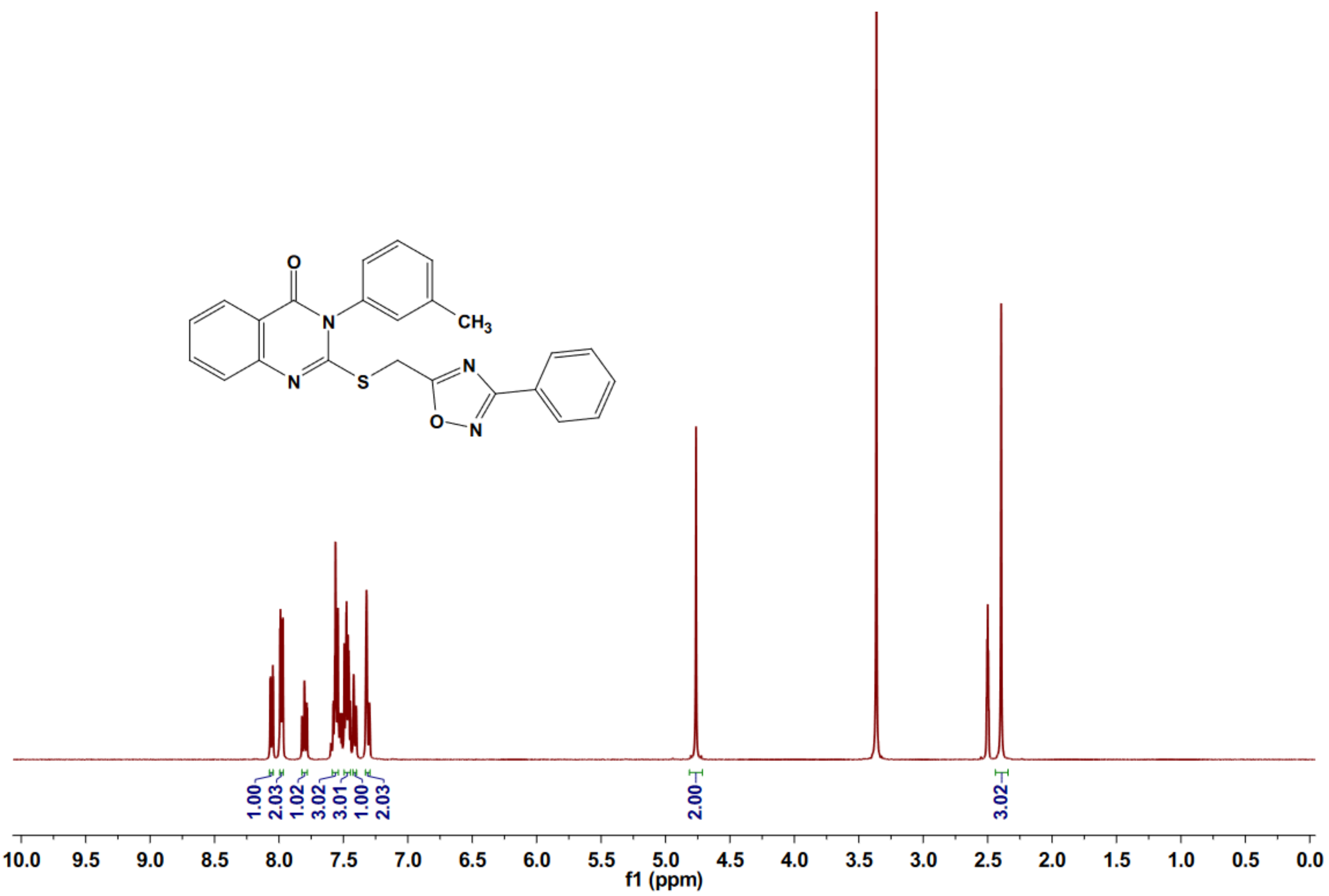

Figure S24: ^{1}H NMR spectrum (400 MHz, DMSO-d_6) of compound **9g**

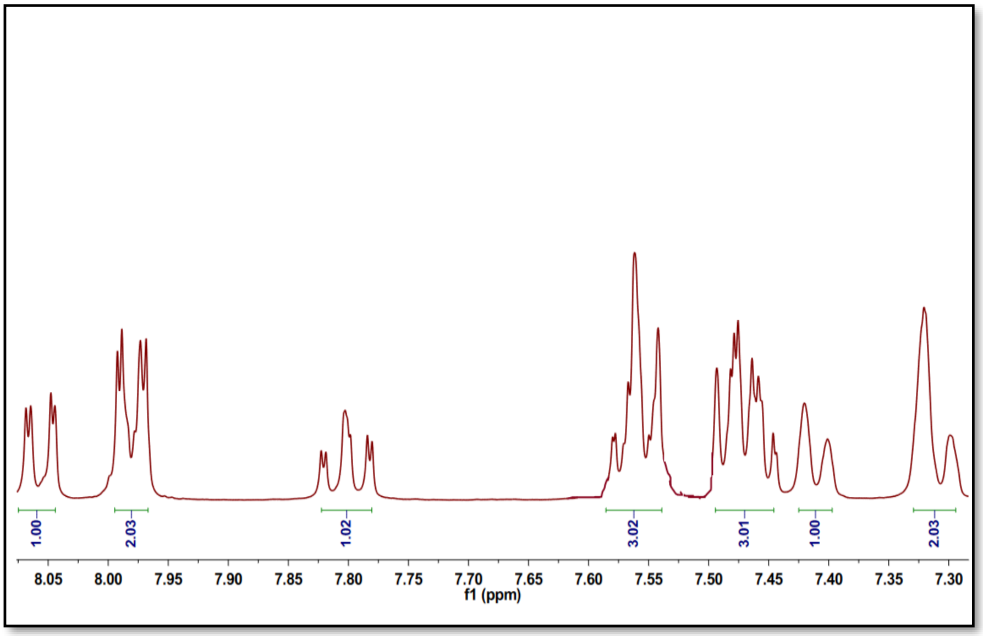

Figure S25: Expanded aromatic region of ^{1}HNMR spectrum (400 MHz, DMSO-d_6) of compound **9g**

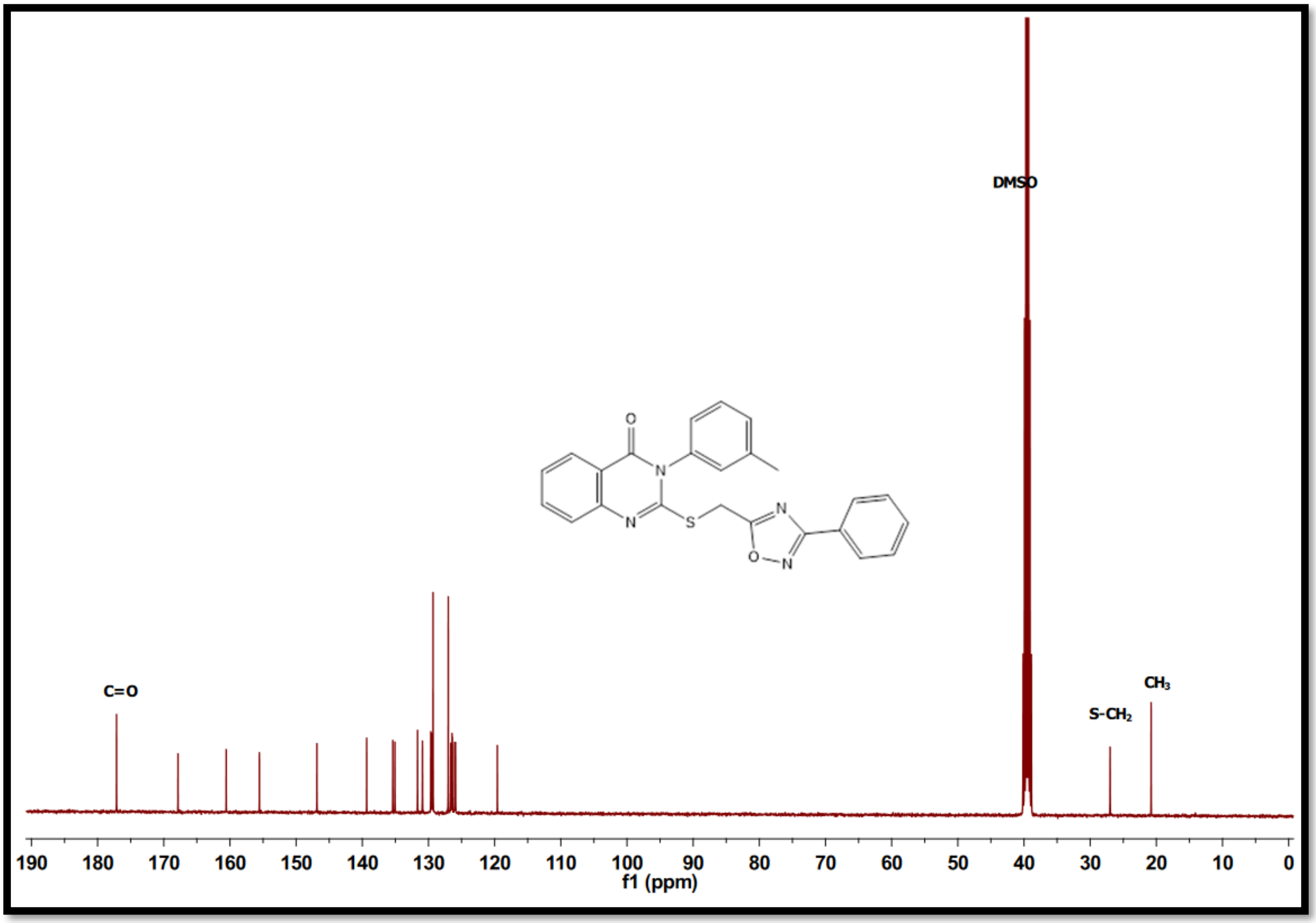

Figure S26: ^{13}C NMR spectrum (100 MHz, DMSO-d_6) of compound **9g**

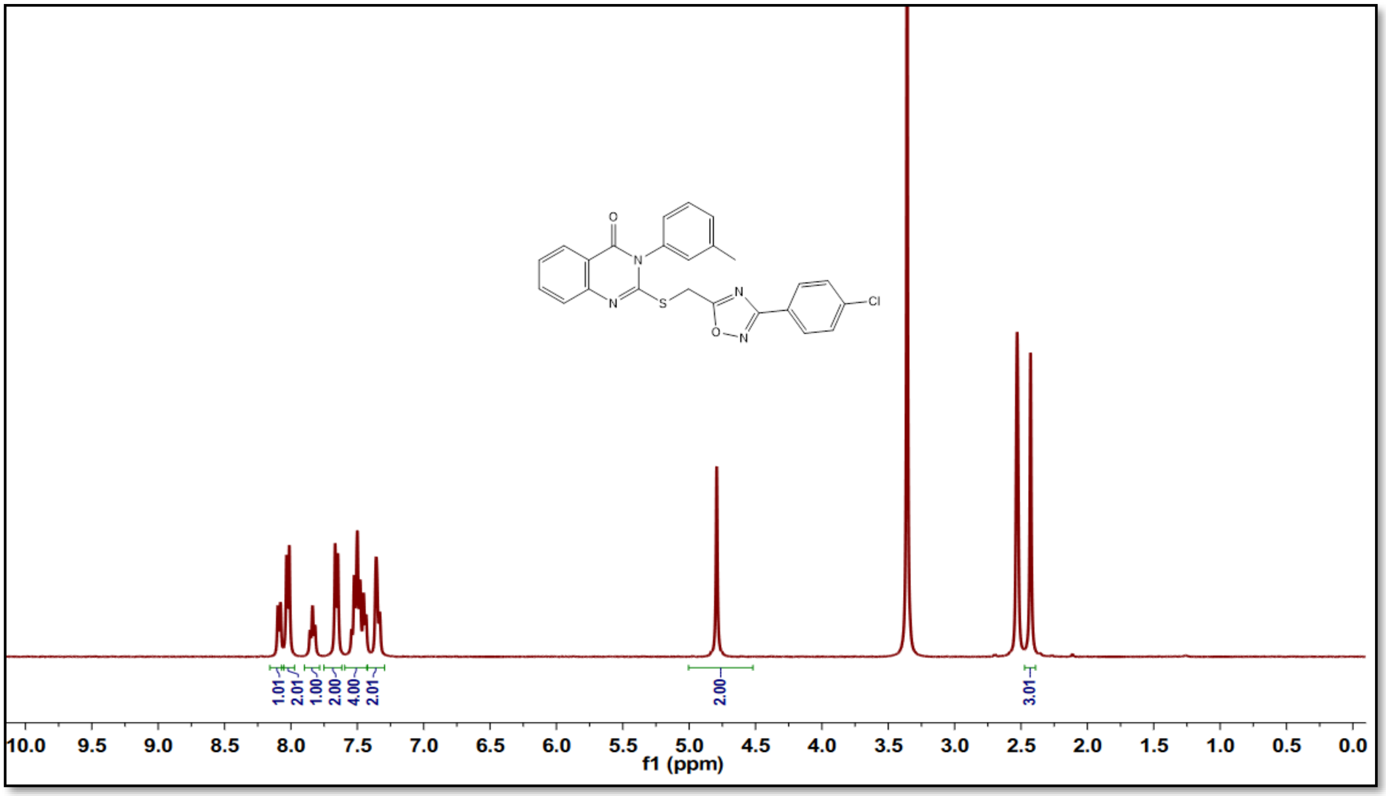

Figure S27: ^{1}H NMR spectrum (400 MHz, DMSO-d_6) of compound **9h**

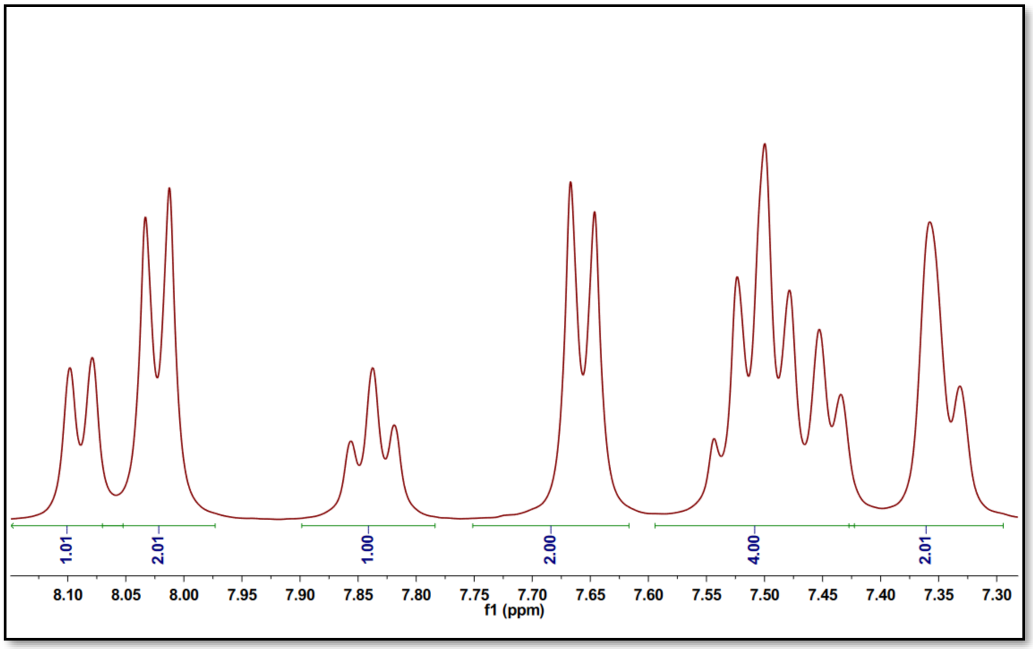

Figure S28: Expanded aromatic region of ^{1}HNMR spectrum (400 MHz, DMSO-d_6) of compound **9h**

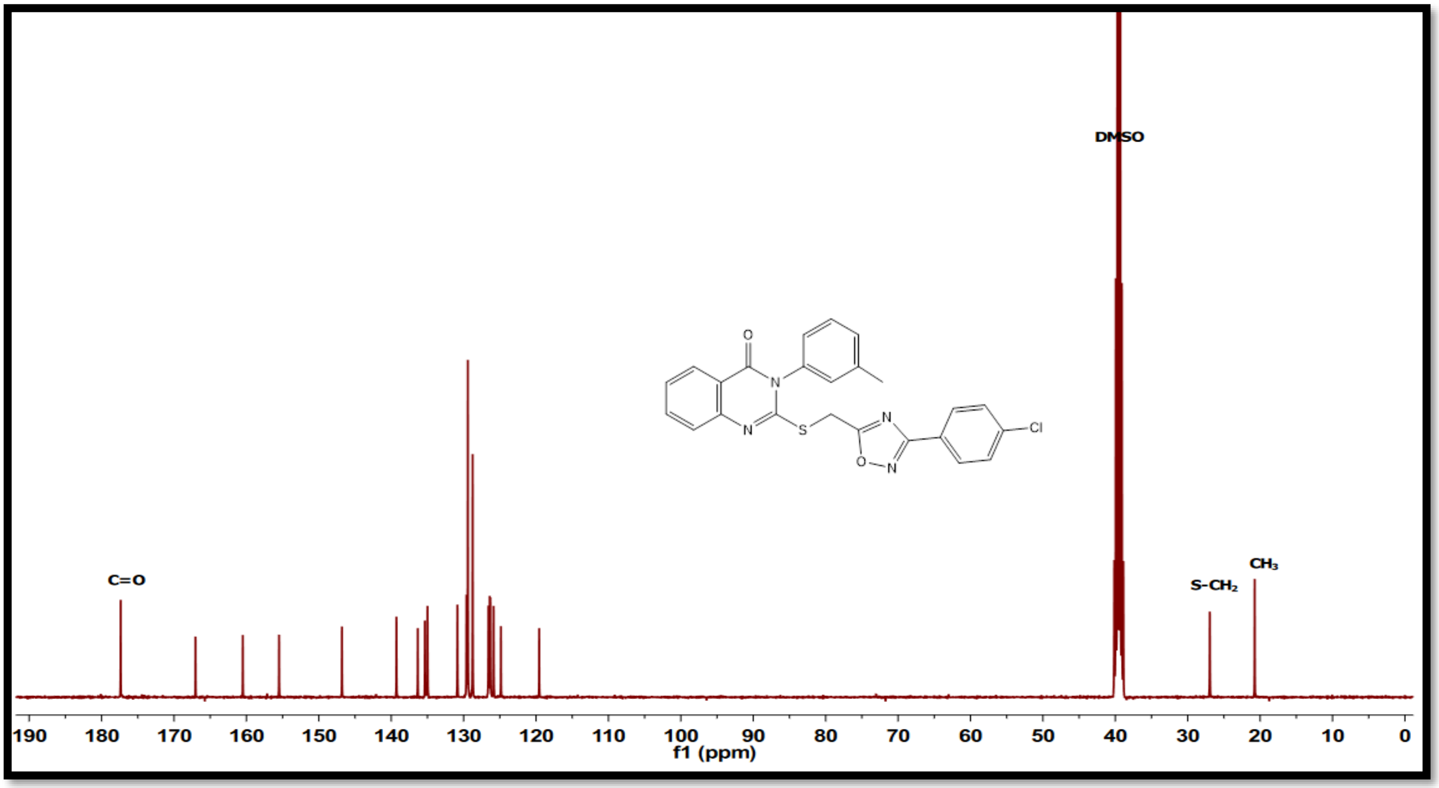

Figure S29: ^{13}C NMR spectrum (100 MHz, DMSO-d_6) of compound **9h**

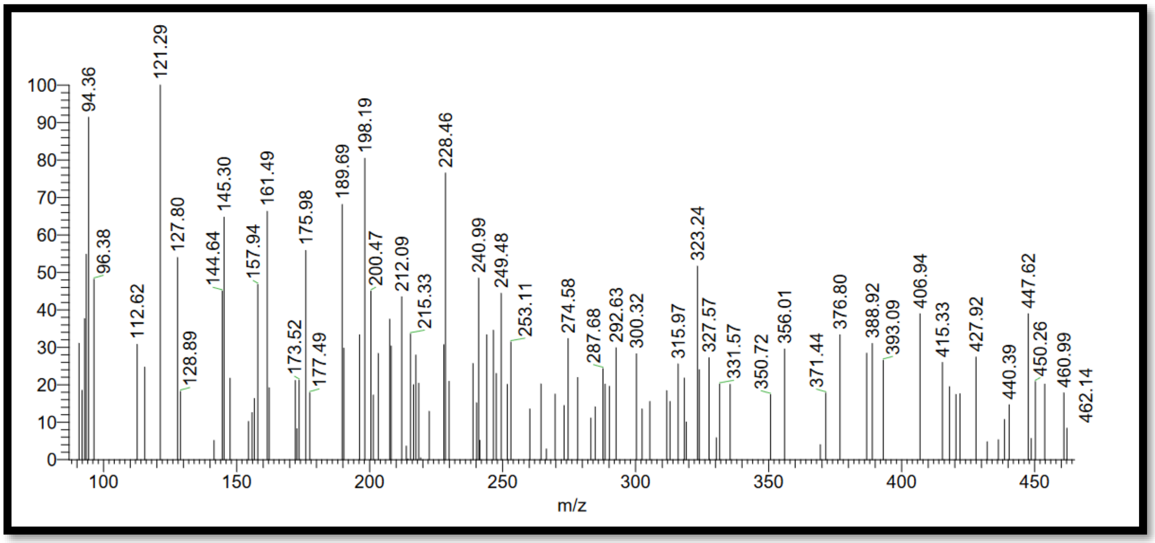

Figure S30: EI-Mass spectrum of compound **9h**

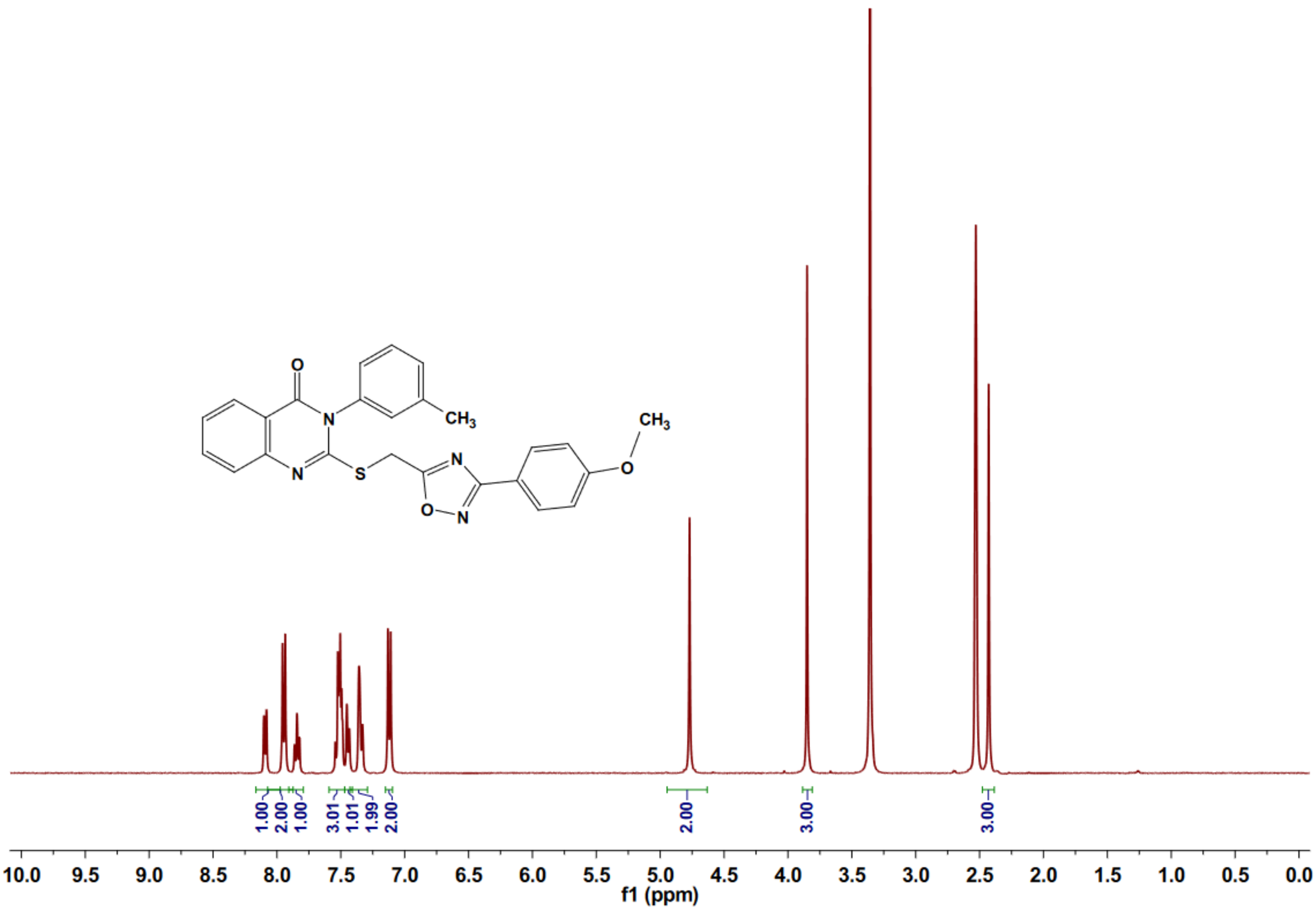

Figure S31: ^{1}H NMR spectrum (400 MHz, DMSO-d_6) of compound **9i**

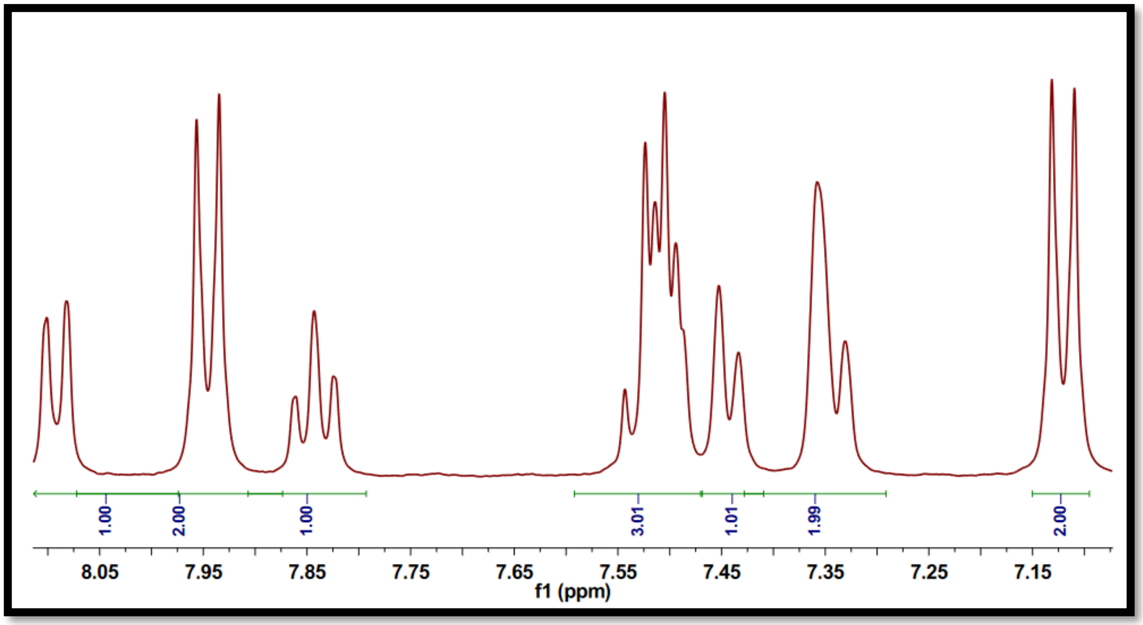

Figure S32: Expanded aromatic region of ^{1}HNMR spectrum (400 MHz, DMSO-d_6) of compound **9i**

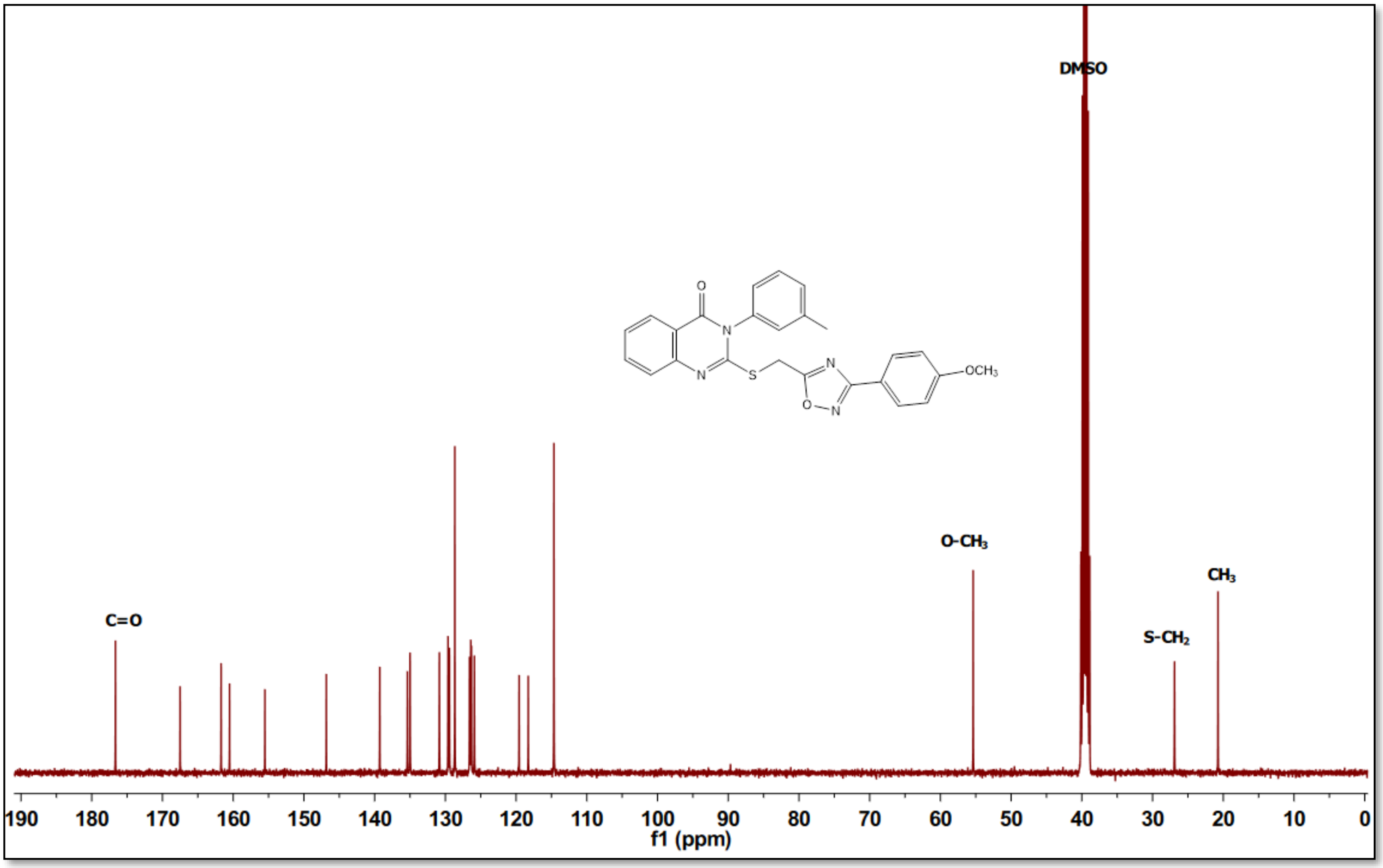

Figure S33: ^{13}C NMR spectrum (100 MHz, DMSO-d_6) of compound **9i**

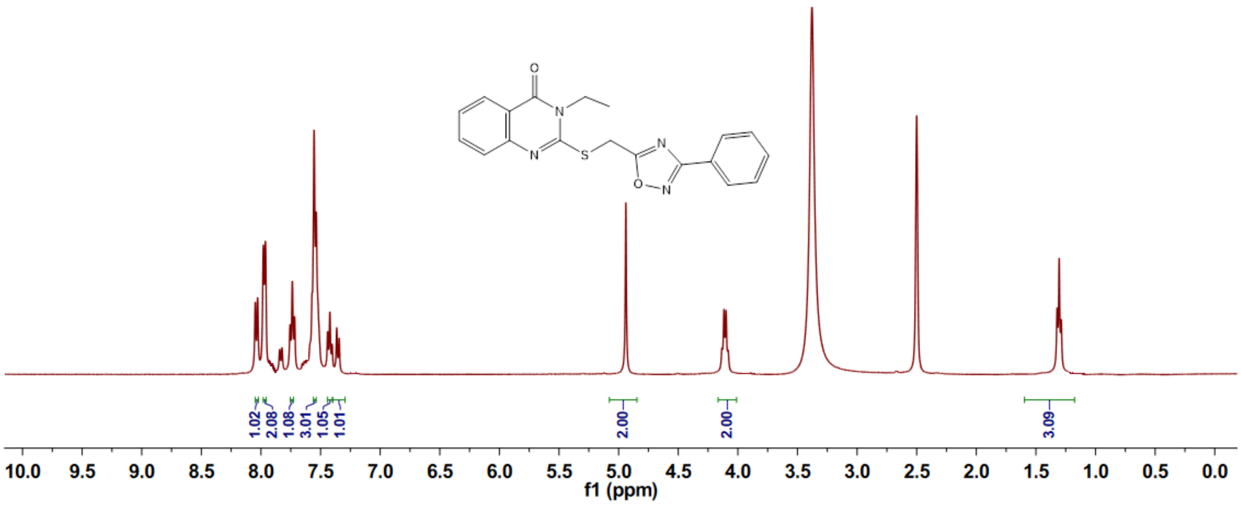

Figure S34: ^{1}H NMR spectrum (400 MHz, DMSO-d_6) of compound **9j**

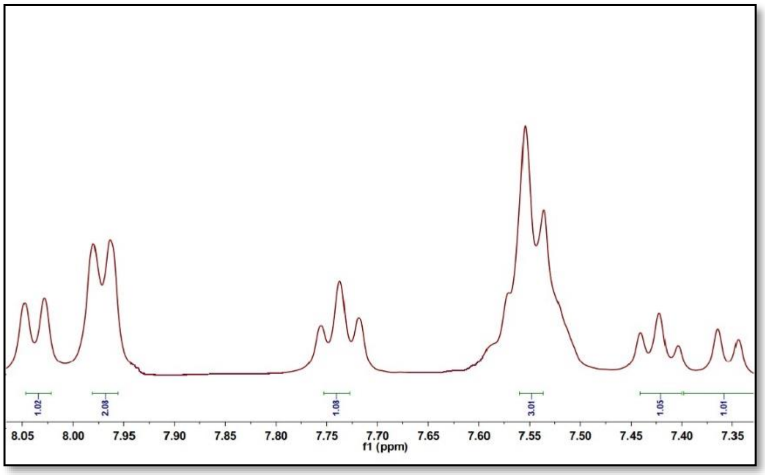

Figure S35: Expanded aromatic region of ^{1}HNMR spectrum (400 MHz, DMSO-d_6) of compound **9j**

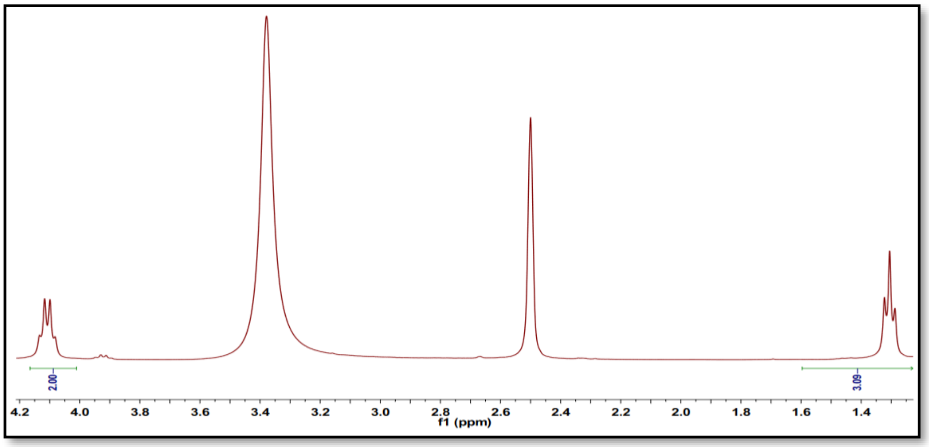

Figure S36: Expanded aliphatic region of ^{1}HNMR spectrum (400 MHz, DMSO-d_6) of compound **9j**

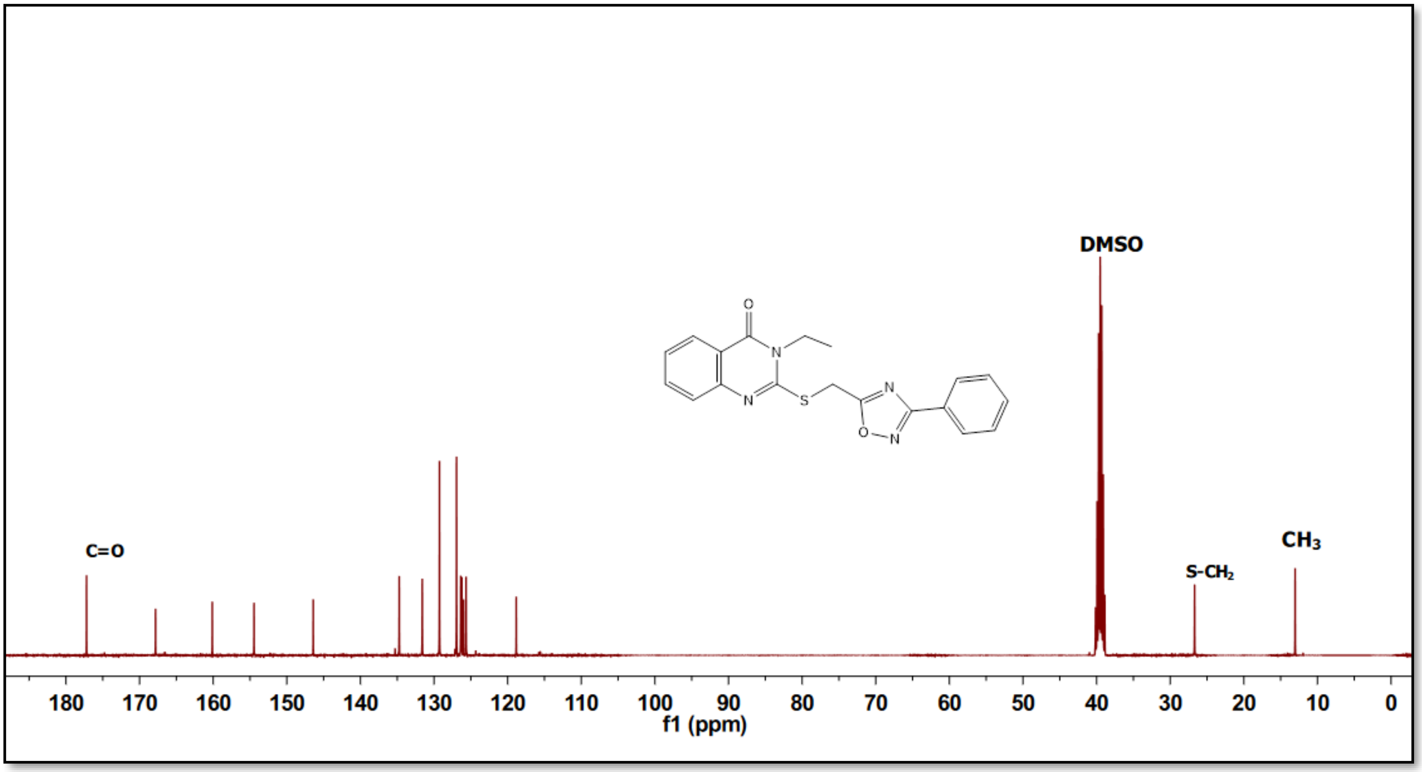

Figure S37: ^{13}C NMR spectrum (100 MHz, DMSO-d_6) of compound **9j**

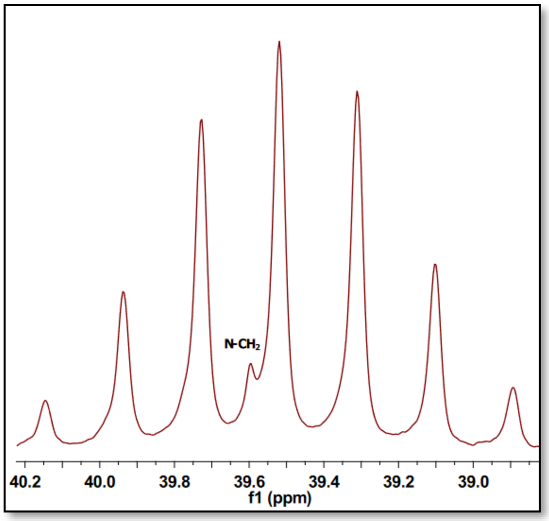

Figure S38: Expanded DMSO region of ^{13}C NMR spectrum (100 MHz, DMSO-d_6) of compound **9j**

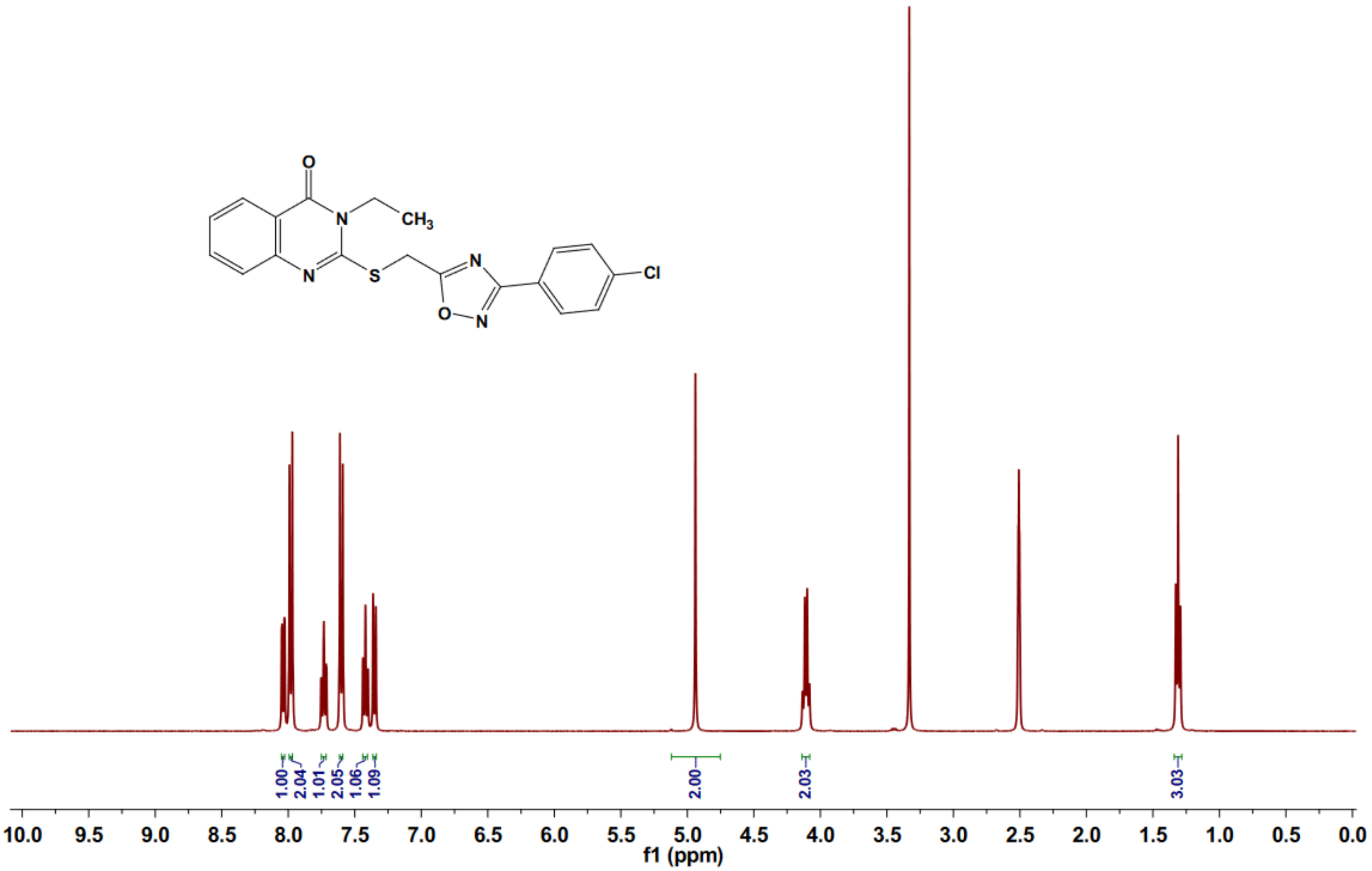

Figure S39: ^{1}H NMR spectrum (400 MHz, DMSO-d_6) of compound **9k**

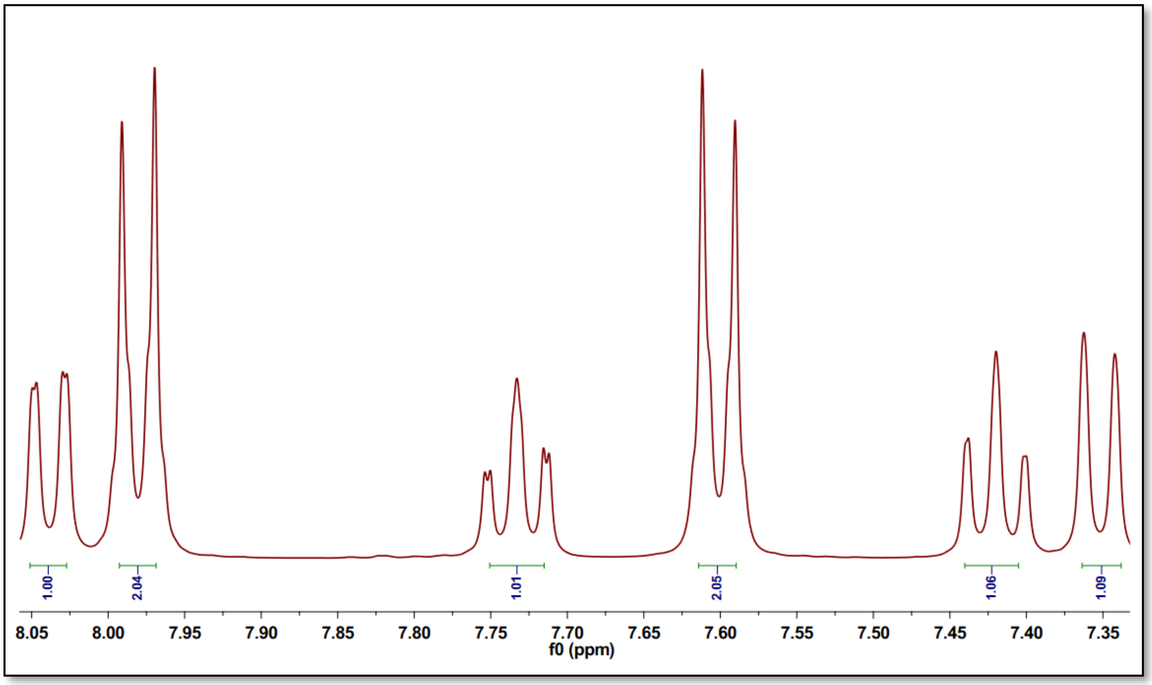

Figure S40: Expanded aromatic region of ^{1}HNMR spectrum (400 MHz, DMSO-d_6) of compound **9k**

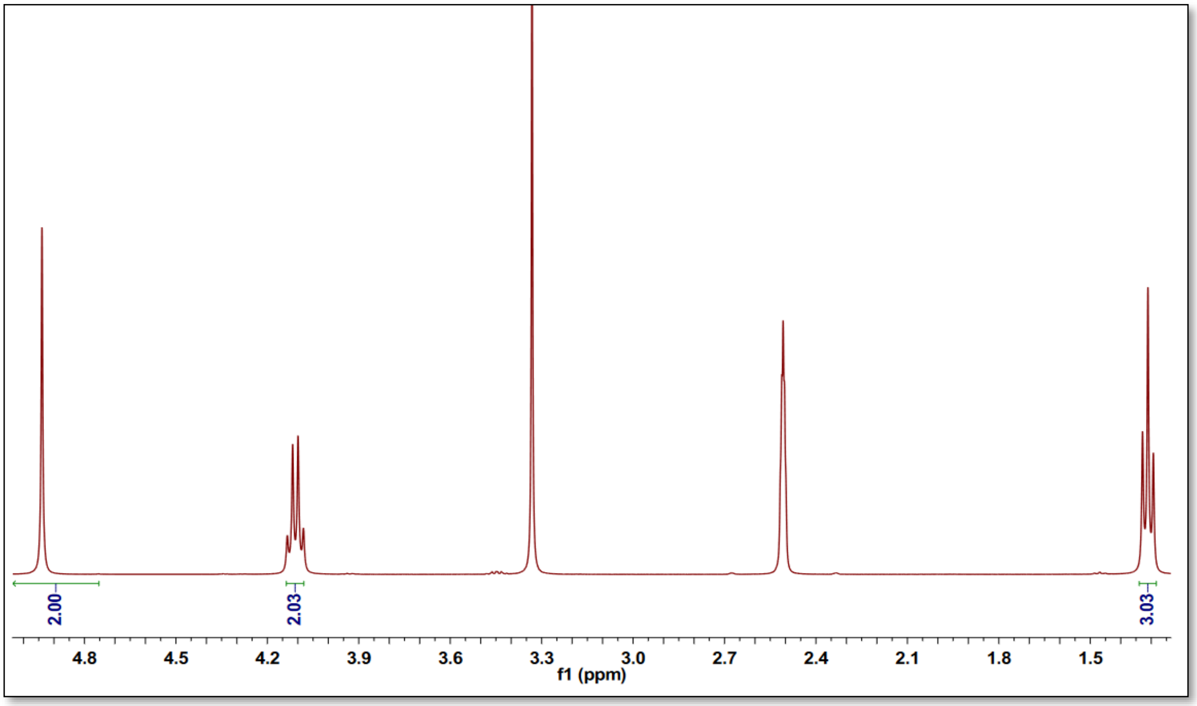

Figure S41: Expanded aliphatic region of ^{1}HNMR spectrum (400 MHz, DMSO-*d*6) of compound **9k**

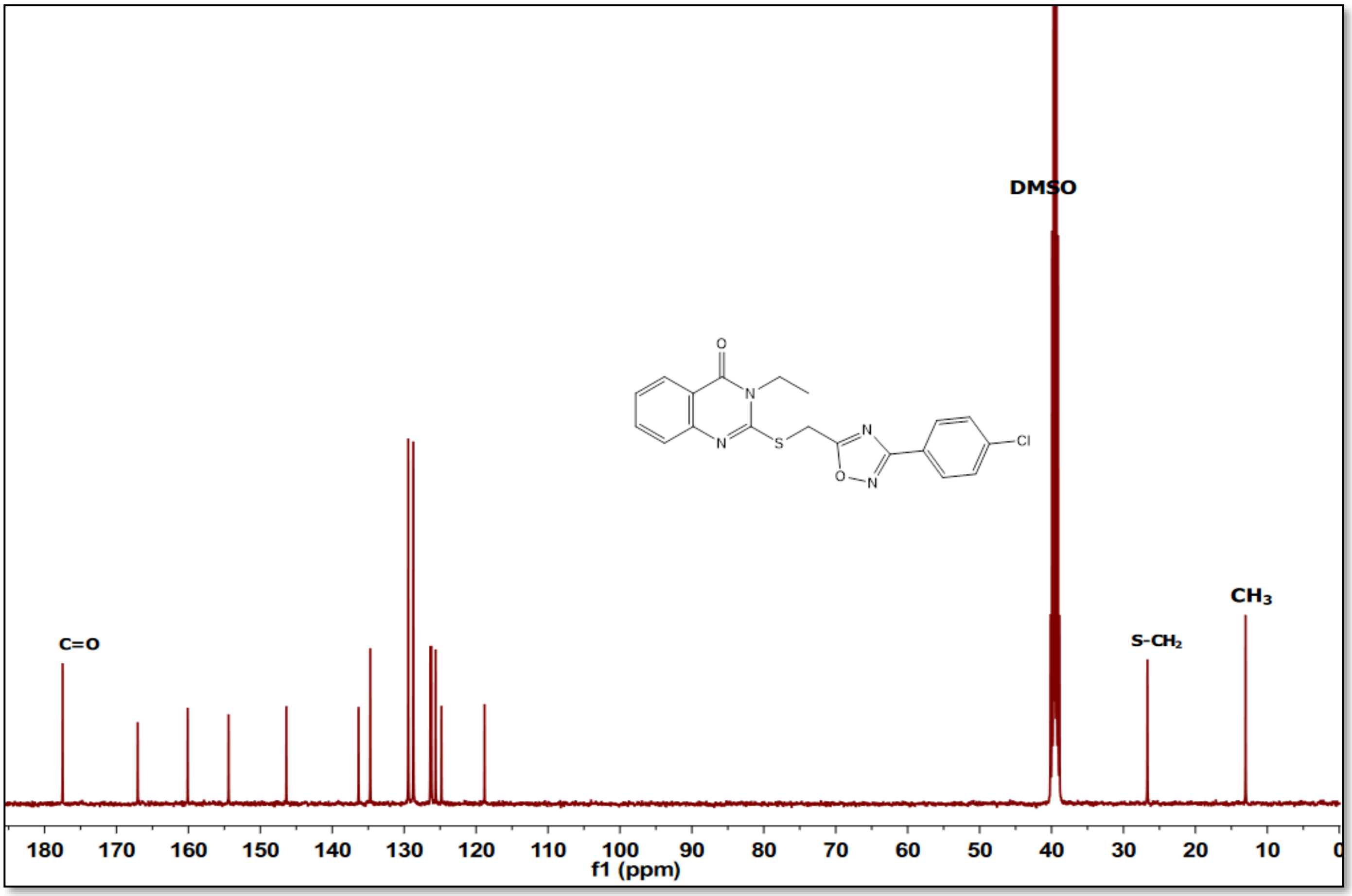

Figure S42: ^{13}C NMR spectrum (100 MHz, DMSO-*d*6) of compound **9k**

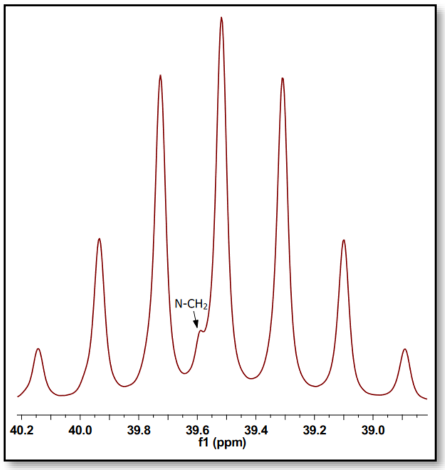

Figure S43: Expanded DMSO region of ^{13}C NMR spectrum (100 MHz, DMSO-d_6) of compound **9k**

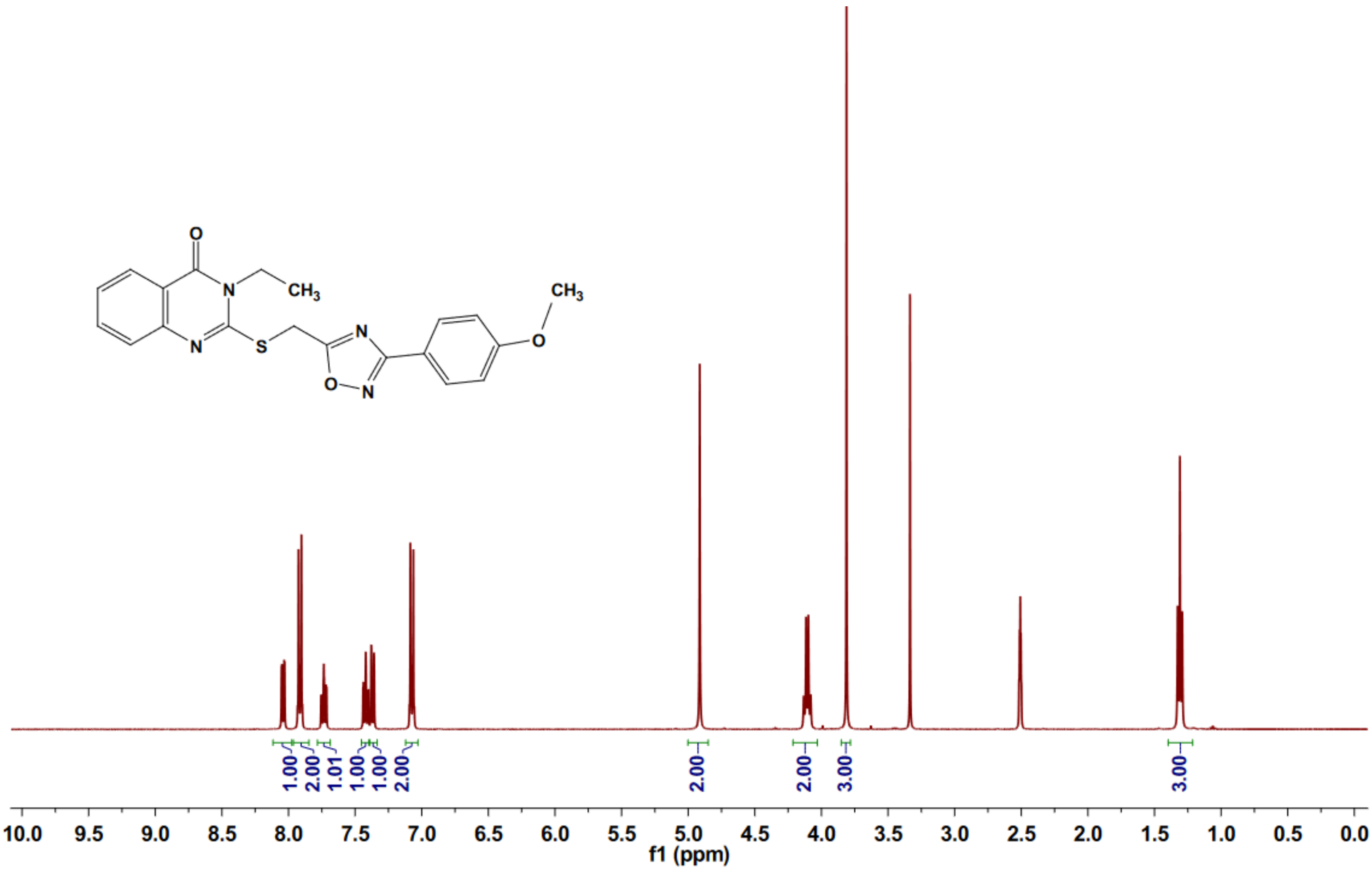

Figure S44: ^{1}H NMR spectrum (400 MHz, DMSO-d_6) of compound **9l**

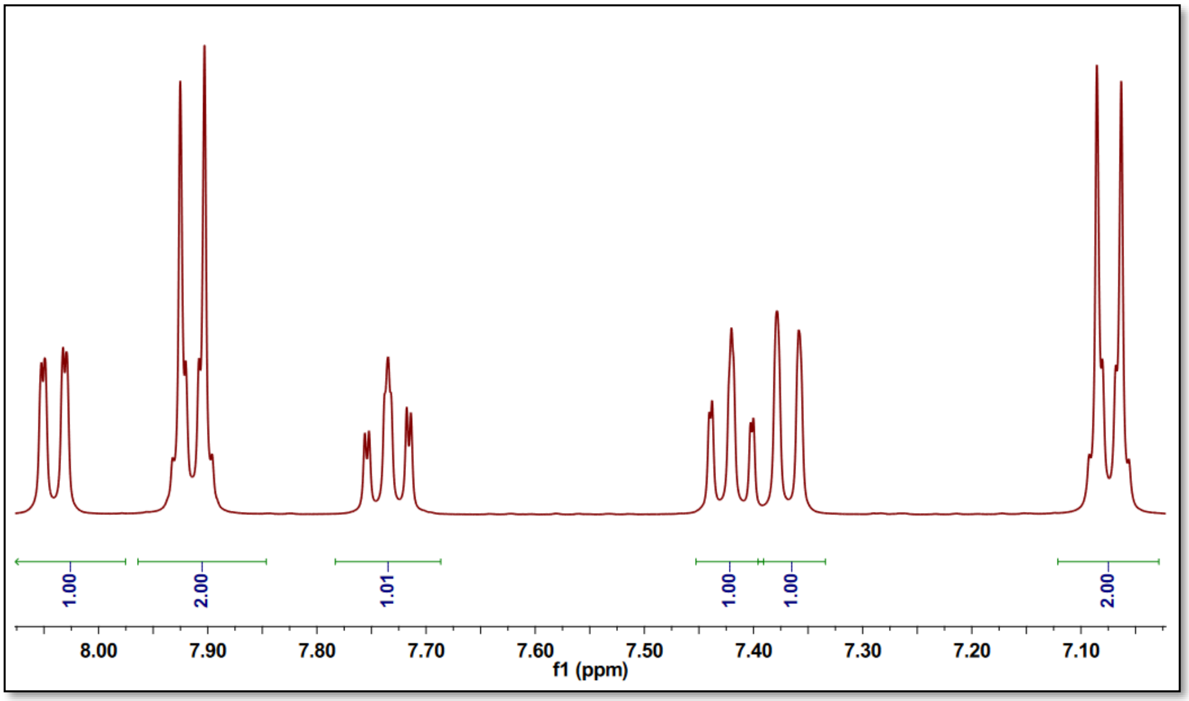

Figure S45: Expanded aromatic region of ^{1}HNMR spectrum (400 MHz, DMSO-d_6) of compound **9l**

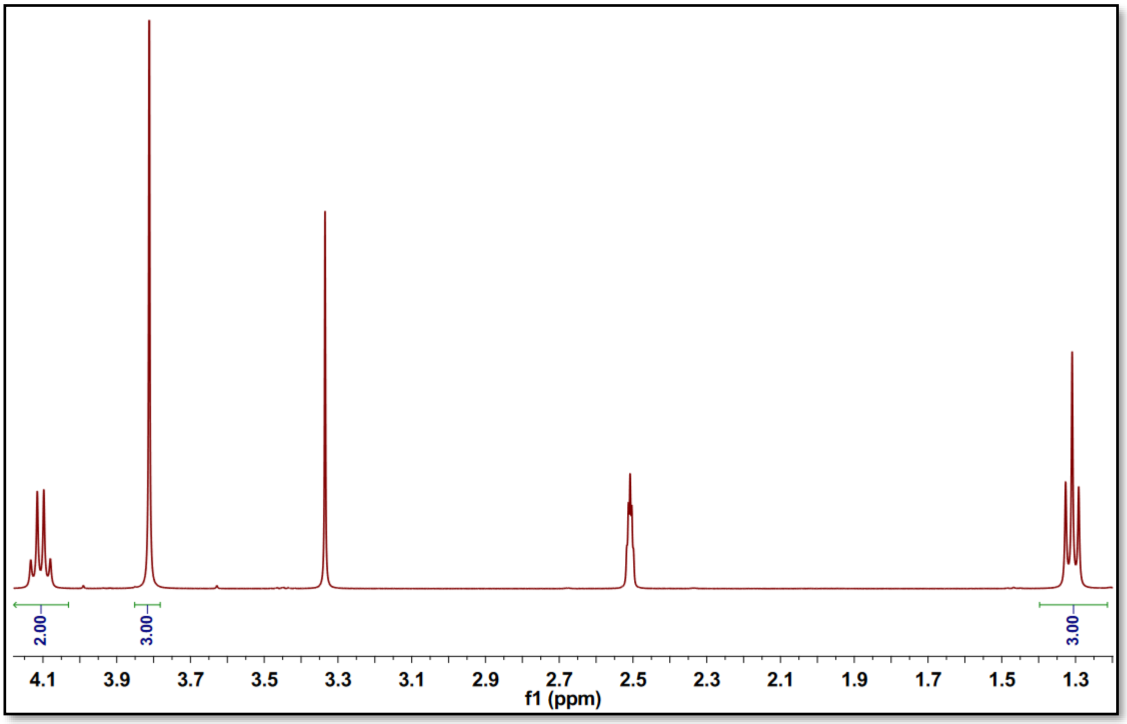

Figure S46: Expanded aliphatic region of ^{1}HNMR spectrum (400 MHz, DMSO-d_6) of compound **9l**

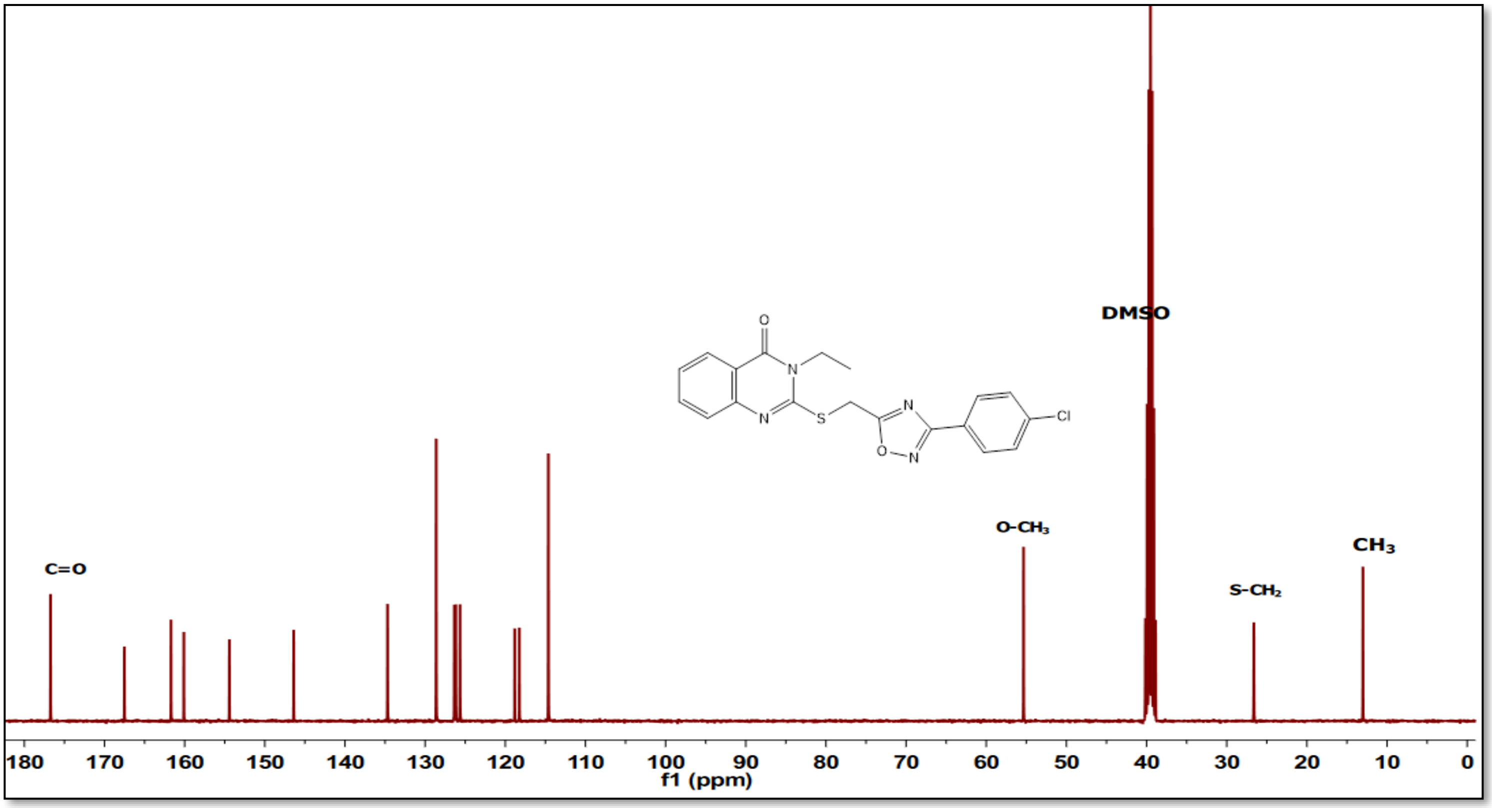

Figure S47: ^{13}C NMR spectrum (100 MHz, DMSO-d_6) of compound **9l**

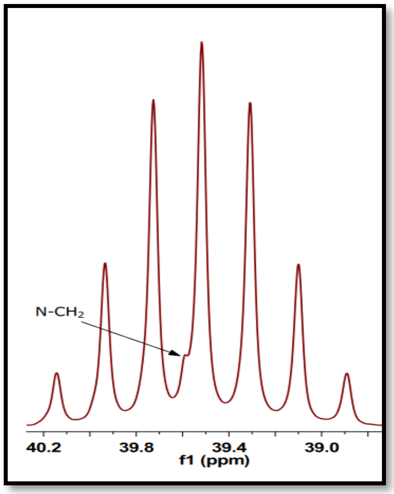

Figure S48: Expanded DMSO region of ^{13}C NMR spectrum (100 MHz, DMSO-d_6) of compound **9l**

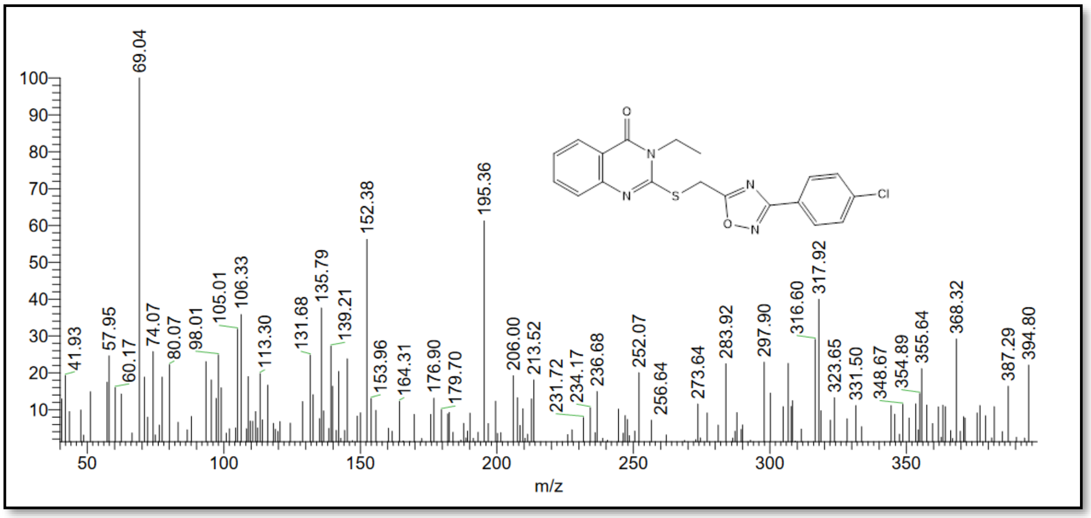

Figure S49: EI-Mass spectrum of compound **9l**

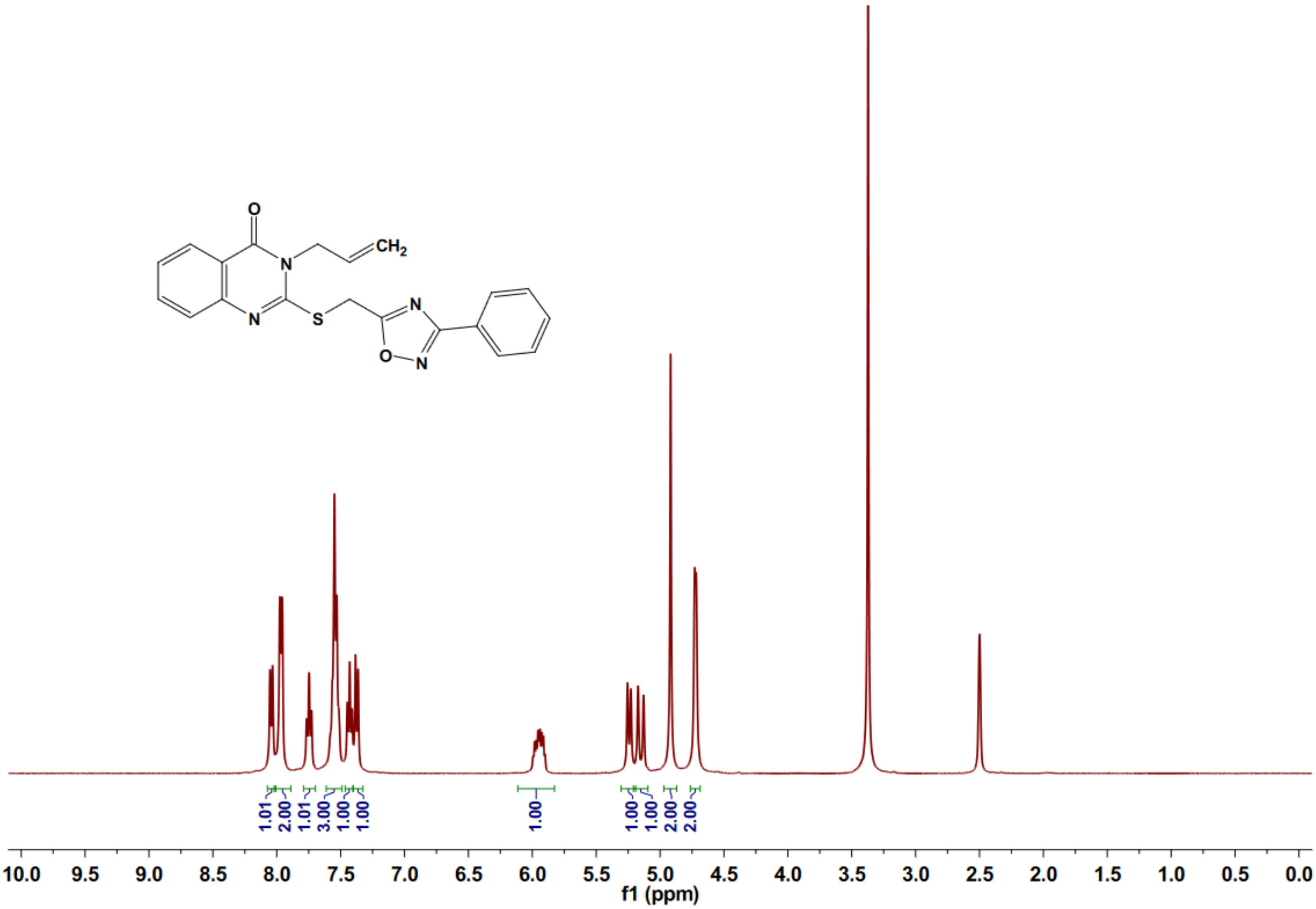

Figure S50: ^{1}H NMR spectrum (400 MHz, DMSO-d_6) of compound **9m**

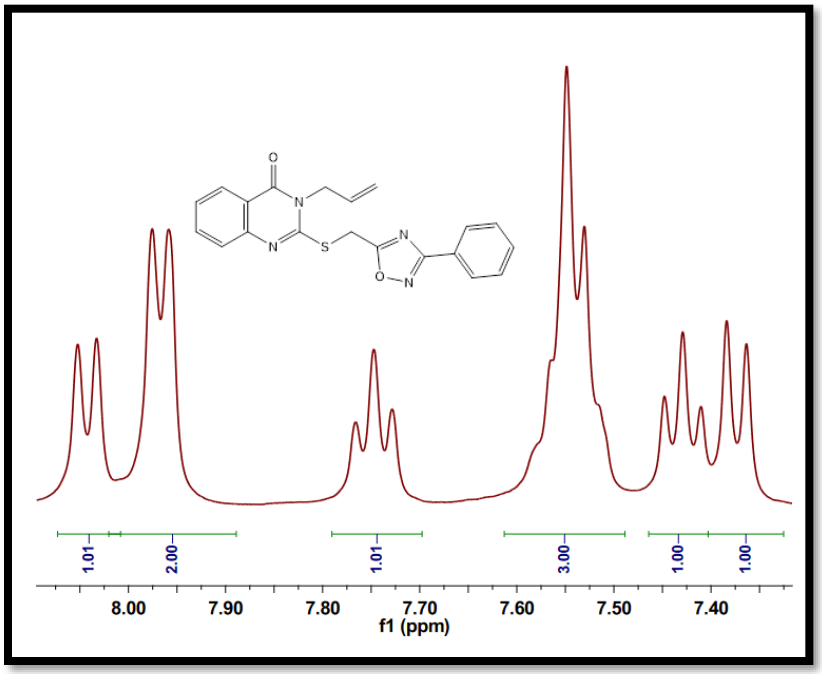

Figure S51: Expanded aromatic region of ^{1}HNMR spectrum (400 MHz, DMSO-d_6) of compound **9m**

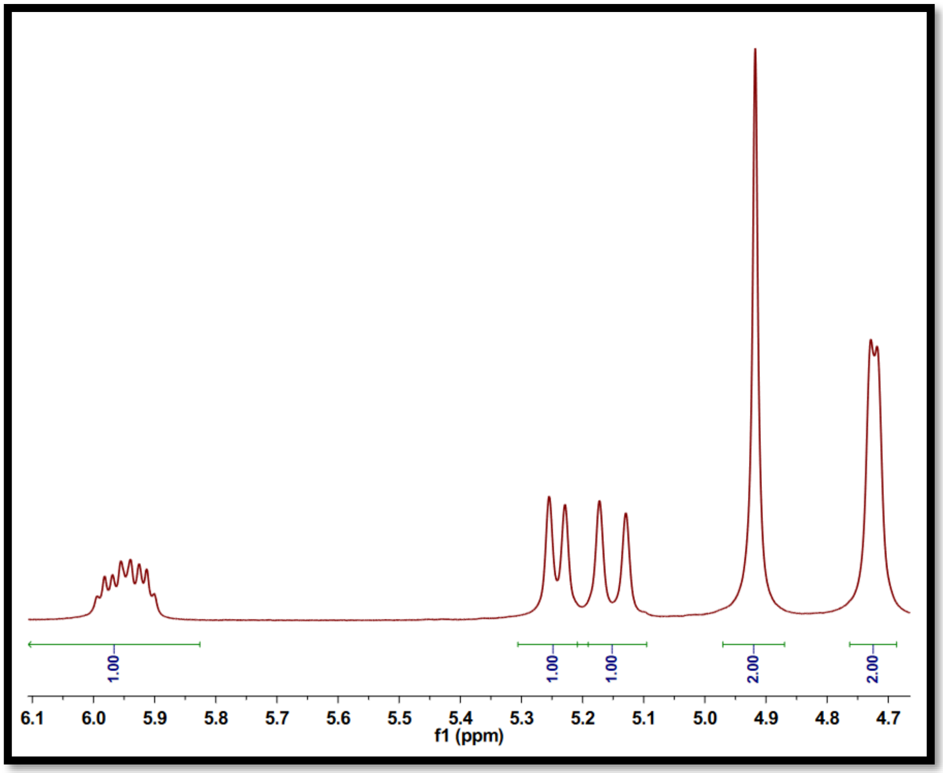

Figure S52: Expanded aliphatic and olefinic regions of ^{1}HNMR spectrum (400 MHz, DMSO-d_6) of compound **9m**

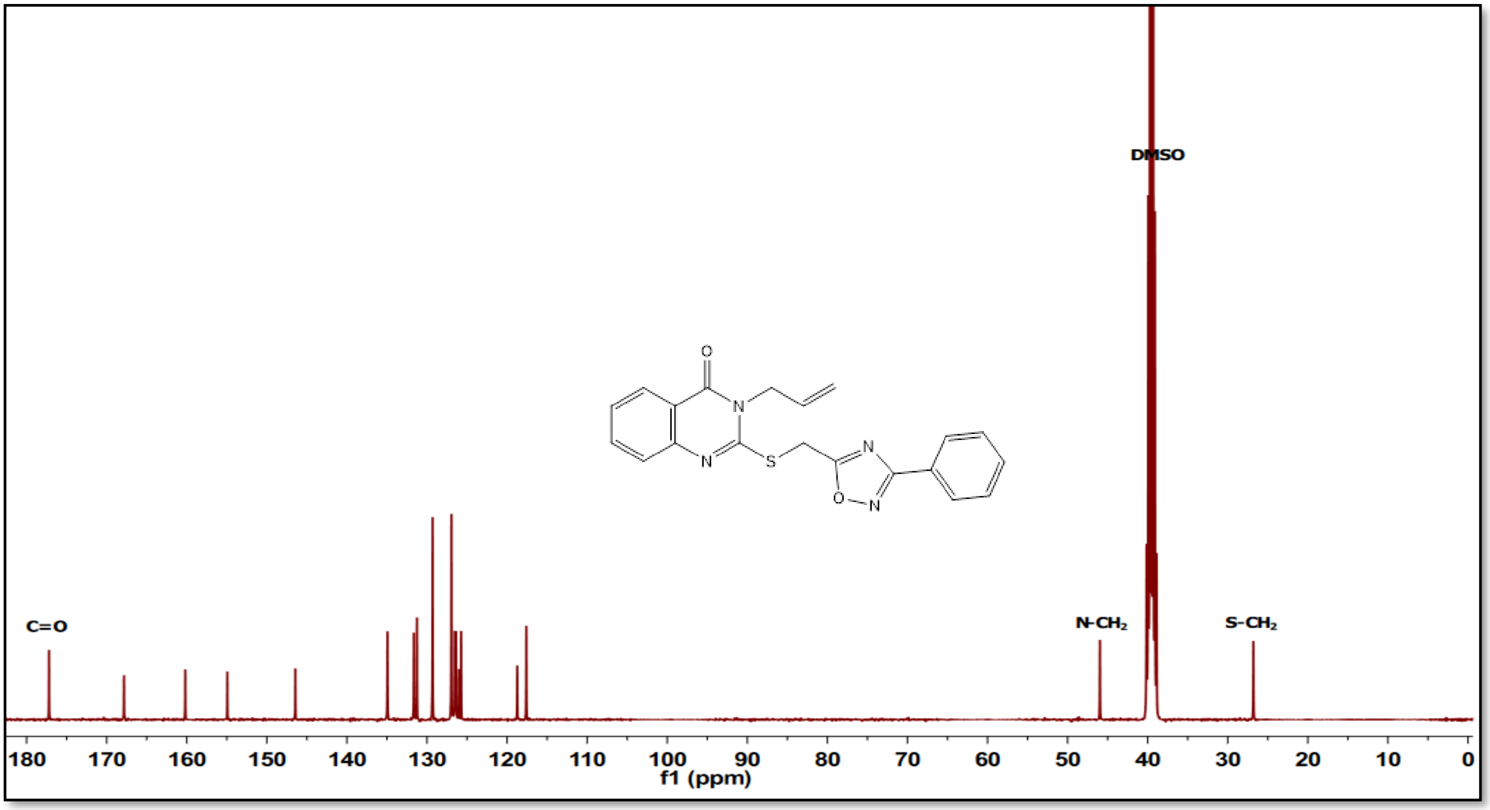

Figure S53: ^{13}C NMR spectrum (100 MHz, DMSO-d_6) of compound **9m**

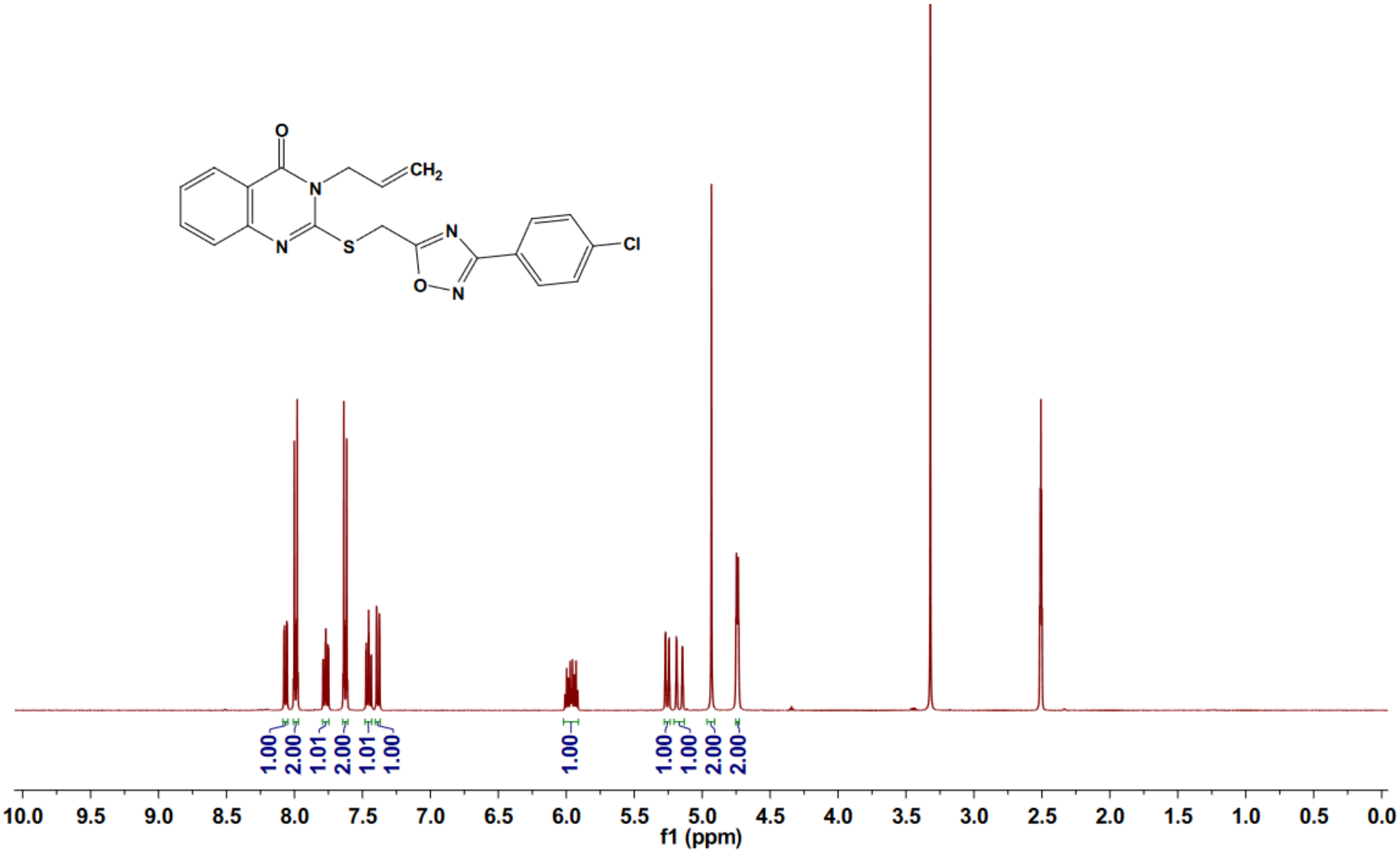

Figure S54: ^{1}H NMR spectrum (400 MHz, DMSO-d_6) of compound **9n**

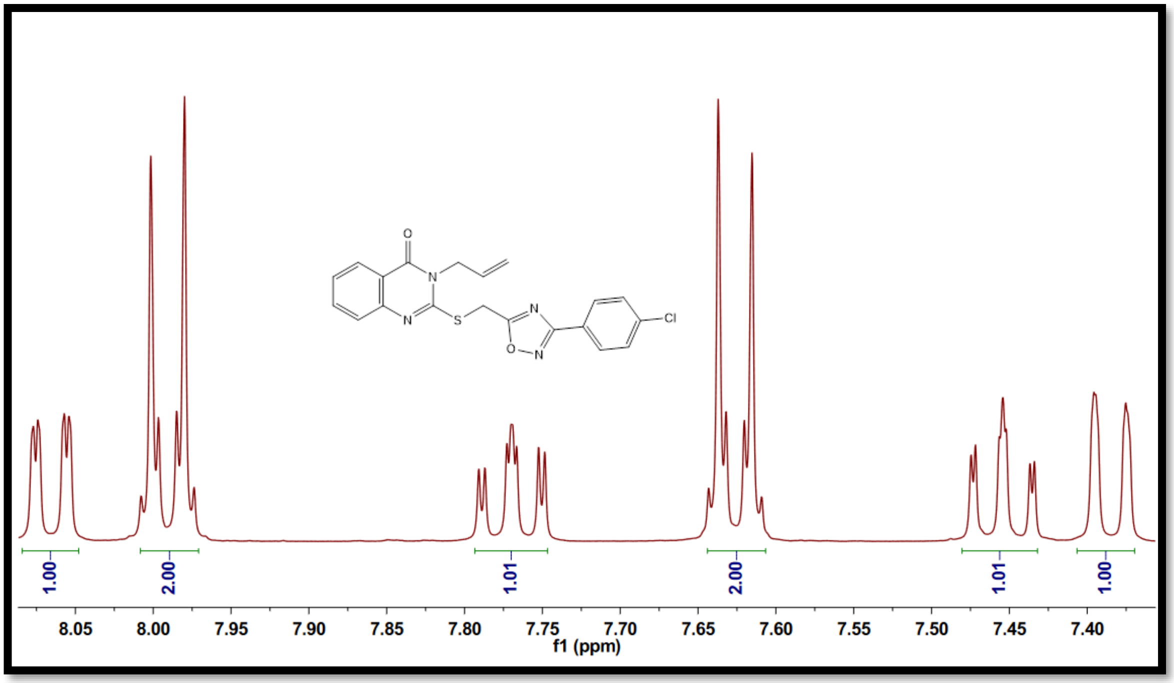

Figure S55: Expanded aromatic region of ^{1}HNMR spectrum (400 MHz, DMSO-d_6) of compound **9n**

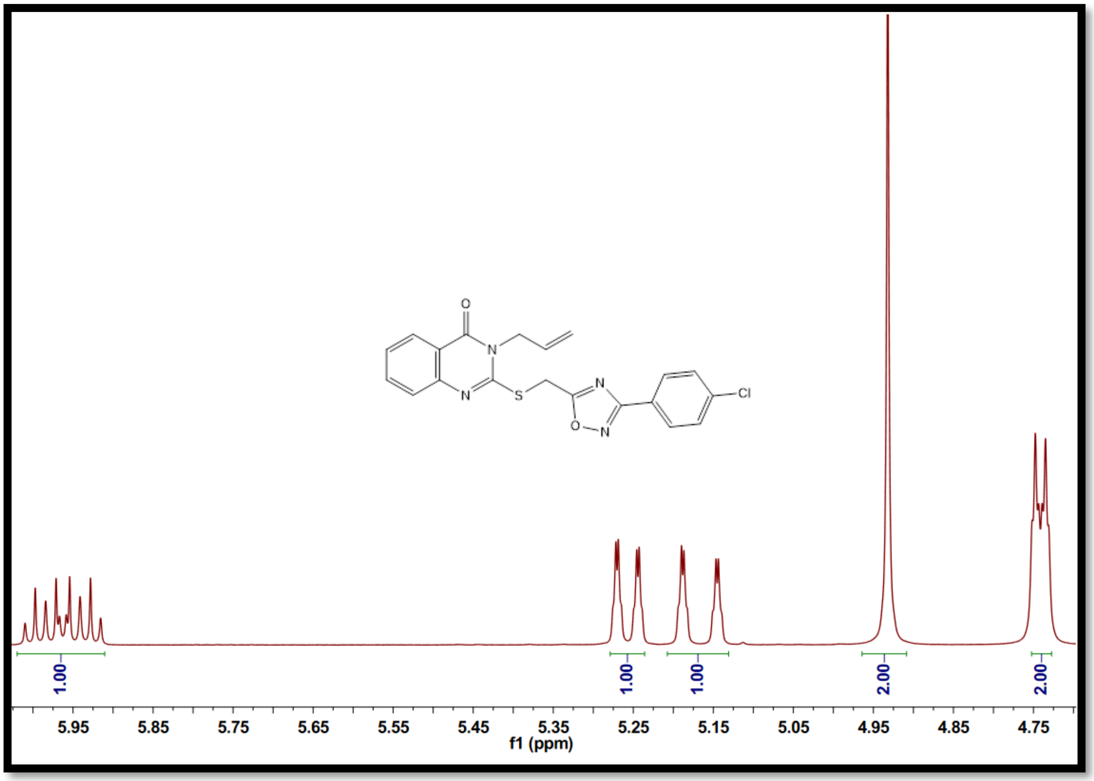

Figure S56: Expanded aliphatic and olefinic regions of ^{1}HNMR spectrum (400 MHz, DMSO-d_6) of compound **9n**

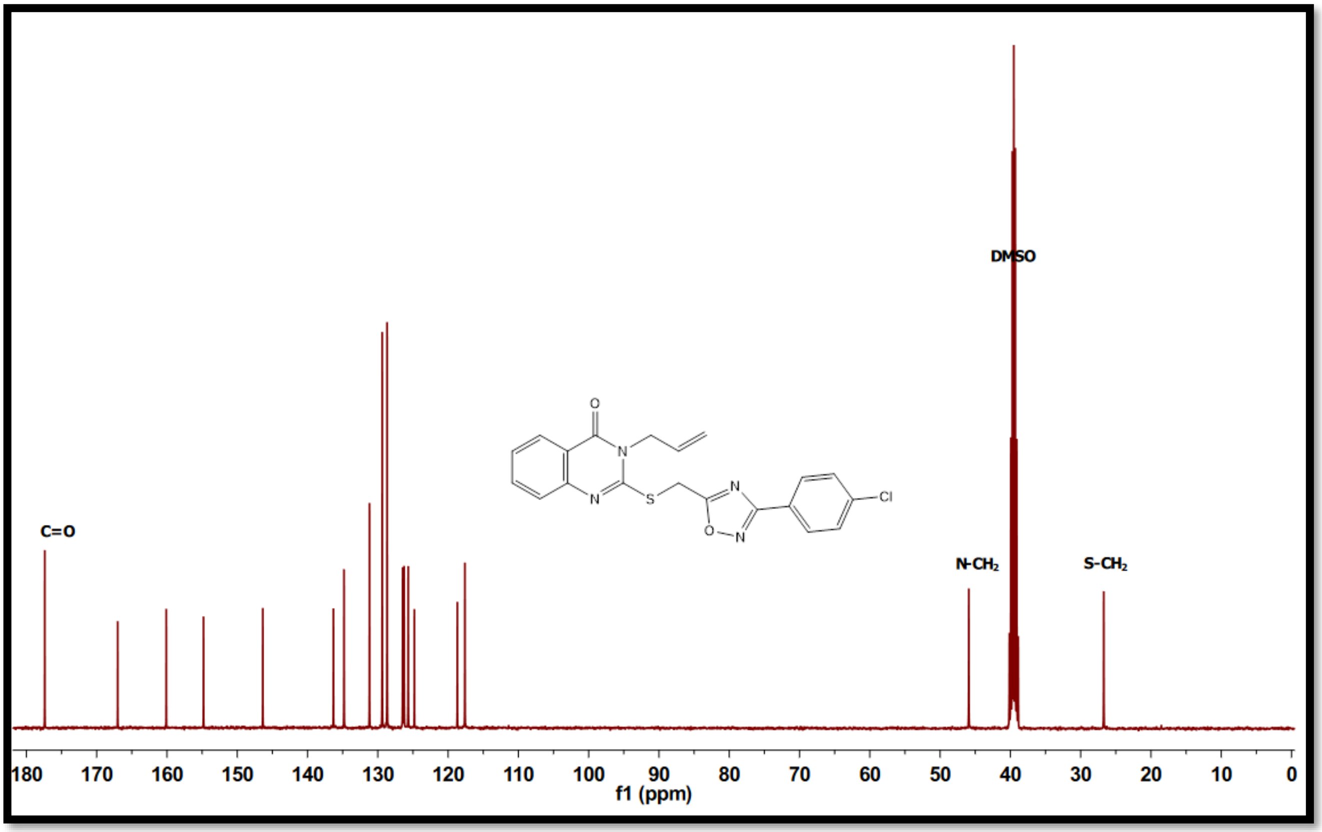

Figure S57: ^{13}C NMR spectrum (100 MHz, DMSO-d_6) of compound **9n**

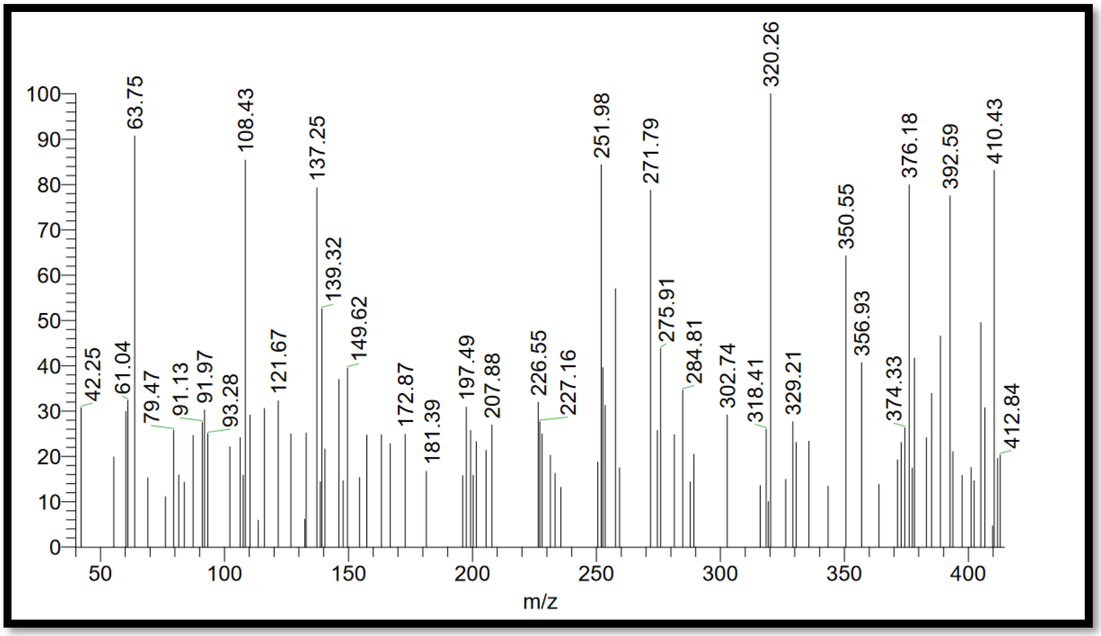

Figure S58: EI-Mass spectrum of compound **9n**

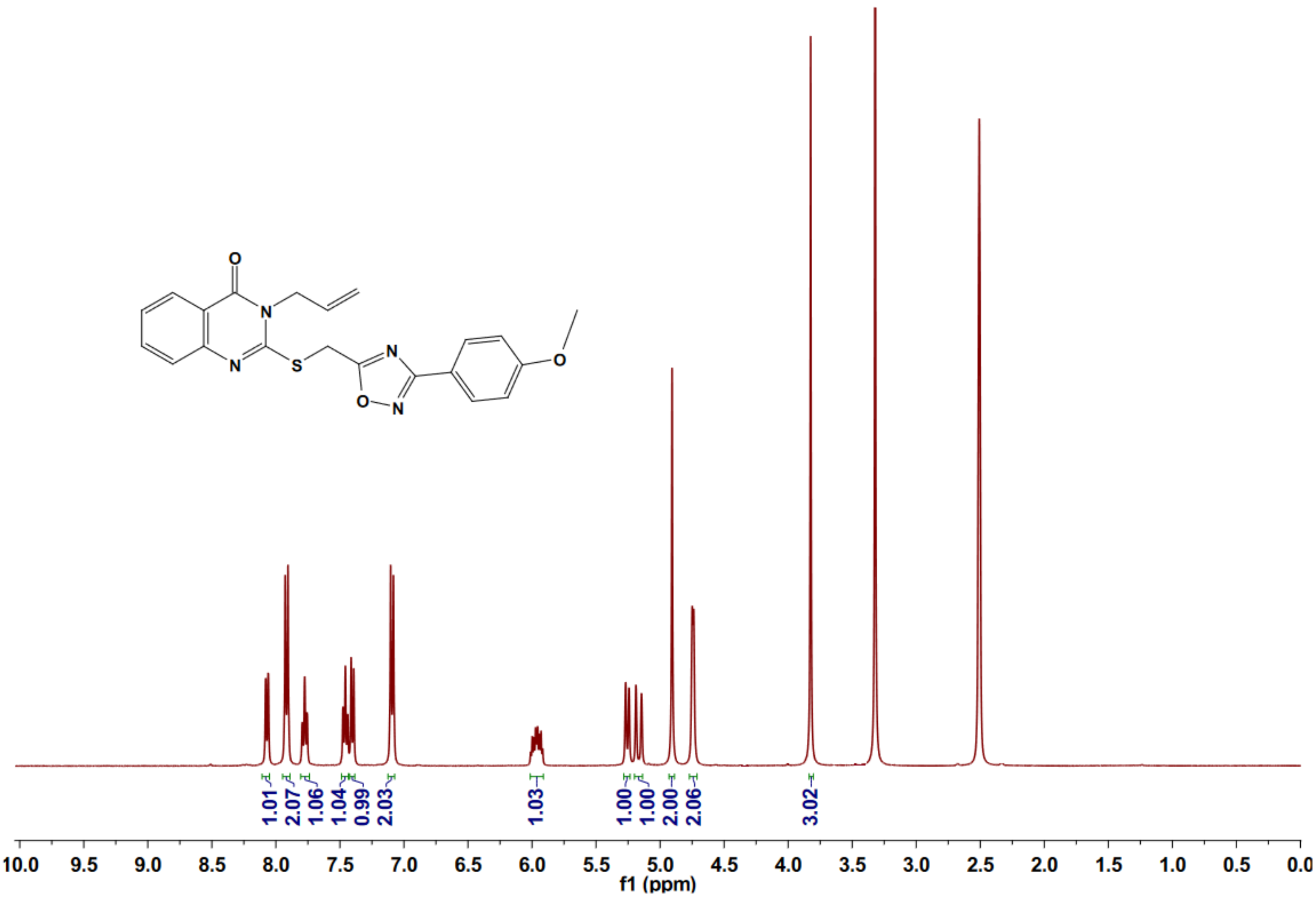

Figure S59: ^{1}H NMR spectrum (400 MHz, DMSO-d_6) of compound **9o**

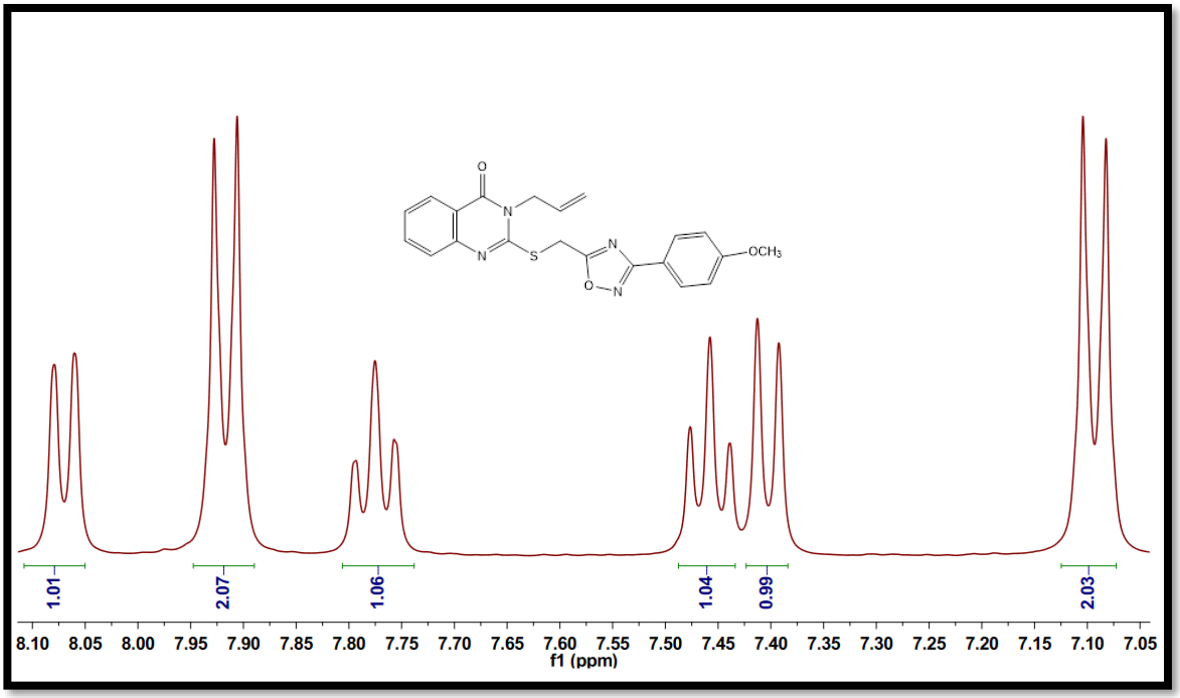

Figure S60: Expanded aromatic region of ^{1}H NMR spectrum (400 MHz, DMSO-d_6) of compound **9o**

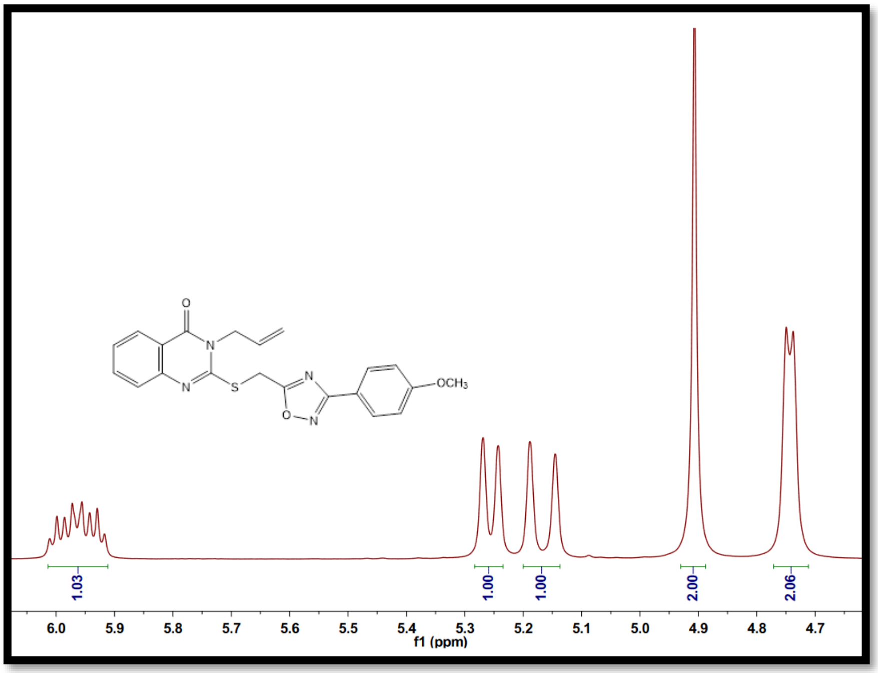

Figure S61: Expanded aliphatic and olefinic regions of ^{1}H NMR spectrum (400 MHz, DMSO-d_6) of compound **9o**

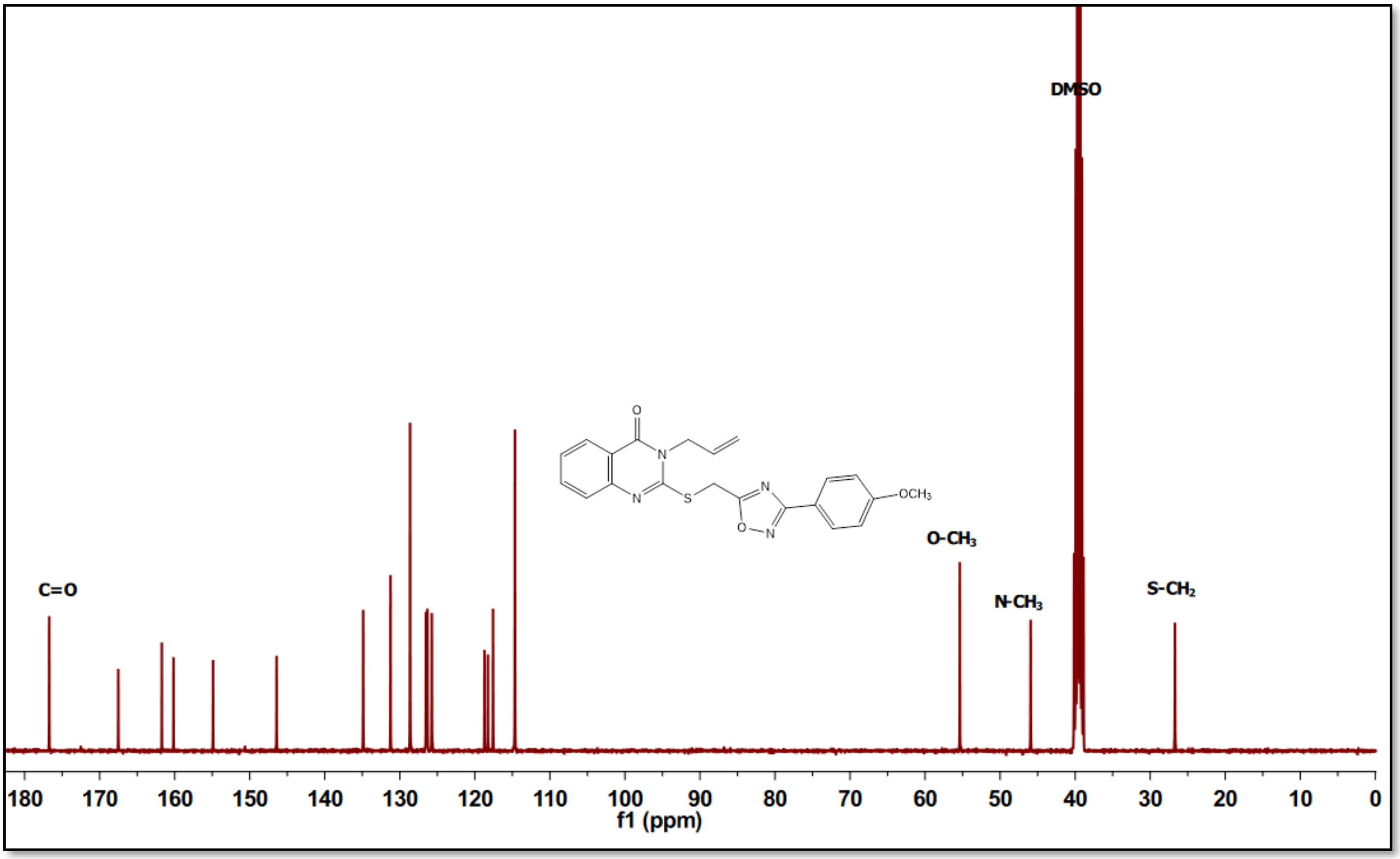

Figure S62: ^{13}C NMR spectrum (100 MHz, DMSO-d_6) of compound **9o**

Al-Azhar University
The Regional Center for Mycology and Biotechnology

Requester Data:

Name: **Dr. Amira Mahmoud**

Authority: Assuit University

Sample Data:

Fifteen samples had been submitted for elemental analysis.

Analysis Report:

Sample Code	C%	H%	N%	S%
AM001p	61.97	3.50	12.71	7.28
AM002m	62.39	3.85	12.40	6.89
AM003A	58.70	3.73	13.91	7.94
AM004E	57.49	3.86	14.27	8.12
AM005P	64.91	4.27	12.89	7.23
AM006m	65.62	4.61	12.49	7.14
AM007A	62.29	4.53	14.02	7.81
AM008E	61.13	4.74	14.37	8.20
AM009	62.37	3.80	12.42	6.89
AM010	65.62	4.61	12.41	6.98
AM011	66.81	3.85	13.82	7.85
AM012	67.36	4.09	13.41	7.60
AM013	64.05	4.42	15.17	8.64
AM014	62.89	4.51	15.62	8.71
AM015	67.34	4.43	13.40	7.68

INVESTIGATOR DIRECTOR

Al-Azhar University Campus - Nasr City, Cairo, Egypt.
Tel: 0202 22620373 Fax : 0202 22620373
E.mail:rcmb@azhar.edu.eg
Website: http://www.azhar.edu.eg.htm * http://www.azhar.edu.eg/pages/fungi_center.htm
Facebook : RCMB AZHAR P.O. box mail : 11751 Nasr City Cairo, Egypt.

Old code	New code
AMoo1P	9b
AM002m	9h
AM003A	9n
AM004E	9k
AM005P	9c
AM006m	9i
AM007A	9o
AM008E	9l
AM009	9e
AM010	9f
AM011	9a
AM012	9g
AM013	9m
AM014	9j
AM015	9d

Oxadiazoles Docking Poses Within EGFR, $EGFR^{T790M}$, & $BRAF^{V600E}$ Active Sites

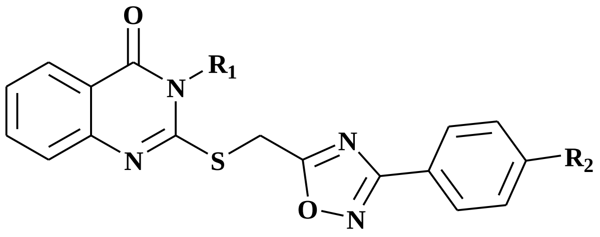

(9a-o)

9a

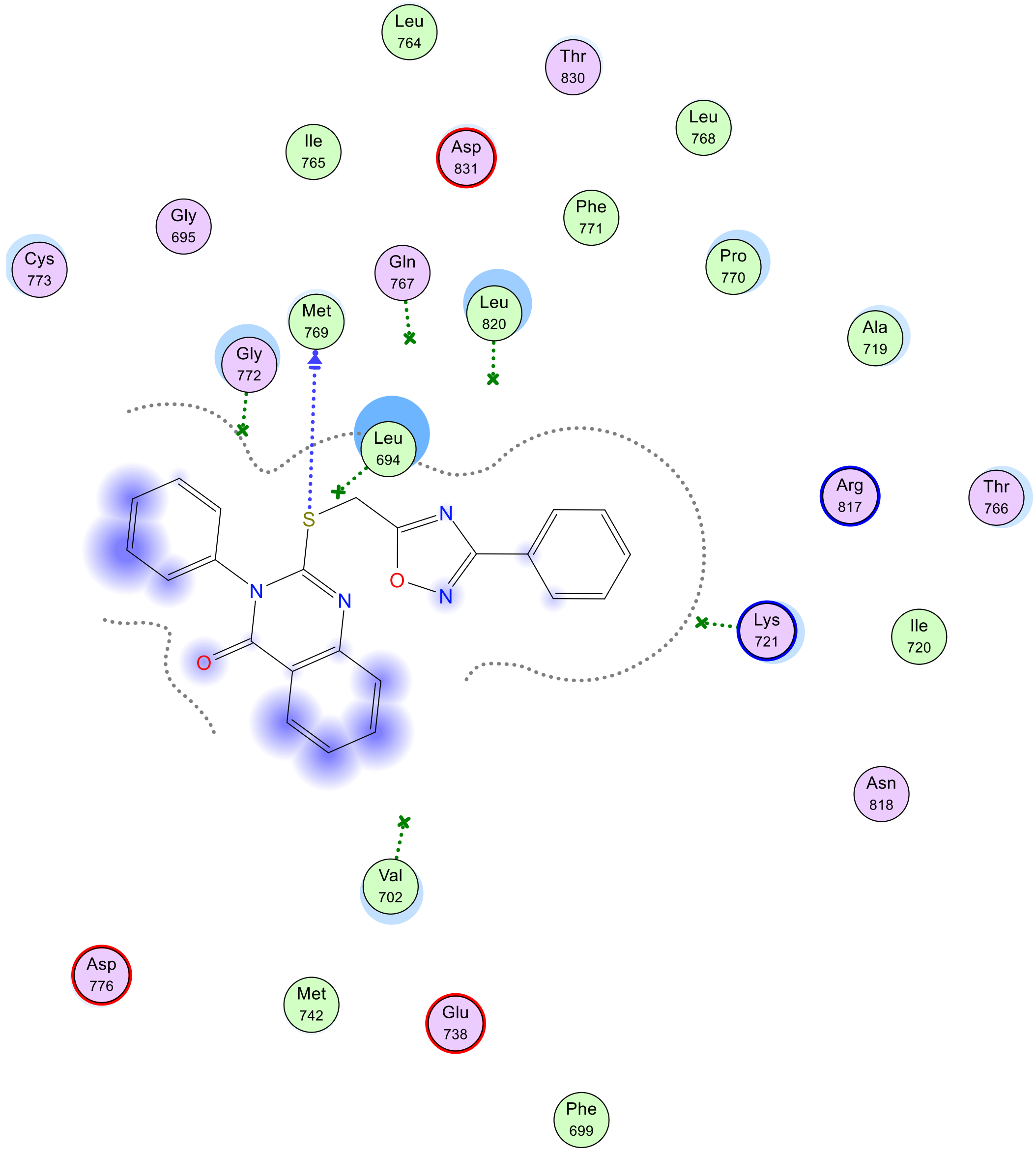

9b

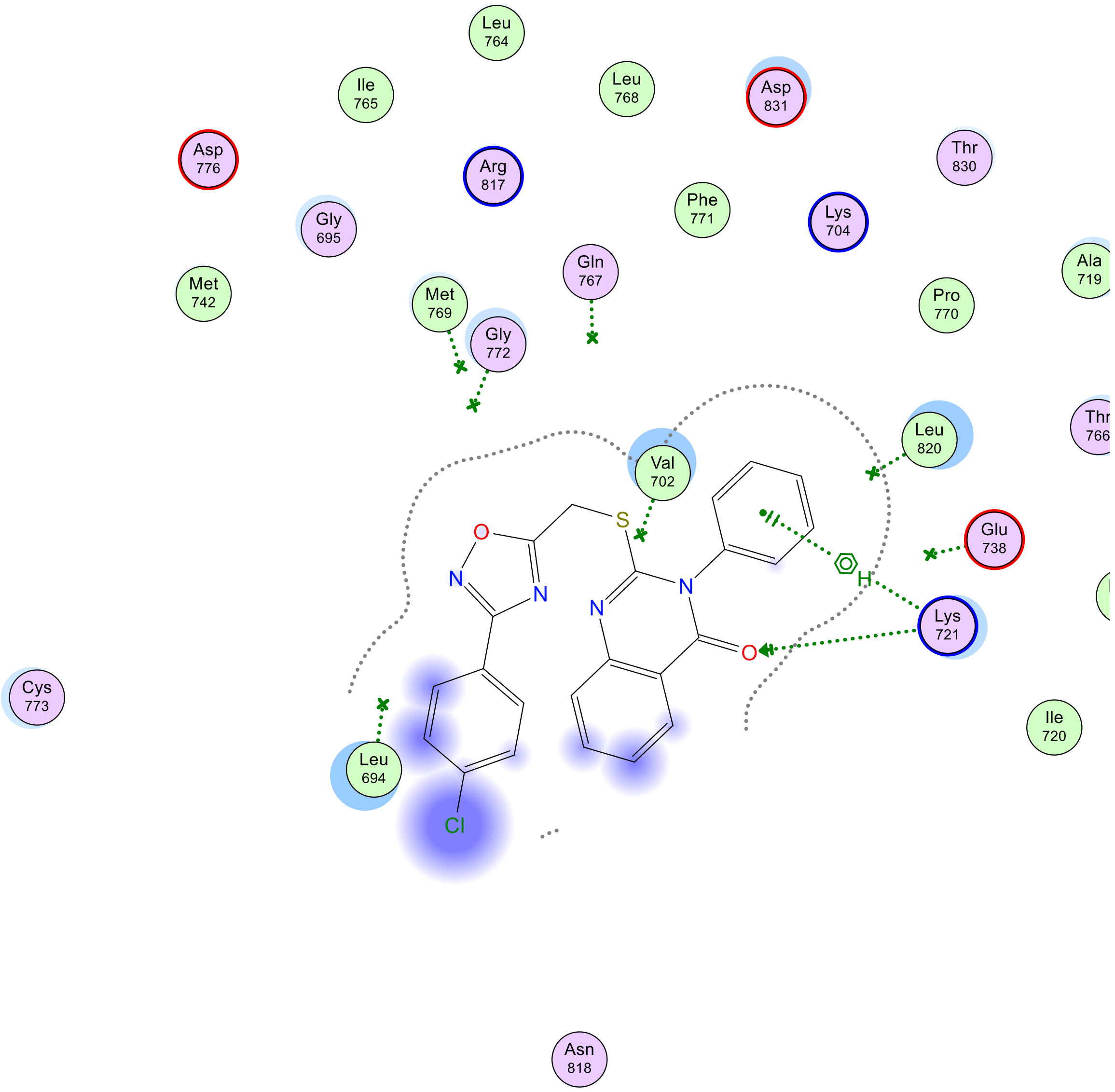
Leu 764
Ile 765
Leu 768
Asp 831
Asp 776
Arg 817
Thr 830
Gly 695
Phe 771
Lys 704
Ala 719
Met 742
Met 769
Gln 767
Pro 770
Gly 772
Leu 820
Thr 766
Val 702
S
O
N
N
N
N
O
H
Glu 738
Lys 721
Cys 773
Leu 694
Cl
Ile 720
Asn 818

9c

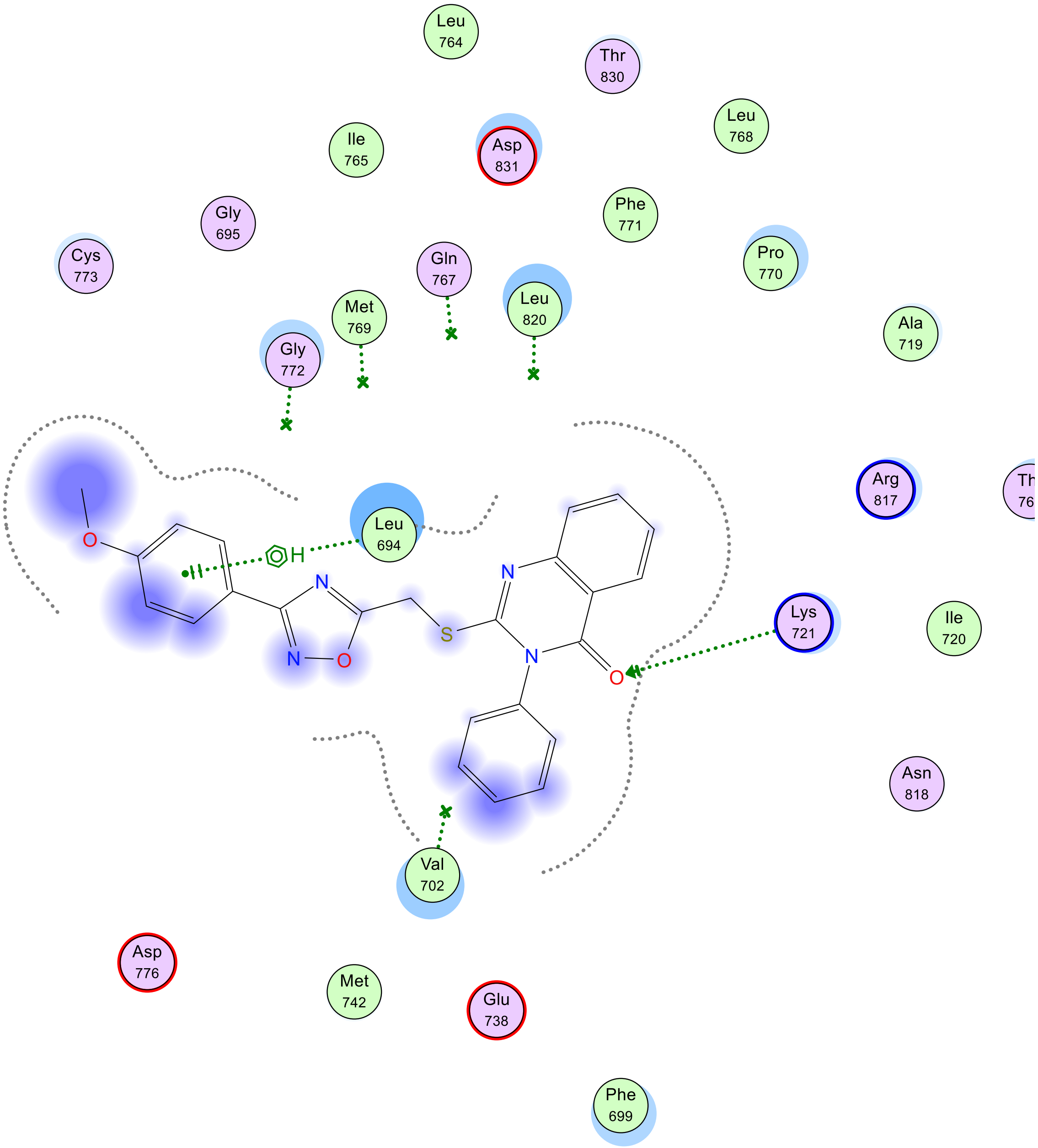
Leu 764
Thr 830
Leu 768
Ile 765
Asp 831
Gly 695
Phe 771
Cys 773
Gln 767
Pro 770
Met 769
Leu 820
Ala 719
Gly 772
Arg 817
Leu 694
Lys 721
Ile 720
Asn 818
Val 702
Asp 776
Met 742
Glu 738
Phe 699

9d

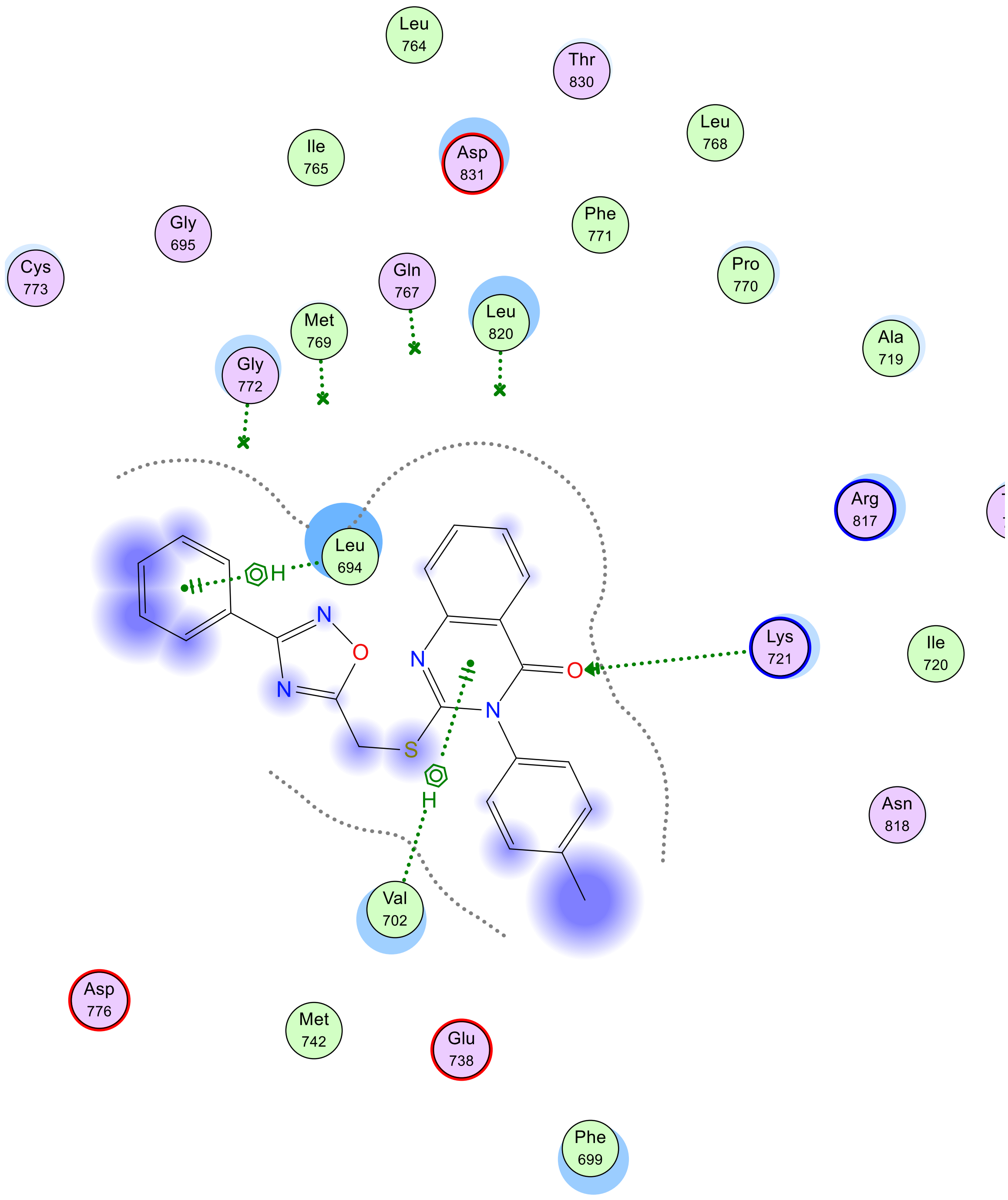
Leu 764
Thr 830
Leu 768
Ile 765
Asp 831
Gly 695
Phe 771
Cys 773
Gln 767
Pro 770
Leu 820
Met 769
Gly 772
Ala 719
Arg 817
Leu 694
Lys 721
Ile 720
Asn 818
Val 702
Asp 776
Met 742
Glu 738
Phe 699

9e

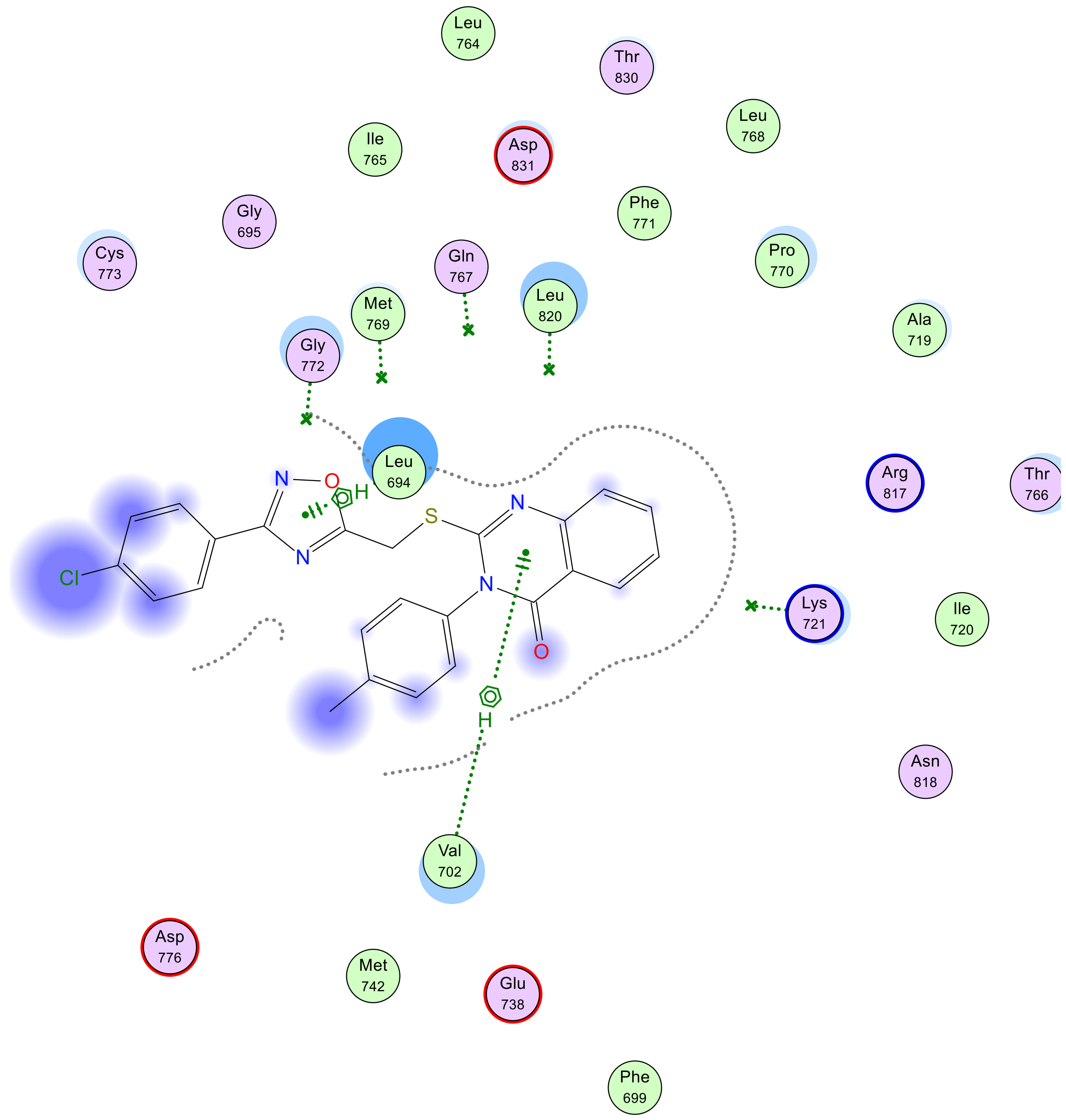

Leu 764
Thr 830
Leu 768
Ile 765
Asp 831
Phe 771
Gly 695
Cys 773
Gln 767
Leu 820
Pro 770
Met 769
Ala 719
Gly 772
Leu 694
Arg 817
Thr 766
N
O
H
S
N
Cl
N
N
Lys 721
Ile 720
O
H
Asn 818
Val 702
Asp 776
Met 742
Glu 738
Phe 699

9f

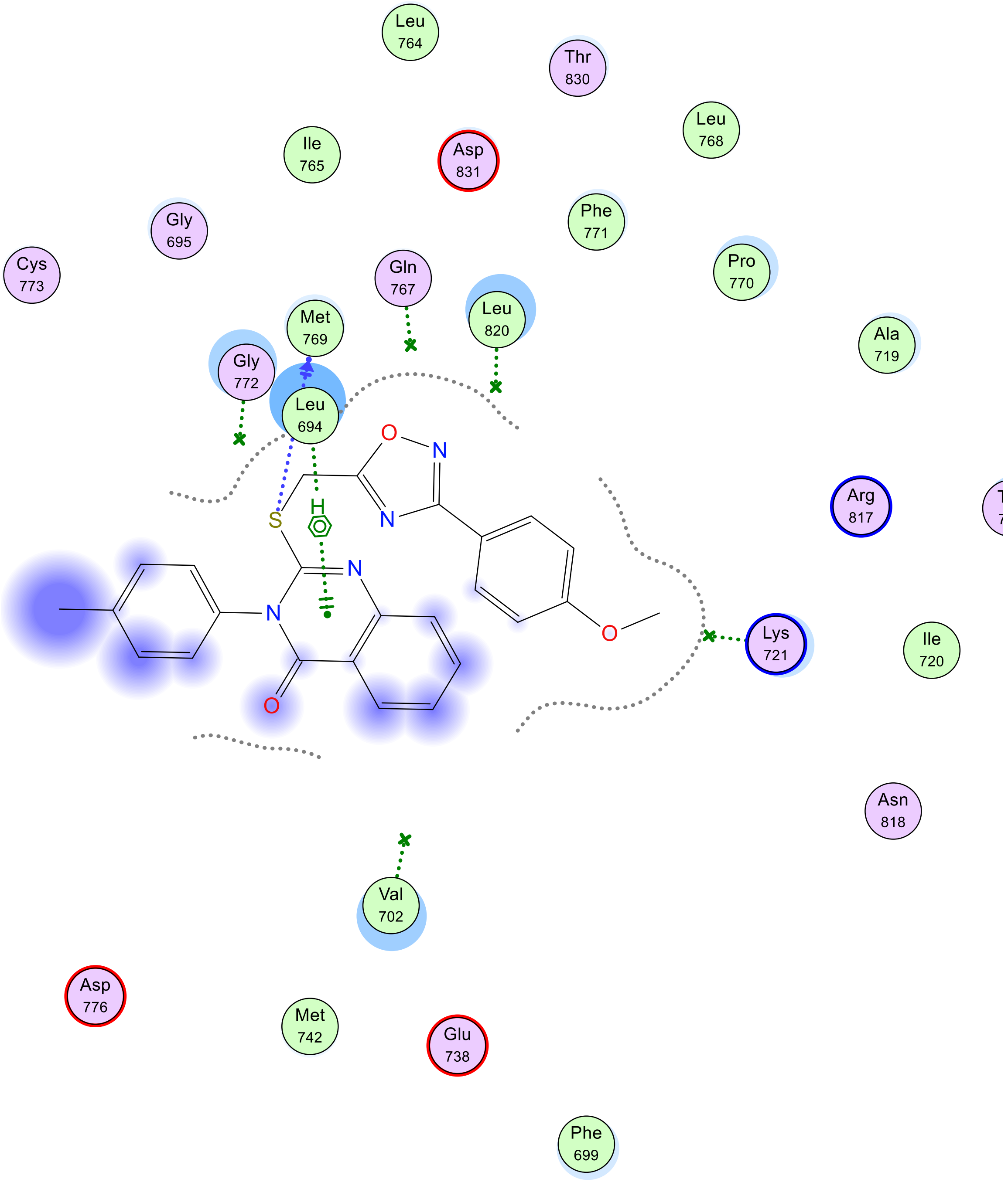

Leu 764
Thr 830
Leu 768
Ile 765
Asp 831
Phe 771
Gly 695
Cys 773
Gln 767
Pro 770
Met 769
Leu 820
Gly 772
Ala 719
Leu 694
O
N
N
S
H
N
Arg 817
N
O
Lys 721
Ile 720
O
Asn 818
Val 702
Asp 776
Met 742
Glu 738
Phe 699

9g

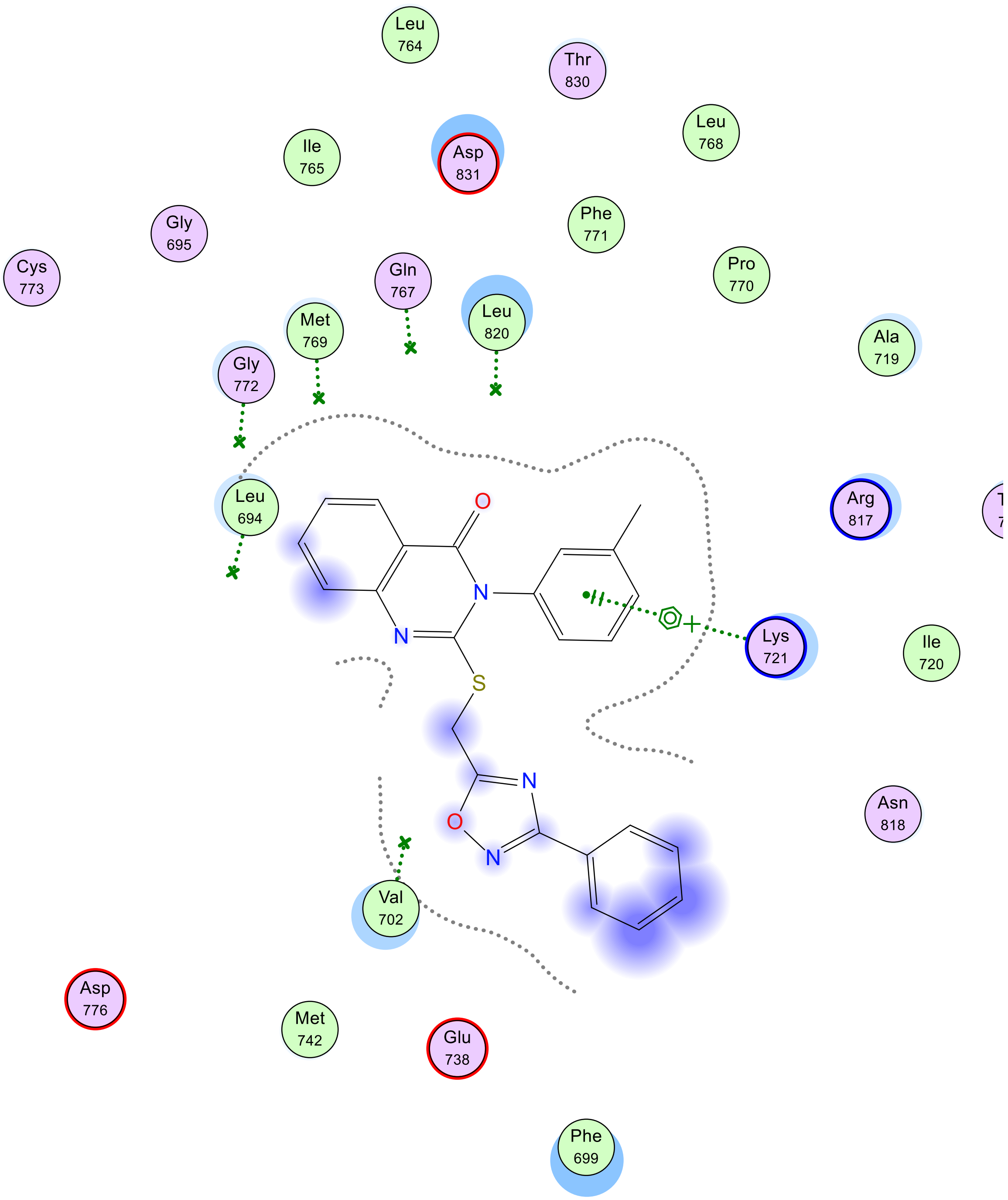
Leu 764
Thr 830
Ile 765
Asp 831
Leu 768
Gly 695
Phe 771
Cys 773
Gln 767
Pro 770
Met 769
Leu 820
Ala 719
Gly 772
Leu 694
Arg 817
O
N
N
Lys 721
Ile 720
S
N
O
N
Asn 818
Val 702
Asp 776
Met 742
Glu 738
Phe 699

9h

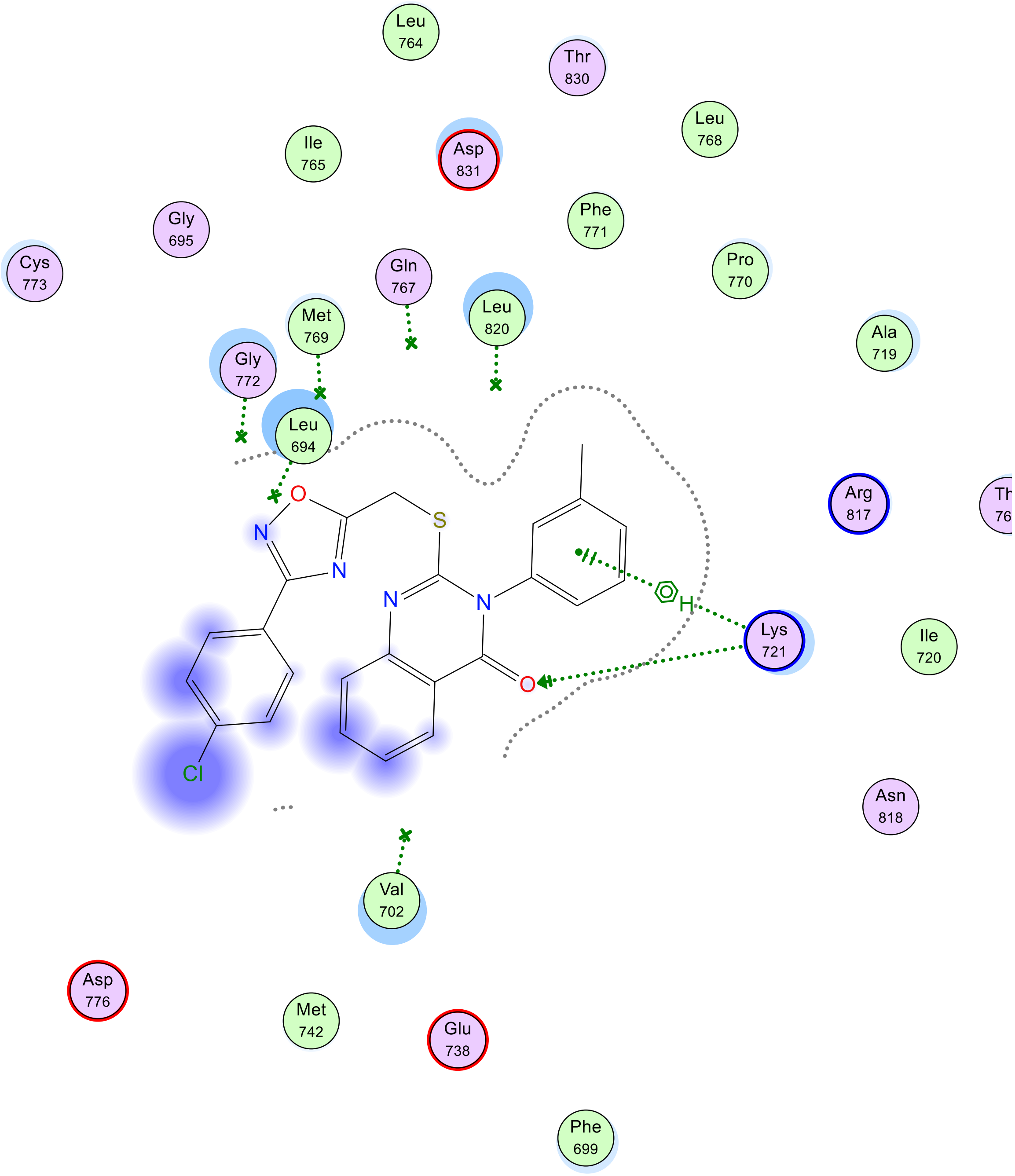

Leu 764
Thr 830
Leu 768
Ile 765
Asp 831
Phe 771
Gly 695
Cys 773
Gln 767
Pro 770
Leu 820
Met 769
Ala 719
Gly 772
Leu 694
Arg 817
O
S
N
N
N
N
H
Lys 721
Ile 720
O
Cl
Asn 818
Val 702
Asp 776
Met 742
Glu 738
Phe 699

9i

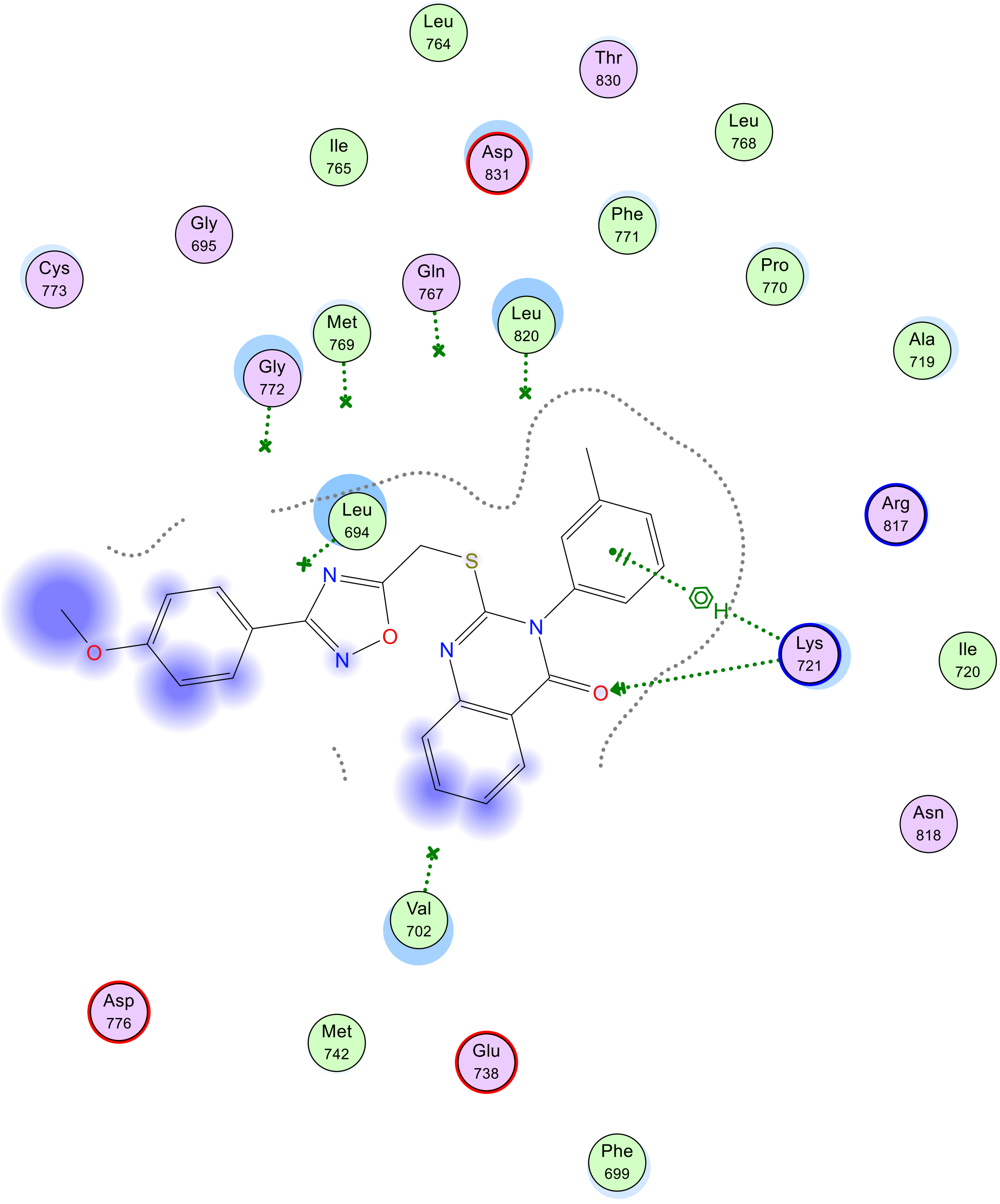

Leu 764
Thr 830
Leu 768
Ile 765
Asp 831
Phe 771
Gly 695
Cys 773
Gln 767
Leu 820
Pro 770
Met 769
Ala 719
Gly 772
Leu 694
Arg 817
S
N
N
O
N
N
O
O
H
Lys 721
Ile 720
Asn 818
Val 702
Asp 776
Met 742
Glu 738
Phe 699

9j

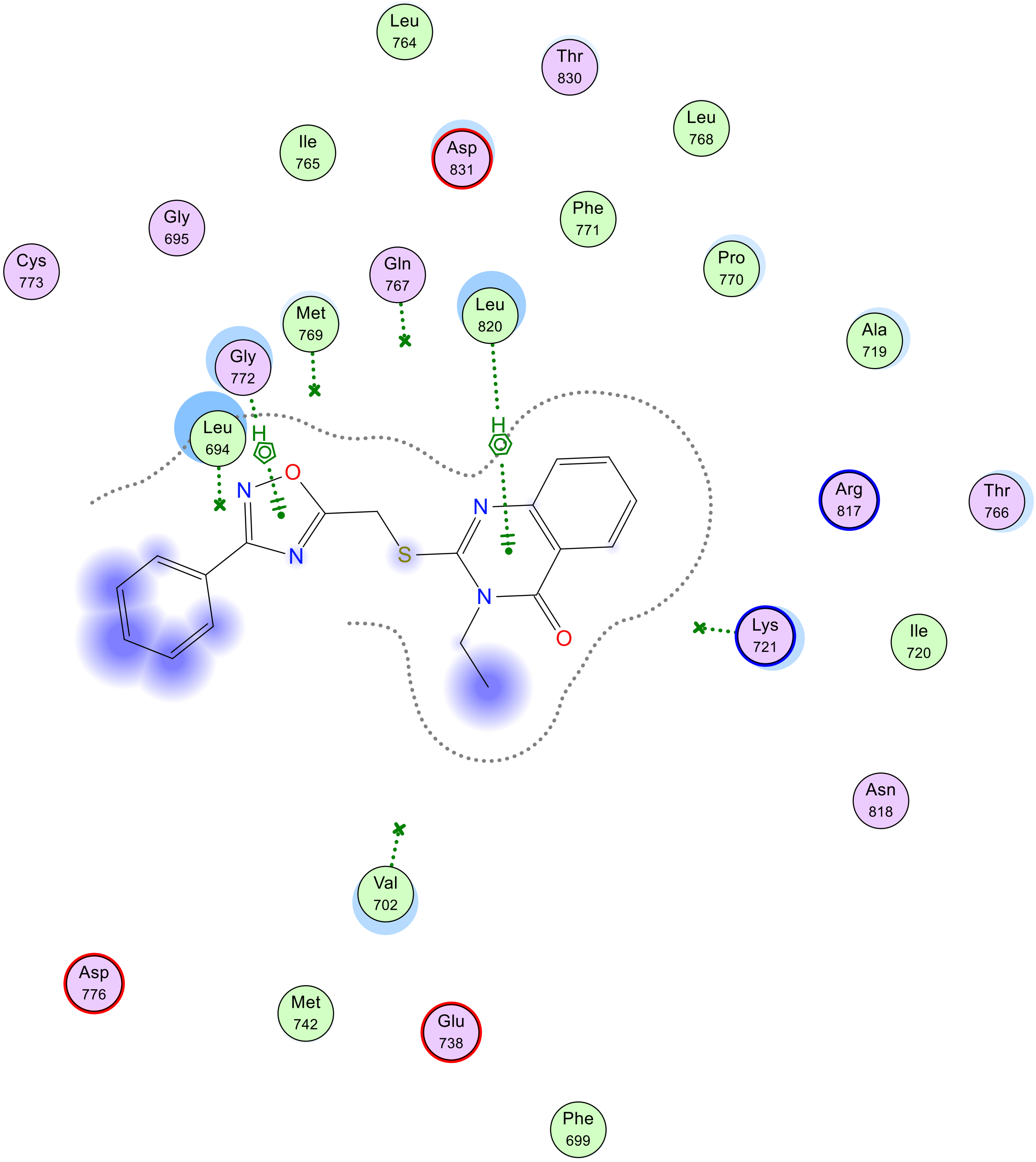

Leu 764
Thr 830
Leu 768
Ile 765
Asp 831
Phe 771
Gly 695
Cys 773
Gln 767
Pro 770
Met 769
Leu 820
Ala 719
Gly 772
Leu 694
H
N
O
H
N
N
S
N
O
Arg 817
Thr 766
Lys 721
Ile 720
Asn 818
Val 702
Asp 776
Met 742
Glu 738
Phe 699

9k

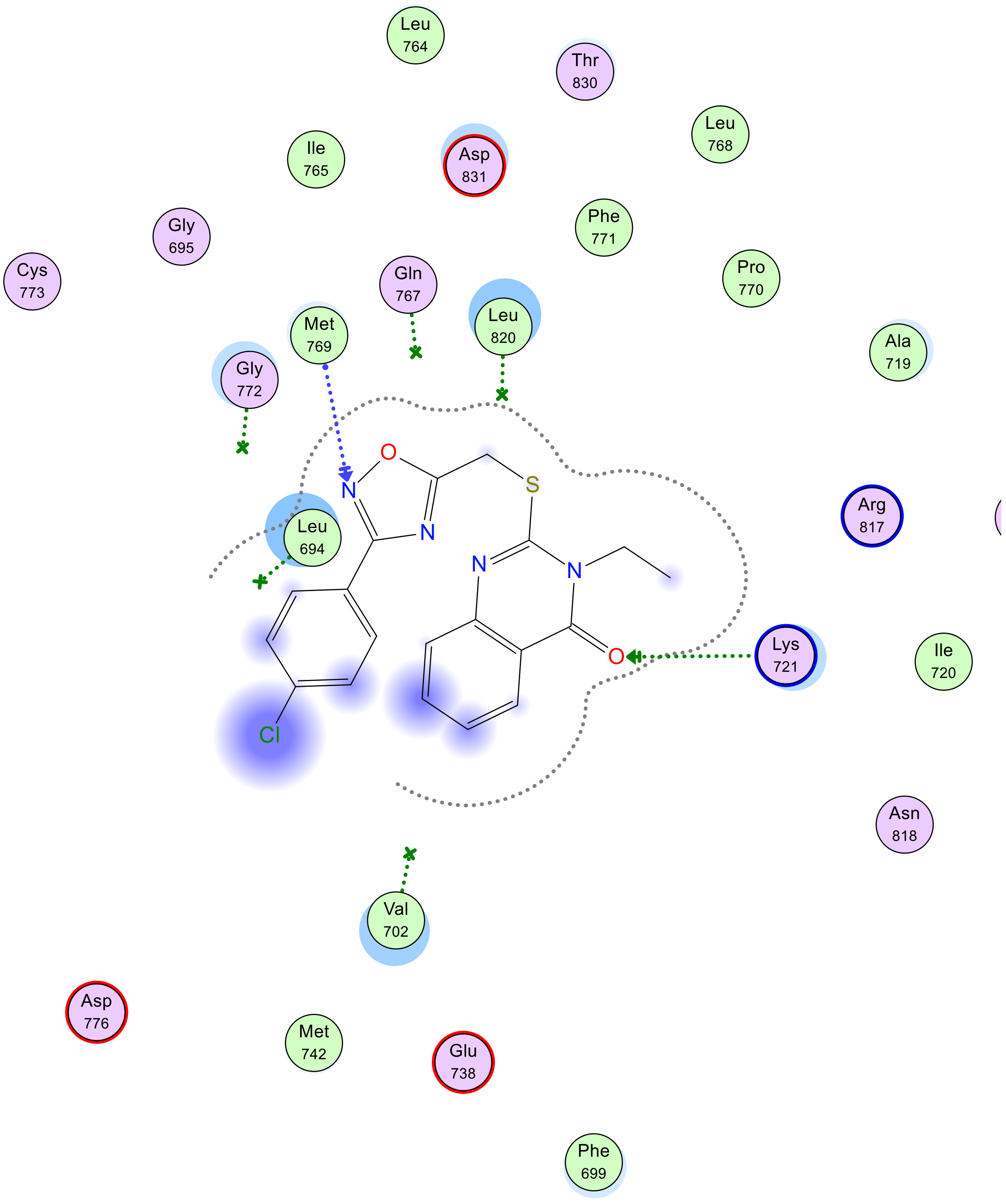

Leu 764
Thr 830
Leu 768
Ile 765
Asp 831
Phe 771
Gly 695
Cys 773
Gln 767
Pro 770
Met 769
Leu 820
Ala 719
Gly 772
Arg 817
Leu 694
Lys 721
Ile 720
Asn 818
Val 702
Asp 776
Met 742
Glu 738
Phe 699

9l

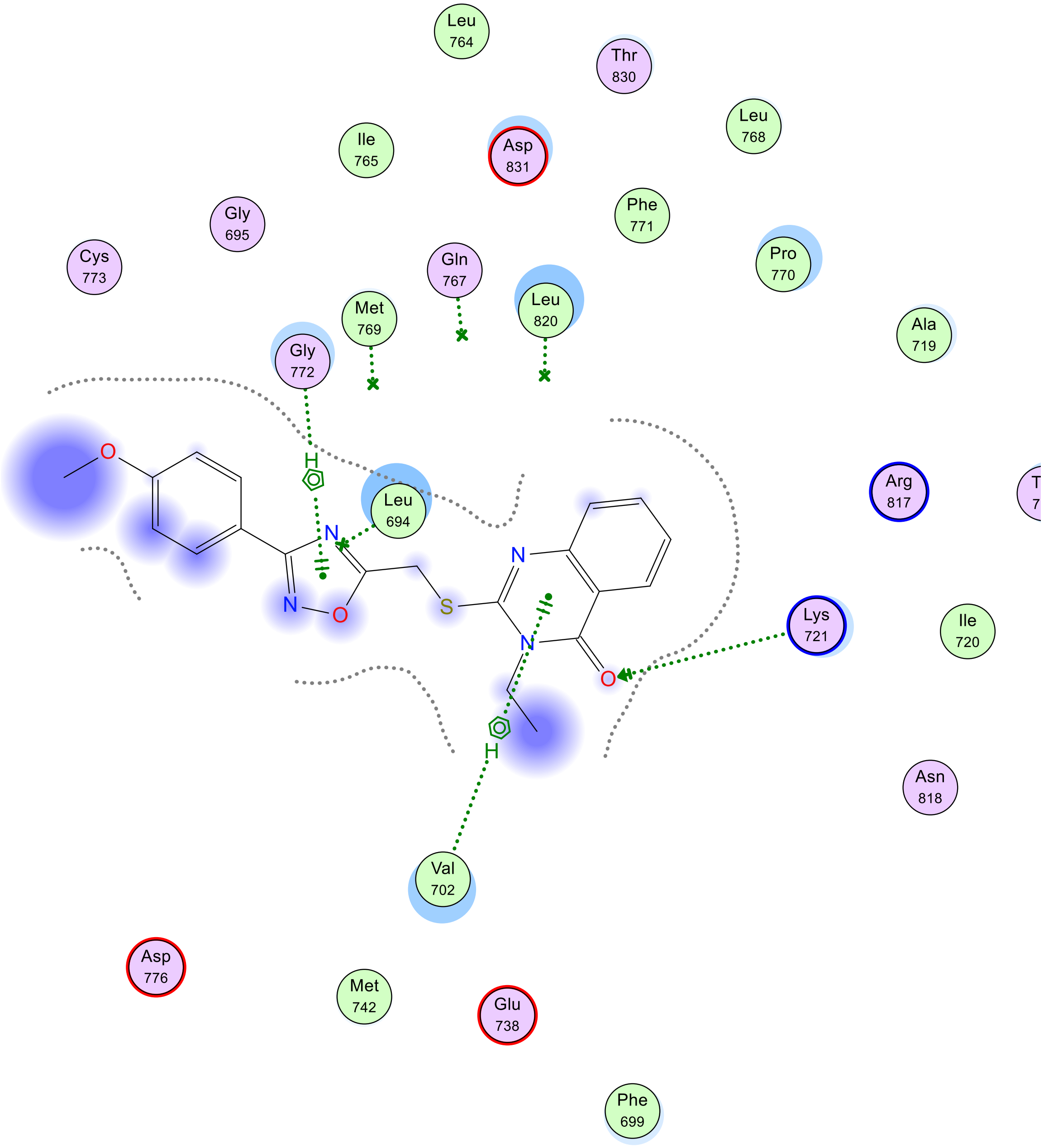

Leu 764
Thr 830
Leu 768
Ile 765
Asp 831
Phe 771
Gly 695
Cys 773
Gln 767
Pro 770
Met 769
Leu 820
Ala 719
Gly 772
Leu 694
Arg 817
Lys 721
Ile 720
Asn 818
Val 702
Asp 776
Met 742
Glu 738
Phe 699

9m

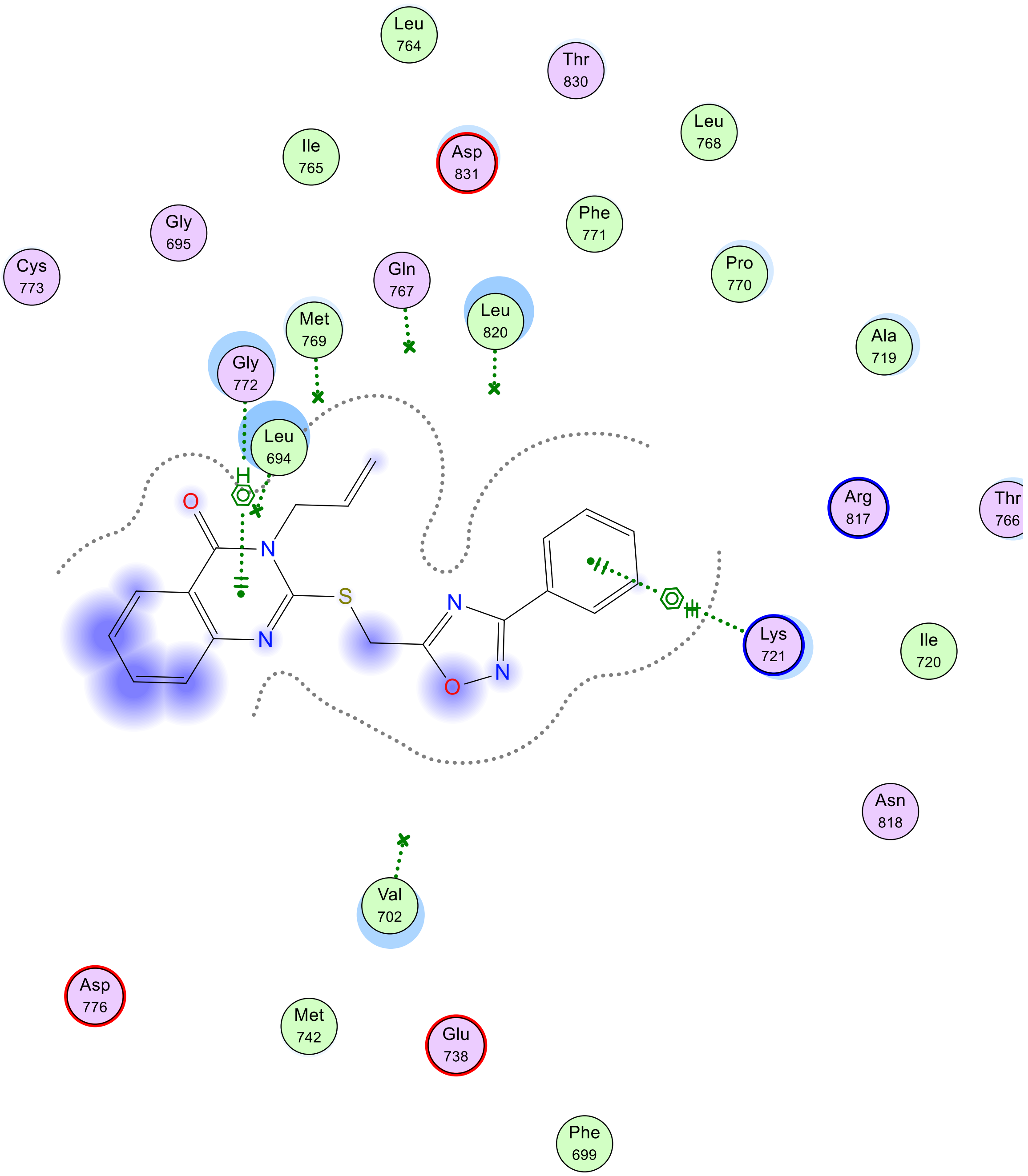

Leu 764
Thr 830
Leu 768
Ile 765
Asp 831
Phe 771
Gly 695
Cys 773
Gln 767
Pro 770
Met 769
Leu 820
Ala 719
Gly 772
Leu 694
Arg 817
Thr 766
Lys 721
Ile 720
Asn 818
Val 702
Asp 776
Met 742
Glu 738
Phe 699

9n

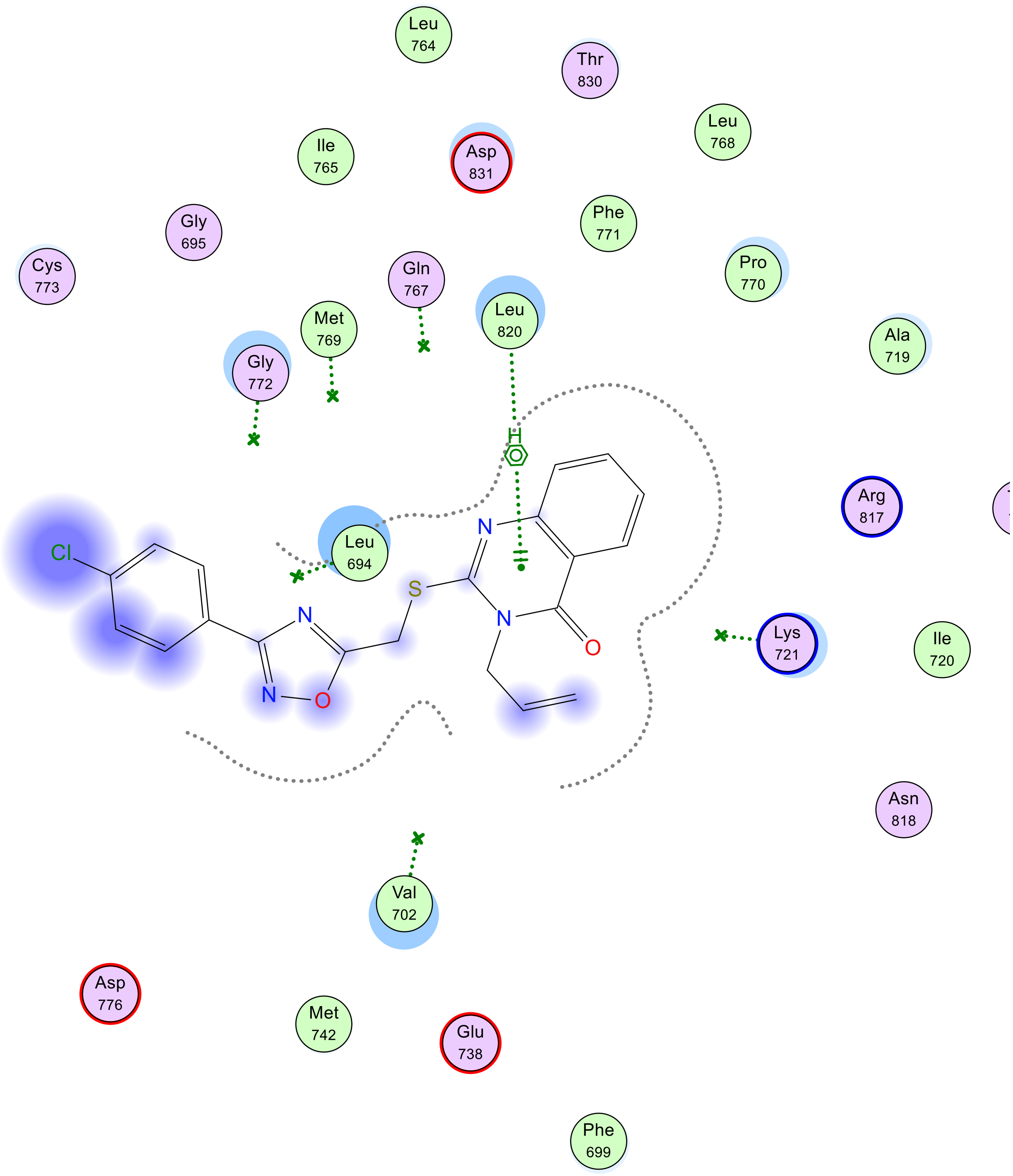

Leu 764
Thr 830
Leu 768
Ile 765
Asp 831
Phe 771
Gly 695
Cys 773
Gln 767
Pro 770
Met 769
Leu 820
Ala 719
Gly 772
Arg 817
Cl
Leu 694
N
S
N
N
O
Lys 721
Ile 720
N
O
Asn 818
Val 702
Asp 776
Met 742
Glu 738
Phe 699

9o

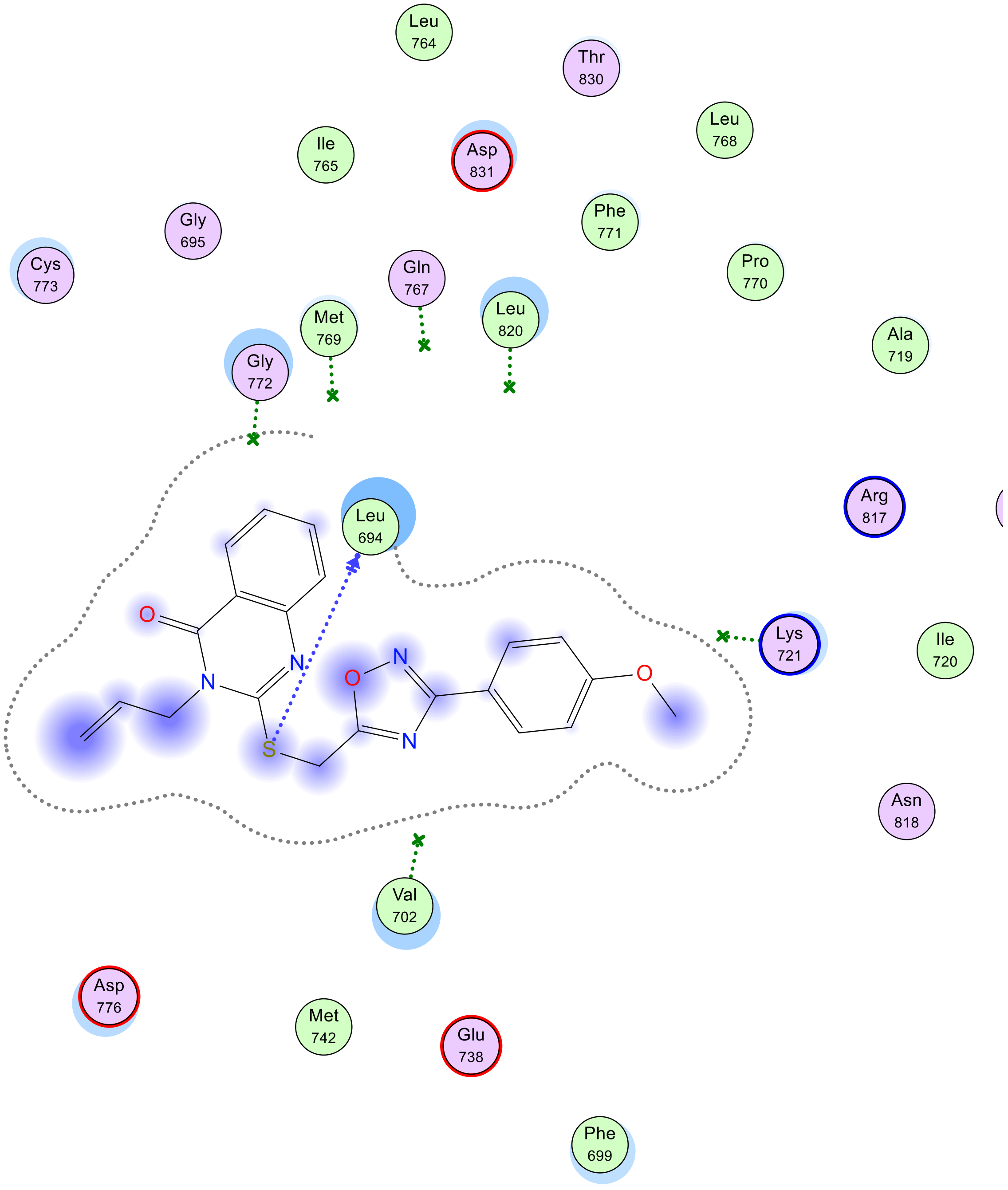

Leu 764
Thr 830
Leu 768
Ile 765
Asp 831
Phe 771
Gly 695
Cys 773
Gln 767
Pro 770
Met 769
Leu 820
Ala 719
Gly 772
Leu 694
Arg 817
Lys 721
Ile 720
Asn 818
Val 702
Asp 776
Met 742
Glu 738
Phe 699

Erlotinib

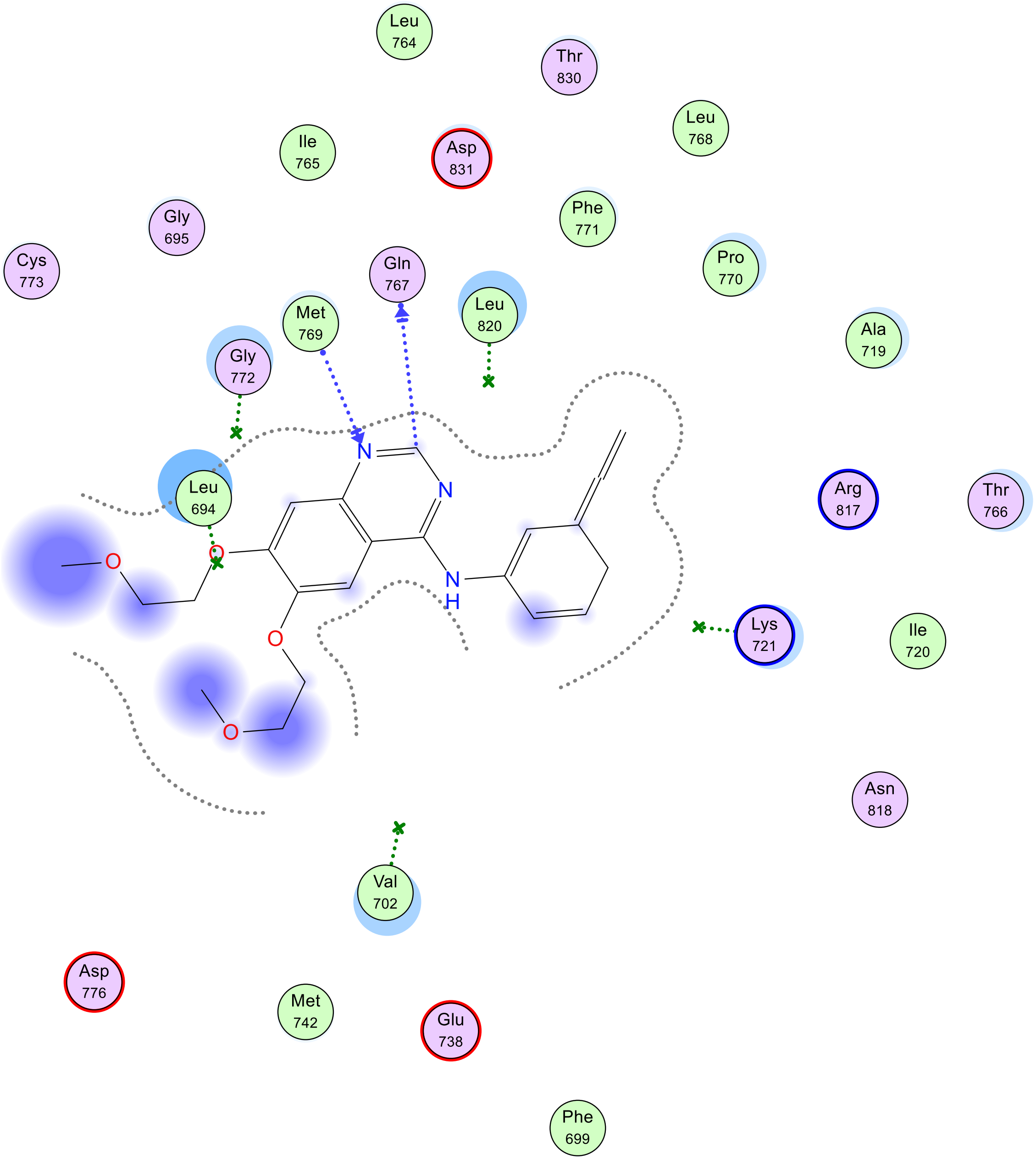

Fig. S.I. ????? Docking poses of derivatives (9a-o) within EGFR (PDB ID: 1M17) active site showing H-bonding and H-Pi interactions with key amino acid residues (Met 769, Glu 767, Lys 721, Leu 684, Val 702).

9a

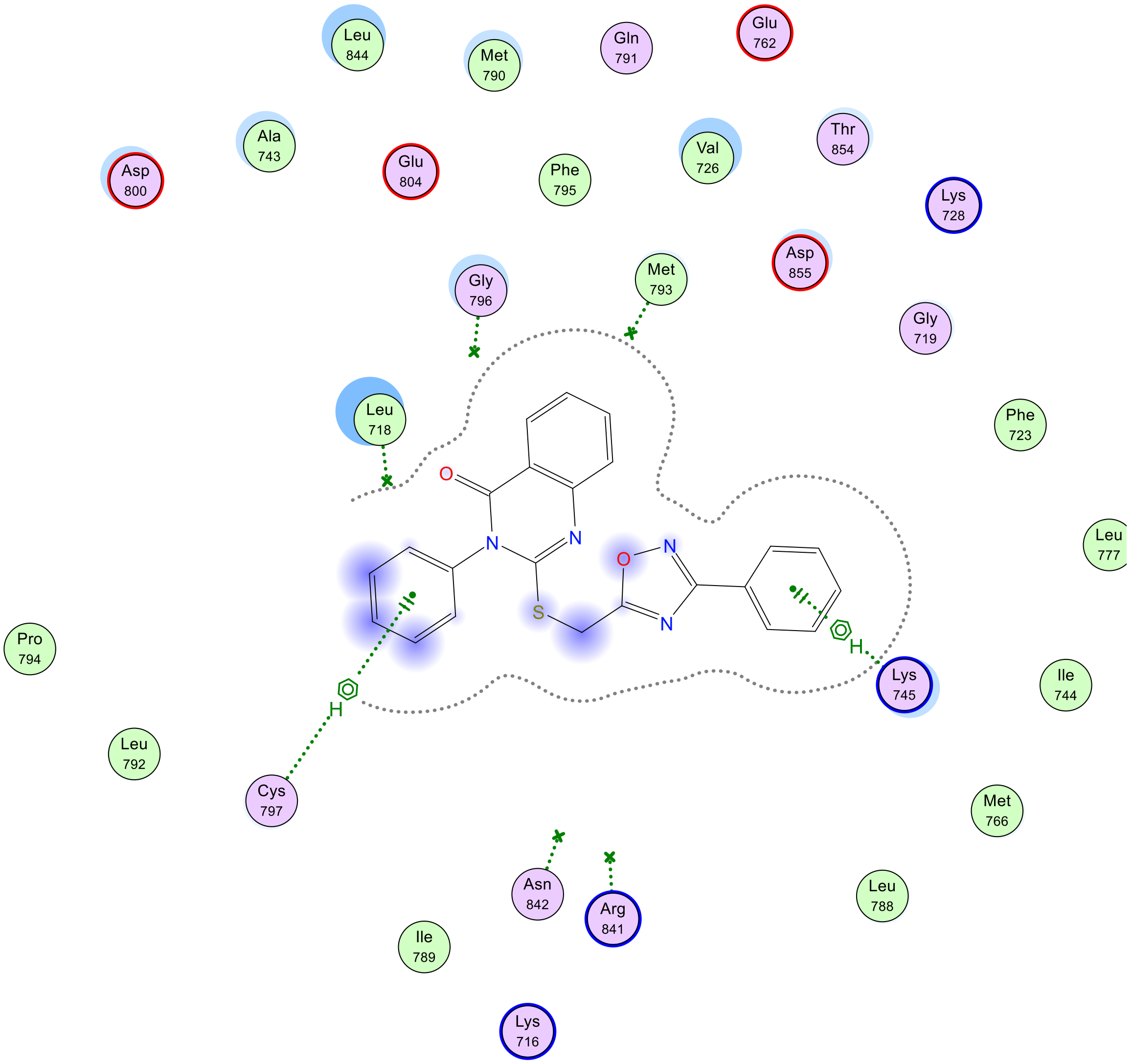

Leu 844
Met 790
Gln 791
Glu 762
Asp 800
Ala 743
Glu 804
Phe 795
Val 726
Thr 854
Lys 728
Gly 796
Met 793
Asp 855
Gly 719
Leu 718
Phe 723
O
N
N
O
N
N
S
H
H
Leu 777
Pro 794
Lys 745
Ile 744
Leu 792
Cys 797
Met 766
Asn 842
Arg 841
Leu 788
Ile 789
Lys 716

9b

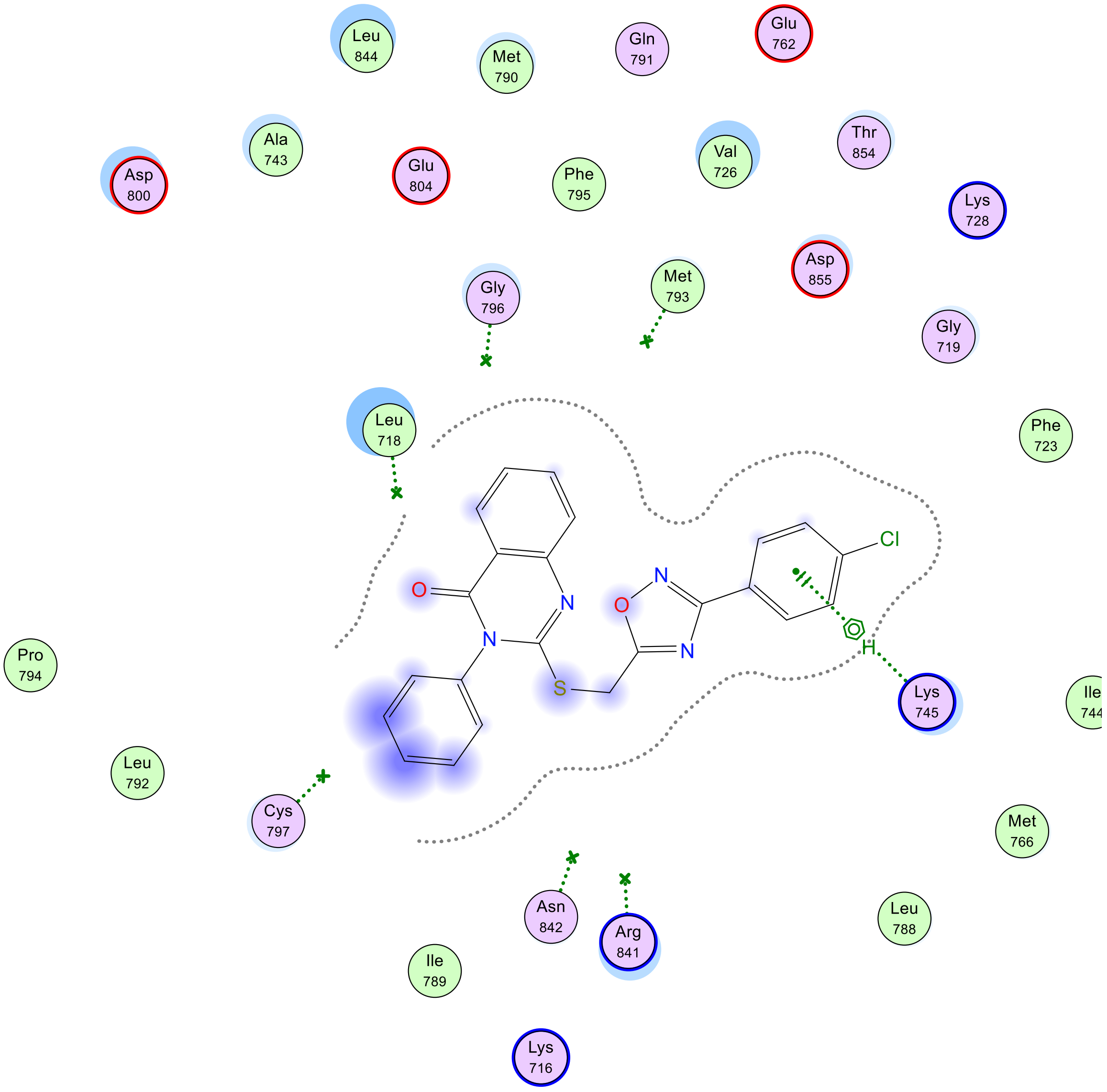

Leu 844
Met 790
Gln 791
Glu 762
Asp 800
Ala 743
Glu 804
Phe 795
Val 726
Thr 854
Lys 728
Asp 855
Gly 796
Met 793
Gly 719
Leu 718
Phe 723
Cl
O
N
S
H
Pro 794
Lys 745
Leu 792
Cys 797
Met 766
Asn 842
Arg 841
Leu 788
Ile 789
Lys 716

9c

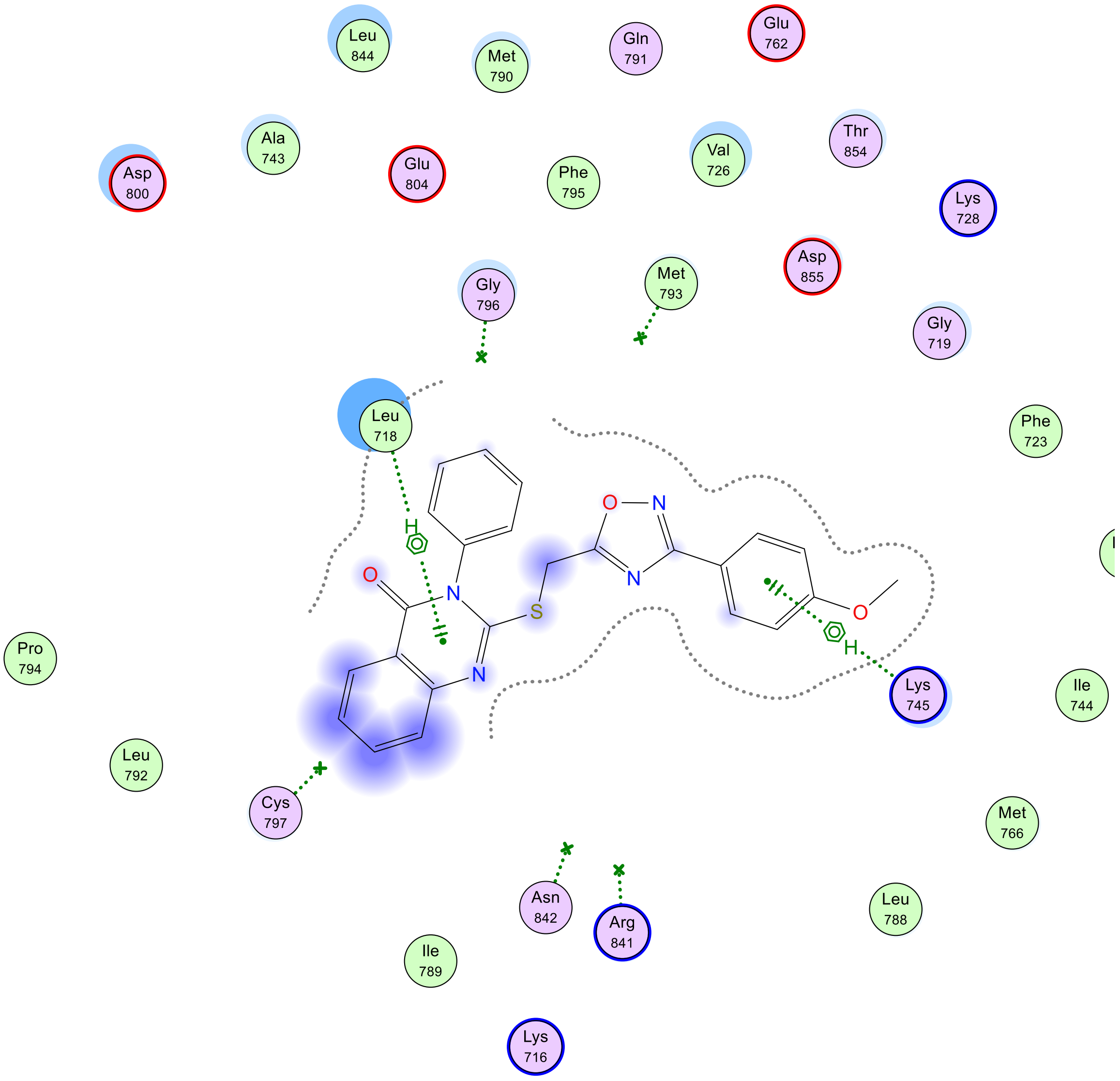

Leu 844
Met 790
Gln 791
Glu 762
Ala 743
Asp 800
Glu 804
Phe 795
Val 726
Thr 854
Lys 728
Asp 855
Gly 796
Met 793
Gly 719
Leu 718
Phe 723
Pro 794
Lys 745
Ile 744
Leu 792
Cys 797
Met 766
Asn 842
Arg 841
Leu 788
Ile 789
Lys 716

9d

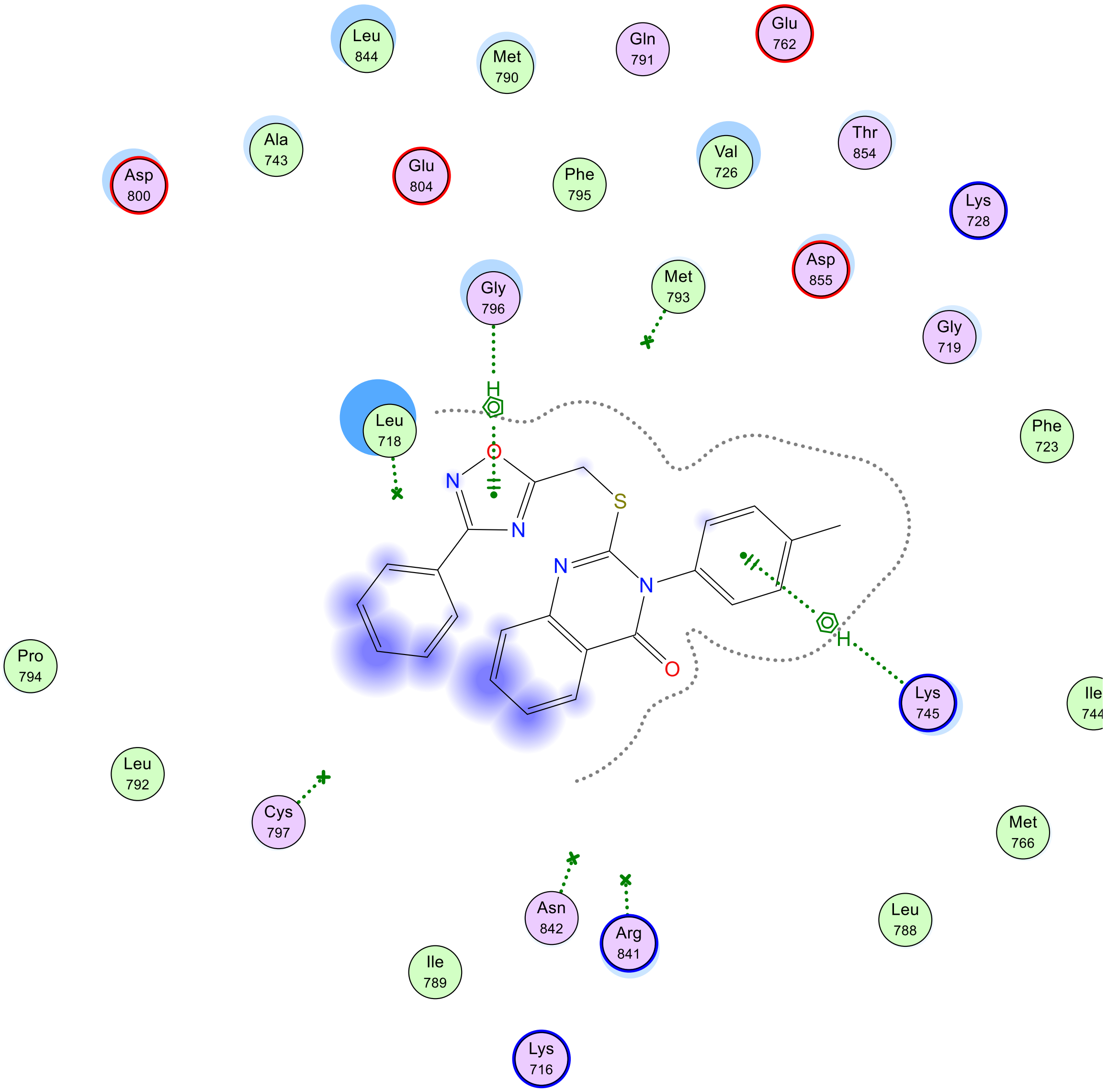
Leu 844
Met 790
Gln 791
Glu 762
Asp 800
Ala 743
Glu 804
Phe 795
Val 726
Thr 854
Lys 728
Gly 796
Met 793
Asp 855
Gly 719
Leu 718
Phe 723
H
O
N
N
S
N
N
O
H
Pro 794
Lys 745
Ile 744
Leu 792
Cys 797
Met 766
Asn 842
Arg 841
Leu 788
Ile 789
Lys 716

9e

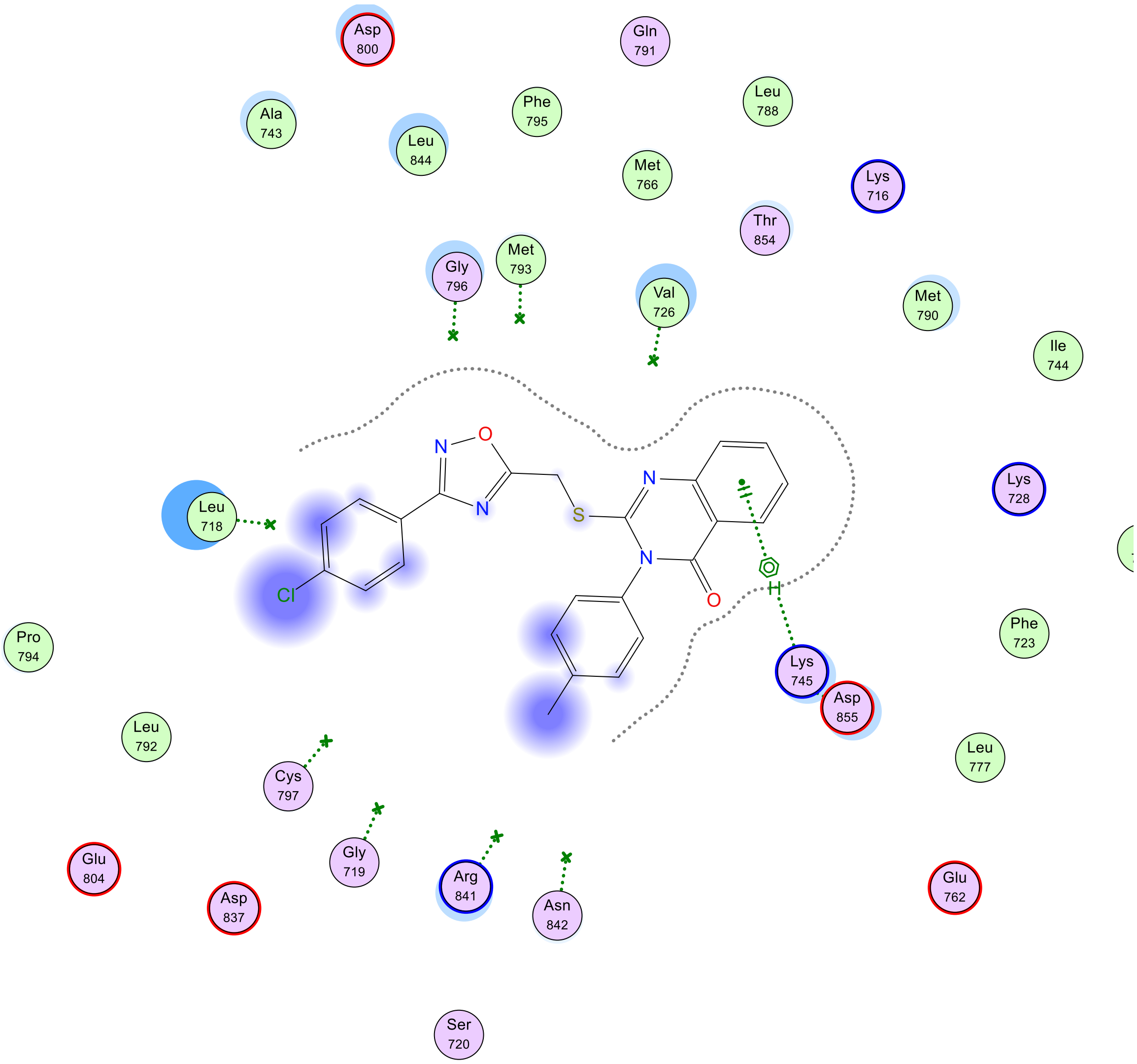
Asp 800
Gln 791
Ala 743
Phe 795
Leu 788
Leu 844
Met 766
Lys 716
Thr 854
Gly 796
Met 793
Val 726
Met 790
Ile 744
Lys 728
Leu 718
N
O
N
S
N
N
O
Cl
H
Phe 723
Pro 794
Lys 745
Asp 855
Leu 792
Leu 777
Cys 797
Gly 719
Arg 841
Asn 842
Glu 804
Asp 837
Glu 762
Ser 720

9f

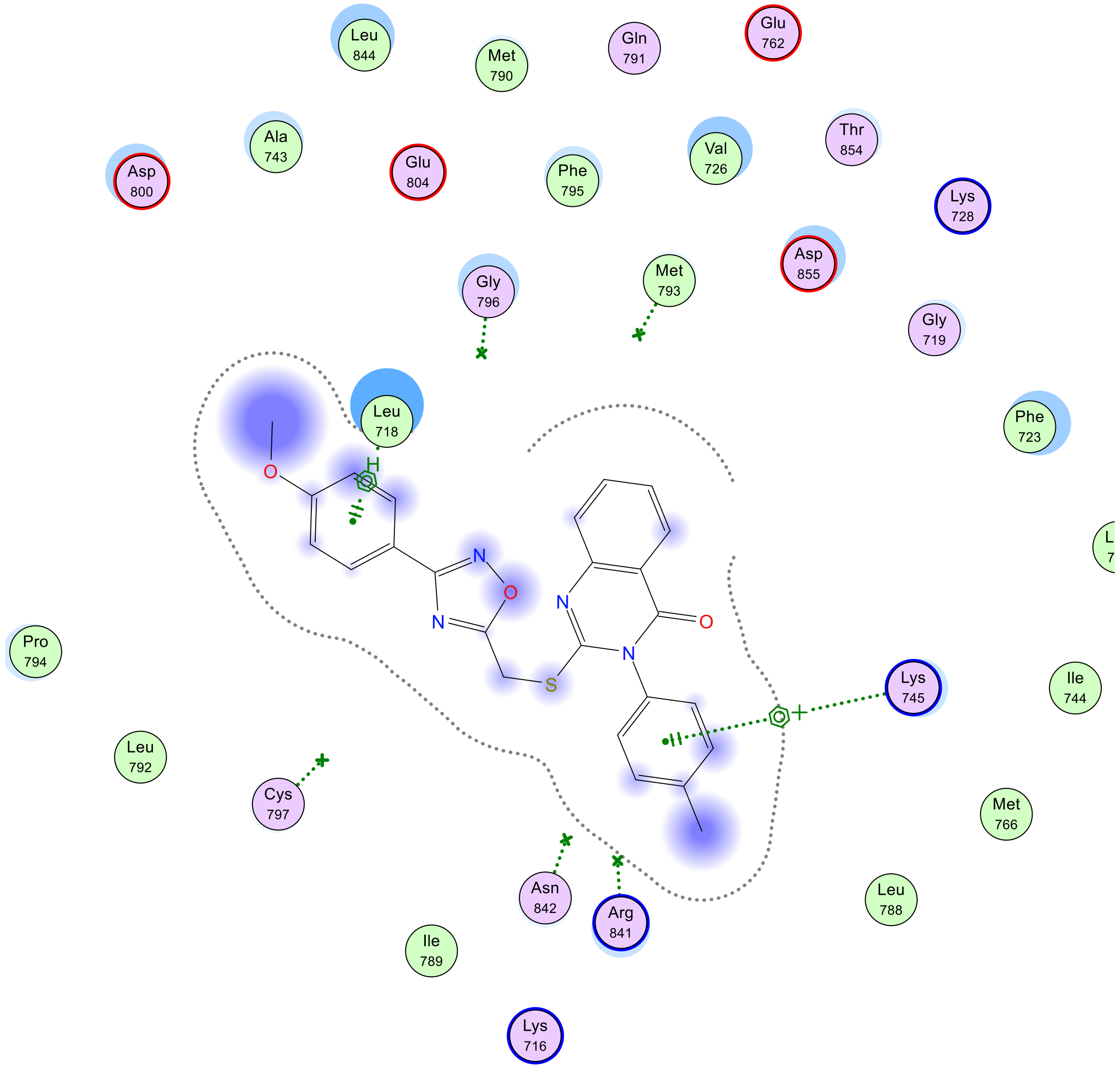

Leu 844
Met 790
Gln 791
Glu 762
Asp 800
Ala 743
Glu 804
Phe 795
Val 726
Thr 854
Lys 728
Gly 796
Met 793
Asp 855
Gly 719
Leu 718
Phe 723
O
H
N
O
N
N
N
O
S
Pro 794
Lys 745
Ile 744
Leu 792
Cys 797
Met 766
Asn 842
Arg 841
Leu 788
Ile 789
Lys 716

9g

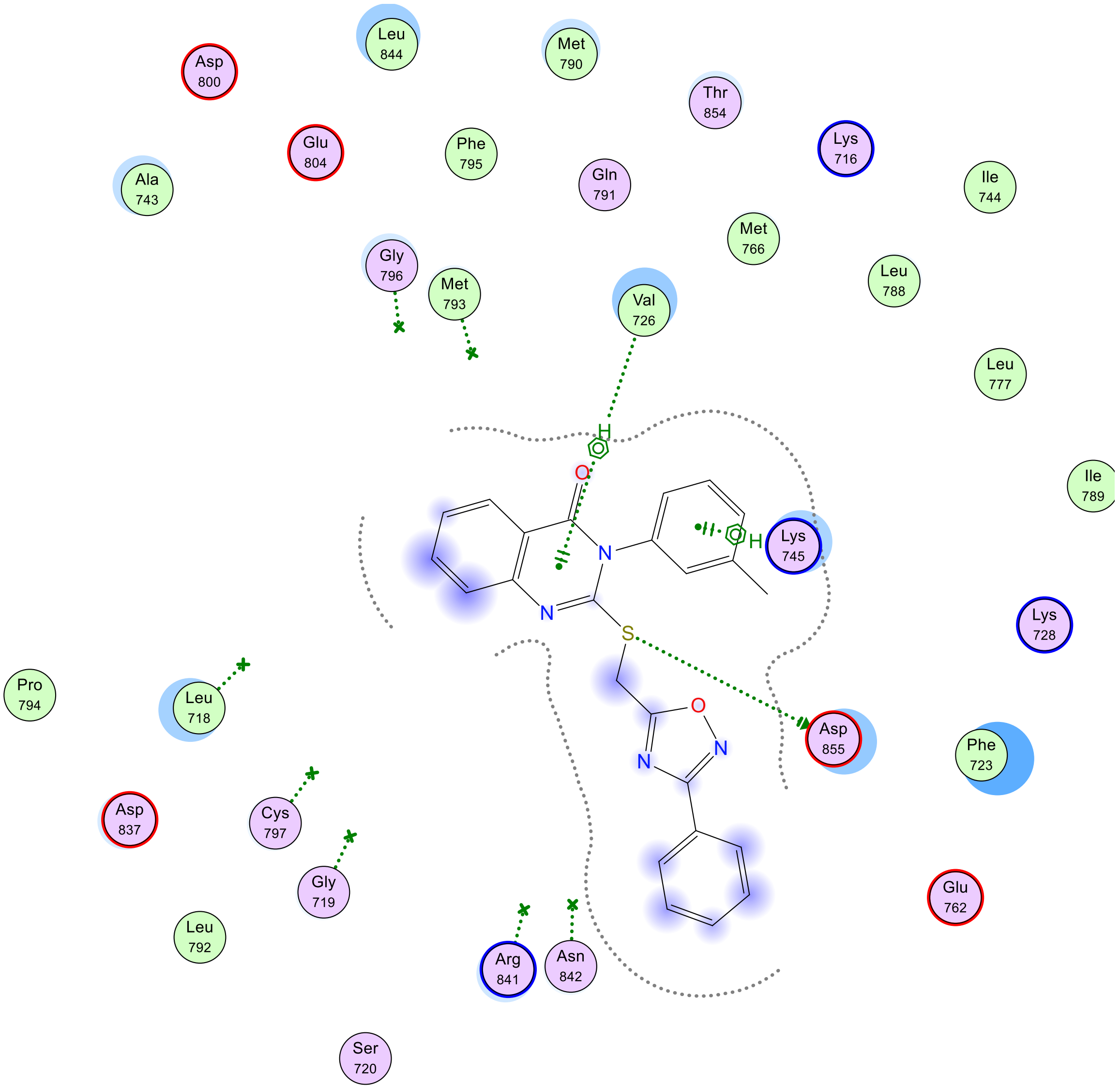
Asp 800
Leu 844
Met 790
Thr 854
Lys 716
Ile 744
Ala 743
Glu 804
Phe 795
Gln 791
Met 766
Leu 788
Gly 796
Met 793
Val 726
Leu 777
Ile 789
Lys 745
Lys 728
Pro 794
Leu 718
Asp 855
Phe 723
Asp 837
Cys 797
Gly 719
Glu 762
Leu 792
Arg 841
Asn 842
Ser 720

9h

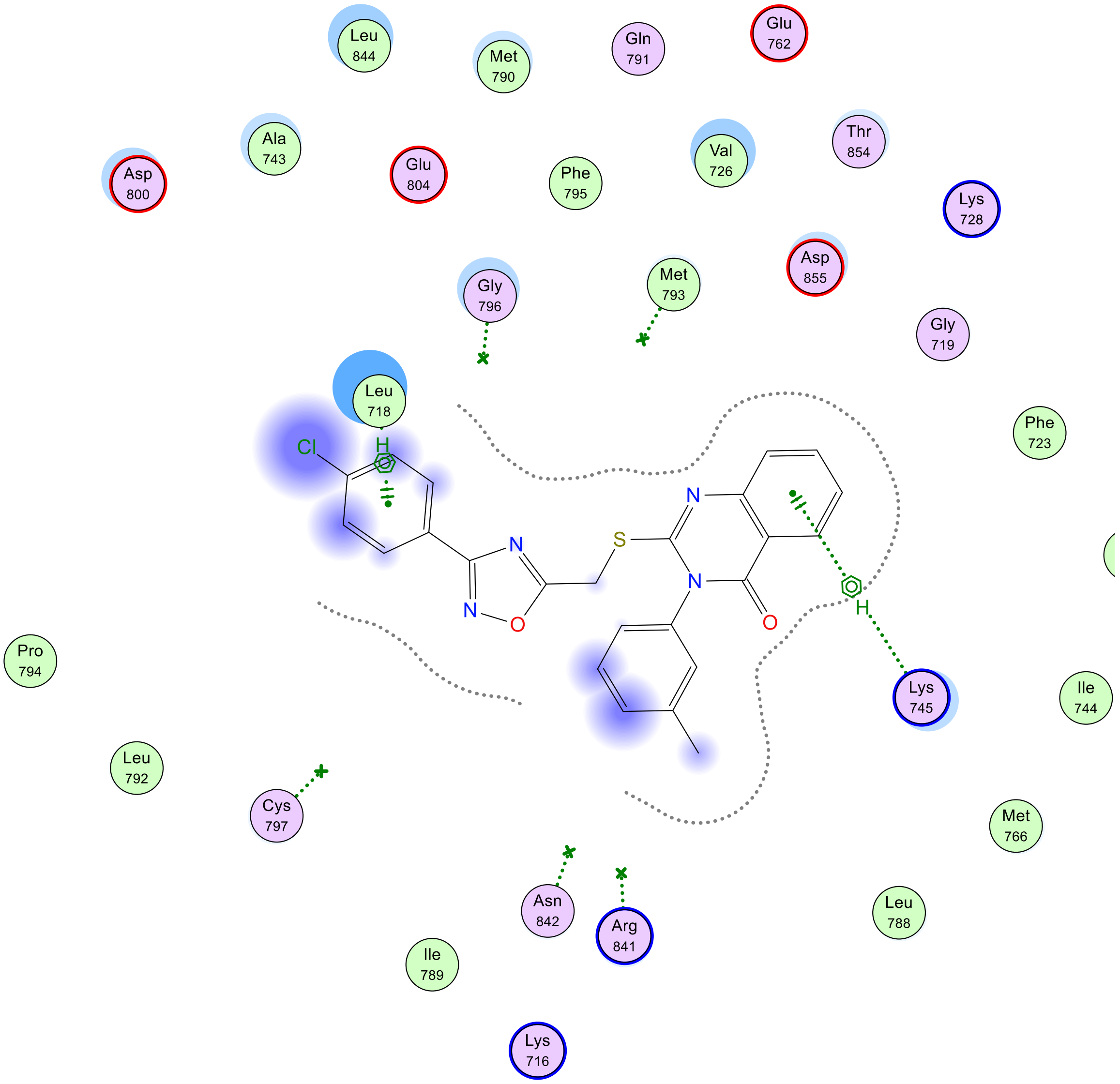

Leu 844
Met 790
Gln 791
Glu 762
Asp 800
Ala 743
Glu 804
Phe 795
Val 726
Thr 854
Lys 728
Gly 796
Met 793
Asp 855
Gly 719
Leu 718
Phe 723
Cl
H
N
S
N
O
N
N
O
H
Pro 794
Lys 745
Ile 744
Leu 792
Cys 797
Met 766
Asn 842
Arg 841
Leu 788
Ile 789
Lys 716

9i

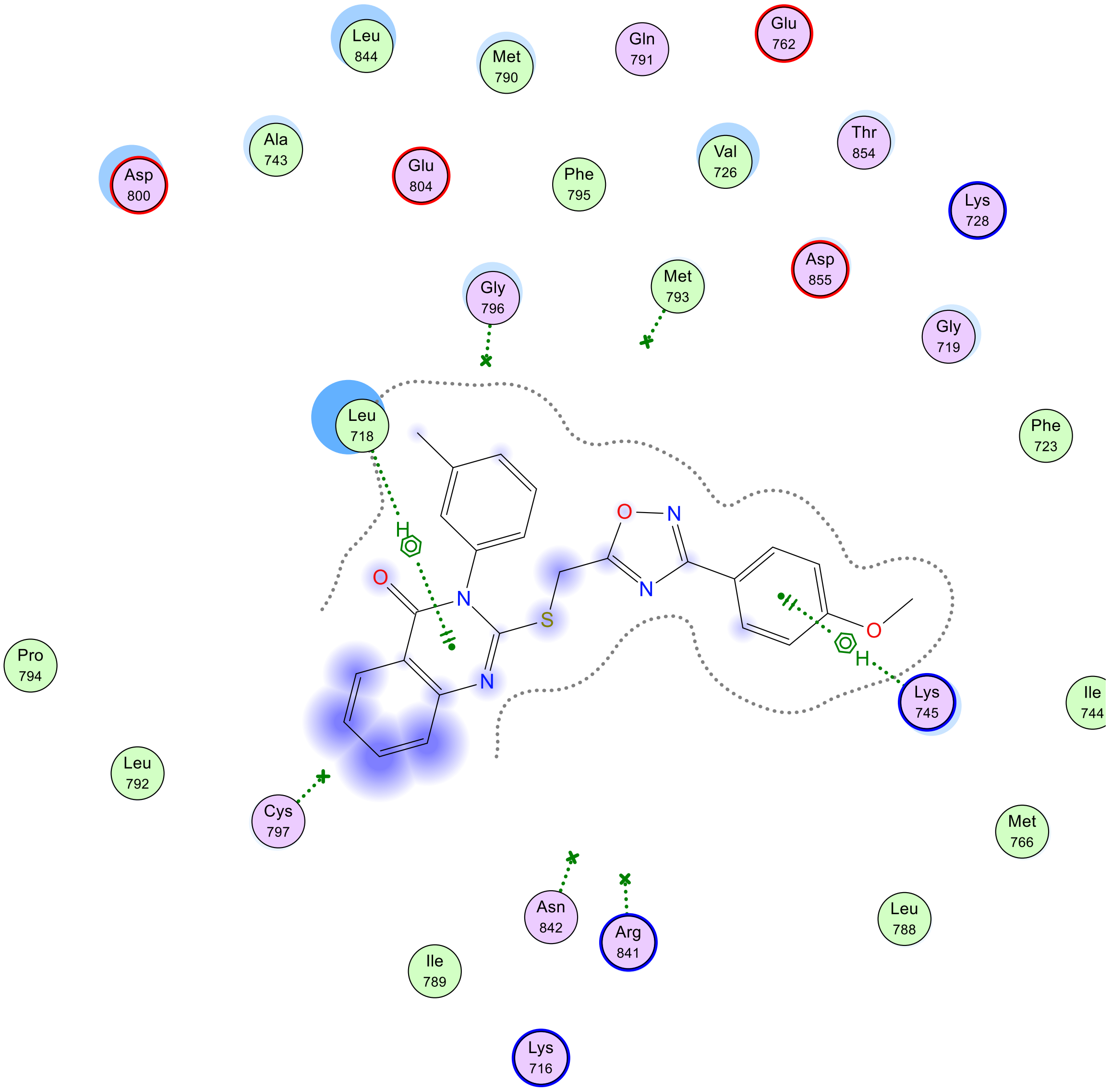
Leu 844
Met 790
Gln 791
Glu 762
Asp 800
Ala 743
Glu 804
Phe 795
Val 726
Thr 854
Lys 728
Gly 796
Met 793
Asp 855
Gly 719
Leu 718
Phe 723
O
N
N
O
N
S
N
O
H
H
Pro 794
Lys 745
Ile 744
Leu 792
Cys 797
Met 766
Asn 842
Arg 841
Leu 788
Ile 789
Lys 716

9j

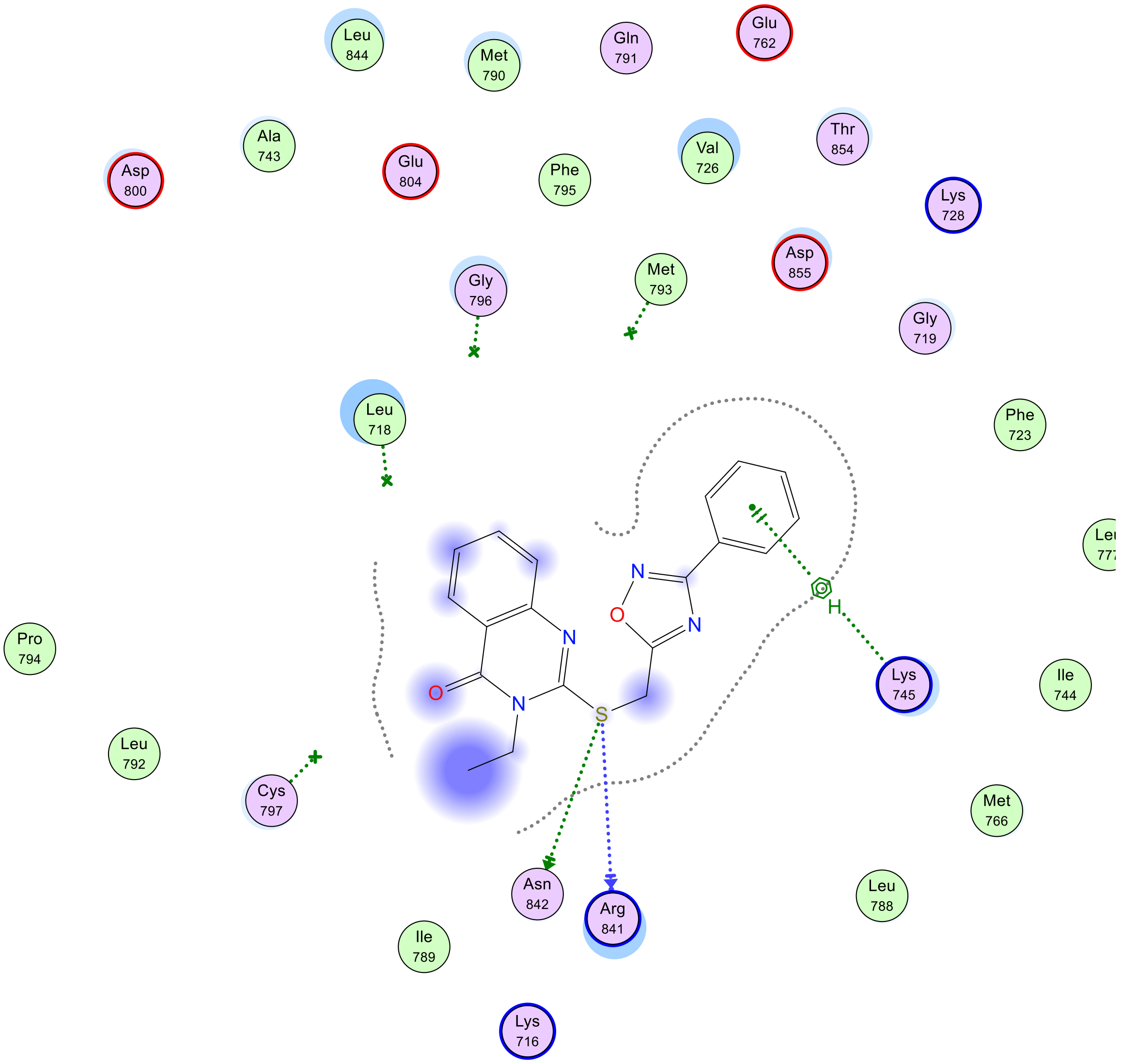
Leu 844
Met 790
Gln 791
Glu 762
Ala 743
Asp 800
Glu 804
Phe 795
Val 726
Thr 854
Lys 728
Gly 796
Met 793
Asp 855
Gly 719
Leu 718
Phe 723
N
O
N
N
O
N
S
H
Pro 794
Lys 745
Ile 744
Leu 792
Cys 797
Met 766
Asn 842
Arg 841
Leu 788
Ile 789
Lys 716

9k

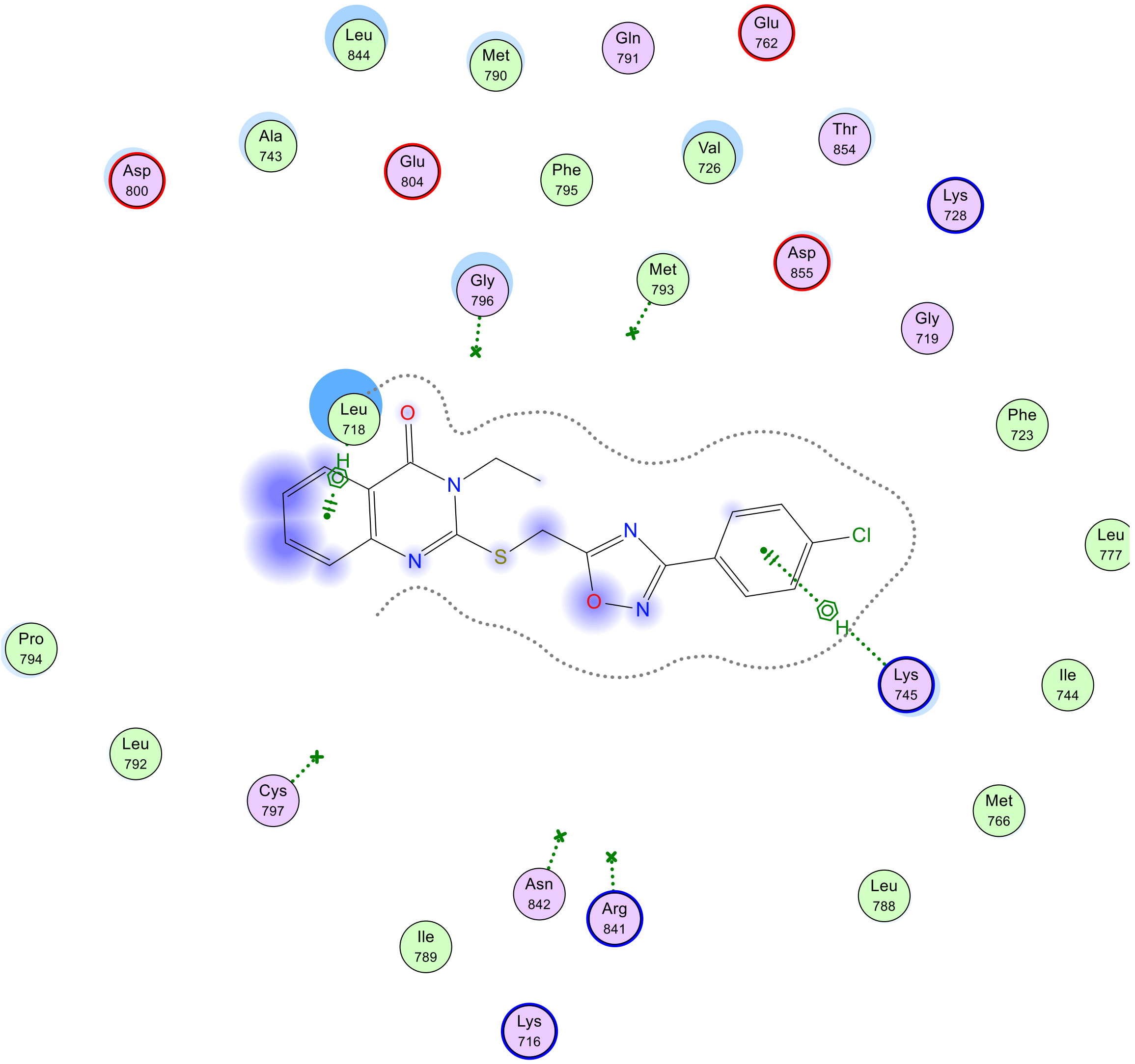
Leu 844
Met 790
Gln 791
Glu 762
Ala 743
Glu 804
Phe 795
Val 726
Thr 854
Asp 800
Lys 728
Asp 855
Gly 796
Met 793
Gly 719
Leu 718
O
H
N
Phe 723
N
N
Cl
Leu 777
S
O
N
H
Pro 794
Lys 745
Ile 744
Leu 792
Cys 797
Met 766
Asn 842
Arg 841
Leu 788
Ile 789
Lys 716

9l

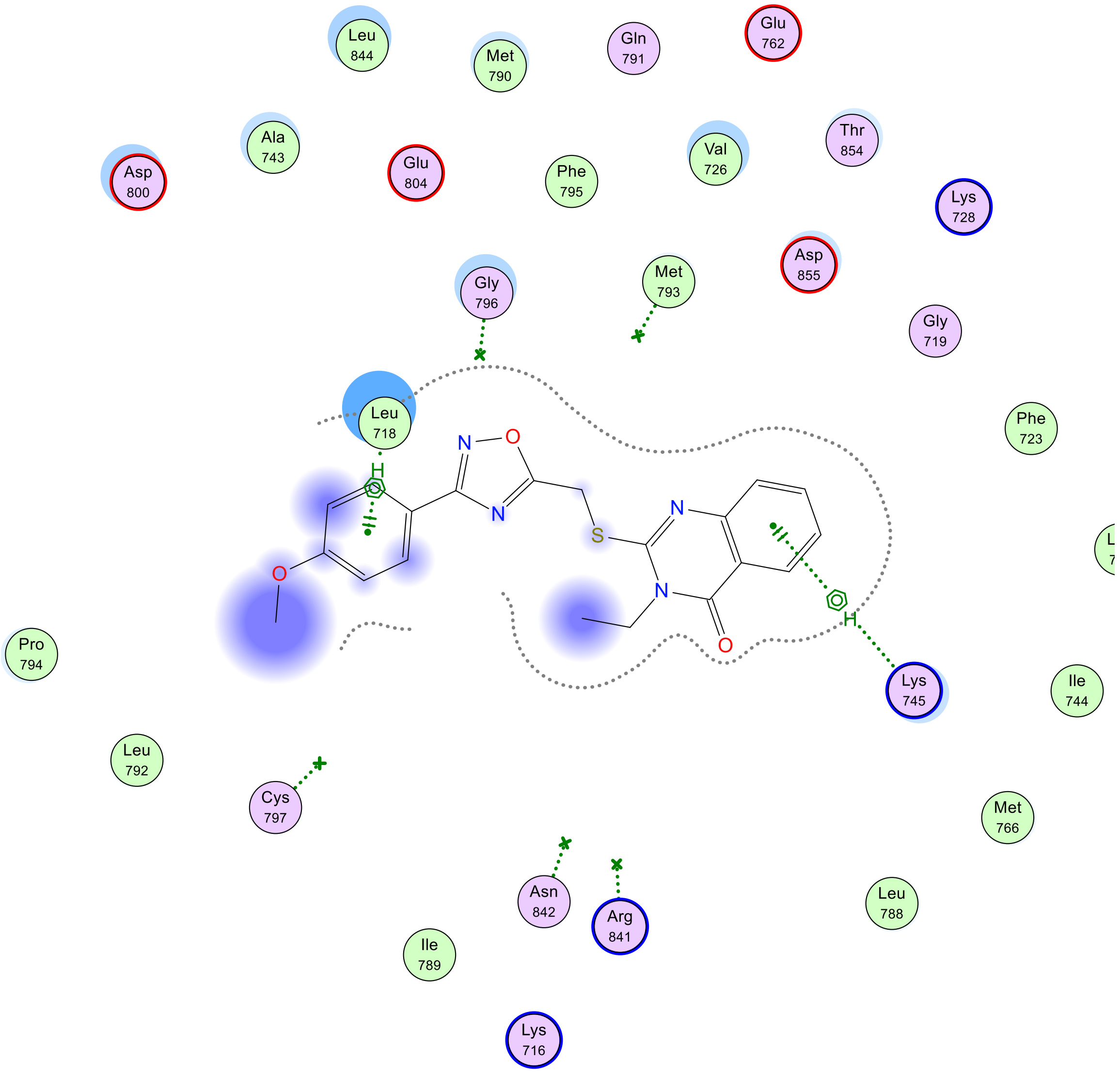

Leu 844
Met 790
Gln 791
Glu 762
Asp 800
Ala 743
Glu 804
Phe 795
Val 726
Thr 854
Lys 728
Asp 855
Gly 796
Met 793
Gly 719
Leu 718
Phe 723
N
O
N
S
N
N
O
O
H
H
Pro 794
Lys 745
Ile 744
Leu 792
Cys 797
Met 766
Asn 842
Arg 841
Leu 788
Ile 789
Lys 716

9m

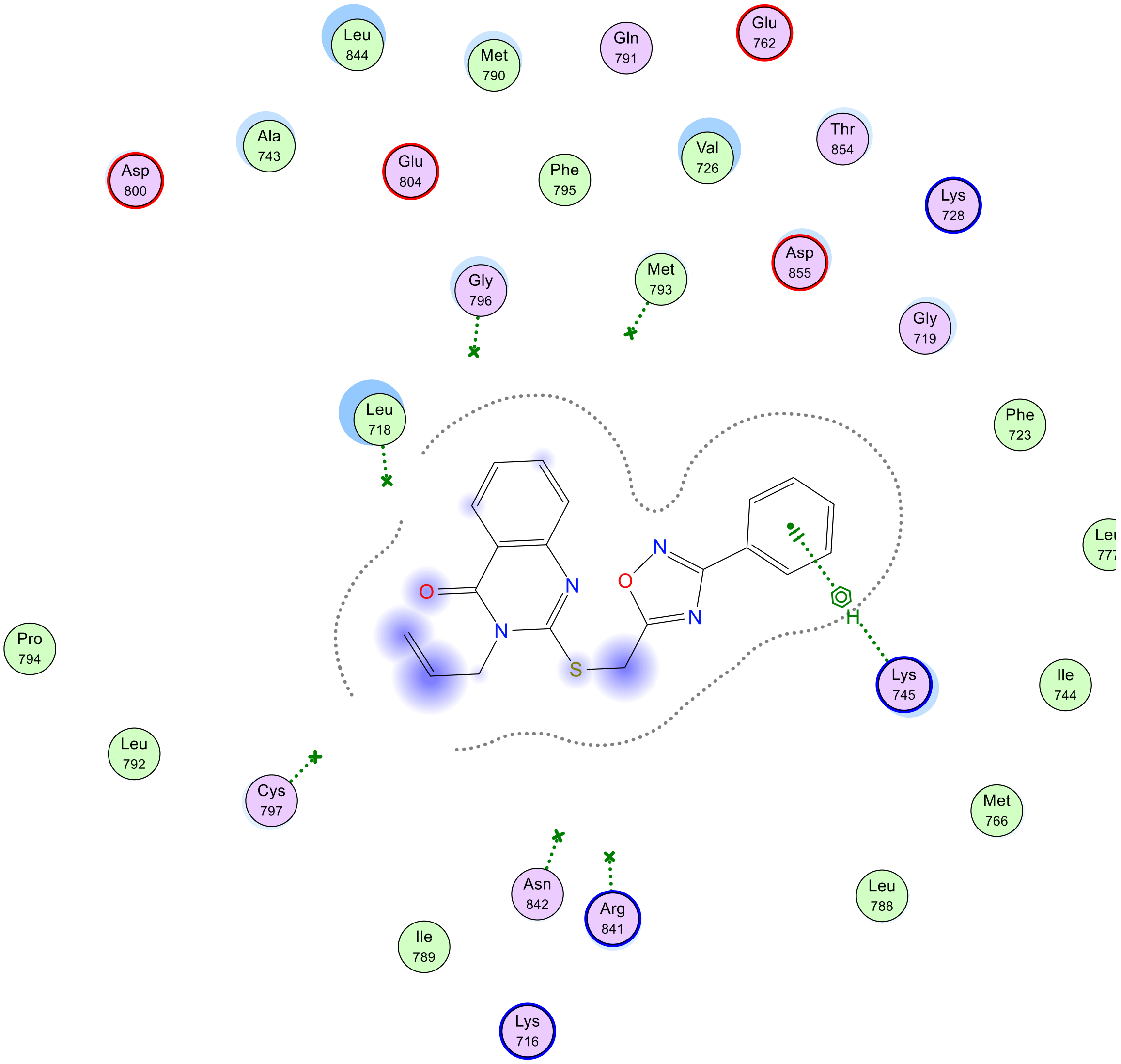

Leu 844
Met 790
Gln 791
Glu 762
Ala 743
Asp 800
Glu 804
Phe 795
Val 726
Thr 854
Lys 728
Gly 796
Met 793
Asp 855
Gly 719
Leu 718
Phe 723
O
N
N
O
N
N
S
H
Pro 794
Lys 745
Ile 744
Leu 792
Cys 797
Met 766
Asn 842
Arg 841
Leu 788
Ile 789
Lys 716

9n

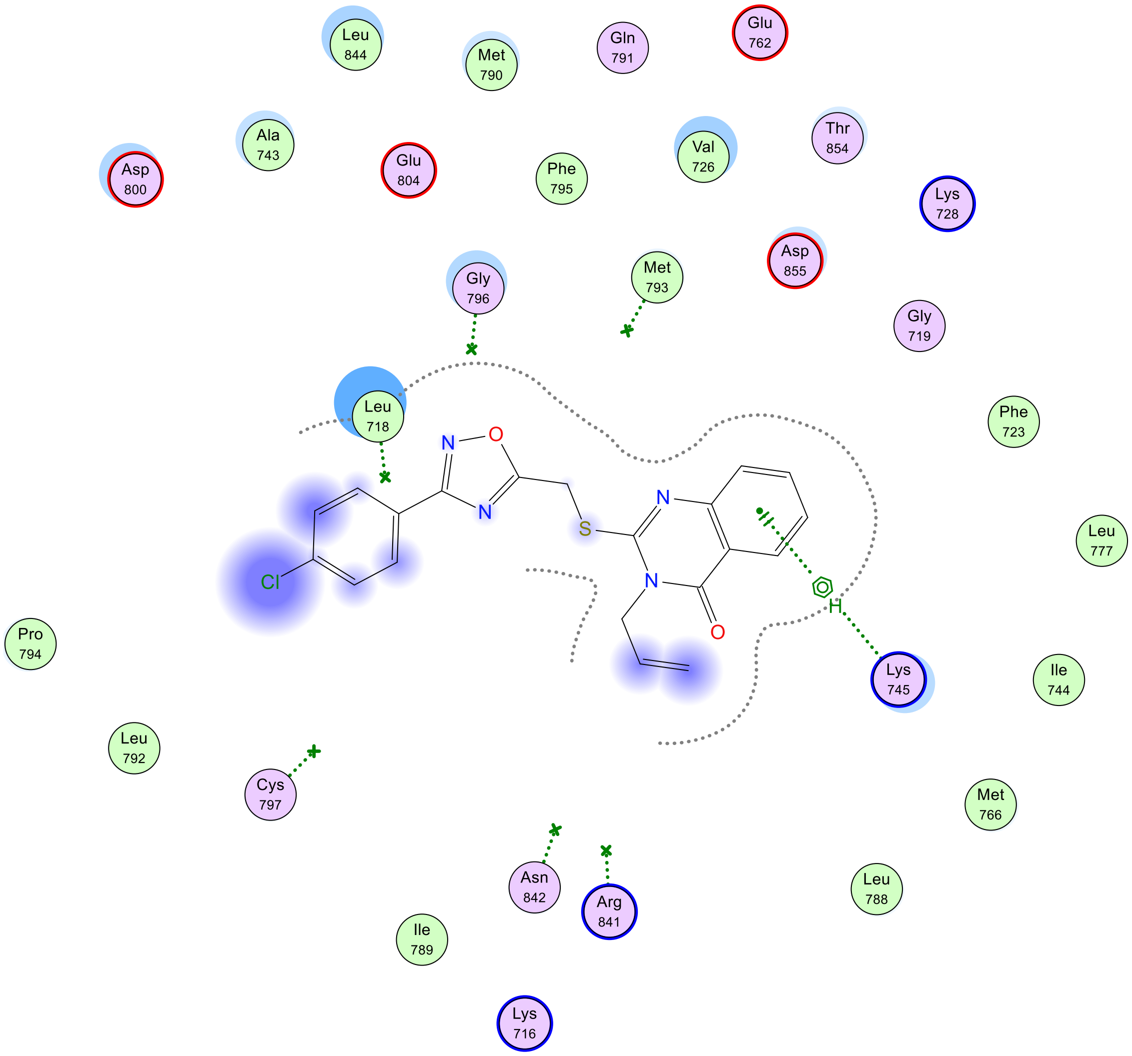

Leu 844
Met 790
Gln 791
Glu 762
Asp 800
Ala 743
Glu 804
Phe 795
Val 726
Thr 854
Lys 728
Asp 855
Gly 796
Met 793
Gly 719
Leu 718
Phe 723
N
O
N
S
N
N
O
Cl
H
Leu 777
Pro 794
Lys 745
Ile 744
Leu 792
Cys 797
Met 766
Asn 842
Arg 841
Leu 788
Ile 789
Lys 716

9o

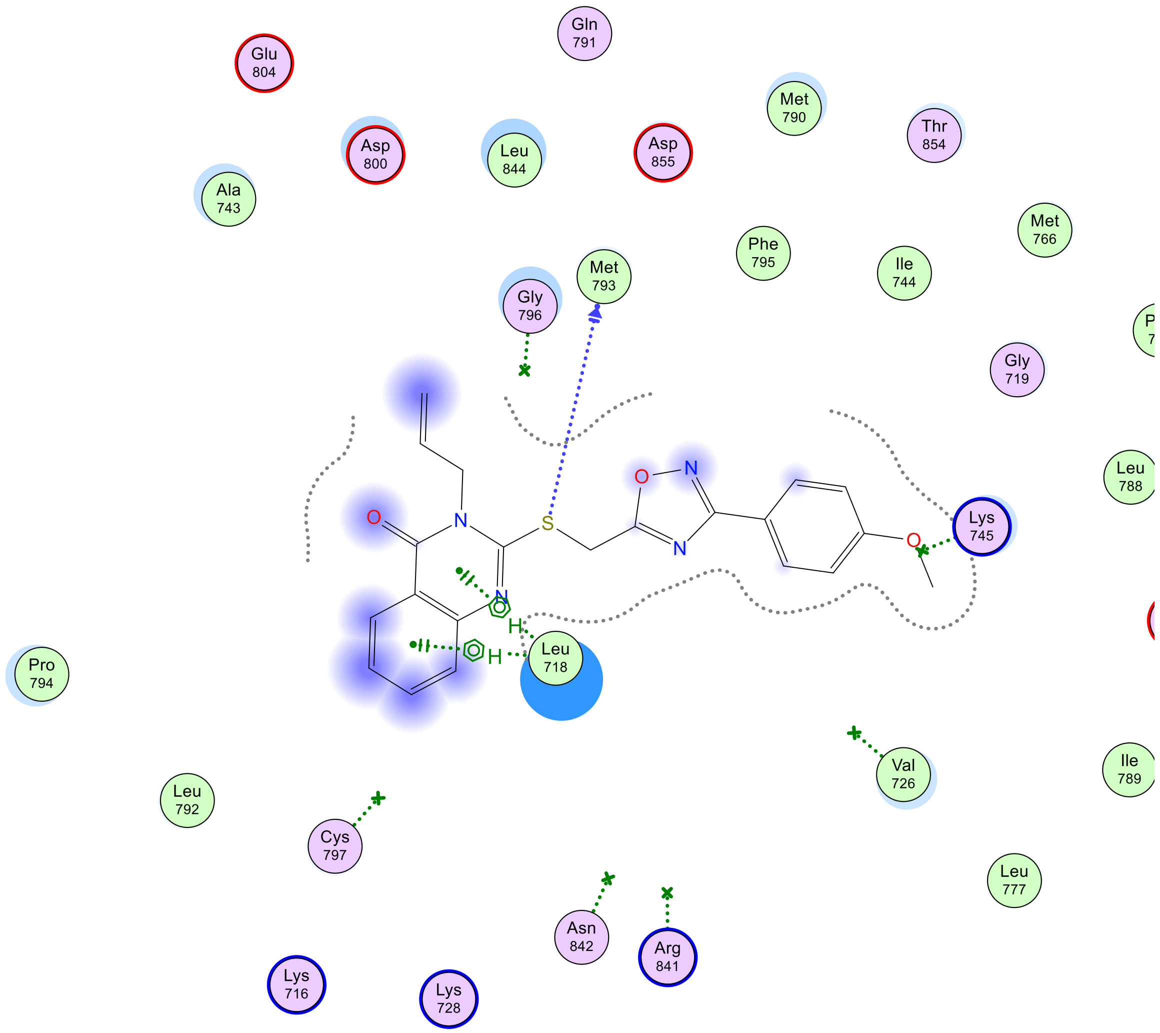
Gln 791
Glu 804
Met 790
Thr 854
Asp 800
Leu 844
Asp 855
Ala 743
Met 766
Phe 795
Ile 744
Met 793
Gly 796
Gly 719
Leu 788
Lys 745
Leu 718
Pro 794
Val 726
Ile 789
Leu 792
Cys 797
Leu 777
Asn 842
Arg 841
Lys 716
Lys 728

Osimertini b

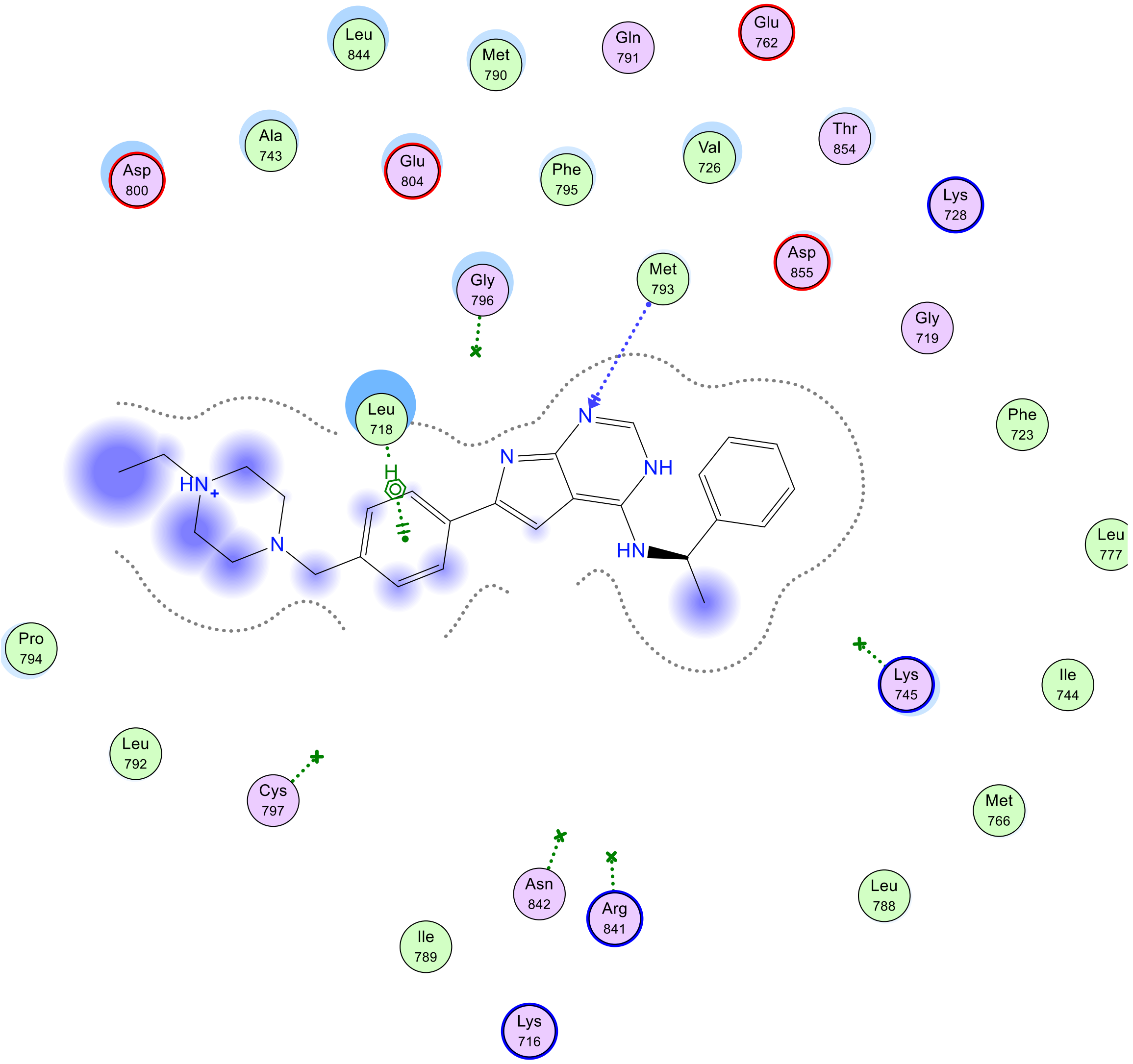

Fig. SI. 2 Docking poses of derivatives (**9a-o**) within EGFR^{T790M} (PDB ID: 2JIU) active site.

9a

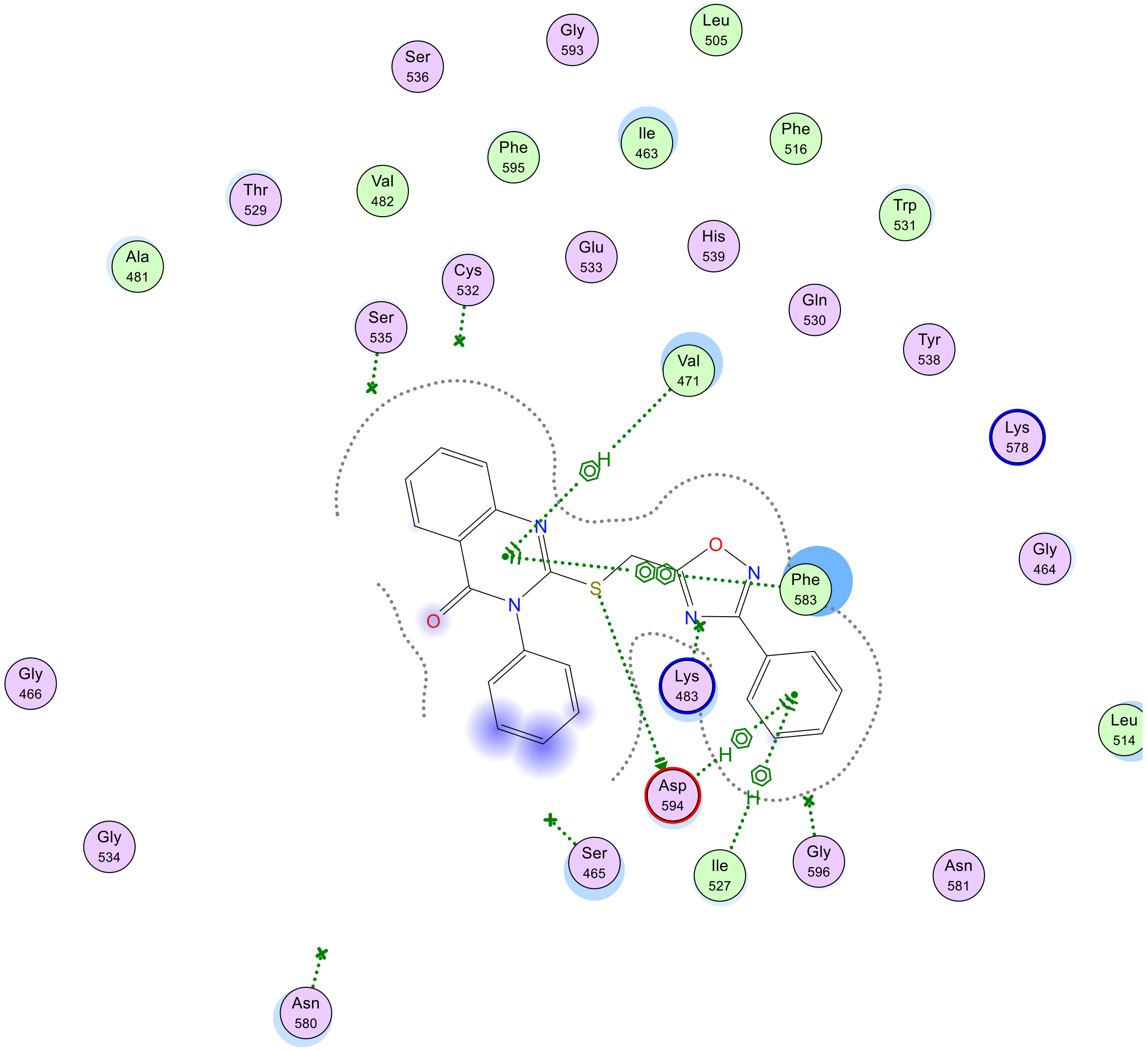
Ser 536
Gly 593
Leu 505
Phe 595
Ile 463
Phe 516
Thr 529
Val 482
Trp 531
Ala 481
Cys 532
Glu 533
His 539
Ser 535
Gln 530
Tyr 538
Val 471
Lys 578
Gly 464
Phe 583
Gly 466
Lys 483
Leu 514
Asp 594
Gly 534
Ser 465
Ile 527
Gly 596
Asn 581
Asn 580

9b

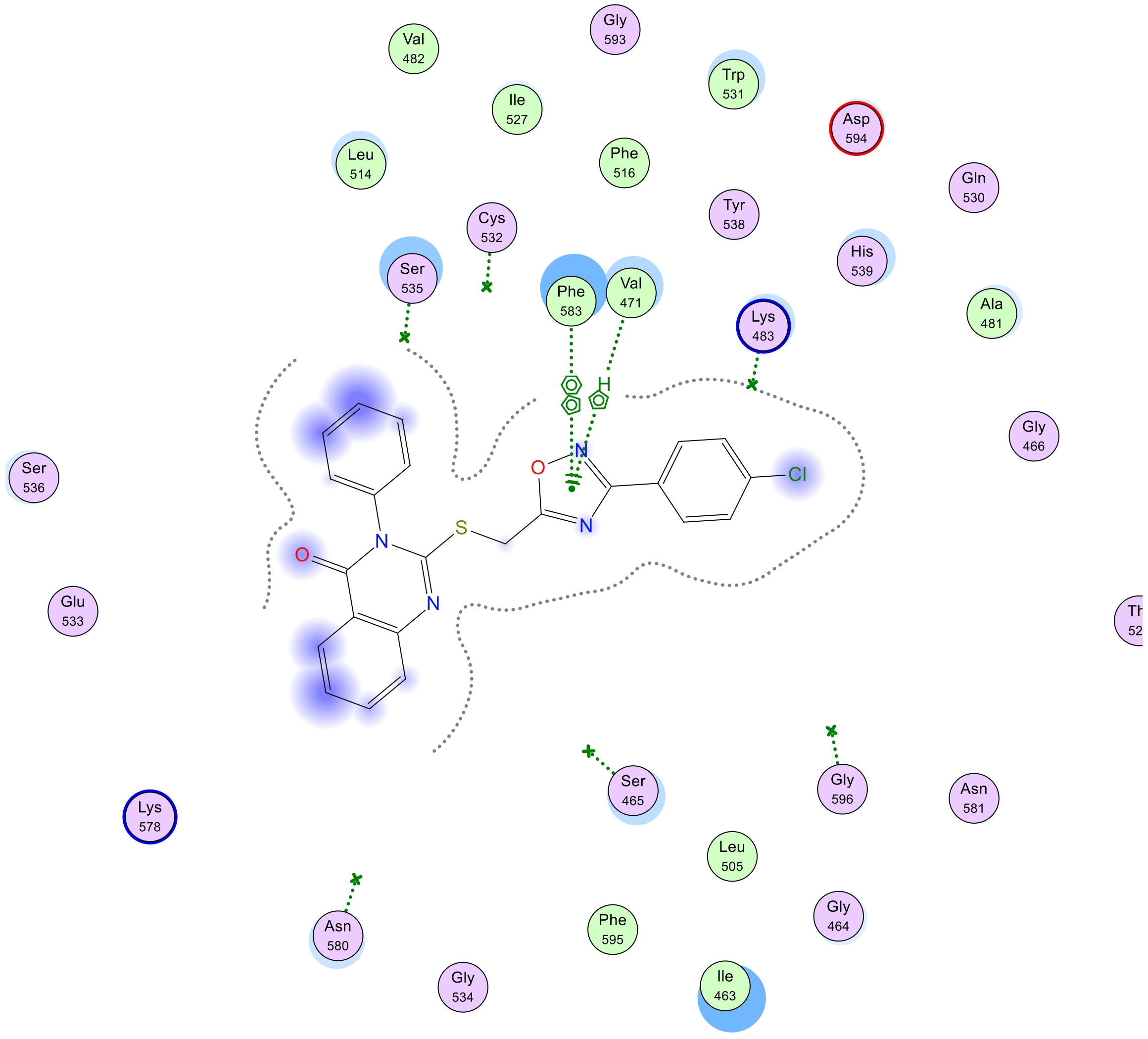

Val 482
Gly 593
Trp 531
Ile 527
Asp 594
Leu 514
Phe 516
Gln 530
Cys 532
Tyr 538
His 539
Ser 535
Phe 583
Val 471
Lys 483
Ala 481
Gly 466
Ser 536
Cl
O
N
S
Glu 533
Ser 465
Gly 596
Asn 581
Lys 578
Leu 505
Gly 464
Asn 580
Phe 595
Gly 534
Ile 463

9c

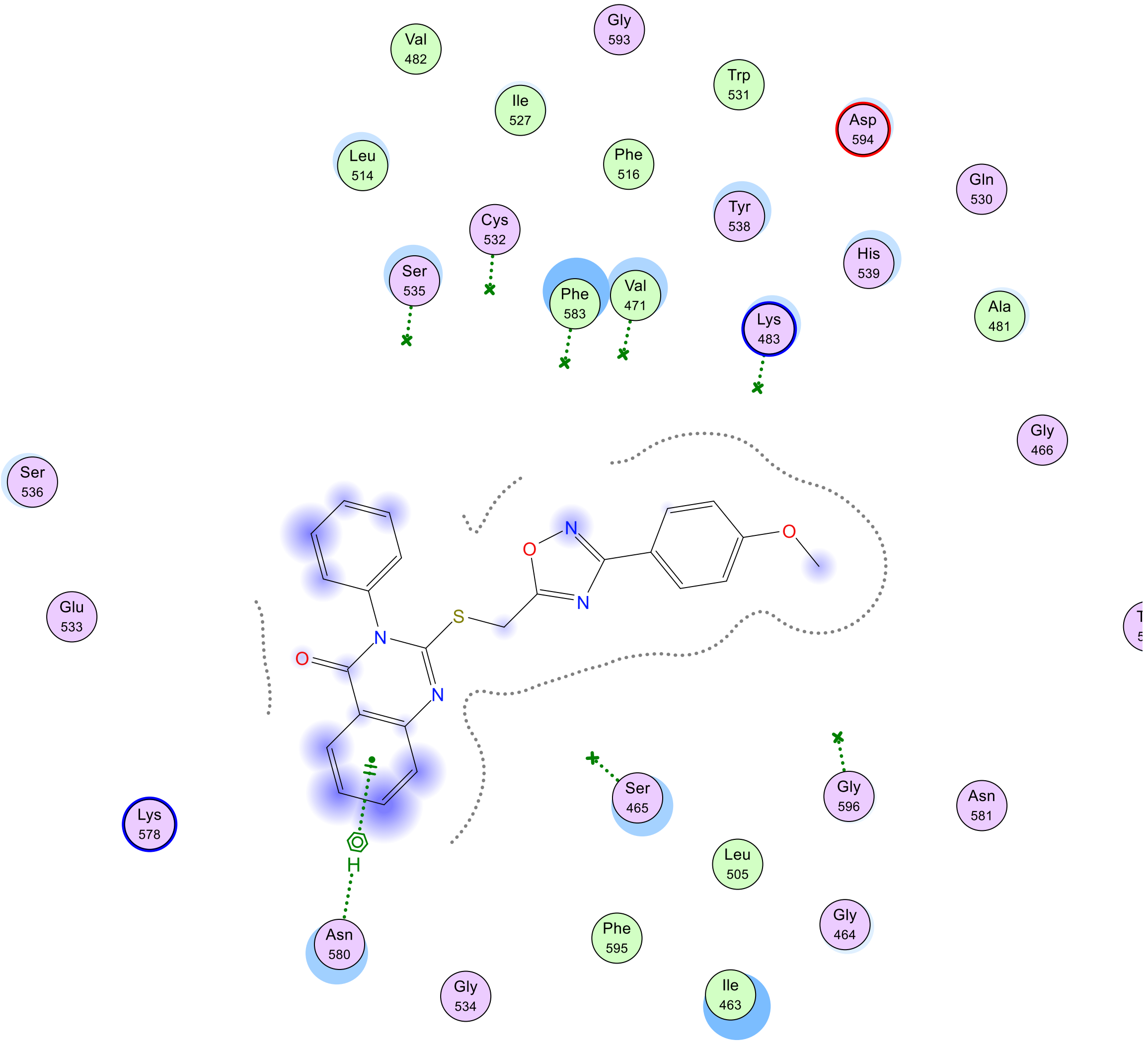

Val 482
Gly 593
Trp 531
Asp 594
Ile 527
Leu 514
Phe 516
Gln 530
Cys 532
Tyr 538
His 539
Ser 535
Phe 583
Val 471
Lys 483
Ala 481
Gly 466
Ser 536
Glu 533
O
N
S
N
O
N
N
O
H
Lys 578
Ser 465
Gly 596
Asn 581
Leu 505
Asn 580
Phe 595
Gly 464
Gly 534
Ile 463

9d

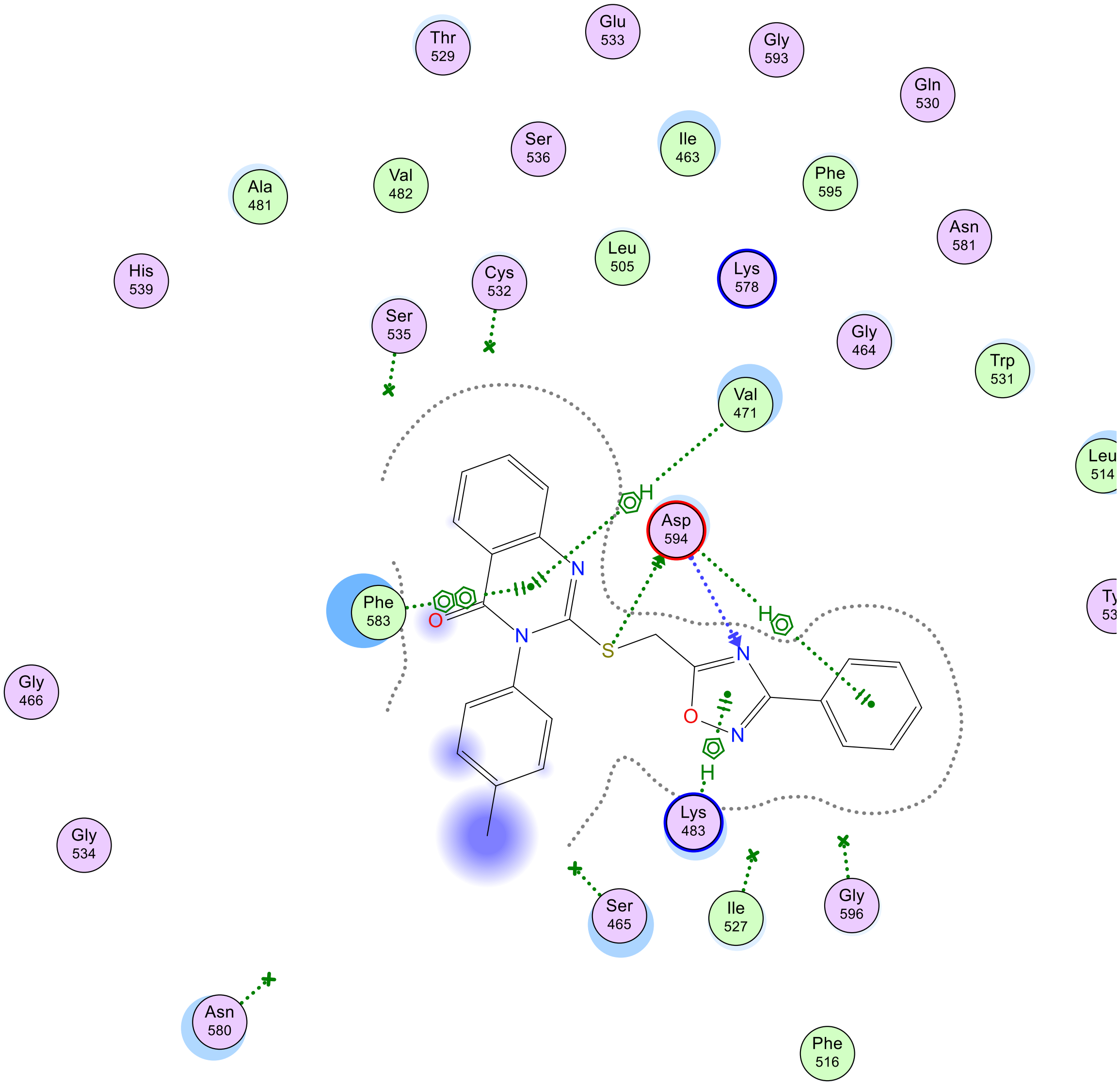

Thr 529
Glu 533
Gly 593
Gln 530
Ser 536
Ile 463
Phe 595
Ala 481
Val 482
Asn 581
His 539
Leu 505
Cys 532
Lys 578
Ser 535
Gly 464
Trp 531
Val 471
Leu 514
H
Asp 594
N
Phe 583
O
N
H
S
N
O
N
Gly 466
H
Lys 483
Gly 534
Ser 465
Ile 527
Gly 596
Asn 580
Phe 516

9e

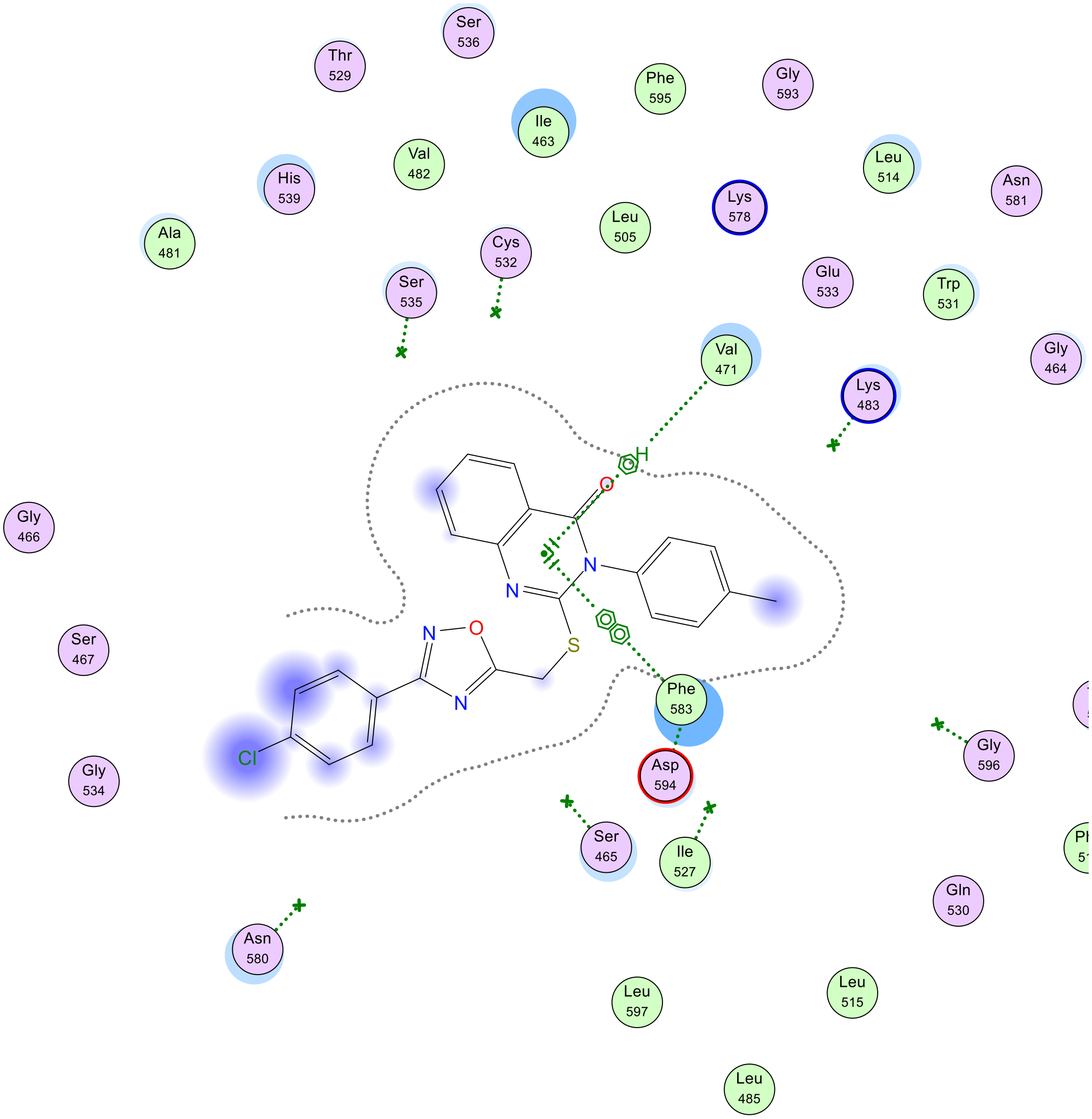

Ser 536
Thr 529
Phe 595
Gly 593
Ile 463
Val 482
His 539
Leu 514
Asn 581
Lys 578
Ala 481
Cys 532
Leu 505
Ser 535
Glu 533
Trp 531
Val 471
Gly 464
Lys 483
Gly 466
O
H
N
N
S
N
O
N
Ser 467
Cl
Phe 583
Gly 596
Asp 594
Gly 534
Ser 465
Ile 527
Gln 530
Asn 580
Leu 597
Leu 515
Leu 485

9f

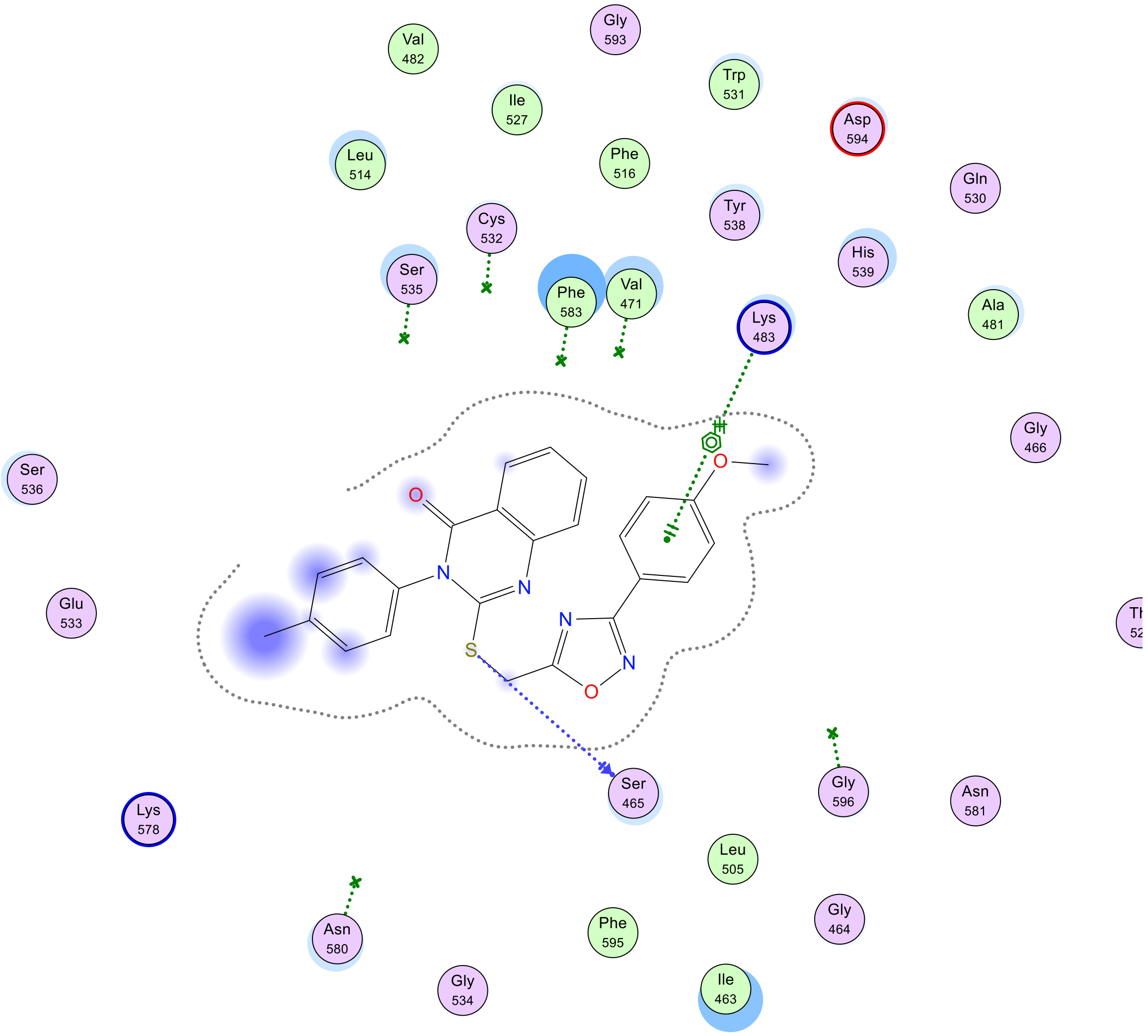

Val 482
Gly 593
Trp 531
Ile 527
Asp 594
Leu 514
Phe 516
Gln 530
Tyr 538
Cys 532
His 539
Ser 535
Phe 583
Val 471
Lys 483
Ala 481
Gly 466
Ser 536
O
O
N
N
Glu 533
S
N
N
O
Ser 465
Gly 596
Asn 581
Lys 578
Leu 505
Asn 580
Phe 595
Gly 464
Gly 534
Ile 463

9g

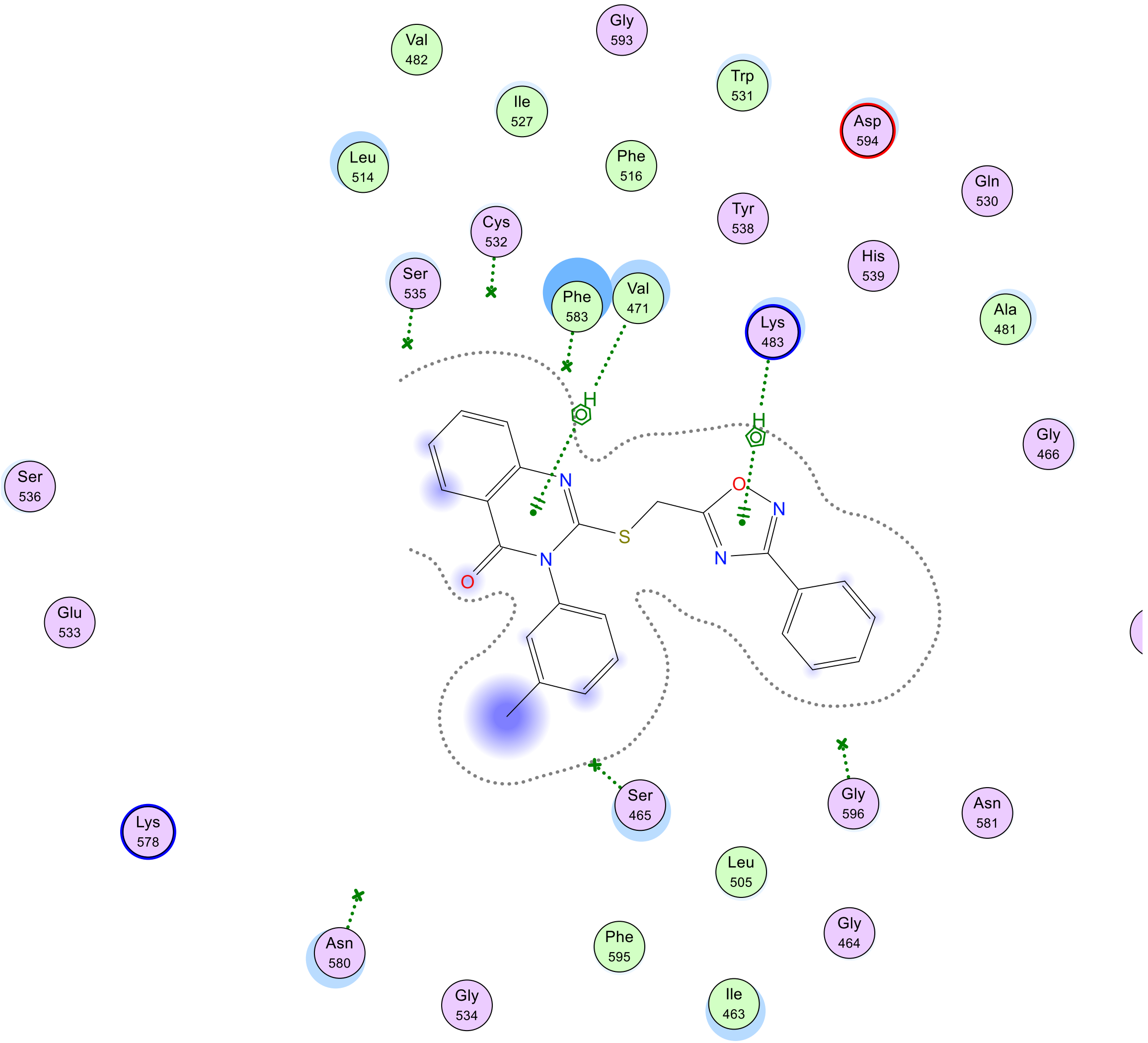

Gly 593
Val 482
Trp 531
Ile 527
Asp 594
Leu 514
Phe 516
Gln 530
Cys 532
Tyr 538
His 539
Ser 535
Phe 583
Val 471
Lys 483
Ala 481
Gly 466
Ser 536
Glu 533
Ser 465
Gly 596
Asn 581
Lys 578
Leu 505
Asn 580
Phe 595
Gly 464
Gly 534
Ile 463

9h

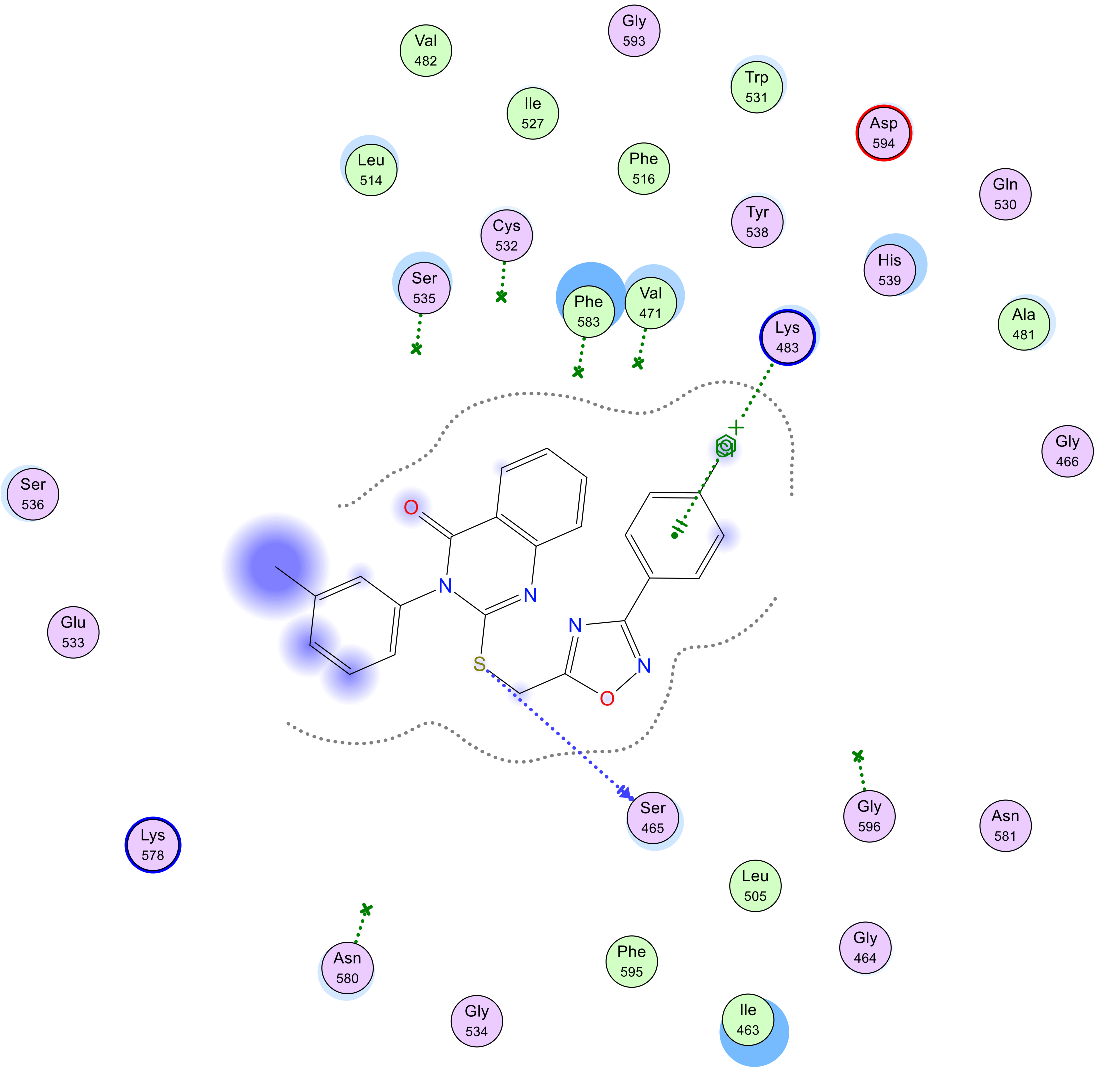

Val 482
Gly 593
Ile 527
Trp 531
Asp 594
Leu 514
Phe 516
Gln 530
Cys 532
Tyr 538
Ser 535
Phe 583
Val 471
His 539
Lys 483
Ala 481
Gly 466
Ser 536
O
N
N
N
N
S
O
Glu 533
Ser 465
Gly 596
Asn 581
Lys 578
Leu 505
Asn 580
Phe 595
Gly 464
Gly 534
Ile 463

9i

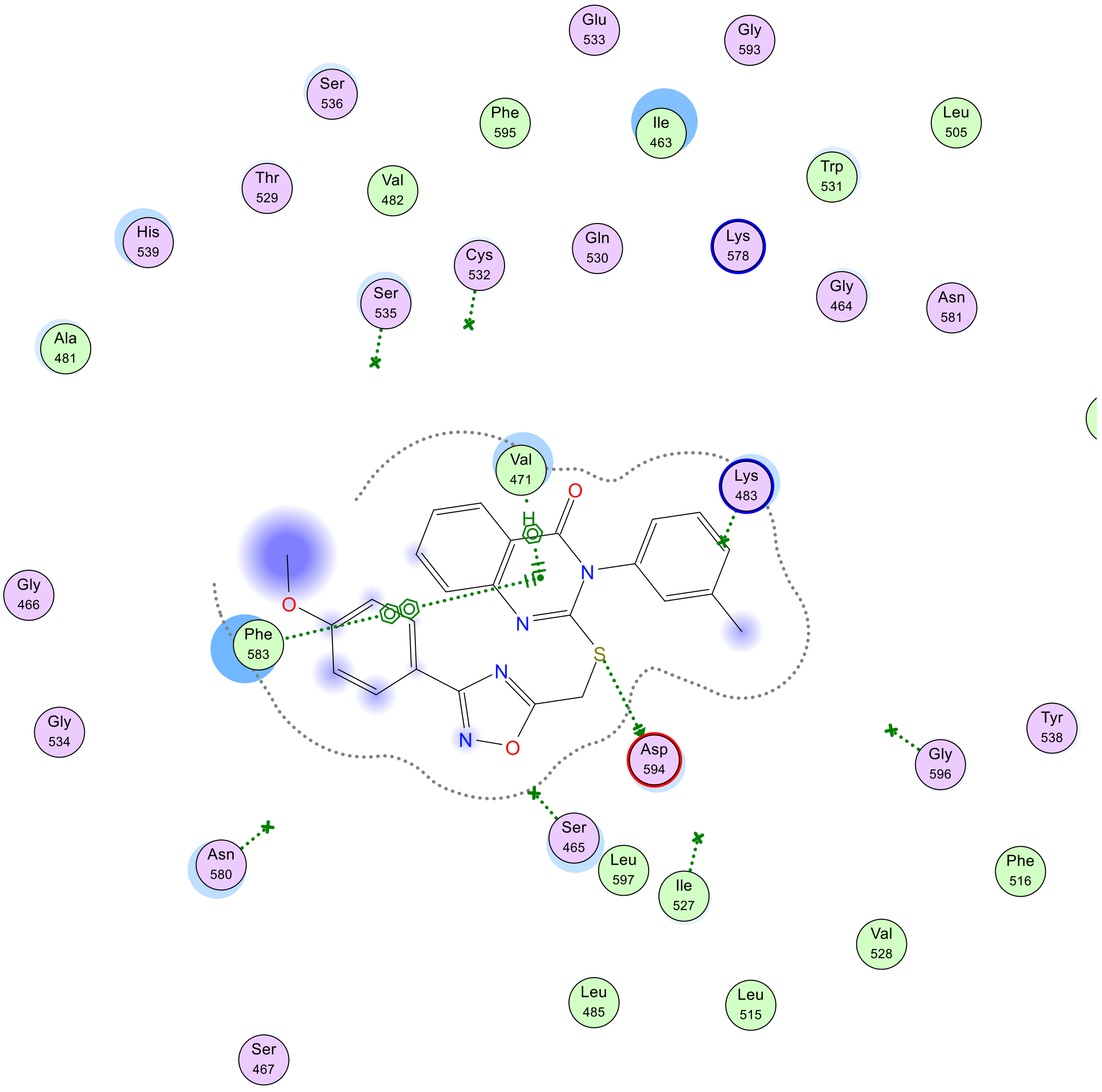

Glu 533
Gly 593
Ser 536
Phe 595
Ile 463
Leu 505
Thr 529
Val 482
Trp 531
His 539
Cys 532
Gln 530
Lys 578
Ser 535
Gly 464
Asn 581
Ala 481
Val 471
Lys 483
Gly 466
Phe 583
Gly 534
Asp 594
Tyr 538
Gly 596
Asn 580
Ser 465
Leu 597
Ile 527
Phe 516
Val 528
Leu 485
Leu 515
Ser 467

9j

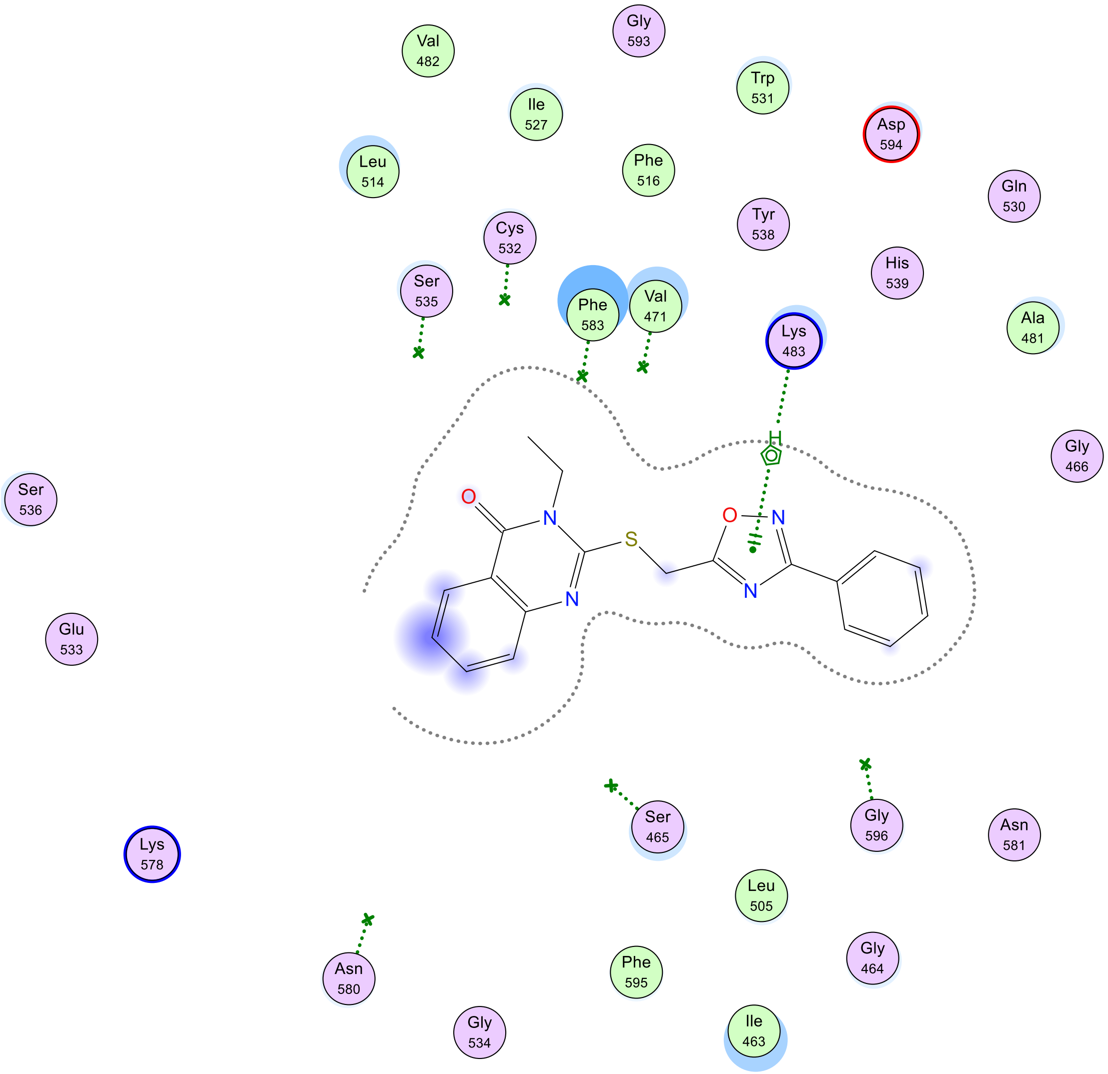

Val 482
Gly 593
Ile 527
Trp 531
Asp 594
Leu 514
Phe 516
Gln 530
Cys 532
Tyr 538
His 539
Ser 535
Phe 583
Val 471
Lys 483
Ala 481
Gly 466
Ser 536
O
N
S
O
N
N
N
Glu 533
Ser 465
Gly 596
Asn 581
Lys 578
Leu 505
Asn 580
Phe 595
Gly 464
Gly 534
Ile 463

9k

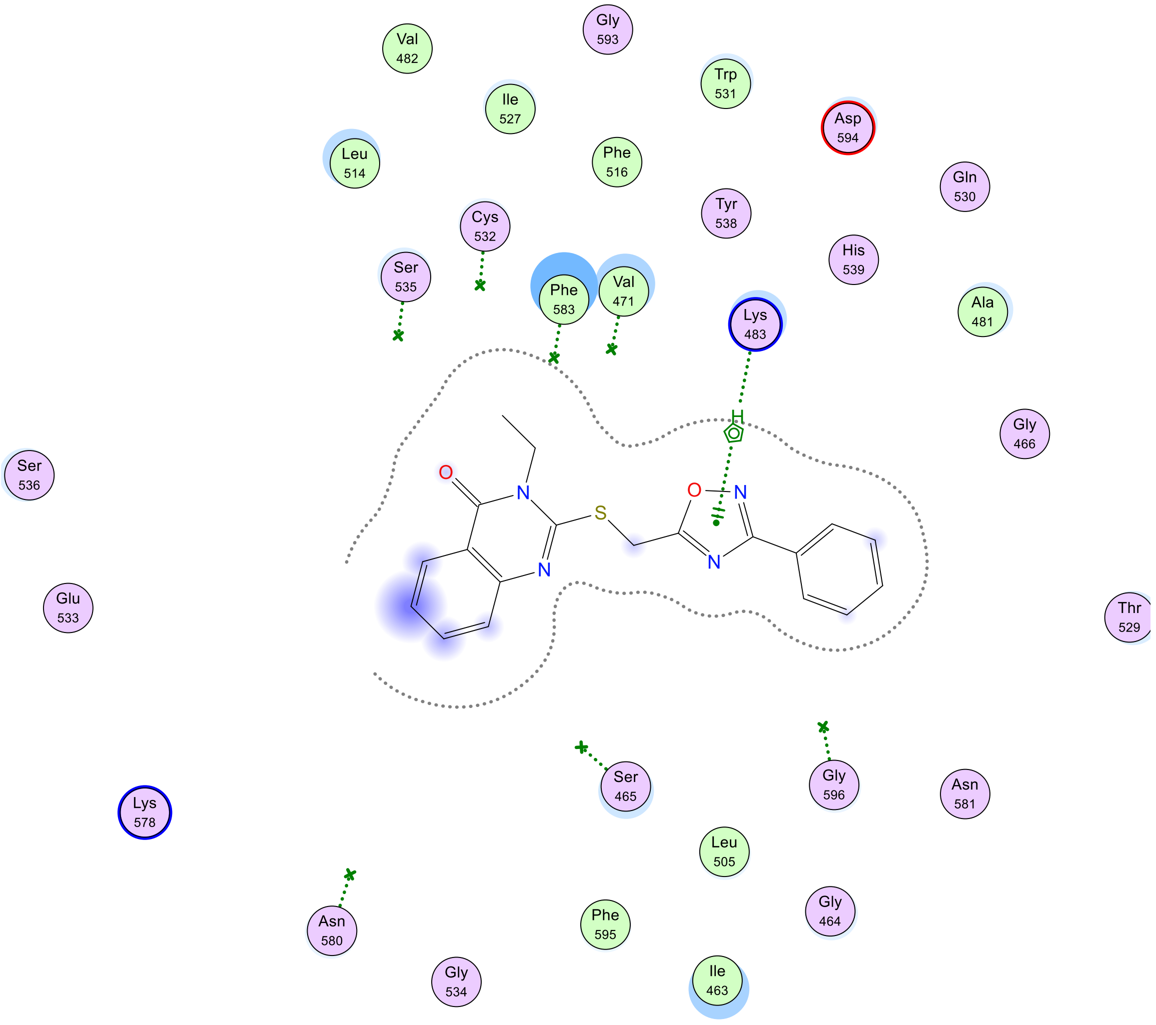
Val 482
Gly 593
Trp 531
Ile 527
Asp 594
Leu 514
Phe 516
Gln 530
Tyr 538
Cys 532
His 539
Ser 535
Phe 583
Val 471
Lys 483
Ala 481
Gly 466
Ser 536
O
N
S
N
O
N
N
H
Glu 533
Thr 529
Ser 465
Gly 596
Asn 581
Lys 578
Leu 505
Asn 580
Gly 464
Phe 595
Gly 534
Ile 463

9l

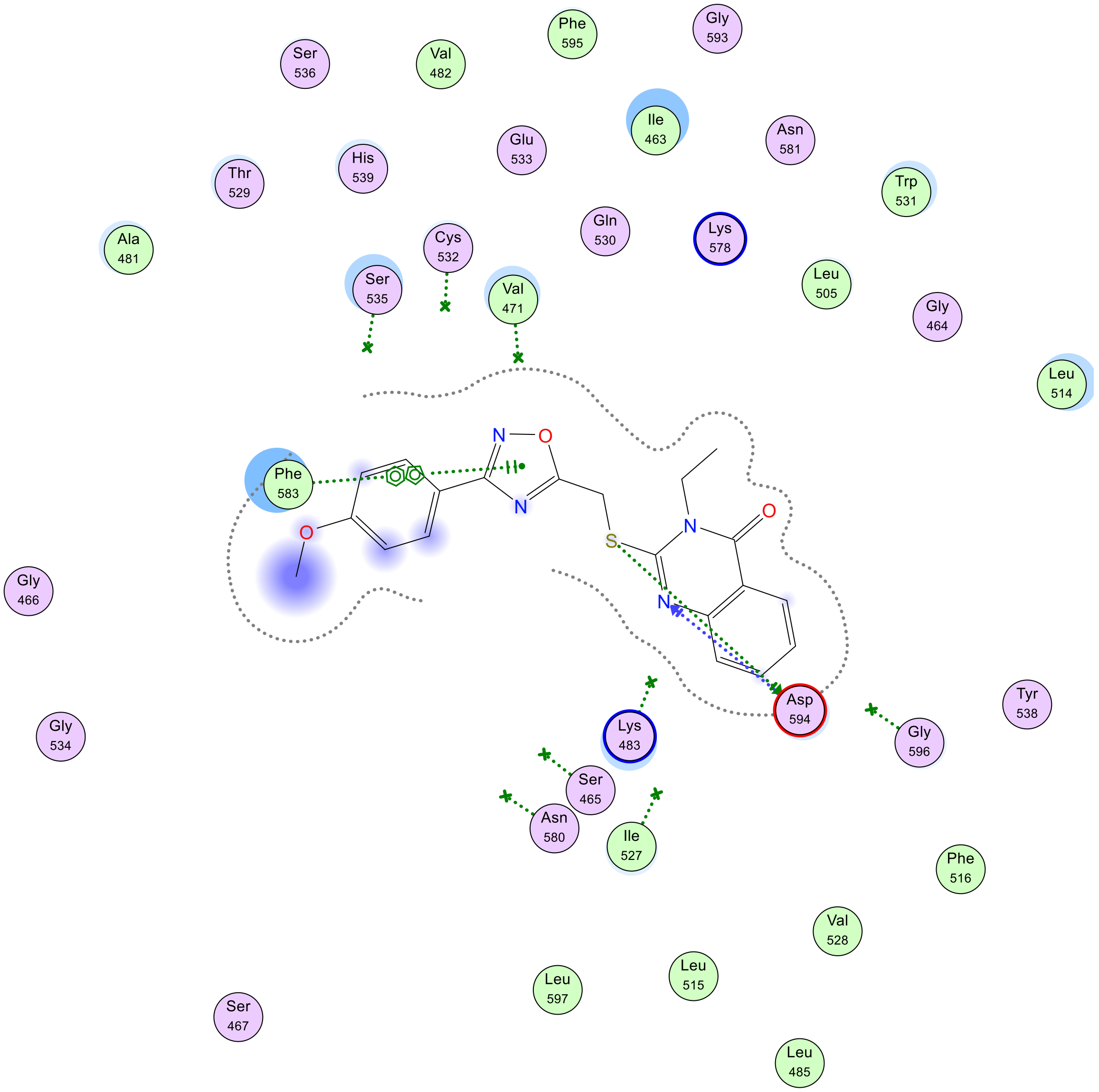
Ser 536
Val 482
Phe 595
Gly 593
Ile 463
Asn 581
Thr 529
His 539
Glu 533
Trp 531
Ala 481
Cys 532
Gln 530
Lys 578
Ser 535
Val 471
Leu 505
Gly 464
Leu 514
Phe 583
Gly 466
Asp 594
Tyr 538
Gly 534
Lys 483
Gly 596
Ser 465
Asn 580
Ile 527
Phe 516
Val 528
Leu 597
Leu 515
Ser 467
Leu 485

9m

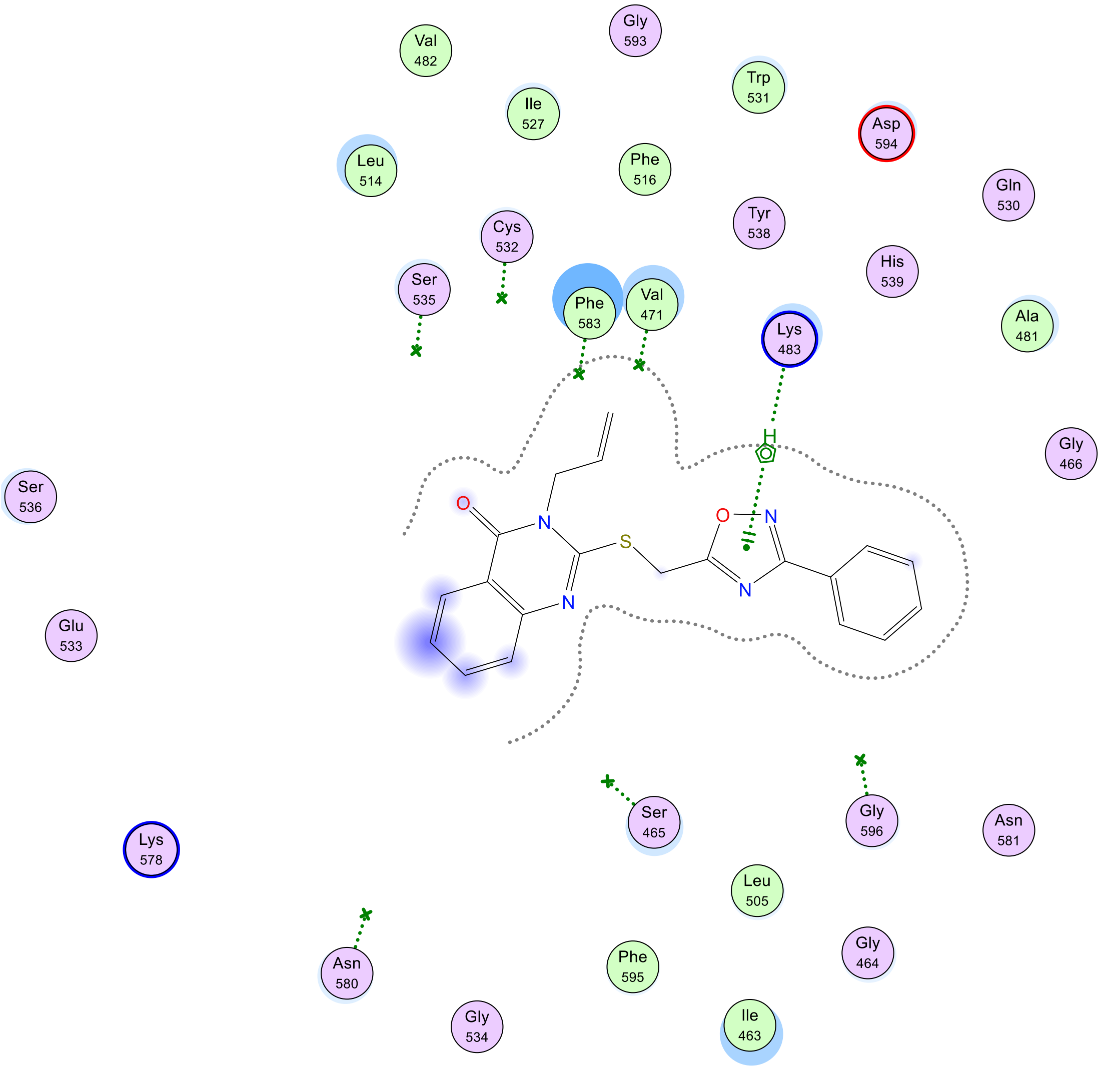

Val 482
Gly 593
Trp 531
Ile 527
Asp 594
Leu 514
Phe 516
Gln 530
Tyr 538
Cys 532
His 539
Ser 535
Phe 583
Val 471
Lys 483
Ala 481
Gly 466
Ser 536
O
N
S
O
N
N
N
Glu 533
Ser 465
Gly 596
Asn 581
Lys 578
Leu 505
Asn 580
Phe 595
Gly 464
Gly 534
Ile 463

9n

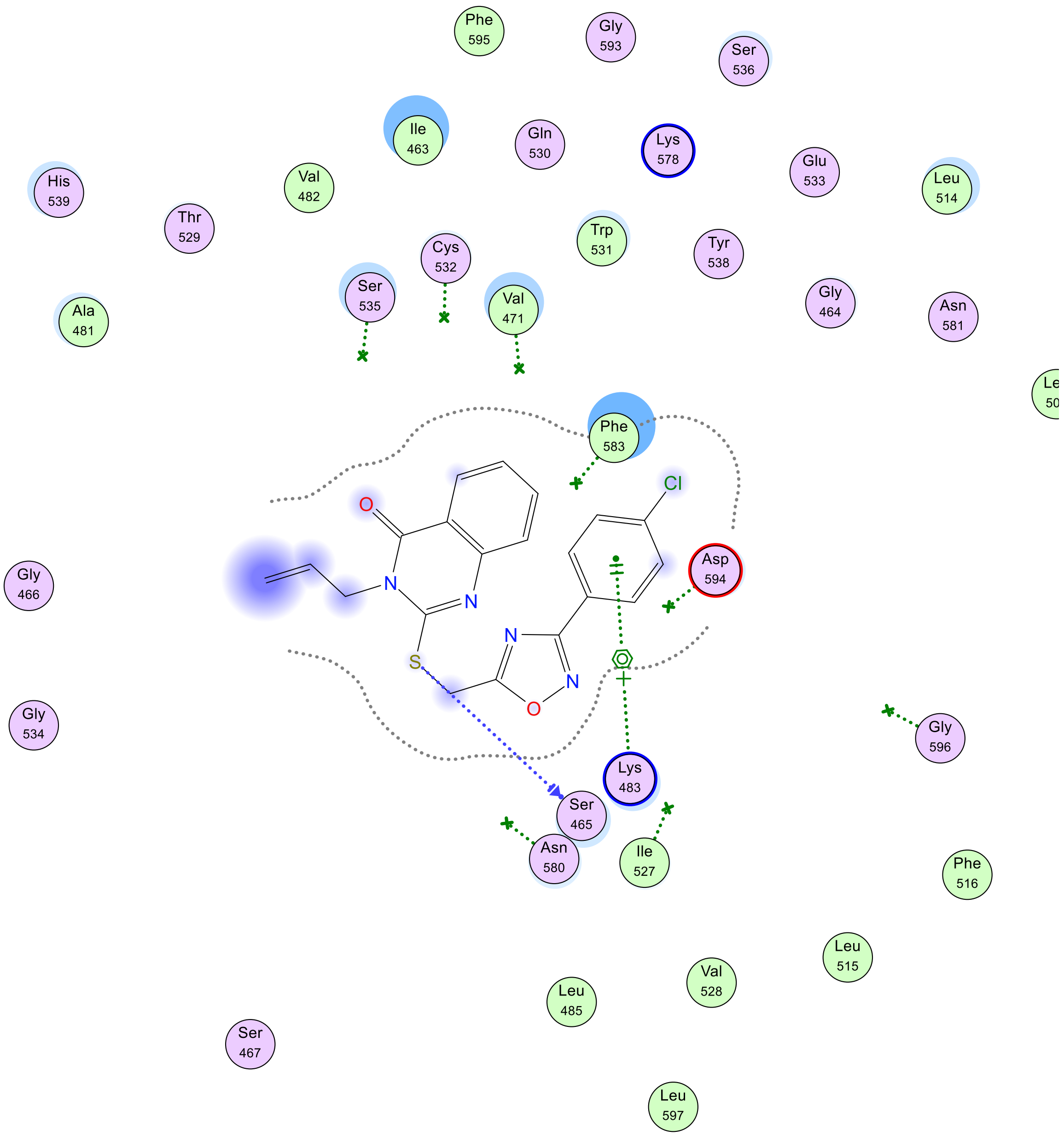

Phe 595
Gly 593
Ser 536
Ile 463
Gln 530
Lys 578
Glu 533
Leu 514
His 539
Val 482
Thr 529
Cys 532
Trp 531
Tyr 538
Gly 464
Asn 581
Ser 535
Val 471
Ala 481
Phe 583
Cl
O
Asp 594
Gly 466
N
N
N
S
N
O
Gly 534
Gly 596
Lys 483
Ser 465
Asn 580
Ile 527
Phe 516
Leu 515
Val 528
Leu 485
Ser 467
Leu 597

9o

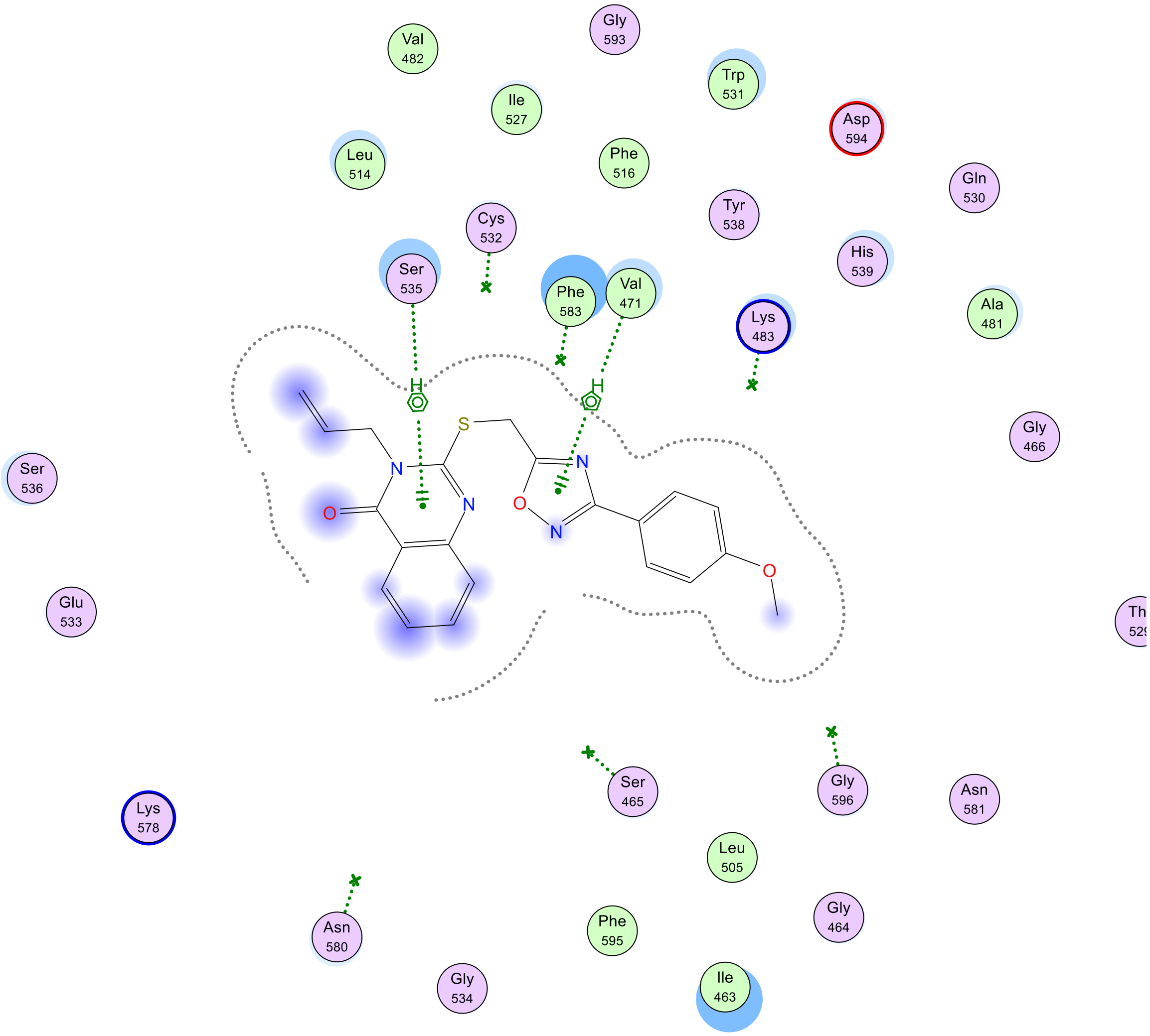

Val 482
Gly 593
Trp 531
Ile 527
Asp 594
Leu 514
Phe 516
Gln 530
Cys 532
Tyr 538
His 539
Ser 535
Phe 583
Val 471
Lys 483
Ala 481
Gly 466
Ser 536
Glu 533
Ser 465
Gly 596
Asn 581
Lys 578
Leu 505
Gly 464
Asn 580
Phe 595
Gly 534
Ile 463

Vemurafe nib

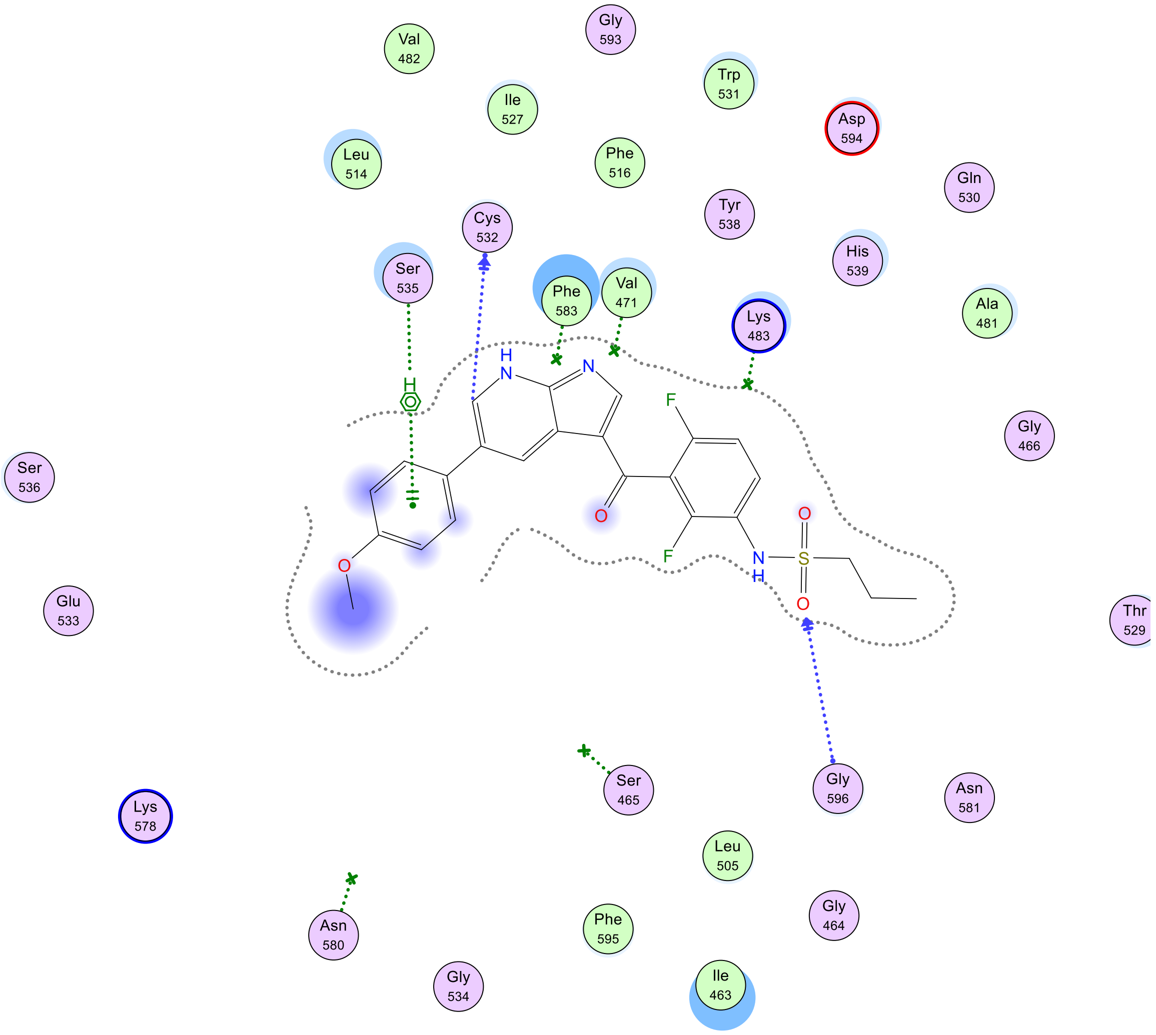

Fig. S.I. 3 Docking poses of derivatives (**9a-o**) within BRAF^{V600E} (PDB ID: 5JRQ) active site showing H-bonding and H-Pi interactions with key amino acid residues.

Appendix A

4. EXPERIMENTAL

4.1. Chemistry

General details

All the reactions progress were monitored with TLC (thin-layer chromatography) on Merck alumina-backed TLC plates and visualized under UV light. All spectral data were measured in DMSO-d_6 on a Bruker AV-300 spectrometer (300 MHz for ^{1}H and 75 MHz for ^{13}C) in the Micro Analytical Center, Cairo University, Egypt. Chemical shifts are expressed in δ (ppm) versus internal Tetramethylsilane (TMS) = 0 ppm for ^{1}H and ^{13}C. Also, the chemical shifts (δ) are reported in parts per million (pm) relative to Tetramethylsilane (TMS) as internal standard, and the coupling constants (*J*) are reported in Hertz (Hz). Splitting patterns are denoted as follows: singlet (s), doublet (d), multiplet (m), triplet (t), quartet (q) and doublet of doublets (dd). Melting points (mp) were determined with a Stuart melting point instrument and are expressed in °C. Elemental analyses were carried out on Perkin device at the Microanalytical Center, Azhar University, Egypt.

4.2. Biological evaluation

4.2.1. Cytotoxic activity using MTT Assay and evaluation of IC_{50}

4.2.1.1. MTT assay

MTT assay was carried out to study the effect of compounds on mammary epithelial cells (MCF-10A). The medium in which cells were propagated contained Dulbecco's modified Eagle's medium (DMEM)/ Ham's F-12 medium (1:1) supplemented with epidermal growth factor (20 ng/mL), hydrocortisone (500 ng/mL), insulin (10 µg/mL), 2 mM glutamine and 10% foetal calf serum. After every 2-3 days, the cells were passaged using trypsin ethylenediamine tetra acetic acid (EDTA). The cells were seeded at a density of 10^4 cells mL^{-1} in flat-bottomed culture plates containing 96 wells each. After 24 h, medium was removed from the plates and the compounds in (in 0.1% DMSO) were added (in 200 µL medium to yield a final concentration of 0.1% v/v) to the wells of plates. A single compound was designated with four wells followed by incubation of plates for 96h at 37°C. After incubation, medium was removed completely from the plates followed by addition of MTT (0.4 mg/mL in medium) to each well and subsequent incubation of plates for 3h. MTT (along with the medium) was removed and DMSO (150µL) was added to each well of the culture plates, followed by vertexing and subsequent measurement of absorbance (at 540 nm) using microplate reader. The data are shown as percentage inhibition of proliferation in comparison with controls containing 0.1% DMSO.

4.2.1.2. Assay for antiproliferative effect

To explore the antiproliferative potential of compounds MTT assay was performed according to previously reported procedure using different cell lines to explore the antiproliferative potential of compounds propidium iodide fluorescence assay was performed using different cell lines. To calculate the total nuclear DNA, a fluorescent dye (propidium iodide, PI) is used which can attach to the DNA, thus offering a quick and precise technique. PI cannot pass through the cell membrane and its signal intensity can be considered as directly proportional to quantity of cellular DNA. Cells whose cell membranes are damaged or have changed permeability are counted as dead ones. The assay was performed by seeding the cells of different cell lines at a density of 3000-7500 cells/well (in 200µl medium) in culture plates followed by incubation for 24h at 37 °C in humidified 5% CO_2/95% air atmospheric conditions. The medium was removed; the compounds were added to the plates at 10 µM concentrations (in 0.1% DMSO) in triplicates, followed by incubation for 48 h. DMSO (0.1%) was used as control. After incubation, medium was removed followed by the addition of PI (25 µl, 50µg/mL in water/medium) to each well of the plates. At -80 °C, the plates were allowed to freeze for 24 h, followed by thawing at 25°C. A fluorometer (Polar-Star BMG Tech) was used to record the readings at excitation and emission wavelengths of 530 and 620 nm for each well. The percentage cytotoxicity of compounds was calculated using the following formula:

$$\% \text{ Cytotoxicity} = \frac{A_C - A_{TC}}{A_C} \times 100$$

Where A_{TC}= Absorbance of treated cells and A_C= Absorbance of control. Erlotinib was used as positive control in the assay.

4.2.1.3. EGFR inhibitory assay

EGFR-TK assay was performed to evaluate the inhibitory potency of the most potent compounds against EGFR. Baculoviral expression vectors including pBlueBacHis2B and pFASTBacHTc were used separately to clone 1.6 kb cDNA coding for EGFR cytoplasmic domain (EGFR-CD, amino acids 645–1186). 5′ upstream to the EGFR sequence comprised a sequence that encoded $(His)_6$. Sf-9 cells were infected for 72h for protein expression. The pellets of Sf-9 cells were solubilized in a buffer containing sodium vanadate (100 µM), aprotinin (10 µg/mL), triton (1%), HEPES buffer (50mM), ammonium molybdate (10 µM), benzamidine HCl (16 µg/mL), NaCl (10 mM), leupeptin (10 µg/mL) and pepstatin (10 µg/mL) at 0°C for 20 min at pH 7.4, followed by centrifugation for 20 min. To eliminate the non-specifically bound material, a Ni-NTA super flow packed column was used to pass through and wash the crude extract supernatant first with 10 mM and then with 100 mM imidazole. Histidine-linked proteins were first eluted with 250 and then with 500 mM imidazole subsequent to dialysis against NaCl (50 mM), HEPES (20 mM), glycerol (10%) and 1 µg/mL each of aprotinin, leupeptin and pepstatin for 120 min. The purification was performed either at 4 °C or on ice. To record autophosphorylation level, EGFR kinase assay was carried out on the basis of DELFIA/Time-Resolved Fluorometry. The compounds were first dissolved in DMSO absolute, subsequent to dilution to appropriate concentration using HEPES (25 mM) at pH 7.4. Each compound (10 µL) was incubated with recombinant enzyme (10 µL, 5 ng for EGFR, 1:80 dilution in 100 mM HEPES) for 10 min at 25°C, subsequent to the addition of 5X buffer (10 µL, containing 2 mM $MnCl_2$, 100 µM Na_3VO_4, 20 mM HEPES and 1 mM DTT) and ATP-$MgCl_2$ (20 µL, containing 0.1 mM ATP and 50 mM $MgCl_2$) and incubation for 1h. The negative and positive controls were included in each plate by the incubation of enzyme either with or without ATP-$MgCl_2$. The liquid was removed after incubation and the plates were washed thrice

using wash buffer. Europium-tagged antiphosphotyrosine antibody (75 µL, 400 ng) was added to each well followed by incubation of 1h and then washing of the plates using buffer. The enhancement solution was added to each well and the signal was recorded at excitation and emission wavelengths of 340 at 615 nm. The autophosphorylation percentage inhibition by compounds was calculated using the following equation:

$$100\% - [(negative\ control)/(positive\ control) - (negative\ control)]$$

Using the curves of percentage inhibition of eight concentrations of each compound, IC_{50} was calculated. Majority of signals detected by antiphosphotyrosine antibody were from EGFR because the enzyme preparation contained low impurities.

4.2.1.4. BRAF kinase assay

V^{600E} mutant BRAF kinase assay was performed to investigate the activity of tested compounds against BRAF. Mouse full-length GST-tagged $BRAF^{V600E}$ (7.5 ng, Invitrogen, PV3849) was pre-incubated with drug (1 µL) and assay dilution buffer (4 µL) for 60 min at 25°C. In assay dilution buffer, a solution (5 µL) containing $MgCl_2$ (30 mM), ATP (200 µM), recombinant human full length (200 ng) and *N*-terminal His-tagged MEK1 (Invitrogen) was added to start the assay, subsequent to incubation for 25 min at 25°C. The assay was stopped using 5X protein denaturing buffer (LDS) solution (5 µL). To further denature the protein, heat (70° C) was applied for 5 min. 4-12% precast NuPage gel plates (Invitrogen) were used to carry out electrophoresis (at 200 V). 10 µL of each reaction was loaded into the precast plates and electrophoresis was allowed to proceed. After completion of electrophoresis, the front part of the precast gel plate (holding hot ATP) was cut and afterwards cast-off. The dried gel was developed using a phosphor screen. A reaction without active enzyme was used as negative control while that containing no inhibitor

served as positive control. To study the effect of compounds on cell-based pERK1/2 activity in cancer cells, commercially available ELISA kits (Invitrogen) were used according to manufacturer's instructions.

4.2.1.6. Cell apoptosis assay

Apoptosis was determined by flow cytometry based on the Annexin-V-fluoresce in isothiocyanate (FITC) and propidium iodide (PI) staining kit (BD Pharmingen, San Diego, USA) [25, 26]. Apoptosis was determined by flow cytometry based on the Annexin-V-fluoresce in isothiocyanate (FITC) and propidium iodide (PI) staining kit (BD Pharmingen, San Diego, USA). Apoptotic cells were defined as Annexin-V-positive. Cells were grown to approximately ~70% confluence and exposed to different concentrations of compounds (0, 2, 4, 6 and8 μmol/L) for 24 h. Treated cells were trypsinzed, washed twice with PBS and transferred into micro centrifuge tubes for centrifugation at1000 rpm for 5 min at room temperature, then resuspended in binding buffer, 5 μL of FITC and PI were added to Eppendorf tube, cells were vortexed, incubated for 15 min at room temperature in dark. Subsequently, cells were analyzed by flow cytometry (Becton Dickinson, Franklin Lakes, and USA).

4.3. Statistical analysis

Computerized Prism 5 program was used to statistically analyzed data using one-way ANOVA test followed by Tukey's as post ANOVA for multiple comparison at $P \leq .05$. Data were presented as mean ± SEM.
