# Supplementary figures and images for: Design and synthesis of new 1,2,4-oxadiazole/quinazoline-4-one hybrids with antiproliferative activity as multitargeted inhibitors
Source: Front Chem. 2024 Aug 30;12:1447618. doi: 10.3389/fchem.2024.1447618 (PMC11393688; doi:10.3389/fchem.2024.1447618)

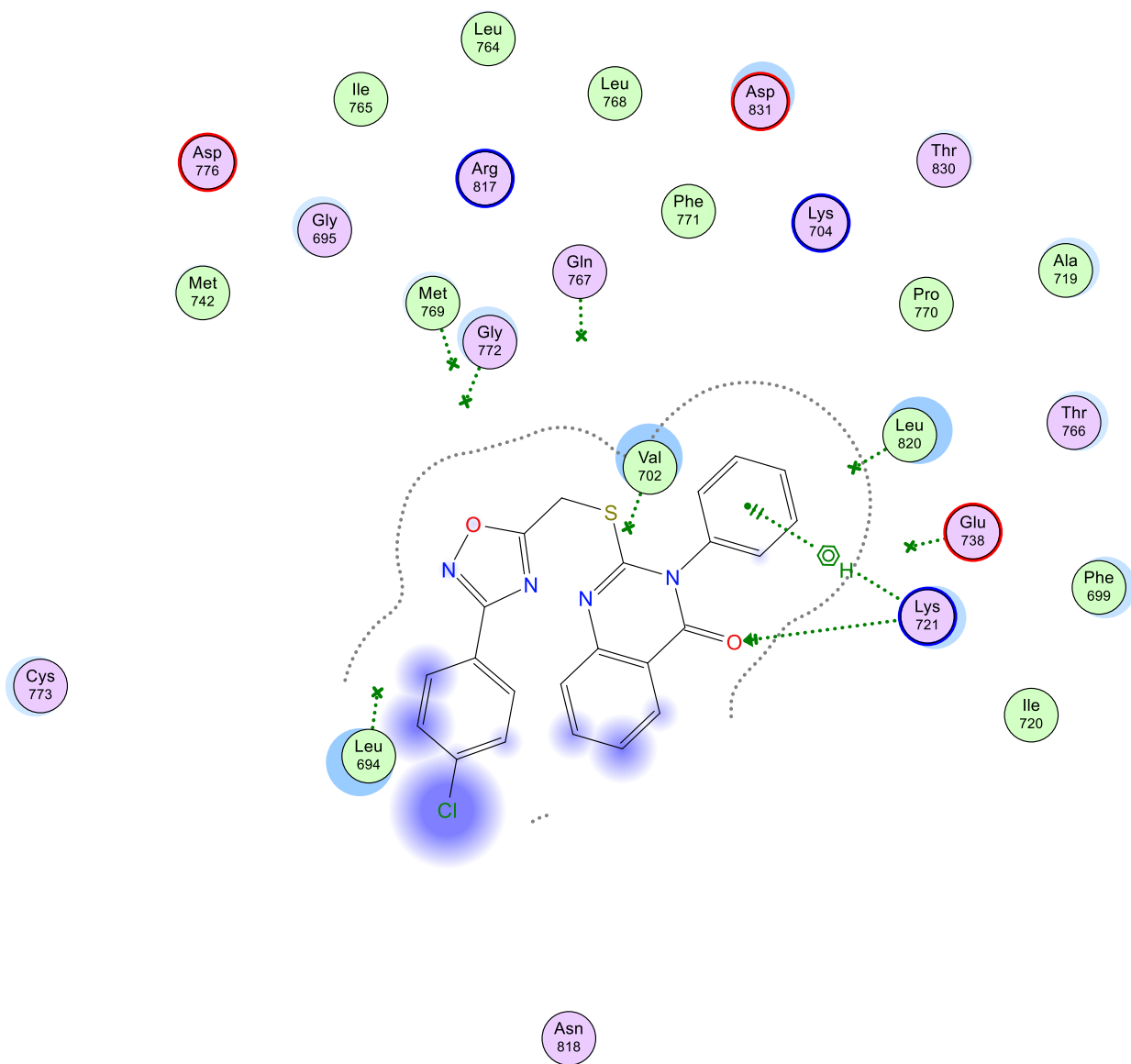

Supplement: Supplementary file 2 [file Image1.pdf]
